# A photo- and cobalt-catalyzed highly selective and divergent hydrofunctionalization of 1,3-dienes with phenols†

**DOI:** 10.1039/d5sc00438a

**Published:** 2025-02-19

**Authors:** Yue Wang, Junhao Miao, Honglin Dong, Dongliang Zhang, Bei Chen, Meihui Guan, Ge Zhang, Qian Zhang

**Affiliations:** a Jilin Province Key Laboratory of Organic Functional Molecular Design & Synthesis, Department of Chemistry, North-East Normal University Changchun 130024 China zhangg492@nenu.edu.cn; b State Key Laboratory of Organometallic Chemistry, Shanghai Institute of Organic Chemistry, Chinese Academy of Sciences Shanghai 200032 China

## Abstract

An applicable cobalt-hydride-mediated selective, divergent hydroetherification and sequential hydroetherification/hydroarylation of 1,3-dienes with simple phenol feedstocks under a photoredox and cobalt catalytic system have been developed. A variety of allyl aryl ethers and value-added chroman derivatives can be obtained in good to excellent yields and stereoselectivity. This method not only obviates the need for extra hydrosilanes and stoichiometric oxidants, thereby offering exceedingly mild conditions for alkene hydroetherification, but also represents the first case of CoH-HAT-catalyzed sequential double hydrofunctionalization of alkenes with a sole nucleophile. The continuous and selective bond-forming catalytic system expands the applications of the cobalt-hydride MHAT reaction and provides a novel approach for the design and synthesis of heterocyclic molecules.

## Abstract 2

Applicable cobalt-hydride-mediated selective, divergent hydroetherification, and sequential hydroetherification/hydroarylation of dienes with simple phenol feedstocks under a photoredox and cobalt catalytic system have been developed.

## Graphical abstract

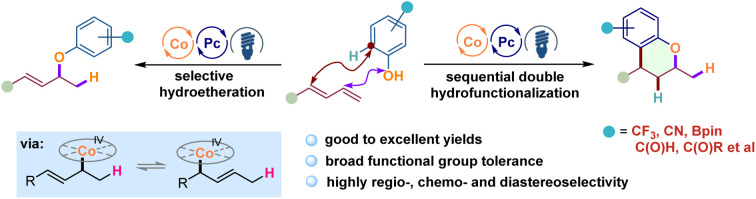

## Introduction

**Scheme 1:**
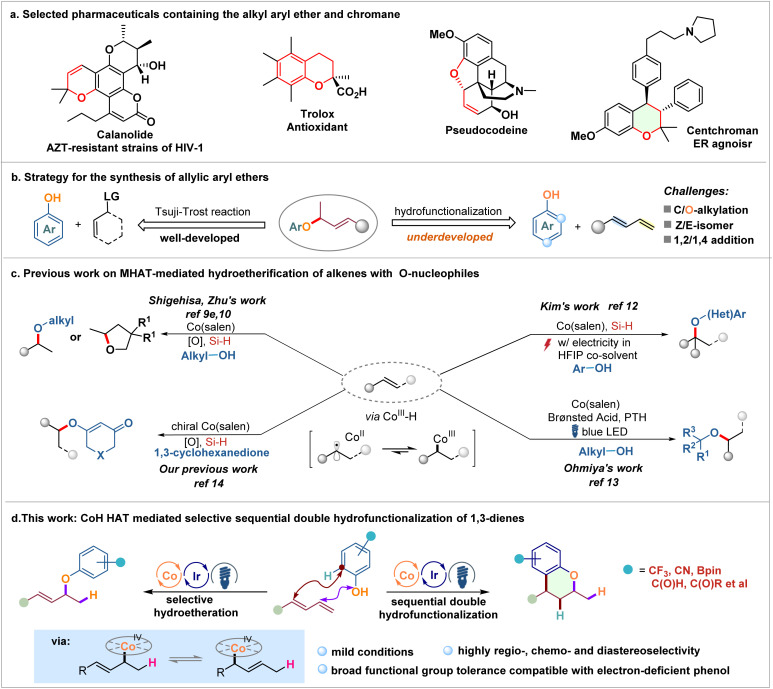
Representative ether-containing compounds and methods for constructing branched allyl aryl ether linkages, as well as the hydrofunctionalization of alkenes with phenols.

Ether linkages are ubiquitous in a myriad of natural products, pharmaceuticals, and agrochemicals (Scheme 1a).^1^ In particular, branched allyl aryl ethers linkages constitute a significant molecular framework that functions as versatile building blocks for a wide range of organic transformations, and various methods have been developed toward their synthesis.^2^ Among them, the palladium-catalyzed Tsuji–Trost reaction represents one of the most powerful and robust methods to prepare these motifs by using allylic electrophiles with oxygen nucleophiles (Scheme 1b, left).^3^ However, accessing pre-functionalized alkenes bearing a leaving group at the allylic position often requires extra synthetic effort. In addition, substituted alkenes, especially internal alkenes, often yield products with unsatisfactory regio- and diastereoselectivity, ultimately limiting the scope of this approach. The direct transition-metal-catalyzed alkene hydroetherification provides a complementary and economical approach using simple and easily available starting materials.^4^ In this regard, various oxygen nucleophiles, including carboxylic acids, alcohols, and ketoximes, have been extensively utilized over the years toward coupling with a variety of alkenes.^5^ However, much less progress has been made with respect to the reactions using simple phenols with 1,3-dienes in catalytic hydroetherification (Scheme 1b, right). This is largely due to the inherent electron-rich structure of phenols, which leads to competitive chemoselectivity between forming the C–O bond (O-allylation)^6^ and the C–C bond (C-allylation) with *ortho* or *para* C–H bonds.^7^ Additionally, conjugated dienes are typically converted into the corresponding 1,2- and/or 1,4-addition products through metal–π-allyl intermediates. Notably, although significant advancements have been made recently in the highly chemo- and regioselective hydrofunctionalization of 1,3-dienes with phenols for C-allylation,^8^ there is a notable scarcity in the selective O-allylation of phenols with conjugated dienes to facilitate the intriguing hydroetherification process, which greatly arouses our interest.

Recently, the emerging cobalt(salen)-catalyzed hydrogen atom transfer (HAT) reactions of alkenes with nucleophiles involving a high-valent alkylcobalt(iv) intermediate have emerged as a versatile platform for alkene hydrofunctionalization (Scheme 1c).^9^ Specifically, by employing Co(salen) catalyst combinations with hydrosilanes and peroxide or *N*-fluoro species, an array of cobalt-catalyzed intra- and intermolecular hydroalkoxylation reactions of alkenes with alcohol nucleophiles have been achieved.^9*e*,10^ This efficient oxidative metal-hydride HAT catalytic system was further developed by Zhu to allow for electrocatalytic oxidative hydroalkoxylation, eliminating the need for stoichiometric chemical oxidants.^11^ In 2022, Kim reported an electrocatalytic hydroetherification of alkenes with phenols, facilitating the modular synthesis of alkyl aryl ethers in high yields.^12^ Meanwhile, Ohmiya demonstrated a novel photoredox/cobalt-catalyzed hydroetherification of alkenes using catalytic amounts of weak Brønsted acid instead of stoichiometric silanes as a hydrogen supply to generate the putative Co(iii)–H species.^13^ These methods provide a convenient and promising synthetic approach for C–O bond construction *via* a radical-polar crossover process. More recently, our research group successfully achieved enantioselective hydroetherification of alkenes by utilizing a chiral Co(salen) catalyst, which performed through a CoH-mediated oxidative metal-hydride hydrogen atom transfer (MHAT) process using symmetric 1,3-diketones as oxygen nucleophiles, thus enabling the synthesis of chiral alkenyl ethers.^14^ Despite these accomplishments, the expansion of this intriguing approach to investigate allyl cobalt(iv) complexes^15^ for selective and divergent hydrofunctionalization of 1,3-dienes with nucleophiles has not yet been established. Our continued interest in Co-catalyzed MHAT reactions^14,16^ has led us to report a photoredox/cobalt-catalyzed radical-polar crossover hydroetherification of dienes with phenol feedstocks (Scheme 1d). Interestingly, a selective sequential hydroetherification/hydroarylation process can also be achieved by prolonging the reaction time, yielding a variety of value-added chroman derivatives^17^ in good to excellent yields and stereoselectivity. This divergent transformation represents the first example of MHAT-mediated selective sequential double hydrofunctionalization to forge radical-involved C–O and C–C bonds in a single operation.

## Results and discussion

We initiated our investigation by subjecting 1-phenyl-1,3-butadiene (1a) and phenol (2a) as model substrates to optimize the hydroetherification reaction conditions under photo- and cobalt dual catalysis (Table 1). The standard conditions included the use of a 1,2-cyclohexdiamine-derived Co(salen) complex (Co-1) as the catalyst, Ir(ppy)_3_ as the photocatalyst, and collidinium triflate (HX-1) as a proton shuttle in DCM at room temperature under 40 W blue LED irradiation, which delivered (*E*)-allylic ether 3a in 85% yield with excellent regioselectivity (>20 : 1 rr) (Table 1, entry 1). Based on previous reports, Ir(ppy)_3_ exhibits a high excited-state reduction potential of *E*_1/2_ [Ir^III*^]/[Ir^IV^] = −1.73 V *vs.* SCE, and its reductively quenched congener possesses a moderate oxidation potential of *E*_1/2_ [Ir^IV^]/[Ir^III^] = 0.77 V *vs.* SCE.^18^ This enables the single-electron reduction of Co(ii) [*E*_red_ (Co-1) = −1.60 V *vs.* SCE] and the single-electron oxidation of alkyl-Co(iii) [*E*_ox_ (Co-1) = 0.00 V *vs.* Fc^+^/Fc],^19^ thereby facilitating the hydroetherification reaction of 1,3-dienes under redox-neutral catalytic conditions. Control experiments demonstrated that light, photocatalyst, the cobalt salen catalyst, and the collidinium ion were all necessary for the reaction (Table 1, entries 2 and 3).

**Table 1:** Optimization of the reaction conditions^a^

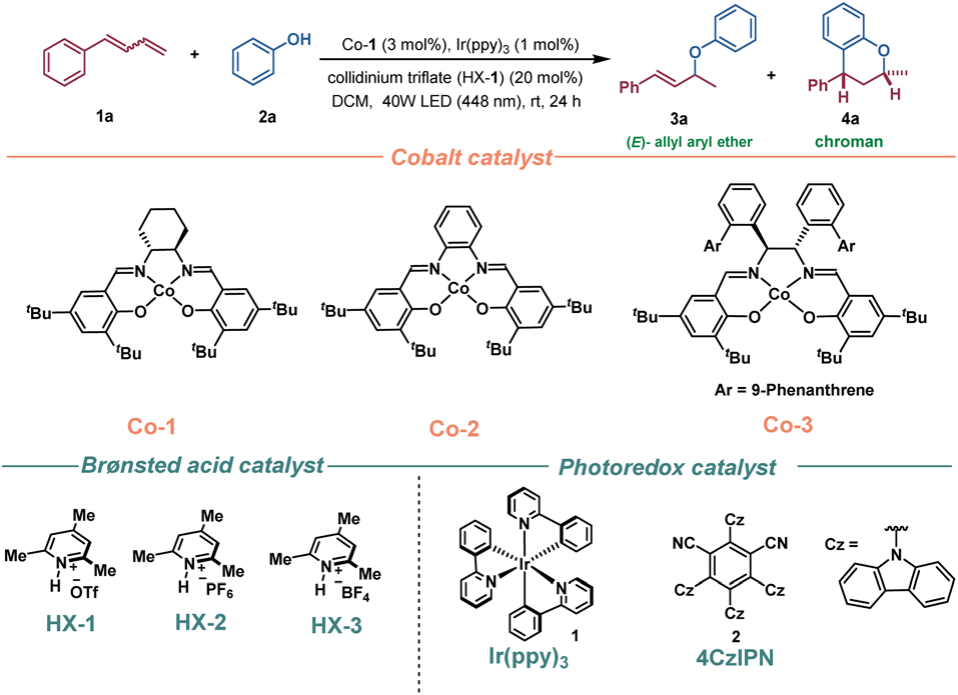			
Entry	Variation of reaction conditions	Yield^b^3a	Yield^b^4a
1	None	85	—
2	w/o light	0	—
3	w/o Ir(ppy)_3_ or Co-1 or HX-1	0	—
4	Co-2 instead of Co-1	78	—
5	Co-3 instead of Co-1	23	—
6	HOTf instead of HX-1	0	—
7	2,4,6-Collidine instead of HX-1	0	—
8	HX-2 instead of HX-1	0	—
9	HX-3 instead of HX-1	0	—
10	4-CzIPN instead of Ir(ppy_)3_	0	—
11	CH_3_CN instead of DCM	23	—
12	48 h instead of 24 h reaction	—	83% (3.3 : 1 dr)^c^
13	2-Chlorophenol (2b) instead of 2a	—	85% (>20 : 1 dr)

a Reaction conditions: the reaction was carried out with 1a (0.4 mmol), 2a (0.2 mmol), photoredox catalyst (0.002 mmol), Co catalyst (0.006 mmol), and Brønsted acid catalyst (0.04 mmol) in DCM (1.0 mL) under 40 W blue LED (448 nm) irradiation for 24 h.

b Yield was determined by ^1^H NMR spectroscopy using CH_2_Br_2_ as an internal standard.

c The values of dr were determined by ^1^H NMR spectroscopy.

The use of cobalt catalyst, Co-2, delivered a 78% yield of product 3a, whereas the utilization of Co-3, resulted in a significant reduction in yield (entries 4 and 5). Using triflic acid, instead of HX-1, failed to deliver the expected product 3a (entry 6). Substituting 2,4,6-collidine for HX-1 resulted in the failure to produce the expected allylic aryl ether 3a, suggesting that the collidinium ion is responsible for Co(i) protonation.^20^ Other collidinium salts, such as HX-1 and HX-2, failed to produce the expected hydroetherification product 3a, likely due to insufficient acidity for protonation of the cobalt(i) species to generate the key CoH necessary for the MHAT process (entries 8 and 9). Additionally, when employing an organic photocatalyst, such as 4CzIPN, no production of 3a was observed (entry 10). Switching the solvent from DCM to MeCN resulted in a significant decrease in yield (entry 11). Furthermore, the observation that using (*E*)-1a, (*Z*)-1a, or a mixture of 1,3-diene 1a (*E*/*Z* = 1 : 1.5) consistently yielded (*E*)-3a indicates that the configuration of the dienes has little effect on reaction efficiency (see ESI Table S3†). Notably, prolonging the reaction time to 48 h led to the observation of sequential double hydrofunctionalization of 1,3-dienes with phenol, resulting in the formation of chroman 4a (83% yield, 3.3 : 1 dr), which are important pharmacophores in medicinal chemistry and biomedical fields (Table 1, entry 12). To our delight, excellent diastereoselectivity (>20 : 1) was observed when substituting 2-chlorophenol for phenol, which delivered the expected chroman derivative 4l in 85% yield (entry 13).

**Scheme 2:**
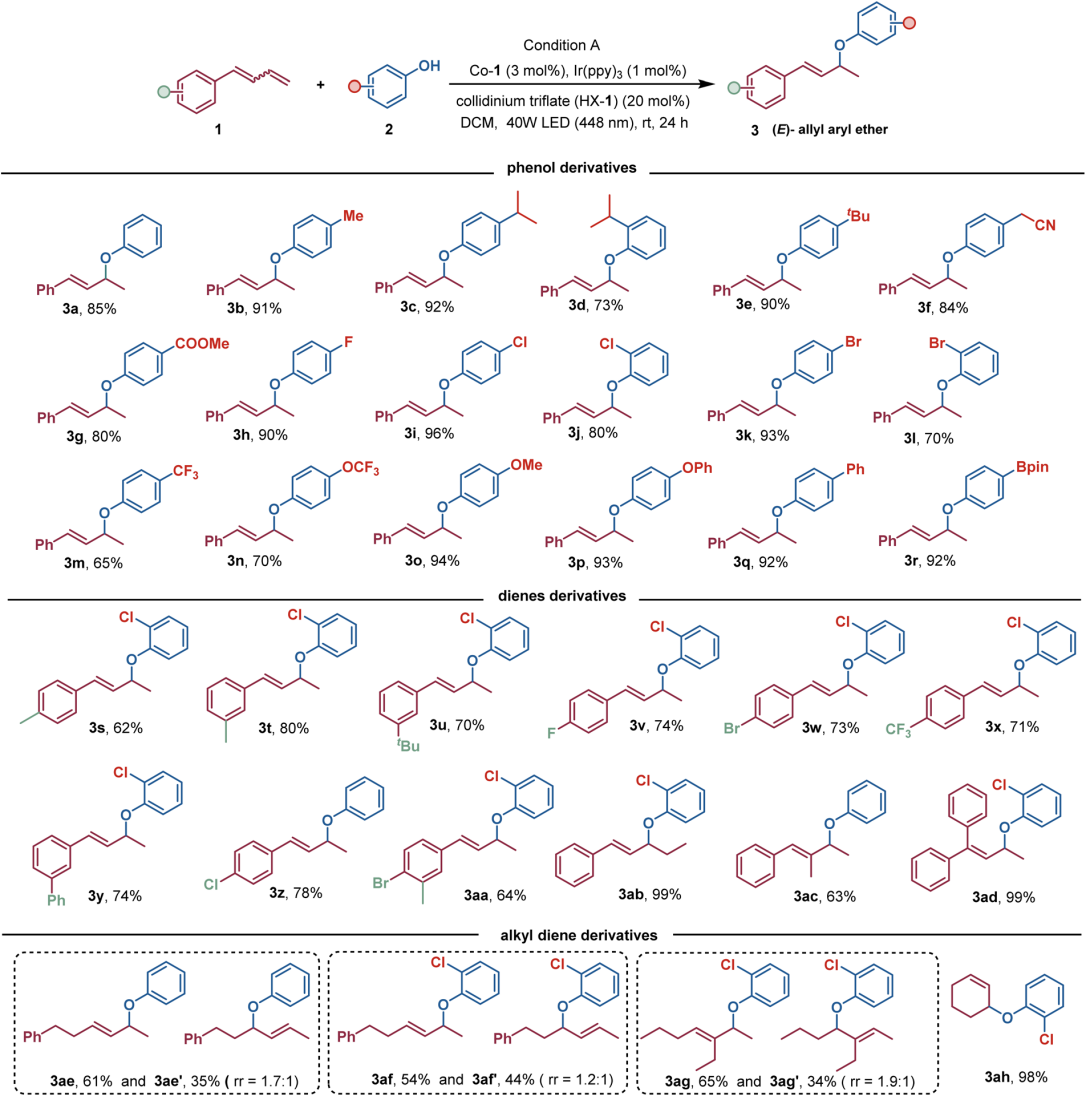
Substrate scope of hydroetherification reaction of 1,3-dienes with phenols. ^*a*^Reaction condition: phenols (0.2 mmol), 1,3-dienes (2.0 equiv.), HX-1 (20 mol%), Ir(ppy)_3_ (1 mol%), and [Co]-1 (3 mol%) in dry dichloromethane (1.0 mL) under 40 W blue LED (448 nm) at room temperature for 24 h. Isolated yields.

With these optimized reaction conditions in hand, we then examined the substrate scope of this photoredox/cobalt-catalyzed hydrofunctionalization. As illustrated in Scheme 2, we first investigated the generality of this method for selective hydroetherification of 1,3-dienes. It was found that an array of phenols bearing a variety of functional groups, including –alkyl, –aryl, –OCH_3_, –CN, –COOMe, –F, –Cl, –Br, –OCF_3_, and –CF_3_, could be effectively alkylated with 1,3-diene 1a to deliver the corresponding (*E*)-allylic aryl ether products in generally good to excellent yields with exclusive regio- and stereoselectivities. The accommodation of boronic acid pinacol ester (3r) and aryl halides (3i–3l) provided more opportunities for further elaborations. In addition, a wide range of 1,3-dienes bearing either electron-donating functional groups or electron-withdrawing functional groups on the aromatic rings were well tolerated under this reaction condition, providing the expected products 3s–3aa in good to excellent yields. Additionally, internal alkenes, as demonstrated for penta-1,3-dien-1-ylbenzene, were also valid substrates for this selective hydroetherification, furnishing the corresponding product 3ab in quantitative yield. Interestingly, conjugated dienes with steric hindrance were also viable to deliver the expected regioselective hydroetherification products 3ac and 3ad in 62% and 99% yield, respectively. These exciting results stimulated us to evaluate whether a wider scope of 1,3-dienes, such as alkyl-substituted alkenes, were amenable to this CoH-mediated hydroetherification. Delightedly, an assortment of alkyl-substituted 1,3-dienes can be efficiently transformed into the desired (*E*)-allyl aryl ether products, 3ae–3ag, in generally favourable yields with simultaneous formation of the competitive 1,4-hydroetherification product. 1,3-Cyclobutadiene can be reacted to produce a sole hydroetherification product 3ah in 98% yield.

**Scheme 3:**
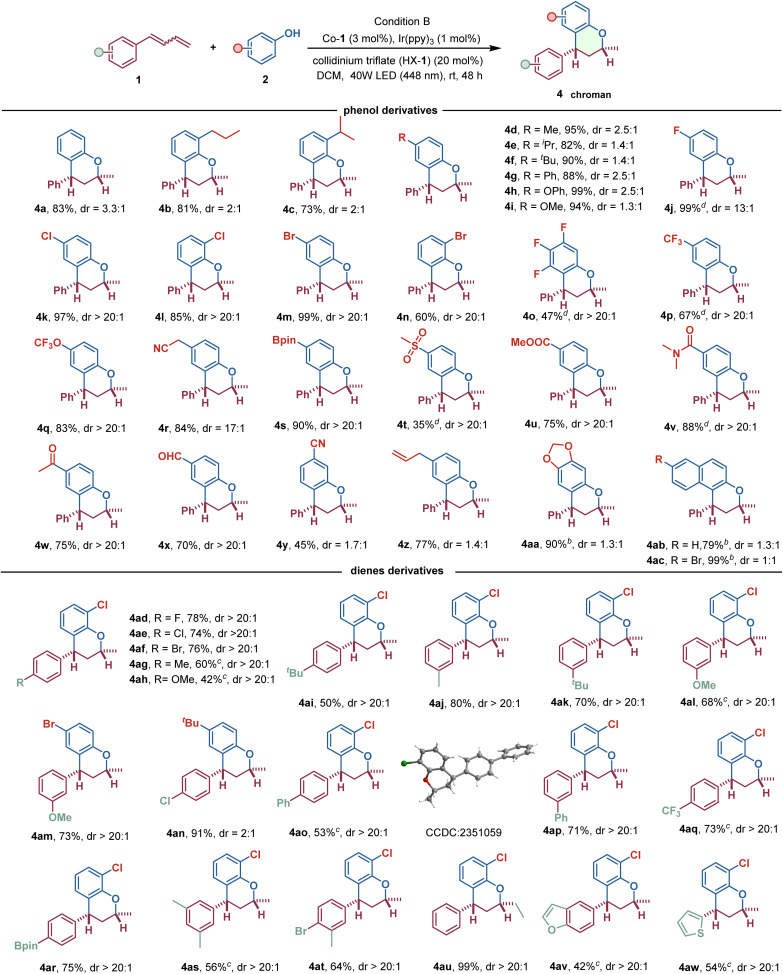
The substrate scope of dihydrofunctionalization reaction of 1,3-dienes with phenols. ^*a*^Reaction condition: phenols (0.2 mmol), 1,3-dienes (2.0 equiv.), HX-1 (20 mol%), Ir(ppy)_3_ (1 mol%), and [Co]-1 (3 mol%) in dry dichloromethane (1.0 mL) under 40 W blue LED (448 nm) at room temperature for 48 h. ^*b*^Reaction conditions: 1,3-dienes (0.2 mmol), phenols (2.0 equiv.), HX-1 (20 mol%), Ir(ppy)_3_ (1 mol%), and [Co]-1 (3 mol%) in dry dichloromethane (1.0 mL) under 40 W blue LED (448 nm) at room temperature for 24 h. ^*c*^4 Å (10 mg) was added. ^*d*^Prolonging the reaction time to 72 h.

Next, we investigated the potential sequence of double hydrofunctionalization reaction of 1,3-dienes with phenols to construct chroman derivatives in a single operation (Scheme 3). To our delight, this catalytic system could be successfully extended to a broad range of 1,3-dienes and phenols, showcasing generally high catalytic efficiency, providing chroman derivatives in good to excellent yields and stereoselectivity. A brief exploration of the phenol substrates revealed that the presence of an electron-withdrawing substituent on the aromatic ring in phenols was beneficial to improve the diastereoselective control in double hydrofunctionalization reaction compared to phenols with electron-donating functional groups (4b–4i*vs.*4j–4x). Hence, several phenols bearing electron-withdrawing substituents, including –F, –Cl, –Br, –CF_3_, –OCF_3_, –CN, –CHO, COMe, –COOMe, –CON(Me)_2_, –SO_2_Me, and –Bpin, reacted with 1a to give the corresponding chroman derivatives in good to excellent yields with up to 20 : 1 diastereoselective control. The high compatibility of this reaction with electron-withdrawing groups significantly enhances the molecular complexity and provides a valuable complement to chroman derivatives that were inaccessible through previous strategies.^17^ Due to the high reactivity of the phenols with electron-donating functional groups (such as alkyl and alkoxyl), the diastereoselective control of the sequential hydroetherification/hydroarylation reaction is relatively poor, and the resulting chroman products have a high yield but with approximately 2 : 1 diastereomeric ratio. In addition, the steric hindrance of substituents in phenols also influences the efficiency of the sequential double hydrofunctionalization of 1a. For instance, the yield of the reaction using *para*-isopropyl-substituted phenol is notably superior to that of *o*-isopropyl-substituted phenol (4c*vs.*4e), which is also demonstrated with the reaction using *p*-Br- and *o*-Br-substituted phenols (4m*vs.*4n). We hypothesize that the discrepancy may be due to steric hindrance caused by the bulky spatial volume at the *ortho*-site of the phenols, which hinders the nucleophilic substitution process of the alkylcobalt(iv) intermediates. The generality of this reaction was further highlighted by the incorporation of aldehyde, allyl, and cyano groups to afford the corresponding products, 4x–4z. The good functional-group tolerance, particularly those susceptible to CoH catalytic systems, offers a lot of opportunities for further chemical transformations. In addition to these simple phenols, electron-rich aromatics, such as sesamol and 2-naphthol, were also viable substrates, all of which can undergo this sequential hydroetherification/hydroarylation reaction to deliver chroman derivatives 4aa–4ac in up to 99% yield, albeit with relatively low diastereocontrol. Interestingly, the diastereocontrol for this selective sequential double hydrofunctionalization process mediated by CoH HAT appears to be less reliant on the electronic and spatial effects of the diene substrates. A broad spectrum of 1,3-dienes with either electron-withdrawing functional groups or electron-donating groups were all viable coupling partners, delivering a variety of chroman derivatives in good to excellent yields with >20 : 1 dr. The position of the substituent on the phenyl ring in 1,3-dienes almost did not alter the diastereoselectivity, as demonstrated with the methyl (4ag, 4aj), tertiary butyl (4ai, 4ak), phenyl (4ao, 4ap), and methoxy (4al, 4am), albeit with discrepancy in product yields. The configuration of the product 4ao was unequivocally determined by single-crystal X-ray diffraction. Additionally, internal alkene could undergo this transformation to afford corresponding chroman 4au in 99% yield and >20 : 1 dr. Heteroaryl-substituted 1,3-dienes were also competent to furnish the products, 4av and 4aw, in moderate yields. However, the alkyl-substituted 1,3-dienes only provided excellent yield of allylic aryl ethers under these reaction conditions, with no sequential double hydrofunctionalization products observed. This may be attributed to the decreased reactivity of the unactivated internal olefin generated after the first hydroetheration, as well as the presumed instability of the alkyl-cobalt intermediates during the second hydrofunctionalization.

Overall, this protocol exhibits excellent tolerance towards a wide range of strong electron-withdrawing substituents, whether in olefin or phenol nucleophiles, representing a significant advancement over traditional Friedel–Crafts strategies for synthesizing chroman derivatives.

**Scheme 4:**
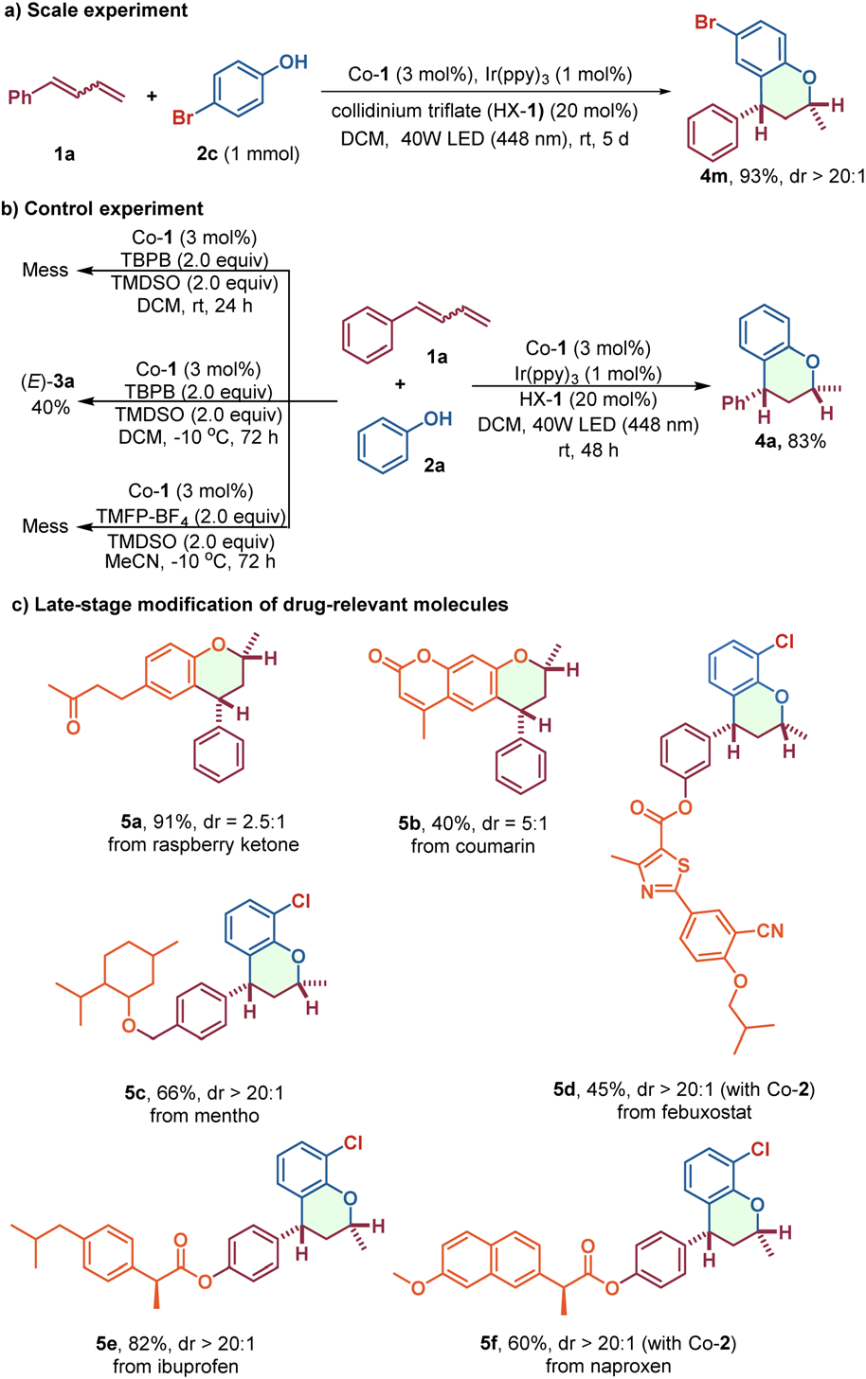
Synthetic applications.

With the aim of further extending the synthetic utility of this methodology, a 1 mmol-scale synthesis was conducted to demonstrate the practicability of this method, and the target chroman 4m was obtained in good yield (Scheme 4a). In addition, a comparison with conventional MHAT reactions using superstoichiometric chemical oxidants highlights the unique advantages of this photoredox/cobalt catalytic system (Scheme 4b). To illustrate, some bioactive molecules, such as raspberry ketone, coumarin, and structurally more complicated menthol, febuxostat, ibuprofen, and naproxen-derived alkenes were conveniently transformed to the corresponding chroman derivatives, 5a–5f, in one step, which demonstrated that this method would be suitable for late-stage functionalization of complex bioactive compounds (Scheme 4c).

**Scheme 5:**
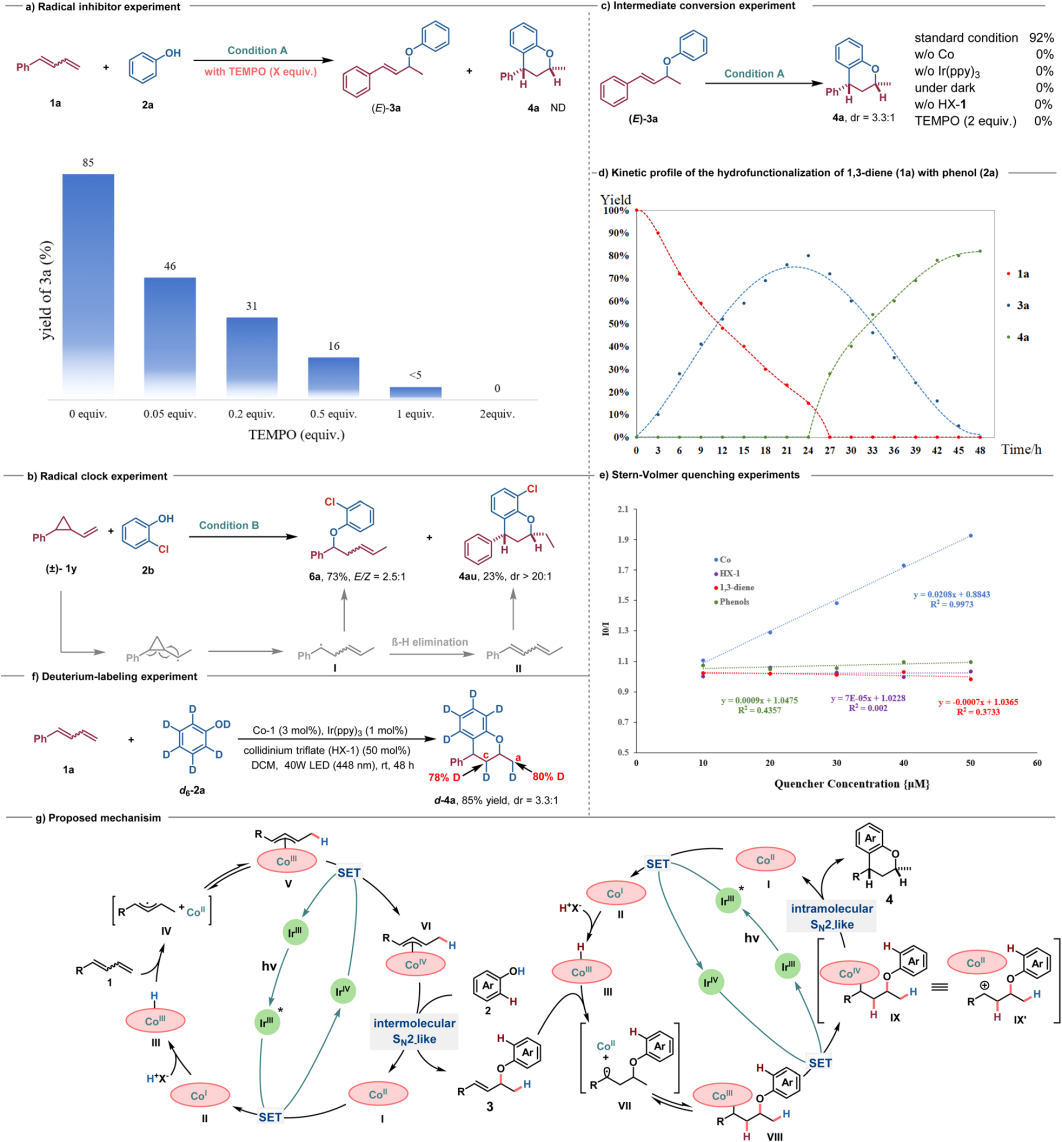
Mechanistic investigation and proposed mechanism (a) radical inhibition experiment. (b) Radical-clock experiment. (c) Intermediate conversion experiment. (d) Kinetic profile of the hydrofunctionalization of 1,3-diene (1a) with phenol (2a). (e) Stern–Volmer quenching experiments. (f) Deuterium-labeling experiment. (g) Proposed mechanism.

To gain some insights into the mechanism of this transformation, several control experiments were performed. As expected, upon the addition of radical inhibitors, such as 2,2,6,6-tetramethyl-1-piperidinyloxy (TEMPO), into the model reaction, the formation of 3a was inhibited (Scheme 5a). In addition, the radical-clock experiment using (2-vinylcyclopropyl)-benzene 1v resulted in the formation of ring-opening/C–O formation product 6a in 73% yield with *E*/*Z* = 2.5 : 1, which may be attributed to the β-hydrogen elimination of alkyl radical intermediate I, leading to the production of 1,3-diene II, followed by a sequential double hydrofunctionalization process resulting in the delivery of 4au (Scheme 5b). These phenomena suggest that a radical intermediate is possibly involved in this transformation, in line with the speculated CoH-mediated HAT process. We also conducted the reaction using isolated 3a as the substrate under standard conditions, resulting in the identical product 4a with a yield of 92% (Scheme 5c). Considering the possibility of Lewis acid or Brønsted acid promoting intramolecular Friedel–Crafts acylation of 3a to form 4a,^21^ a series of control experiments were performed. The results showed that light, cobalt catalyst, photosensitizer, and Brønsted acid are all essential for the formation of 4a (Scheme 5c). To gain further insights into the dynamic progress of the reaction, we monitored the amounts of 1a, 3a, and 4a over time (Scheme 5d). Within the initial 24 h, there was a gradual accumulation of product 3a, with almost undetectable levels of 4a. The highest yield of 3a was achieved at 24 h. Subsequently, there was a rapid consumption of 3a over the following 24 h, gradually converting it to 4a until completion. The Stern–Volmer quenching experiments further indicated that photoexcited Ir(iii)* could be quenched by the Co catalyst rather than by HX-1, 1,3-dienes, or phenols (Scheme 5e). Additionally, a deuterium-labeled experimental reaction of 1a using phenol-*d*_6_ (*d*_6_-2a) as the nucleophile was conducted, yielding *d*-4a in 85% yield with a 3.3 : 1 dr, and 80% and 78% deuterium incorporation into carbons C(a) and C(c) of the product, respectively (Scheme 5f). These results demonstrated that proton transfer occurred from the O–D group of *d*_6_-phenol to the 1,3-dienes in both hydrofunctionalization processes. The deuterium incorporation of no more than 80% suggested that HX-1 serves as the proton shuttle in the Co(iii)H-mediated MHAT process, with part of the hydrogen source coming from HX-1 itself.

Based on these experimental results and literature precedents, a possible catalytic cycle for this photoredox/cobalt-catalyzed selective mono- and dihydrofunctionalization is shown in Scheme 5g. Under visible light irradiation, the Ir(iii) catalyst transitions to the excited state Ir(iii)*, bearing high reducing ability, which undergoes a single electron transfer (SET) to SalenCo(ii) to form SalenCo(i) and Ir(iv) species.^9*d*,13^ The low-valent Co(i) then reacts with a proton in collidinium triflate (HX-1) to form the putative Co(iii)–H species III.^22,23^ Subsequently, a Co(iii)–H mediated MHAT to alkenes could yield a metallic cobalt(ii) and an allylic radical pair IV, which are in fast equilibrium with allylcobalt(iii) species V.^9*a*,24–28^ A further SET oxidation of allylcobalt(iii) species V by Ir(iv) generates the pivotal allylcobalt(iv) species VI, which undergoes an intermolecular S_N_2-substitution reaction with the phenol nucleophile to afford the expected allyl aryl ether 3 and regenerate Co(ii), thus closing the catalytic cycle.^15,29–34^ Thereafter, allyl aryl ether 3 could undergo a similar cobalt(iii)H-mediated MHAT process by participating in an intramolecular nucleophilic substitution reaction involving benzylcobalt(iv) species IX with phenol as the carbon-based nucleophile for C–C bond construction to deliver chromans 4. Given that Co-MHAT catalysis for alkene substrates involving cation-stabilizing groups (such as benzylic or tertiary carbocations) has proposed a carbocation-Co(ii) ion pair alongside Co(iv)-alkyl species,^19,35^ the possibility of direct cyclization *via* carbocation IX′ during the second nucleophilic cyclization cannot be excluded.

## Conclusions

By exploiting a photoredox catalysis combined with Co catalysis, we have accomplished the first highly selective, divergent mono- and dihydrofunctionalization of simple 1,3-dienes with phenols, thereby enabling an efficient and alternative strategy to access allylic aryl ethers and valuable chroman derivatives *via* Co(iii)H-mediated MHAT, followed by S_N_2-substitution of key alkyl-Co(iv) with nucleophiles. This radical-based strategy features high atom economy; broad functional group tolerance; high regio-, chemo-, and diastereoselectivity; and enables the installation of electron-poor groups that are typically not compatible with previous Friedel–Crafts reactions. Meanwhile, this reaction can be used in the last-stage functionalization of complex bioactive compounds. The continuous and selective formation of C–O and C–C bonds expands the application range of cobalt-hydride MHAT reaction and offers new opportunities for the future design and synthesis of heterocyclic molecules. Further investigations on the enantioselective version and the development of new cobalt catalytic systems and their application in selective, divergent MHAT hydrofunctionalization are underway in our laboratory.

## Data availability

All data supporting the findings of this study including the experimental procedures and characterization of the compounds are available within the article and its ESI.† Crystallographic data for compound 4ao has been deposited at the CCDC under [CCDC 2351059] and can be obtained free of charge from the Cambridge Crystallographic Data Centre *via*https://www.ccdc.cam.ac.uk/data_request/cif.

## Author contributions

G. Z. and Q. Z. directed the project. G. Z. conceived the idea, designed the experiments, and wrote the manuscript draft. Y. W. performed the experiments, analyzed the data, and prepared the ESI.† J. M. performed part of the hydroetherification reaction. H. D., D. Z., B. C., and M. G. helped synthesize some substrates and repeated the reactions. All authors participated in the discussion and preparation of the manuscript.

## Conflicts of interest

There are no conflicts to declare.

## Supplementary Material

SC-OLF-D5SC00438A-s001

SC-OLF-D5SC00438A-s002
