# Supplementary material for: A photo- and cobalt-catalyzed highly selective and divergent hydrofunctionalization of 1,3-dienes with phenols
Source: Chem Sci. 2025 Feb 19;16(13):5640–50. doi: 10.1039/d5sc00438a (PMC11873908; doi:10.1039/d5sc00438a)
Supplement: SC-016-D5SC00438A-s001 [file SC-016-D5SC00438A-s001.pdf]

Supplementary Information (SI) for Chemical Science.
This journal is © The Royal Society of Chemistry 2025

Supporting Information

Table of Contents

I. General Information……………………………………………….…S2

II. The Preparation of Substrates……………………………………S3

III. Optimization of Reaction Conditions……………….……….…...S5

IV. General Procedures…....…………………….……..……...…S10

V. Analytical Data of Products……………………………………....S12

VI. Structure Analysis X-Ray Crystallography of 4ao…………….S35

VII. Mechanistic Experiments……………………...…………….S37

VIII. Reference……………………………………………...…….S46

IX. NMR spectra…………………………………………….…S47

I. General Information

All reactions were performed under nitrogen atmosphere in flame dried flasks. All reactions were monitored by thin layer chromatography (TLC) using Macherey-Nagel 0.20 mm silica gel 60 plates. Flash column chromatography was performed on silica gel 60 (particle size 300-400 mesh ASTM, purchased from Taizhou, China). ^{1}H, ^{13}C, ^{19}F spectra were recorded with Varian 500 MHz (Inova-500) or Bruker 600 MHz (Avance-600) instrument. All ^{1}H NMR data are reported in δ units, parts per million (ppm), and were measured relative to the residual proton signal in the deuterated solvent at 7.26 ppm ($CDCl_3$). All ^{13}C NMR spectra are decoupled and reported in ppm relative to the solvent signal at 77.00 ppm ($CDCl_3$). The data is being reported as (s = singlet, d = doublet, dd = doublet of doublet, t = triplet, m = multiplet or unresolved, coupling constant(s) in Hz, integration). High-resolution mass spectra HRMS (ESI-TOF) were recorded on Brucker microtof. Compounds were visualized by irradiation with UV light or stained with iodine/silica gel or potassium permanganate. Preparatory thin-layer chromatography (Prep-TLC) was performed on silica gel GF with UV 254 (20 × 20 cm, 1000 microns, from Yantai Jiang you Silica Gel Development Co., Ltd.) and visualized with UV light. Photochemical reactions were performed utilizing a 40w LED light (448 nm). Solvents CH_2Cl_2 was distilled over CaH_2 and stored under nitrogen atmosphere. All other commercial reagents and solvents were purchased from Energy-Chemical Ltd, and used as received unless otherwise noted. Alkene substrates are known compounds and were prepared by previously reported procedures.[1-4] Brønsted acid catalysts are known compounds and were prepared by previously reported procedures.[5]

II. The Preparation of Substrates

1. General Procedure for the Synthesis of alkene.

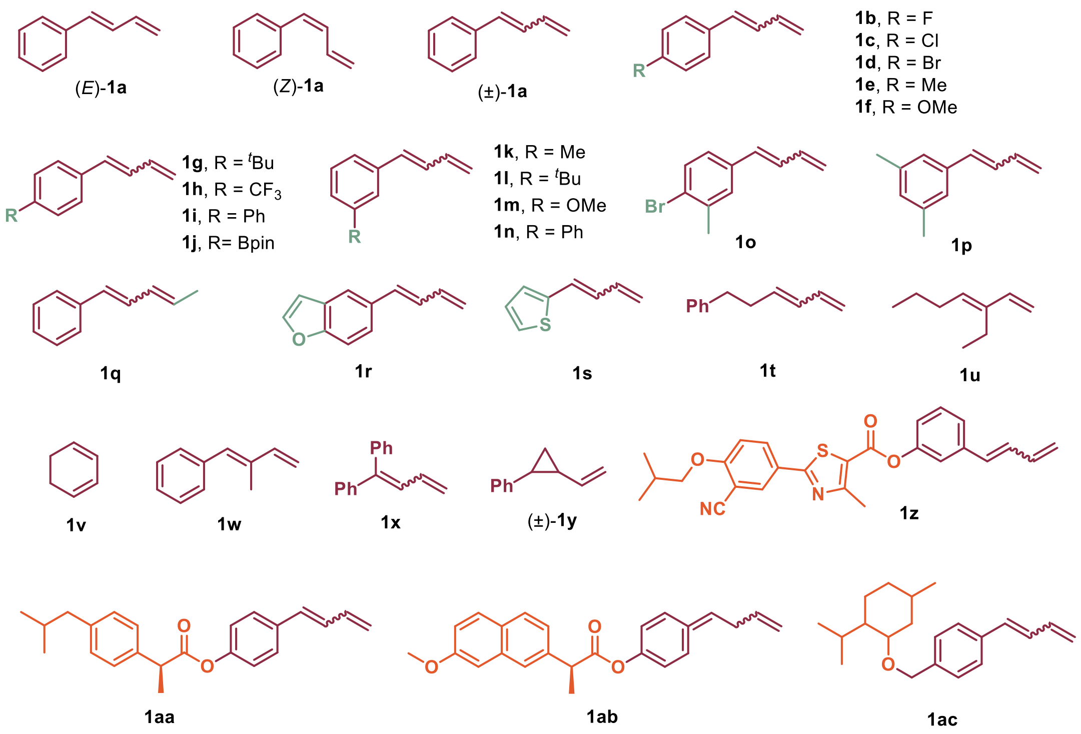

Fig. S1. Alkene used in this transformation.

Diene **1z** is commercially available.

Dienes **(±)-1a**, **1b ~ 1t**, **1x, 1z ~ 1ac** were synthesized via Wittig reaction using allyltriphenylphosphonium bromide according to the known procedures.[1]

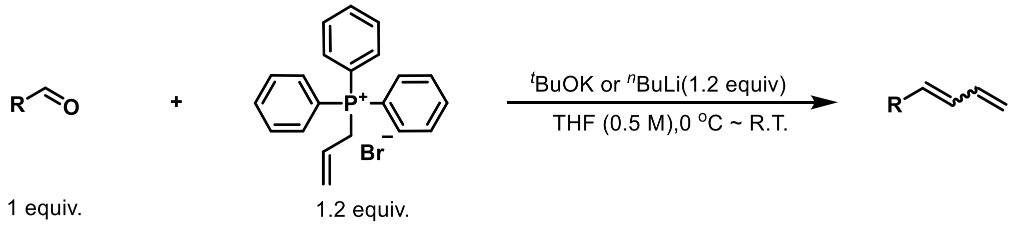

Dienes (*E*)-**1a**, **1u** and **1w**, were synthesized via Wittig reaction using methyltriphenylphosphonium bromide.[2]

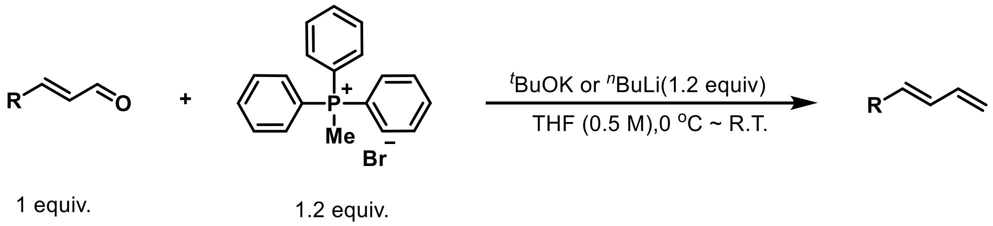

(±)-**1y** are known compounds and were prepared by previously reported procedures.[3]

(*Z*)-**1a** are known compounds and were prepared by previously reported procedures.[4]

2. Commercially Materials

The following known starting materials (phenols) were commercially available and used without further purifications:

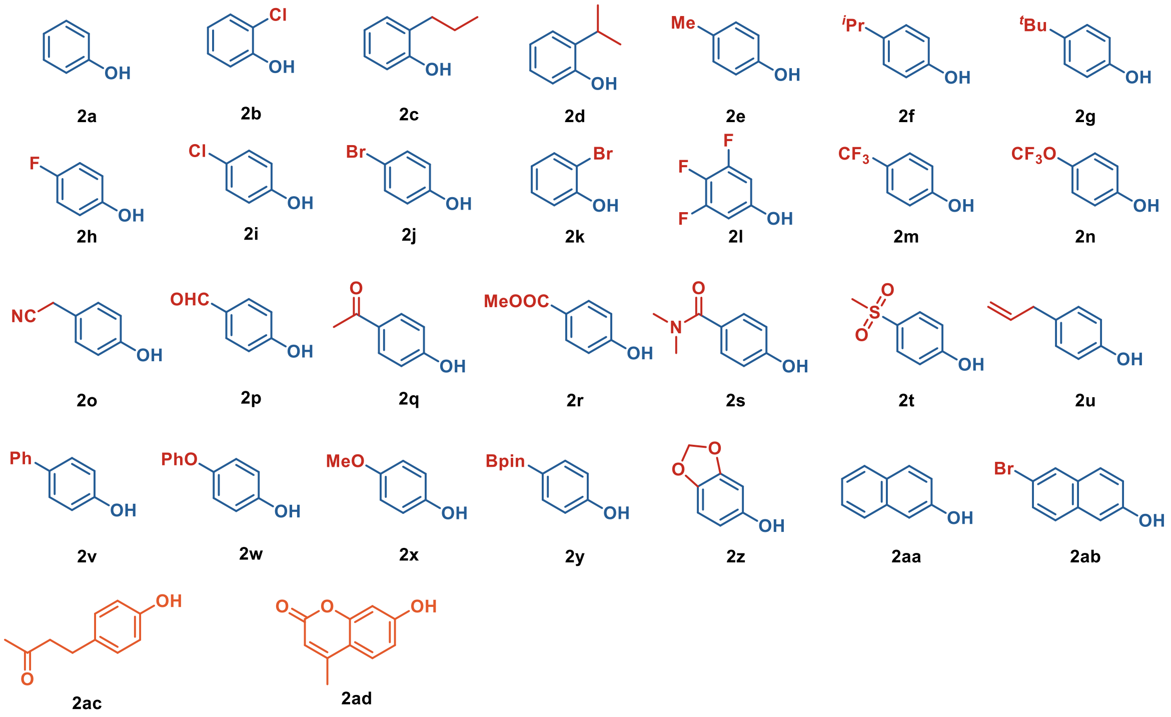

Fig. S2. The commercially available phenols used in this transformation.

III. Optimization of Reaction Conditions

Table S1. The screening of solvents and photocatalyst.[a]

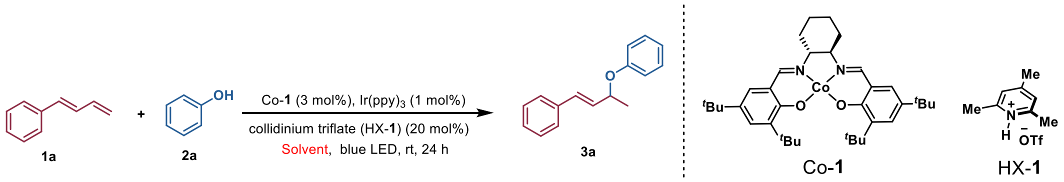

Entry	Solvents	Photocatalyst	Wavelength (nm)	Yield(%)[b]
1	DCM	$Ir(ppy)_3$	448	76%
2	DCE	$Ir(ppy)_3$	448	52%[c] (12%[d])
3	THF	$Ir(ppy)_3$	448	13%
4	Tol	$Ir(ppy)_3$	448	< 5%
5	Tol-CF_3	$Ir(ppy)_3$	448	< 5%
6	EA	$Ir(ppy)_3$	448	< 5%
7	dioxane	$Ir(ppy)_3$	448	< 5%
8	CCl_3H	$Ir(ppy)_3$	448	10%
9	MeCN	$Ir(ppy)_3$	448	23%
10	DCM	4-CzIPN	448	0%
11	DCM	4-CzIPN	410	0%

[a]Reaction conditions: **1a** (0.2 mmol), **2a** (3.0 equiv), Co-**1** (3 mol%), $Ir(PPy)_3$ (1 mol%), collidinium triflate (HX-**1**) (20 mol%), solvent (0.2 M). [b]Yield determined by ^{1}H NMR spectroscopy using CH_2Br_2 as an internal standard. [c]Using simple and undried dichloroethane (DCE) as solvent. [d]Using commercially available DCE. THF = Tetrahydrofuran. DME = 1,2-Dimethoxyethane. DCM = Dichloromethane. DCE = 1,2-Dichloroethane. Tol = Toluene. Tol-CF_3 = Benzotrifluoride. EA = Ethyl acetate.

Note: no target product has been observed from coarse ^{1}H NMR under the conditions of entry 11

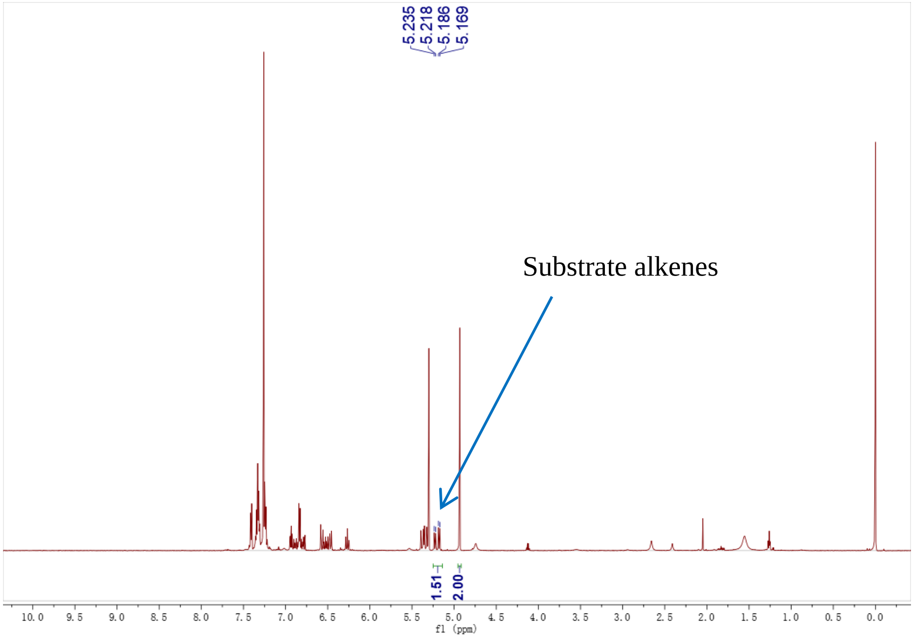

Fig. S3 ^{1}H NMR data of entry 11

Table S2. The screening of scale.[a]

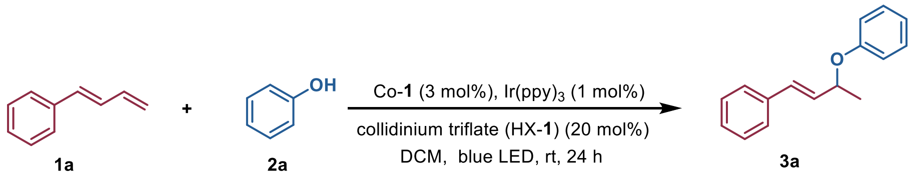

Entry	1a:2a	Yield[b]
1	1:3	76%
2	3:1	80%
3	2:1	85%

[a]Reaction conditions: **1a**, **2a**, Co-**1** (3 mol%), Ir(PPy)$_3$ (1 mol%), collidinium triflate (HX-**1**) (20 mol%), DCM (0.2 M). [b]Yield determined by ^{1}H NMR spectroscopy using CH_2Br_2 as an internal standard.

Table S3. The effect of olefin configuration on reaction [a]

Entry	1a	Yield[b]
1	(*E*)-**1a**	85%
2	(*Z*)-**1a**	84%
3	(*Z*/*E*)-**1a** mixture	85%

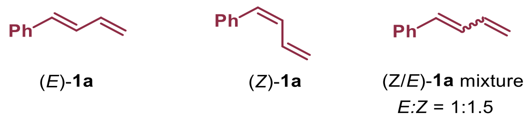

[a]Reaction conditions: **1a** (2.0 equiv), **2a** (0.2 mmol), **Co.cat** (3 mol%), Ir(PPy)$_3$ (1 mol%), collidinium triflate (HX-**1**) (20 mol%), DCM (0.2 M). [b]Yield determined by ^{1}H NMR spectroscopy using CH_2Br_2 as an internal standard.

(Z)-buta-1,3-dien-1-ylbenzene

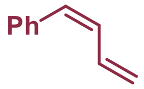

^{1}H NMR (600 MHz, CDCl$_3$) δ 7.36 – 7.30 (m, 4H), 7.25 (d, *J* = 4.8 Hz, 1H), 6.93 – 6.84 (m, 1H), 6.46 (d, *J* = 11.4 Hz, 1H), 6.26 (t, *J* = 11.4 Hz, 1H), 5.37 (d, *J* = 16.8 Hz, 1H), 5.22 (d, *J* = 10.2 Hz, 1H). **^{13}C NMR (151 MHz, CDCl$_3$)** δ 137.4, 133.2, 130.8, 130.4, 129.0, 128.2, 127.0, 119.6. **HRMS** (ESI-TOF) (m/z): calcd for $C_{10}H_{10}Na$ ([M+Na]$^+$), 153.0675, found, 153.0671.

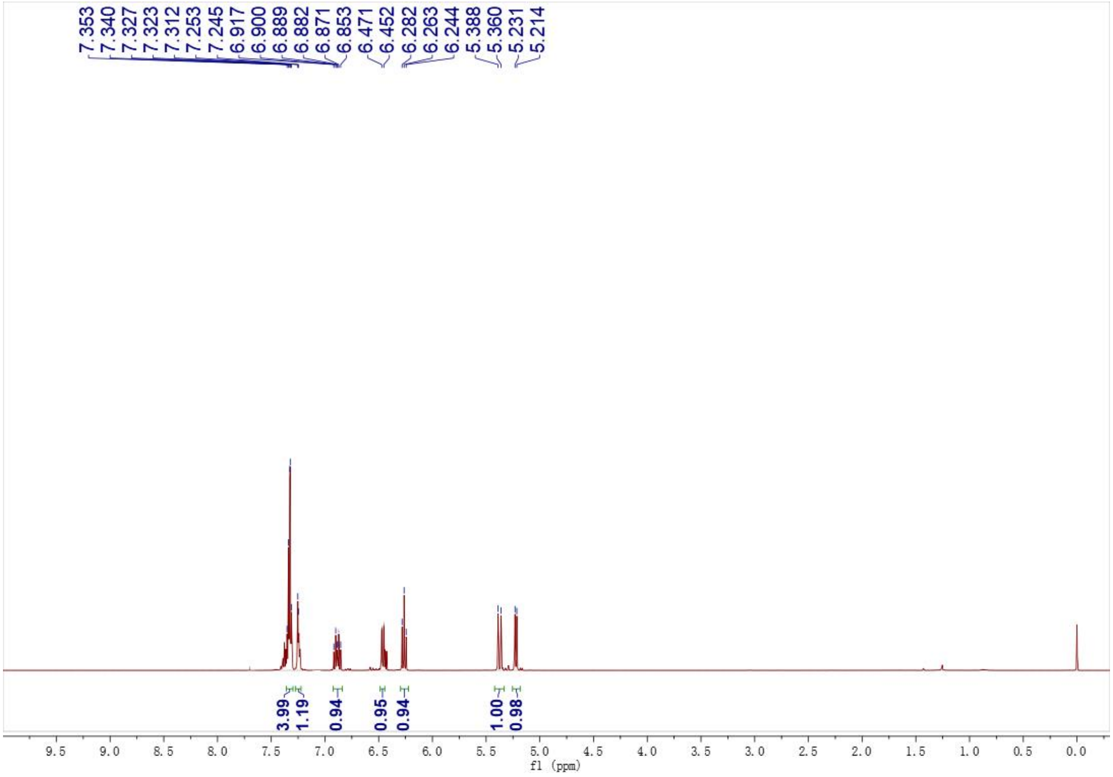

Fig. S4 ^{1}H NMR data of (*Z*)-1a

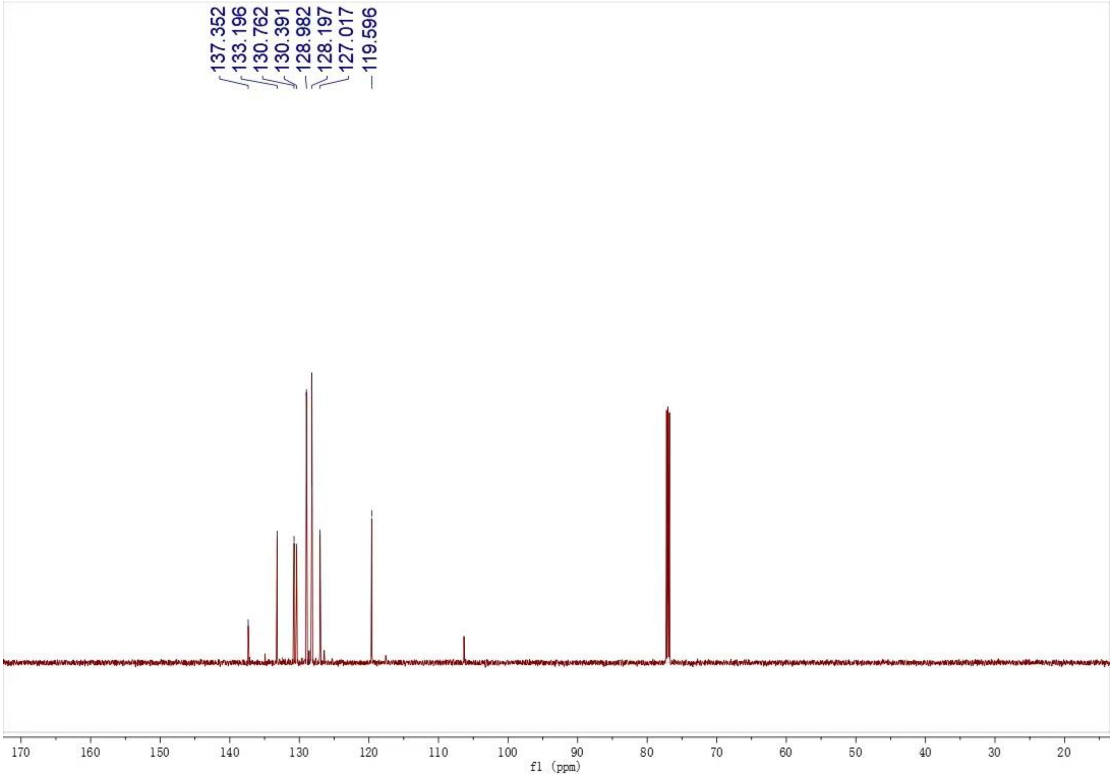

Fig. S5 ^{13}C NMR data of (*Z*)-1a

Table S4. The screening of Co. catalysts for Co(III)H-catalyzed sequence double hydrofunctionalization reaction.

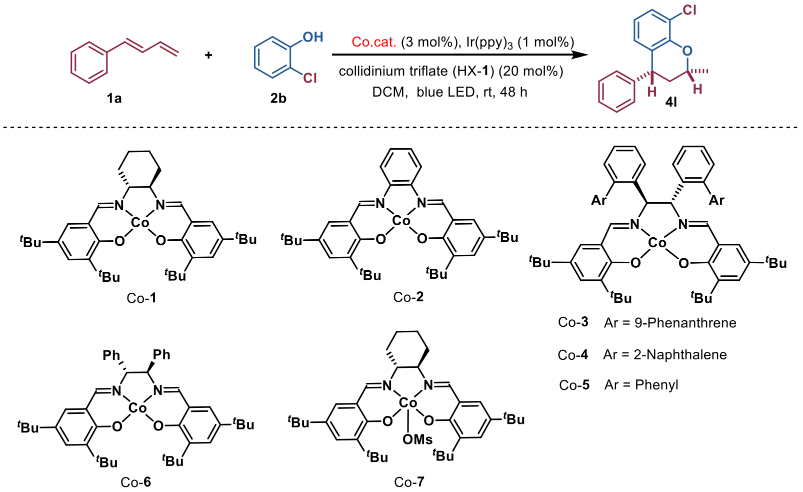

Entry[a]	Co.cat	Yield 4l[b]	Dr. 4l[c]	Er. 4l[d]
1	Co-**1**	85%	>20:1	49:51
2	Co-**3**	41%	>20:1	52:48
3	Co-**4**	36%	>20:1	53:47
4	Co-**5**	62%	>20:1	52:48
5	Co-**6**	62%	>20:1	47:53
6	Co-**7**	64%	>20:1	49:51
7[e]	Co-**6**	62%	3.3:1	n.d.

[a]Reaction conditions: **1a** (2.0 equiv), 2-chlorophenol **2b** (0.2 mmol), **Co.cat** (3 mol%), Ir(PPy)$_3$ (1 mol%), collidinium triflate (HX-**1**) (20 mol%), DCM (0.2 M). [b]Yield determined by ^{1}H NMR spectroscopy using CH_2Br_2 as an internal standard. [c]The values of dr were determined by ^{1}H NMR spectroscopy. [d]The values of er were determined by chiral HPLC analysis. [e]Using phenol **2a** instead of **2b**. n.d. = no detection.

Table S5. The screening of Co. catalysts for Co(III)H-catalyzed hydroetherification.

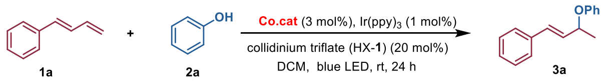

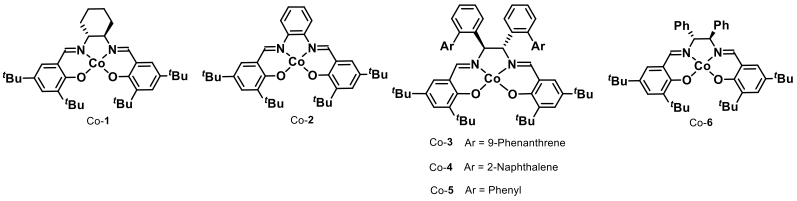

Entry[a]	Co.cat	Yield 3a[b]	EE. 3a[d]
1	Co-**1**	76%	1%
2	Co-**3**	41%	2%
3	Co-**4**	38%	2%
4	Co-**5**	37%	2%
5	Co-**6**	30%	2%

[a]Reaction conditions: **1a** (0.2 mmol), **2a** (3.0 equiv), **Co.cat** (3 mol%), Ir(PPy)3 (1 mol%), collidinium triflate (HX-**1**) (20 mol%), DCM (0.2 M). [b]Yield determined by ^1H NMR spectroscopy using CH_2Br_2 as an internal standard. [c]The values of dr were determined by ^1H NMR spectroscopy. [d]The values of ee were determined by chiral HPLC analysis.

IV. General Procedures

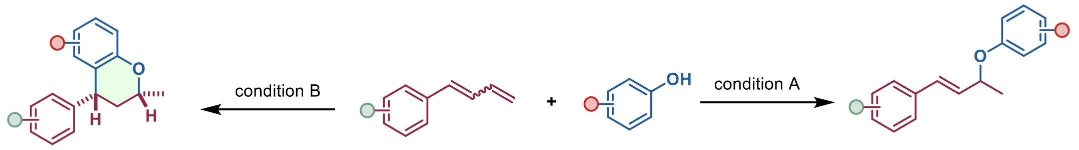

Condition A:

In a glovebox, an oven-dried vial with a stirring bar was charged with photoredox catalyst Ir(ppy)$_3$ (1.3 mg, 0.002 mmol), Co-**1** (3.6 mg, 0.006 mmol), HX-**1** (10.8 mg, 0.04 mmol) and DCM (1.0 mL). Subsequently, phenol derivative **2** (0.2 mmol) and 1,3-dienes derivatives **1** (0.4 mmol) were added to the reaction mixture successively. The vial was then sealed and removed from the glove box for stirring and irradiation with a 40W blue LED (448 nm) equipped with a cooling fan to maintain the temperature at approximately 40 ^{o}C. After 24 hours, the reaction mixture was transferred to a separatory funnel and quenched with saturated $NaHCO_3$ solution followed by extraction with DCM (3×5 mL). The combined organic layer was washed with brine solution and dried over Na_2SO_4. The crude product was obtained after removal of solvent. Subsequently, the crude products were purified with silica gel column chromatography to afford pure compound.

Condition B:

In a glovebox, an oven-dried vial with a stirring bar was charged with photoredox catalyst Ir(ppy)$_3$ (1.3 mg, 0.002 mmol), Co-**1** (3.6 mg, 0.006 mmol), HX-**1** (10.8 mg, 0.04 mmol) and DCM (1.0 mL). Then, phenol derivative **2** (0.2 mmol) and 1,3-dienes derivatives **1** (0.4 mmol) were added to the reaction mixture successively. The vial was then sealed and removed from the glove box for stirring and irradiation with a 40W blue LED (448 nm) equipped with a cooling fan to maintain the temperature at approximately 40 ^{o}C. The reaction is detected by TLC until the substrate is completely converted to chroman **4a**, the reaction mixture was then transferred to a separatory funnel and quenched with saturated $NaHCO_3$ solution followed by extraction with DCM (3×5 mL). The combined organic layer was washed with brine solution and dried over Na_2SO_4. The crude product was obtained after removal of solvent. Crude products were purified with silica gel column chromatography to afford pure compound.

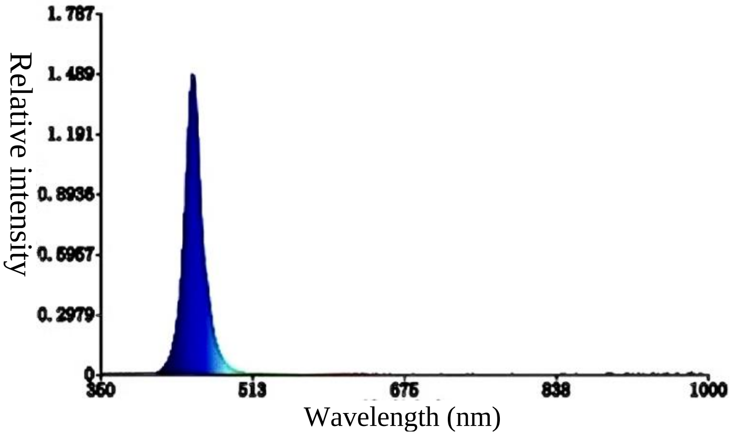

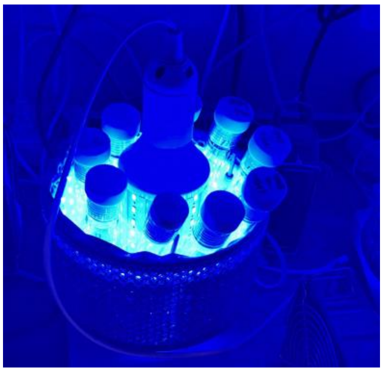

Fig S6 Emission spectrum of the utilised 40 W LED (448 nm) Light set up.

Scale up experiment.

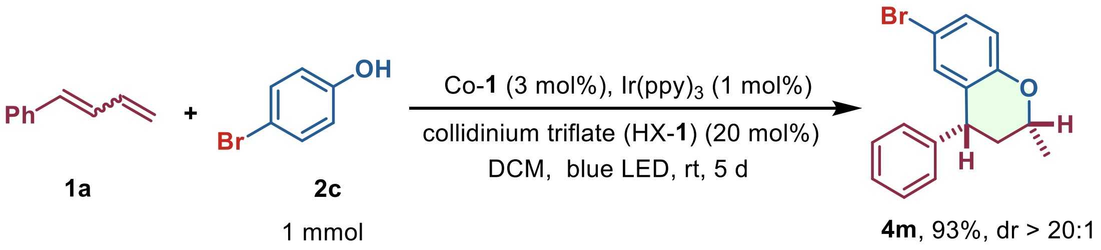

In a glovebox, an oven-dried vial with a stirring bar was charged with photoredox catalyst Ir(ppy)$_3$ (6.5 mg, 0.01 mmol), Co-**1** (18.0 mg, 0.03 mmol), HX-**1** (54 mg, 0.2 mmol) and DCM (5.0 mL). Then, 4-bromophenol (1.0 mmol) and 1-Phenyl-1,3-butadiene (2.0 mmol) were added to the reaction mixture. The vial was then sealed and removed from the glove box for stirring and irradiation with a 40W blue LED equipped with a cooling fan to maintain the temperature at approximately 40 ^{o}C. After 5 days, the reaction mixture was then transferred to a separatory funnel and quenched with saturated $NaHCO_3$ solution. After extractions with DCM, combined organic layer was washed with brine solution and dried over Na_2SO_4. The crude product was obtained after removal of solvent. Crude products were purified with silica gel column chromatography to afford pure compound **4m** as a yellow oil. The product yield of the 1 mmol scale experiment was 93% and the dr > 15:1.

V. Analytical Data of Products

(*E*)-(3-phenoxybut-1-en-1-yl)benzene (3a)

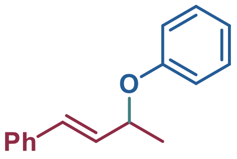

Condition A. Yellow oil, 85% yield (38.1 mg), >20:1 rr. **^{1}H NMR (600 MHz, $CDCl_3$)** δ 7.36 (d, *J* = 7.8 Hz, 2H), 7.29 (t, *J* = 7.5 Hz, 2H), 7.27 – 7.20 (m, 3H), 6.95 (d, *J* = 9.0 Hz, 2H), 6.92 (t, *J* = 7.2 Hz, 1H), 6.60 (d, *J* = 16.2 Hz, 1H), 6.32 – 6.23 (m, 1H), 5.04 – 4.90 (m, 1H), 1.52 (d, *J* = 6.0 Hz, 3H). **^{13}C NMR (151 MHz, $CDCl_3$)** δ 158.0, 136.5, 130.7, 130.6, 129.4, 128.5, 127.7, 126.5, 120.8, 116.1, 74.5, 21.7. **HRMS** (ESI-TOF) (m/z): calcd for $C_{16}H_{16}NaO$ ($[M+Na]^+$), 247.1093, found, 247.1094.

(*E*)-1-methyl-4-((4-phenylbut-3-en-2-yl)oxy)benzene (3b)

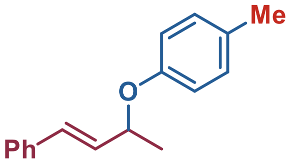

Condition A. Yellow oil, 91% yield (43.3 mg), >20:1 rr. **^{1}H NMR (500 MHz, $CDCl_3$)** δ 7.35 (d, *J* = 7.5 Hz, 2H), 7.28 (t, *J* = 7.5 Hz, 2H), 7.21 (d, *J* = 7.0 Hz, 1H), 7.04 (d, *J* = 8.5 Hz, 2H), 6.85 (d, *J* = 8.5 Hz, 2H), 6.58 (d, *J* = 16.0 Hz, 1H), 6.26 (dd, *J* = 16.0, 6.5 Hz, 1H), 4.94 – 4.86 (m, 1H), 2.26 (s, 3H), 1.50 (d, *J* = 6.0 Hz, 3H). **^{13}C NMR (151 MHz, $CDCl_3$)** δ 155.8, 136.5, 130.9, 130.5, 130.0, 129.8, 128.5, 127.6, 126.4, 116.1, 74.7, 21.7, 20.5. **HRMS** (ESI-TOF) (m/z): calcd for $C_{17}H_{18}NaO$ ($[M+Na]^+$), 261.1250, found, 261.1252.

(*E*)-1-isopropyl-4-((4-phenylbut-3-en-2-yl)oxy)benzene (3c)

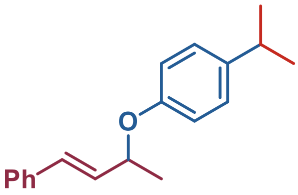

Condition A. Yellow oil, 92% yield (49.0 mg), >20:1 rr. **^{1}H NMR (600 MHz, $CDCl_3$)** δ 7.36 (d, *J* = 7.2 Hz, 2H), 7.30 (t, *J* = 7.2 Hz, 2H), 7.24 – 7.20 (m, 1H), 7.11 (d, *J* = 9.0 Hz, 2H), 6.88 (d, *J* = 9.0 Hz, 2H), 6.60 (d, *J* = 16.2 Hz, 1H), 6.29 (dd, *J* = 16.2, 6.6 Hz, 1H), 4.96 – 4.89 (m, 1H), 2.88 – 2.80 (m, 1H), 1.50 (d, *J* = 6.6 Hz, 3H), 1.21 (d, *J* = 7.2 Hz, 6H). **^{13}C NMR (151 MHz, $CDCl_3$)** δ 156.1, 141.1, 136.6, 131.0, 130.4, 128.5, 127.6, 127.2, 126.4, 115.8, 74.6, 33.2, 24.2, 24.1, 21.8. **HRMS** (ESI-TOF) (m/z): calcd for $C_{19}H_{22}NaO$ ($[M+Na]^+$), 289.1563, found, 289.1565.

(*E*)-1-isopropyl-2-((4-phenylbut-3-en-2-yl)oxy)benzene (3d)

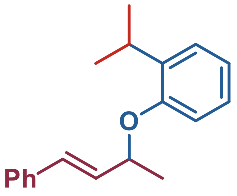

Condition A. Yellow oil, 73% yield (38.9 mg), >20:1 rr. **^{1}H NMR (600 MHz, $CDCl_3$)** δ 7.36 (d, *J* = 7.2 Hz, 2H), 7.30 (t, *J* = 7.8 Hz, 2H), 7.24 – 7.20 (m, 2H), 7.11 – 7.07 (m, 1H), 6.93 – 6.88 (m, 2H), 6.59 (d, *J* = 16.2 Hz, 1H), 6.30 (dd, *J* = 16.2, 6.0 Hz, 1H), 4.99 – 4.92 (m, 1H), 3.45 – 3.36 (m, 1H), 1.53 (d, *J* = 6.0 Hz, 3H), 1.24 (d, *J* = 7.2 Hz, 6H). **^{13}C NMR (151 MHz, $CDCl_3$)** δ 155.1, 137.8, 136.6, 131.1, 130.3, 128.5, 127.6, 126.5, 126.3, 126.1, 120.7, 113.6, 74.7, 26.9, 22.8, 21.8. **HRMS** (ESI-TOF) (m/z): calcd for $C_{19}H_{22}NaO$ ($[M+Na]^+$), 289.1563, found, 289.1556.

(*E*)-1-(tert-butyl)-4-((4-phenylbut-3-en-2-yl)oxy)benzene (3e)

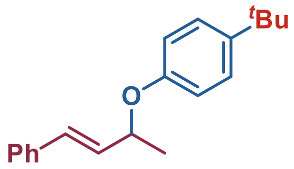

Condition A. Yellow oil, 90% yield (50.4 mg), >20:1 rr. **^{1}H NMR (500 MHz, $CDCl_3$)** δ 7.37 (d, *J* = 7.5 Hz, 2H), 7.33 – 7.25 (m, 4H), 7.23 (d, *J* = 7.0 Hz, 1H), 6.88 (d, *J* = 9.0 Hz, 2H), 6.61 (d, *J* = 16.5 Hz, 1H), 6.29 (dd, *J* = 16.0, 6.5 Hz, 1H), 4.98 – 4.89 (m, *J* = 6.3 Hz, 1H), 1.51 (d, *J* = 6.5 Hz, 3H), 1.28 (s, 9H). **^{13}C NMR (151 MHz, $CDCl_3$)** δ 155.8, 143.4, 136.6, 131.0, 130.5, 128.6, 127.7, 126.5, 126.2, 115.4, 74.5, 34.1, 31.5, 21.8. **HRMS** (ESI-TOF) (m/z): calcd for $C_{20}H_{24}NaO$ ([M+Na]$^+$), 303.1719, found, 303.1723.

(*E*)-2-(4-((4-phenylbut-3-en-2-yl)oxy)phenyl)acetonitrile (3f)

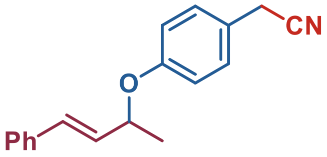

Condition A. Yellow oil, 84% yield (41.9 mg), >20:1 rr. **^{1}H NMR (600 MHz, $CDCl_3$)** δ 7.36 (d, *J* = 7.2 Hz, 2H), 7.30 (t, *J* = 7.8 Hz, 2H),7.24 (d, *J* = 7.2 Hz, 1H), 7.20 (d, *J* = 8.4 Hz, 2H), 6.94 (d, *J* = 8.4 Hz, 2H), 6.59 (d, *J* = 16.2 Hz, 1H), 6.25 (dd, *J* = 15.6, 6.0 Hz, 1H), 4.98 – 4.93 (m, 1H), 3.66 (s, 2H), 1.53 (d, J = 6.6 Hz, 3H). **^{13}C NMR (151 MHz, $CDCl_3$)** δ 157.8, 136.3, 131.0, 130.2, 129.0, 128.6, 127.8, 126.5, 121.9, 116.7, 74.8, 22.8, 21.7. **HRMS** (ESI-TOF) (m/z): calcd for $C_{18}H_{17}NNaO$ ([M+Na]$^+$), 286.1202, found, 286.1200.

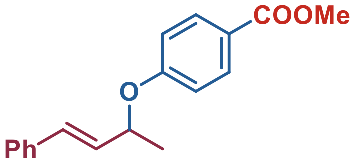

methyl (*E*)-4-((4-phenylbut-3-en-2-yl)oxy)benzoate (3g)

Condition A. Yellow oil, 80% yield (45.1 mg), >20:1 rr. **^{1}H NMR (500 MHz, $CDCl_3$)** δ 7.96 (d, *J* = 9.0 Hz, 2H), 7.35 (d, *J* = 7.0 Hz, 2H), 7.30 (t, *J* = 7.5 Hz, 2H), 7.24 (d, *J* = 6.0 Hz, 1H), 6.95 (d, *J* = 9.0 Hz, 2H), 6.61 (d, *J* = 16.0 Hz, 1H), 6.25 (dd, *J* = 16.0, 6.0 Hz, 1H), 5.09 – 5.00 (m, 1H), 3.86 (s, 3H), 1.54 (d, *J* = 6.5 Hz, 3H). **^{13}C NMR (151 MHz, $CDCl_3$)** δ 166.8, 161.8, 136.2, 131.5, 131.1, 129.7, 128.6, 127.9, 126.4, 122.4, 115.3, 74.6, 51.8, 21.6. **HRMS** (ESI-TOF) (m/z): calcd for $C_{18}H_{18}NaO_3$ ([M+Na]$^+$), 305.1148, found, 305.1154.

(*E*)-1-fluoro-4-((4-phenylbut-3-en-2-yl)oxy)benzene (3h)

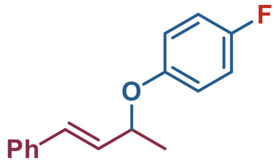

Condition A. Yellow oil, 90% yield (43.6 mg), >20:1 rr. **^{1}H NMR (500 MHz, $CDCl_3$)** δ 7.36 (d, *J* = 7.5 Hz, 2H), 7.30 (t, *J* = 7.5 Hz, 2H), 7.24 (d, *J* = 7.0 Hz, 1H), 6.94 (t, *J* = 8.5 Hz, 2H), 6.90 – 6.85 (m, 2H), 6.57 (d, *J* = 16.0 Hz, 1H), 6.24 (dd, *J* = 16.0, 6.5 Hz, 1H), 4.90 – 4.82 (m, 1H), 1.51 (d, *J* = 6.5 Hz, 3H). **^{13}C NMR (151 MHz, $CDCl_3$)** δ 157.3 (d, *J* = 238.2 Hz), 154.1, 136.4, 130.9, 130.4, 128.6, 127.8, 126.5, 117.4 (d, *J* = 8.1 Hz), 115.7 (d, *J* = 22.2 Hz), 75.6, 21.7. **^{19}F NMR (565 MHz, $CDCl_3$)** *δ* = -123.648 (s, 1F). **HRMS** (ESI-TOF) (m/z): calcd for $C_{16}H_{15}FNaO$ ([M+Na]$^+$), 265.0999, found, 265.1003.

(*E*)-1-chloro-4-((4-phenylbut-3-en-2-yl)oxy)benzene (3i)

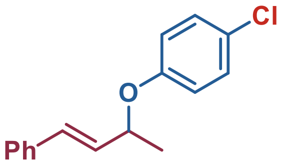

Condition A. Yellow oil, 96% yield (49.6 mg), >20:1 rr. **^{1}H NMR (600 MHz, $CDCl_3$)** δ 7.35 (d, *J* = 7.2 Hz, 2H), 7.30 (t, *J* = 7.8 Hz, 2H), 7.24 (d, *J* = 7.2 Hz, 1H), 7.20 (d, *J* = 9.0 Hz, 2H), 6.87 (d, *J* = 9.0 Hz, 2H), 6.58 (d, *J* = 16.2 Hz, 1H), 6.23 (dd, *J* = 15.6, 6.0 Hz, 1H), 4.93 – 4.87 (m, 1H), 1.51 (d, *J* = 6.0 Hz, 3H). **^{13}C NMR (151 MHz, $CDCl_3$)** δ

156.6, 136.3, 131.0, 130.1, 129.2, 128.6, 127.8, 126.5, 125.6, 117.4, 75.0, 21.7. **HRMS** (ESI-TOF) (m/z): calcd for $C_{16}H_{15}ClNaO$ ($[M+Na]^+$), 281.0704, found, 281.0698.

(*E*)-1-chloro-2-((4-phenylbut-3-en-2-yl)oxy)benzene (3j)

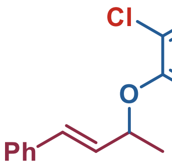

Condition A. Yellow oil, 80% yield (41.3 mg), >20:1 rr. **^{1}H NMR (600 MHz, $CDCl_3$)** δ 7.36 (t, *J* = 6.6 Hz, 3H), 7.30 (t, *J* = 7.2 Hz, 2H), 7.24 (d, *J* = 10.8 Hz, 1H), 7.14 (t, *J* = 7.8 Hz, 1H), 7.00 (d, *J* = 8.4 Hz, 1H), 6.88 (t, *J* = 7.8 Hz, 1H), 6.59 (d, *J* = 15.6 Hz, 1H), 6.29 (dd, *J* = 16.2, 6.6 Hz, 1H), 4.99 – 4.93 (m, 1H), 1.58 (d, *J* = 6.6 Hz, 3H). **^{13}C NMR (151 MHz, $CDCl_3$)** δ 153.7, 136.4, 131.1, 130.3, 130.1, 128.6, 127.8, 127.5, 126.5, 124.2, 121.8, 116.8, 76.7, 21.7. **HRMS** (ESI-TOF) (m/z): calcd for $C_{16}H_{15}ClNaO$ ($[M+Na]^+$), 281.0704, found, 281.0701.

(*E*)-1-bromo-4-((4-phenylbut-3-en-2-yl)oxy)benzene (3k)

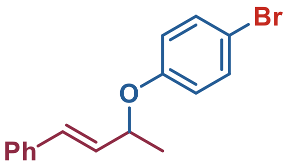

Condition A. Yellow oil, 93% yield (56.2 mg), >20:1 rr. **^{1}H NMR (500 MHz, $CDCl_3$)** δ 7.37 – 7.27 (m, 6H), 7.23 (t, *J* = 7.0 Hz, 1H), 6.82 (d, *J* = 9.0 Hz, 2H), 6.57 (d, *J* = 16.0 Hz, 1H), 6.22 (dd, *J* = 16.5, 6.5 Hz, 1H), 4.93 – 4.86 (m, 1H), 1.51 (d, *J* = 6.0 Hz, 3H). **^{13}C NMR (151 MHz, $CDCl_3$)** δ 157.1, 136.3, 132.2, 131.1, 130.1, 128.6, 127.8, 126.5, 117.9, 112.9, 74.9, 21.7. **HRMS** (ESI-TOF) (m/z): calcd for $C_{16}H_{15}BrNaO$ ($[M+Na]^+$), 325.0198, found, 325.0200.

(*E*)-1-bromo-2-((4-phenylbut-3-en-2-yl)oxy)benzene (3l)

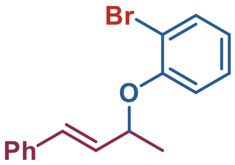

Condition A. Yellow oil, 70% yield (42.3 mg), >20:1 rr. **^{1}H NMR (500 MHz, $CDCl_3$)** δ 7.53 (dd, *J* = 7.5, 1.5 Hz, 1H), 7.37 (d, *J* = 7.5 Hz, 2H), 7.30 (t, *J* = 7.5, 2H), 7.24 (d, *J* = 6.0 Hz, 1H), 7.21 – 7.17 (m, 1H), 6.96 (d, *J* = 9.0 Hz, 1H), 6.81 (t, *J* = 8.5 Hz, 1H), 6.60 (d, *J* = 16.0 Hz, 1H), 6.29 (dd, *J* = 16.0, 6.5 Hz, 1H), 5.00 – 4.92 (m, *J* = 6.3 Hz, 1H), 1.59 (d, *J* = 6.5 Hz, 3H). **^{13}C NMR (151 MHz, $CDCl_3$)** δ 154.6, 136.4, 133.4, 131.1, 130.1, 128.6, 128.2, 127.8, 126.5, 122.2, 116.5, 113.6, 76.7, 21.7. **HRMS** (ESI-TOF) (m/z): calcd for $C_{16}H_{15}BrNaO$ ($[M+Na]^+$), 325.0198, found, 325.0195.

(*E*)-1-((4-phenylbut-3-en-2-yl)oxy)-4-(trifluoromethyl)benzene (3m)

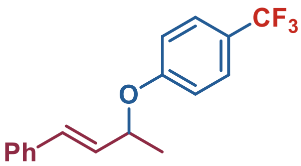

Condition A. Yellow oil, 65% yield (37.8 mg), >20:1 rr. **^{1}H NMR (500 MHz, $CDCl_3$)** δ 7.51 (d, *J* = 8.5 Hz, 2H), 7.36 (d, *J* = 7.0 Hz, 2H), 7.31 (t, *J* = 7.5 Hz, 2H), 7.26 – 7.23 (m, 1H), 7.00 (d, *J* = 8.5 Hz, 2H), 6.61 (d, *J* = 16.0 Hz, 1H), 6.24 (dd, *J* = 16.0, 6.5 Hz, 1H), 5.06 – 4.98 (m, 1H), 1.55 (d, *J* = 6.0 Hz, 3H).**^{13}C NMR (151 MHz, $CDCl_3$)** δ 160.5, 136.1, 131.2, 129.7, 128.6, 128.0, 126.8 (q, *J* = 3.8 Hz), 126.5, 124.4 (q, *J* = 271.0 Hz), 122.7 (q, *J* = 32.5 Hz), 115.8, 74.8, 21.7. **^{19}F NMR (565 MHz, $CDCl_3$)** *δ* = -61.490 (s, $1CF_3$). **HRMS** (ESI-TOF) (m/z): calcd for $C_{17}H_{15}F_3NaO$ ($[M+Na]^+$), 315.0967, found, 315.0969.

(*E*)-1-((4-phenylbut-3-en-2-yl)oxy)-4-(trifluoromethoxy)benzene (3n)

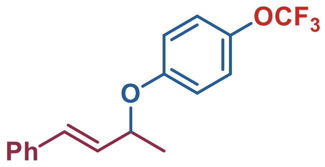

Condition A. Yellow oil, 70% yield (43.0 mg), >20:1 rr. **^{1}H NMR (500 MHz, CDCl$_3$)** δ 7.36 (d, *J* = 7.5 Hz, 2H), 7.30 (t, *J* = 7.5 Hz, 2H), 7.23 (t, *J* = 7.5 Hz, 1H), 7.10 (d, *J* = 9.0 Hz, 2H), 6.92 (d, *J* = 9.0 Hz, 2H), 6.59 (d, *J* = 16.0 Hz, 1H), 6.24 (dd, *J* = 16.0, 6.0 Hz, 1H), 4.95 – 4.88 (m, 1H), 1.52 (d, *J* = 6.0 Hz, 3H). **^{13}C NMR (151 MHz, CDCl$_3$)** δ 156.5, 142.7 (d, *J* = 2.3 Hz), 136.3, 131.0, 130.1, 128.6, 127.9, 126.5, 122.3, 120.5 (q, *J* = 255.9Hz), 116.8, 75.2, 21.7. **^{19}F NMR (565 MHz, CDCl$_3$)** *δ* = -58.327 (s, 1OCF$_3$). **HRMS** (ESI-TOF) (m/z): calcd for $C_{17}H_{15}F_3NaO_2$ ([M+Na]$^+$), 331.0916, found, 331.0919.

(*E*)-1-methoxy-4-((4-phenylbut-3-en-2-yl)oxy)benzene (3o)

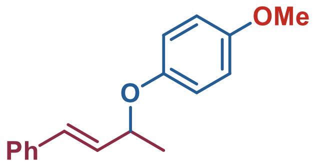

Condition A. Yellow oil, 94% yield (47.8 mg), >20:1 rr. **^{1}H NMR (500 MHz, CDCl$_3$)** δ 7.35 (d, *J* = 10.0 Hz, 2H), 7.29 (t, *J* = 5.0 Hz, 2H), 7.25 – 7.18 (m, 1H), 6.92 – 6.86 (m, 2H), 6.82 – 6.76 (m, 2H), 6.56 (dd, *J* = 5.0, 15.0 Hz, 1H), 6.26 (dd, *J* = 5.0, 15.0 Hz, 1H), 4.86 – 4.79 (m, 1H), 3.73 (s, 3H), 1.49 (d, *J* = 10 Hz, 3H). **^{13}C NMR (151 MHz, CDCl$_3$)** δ 154.0, 152.0, 136.5, 131.0, 130.6, 128.5, 127.6, 126.4, 117.6, 114.5, 75.7, 55.6, 21.7. **HRMS** (ESI-TOF) (m/z): calcd for $C_{17}H_{18}NaO_2$ ([M+Na]$^+$), 277.1199, found, 277.1203.

(*E*)-1-phenoxy-4-((4-phenylbut-3-en-2-yl)oxy)benzene (3p)

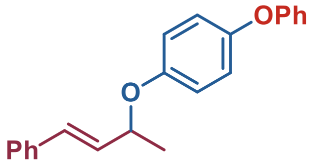

Condition A. Yellow oil, 93% yield (58.8 mg), >20:1 rr. **^{1}H NMR (500 MHz, CDCl$_3$)** δ 7.36 (d, *J* = 7.5 Hz, 2H), 7.32 – 7.24 (m, 4H), 7.23 (d, *J* = 7.5 Hz, 1H), 7.02 (t, *J* = 7.5 Hz, 1H), 6.97 – 6.89 (m, 6H), 6.59 (d, *J* = 16.0 Hz, 1H), 6.27 (dd, *J* = 16.0, 6.5 Hz, 1H), 4.93 – 4.85 (m, 1H), 1.51 (d, *J* = 6.5 Hz, 3H). **^{13}C NMR (151 MHz, CDCl$_3$)** δ 158.3, 154.2, 150.3, 136.4, 130.8, 130.6, 129.6, 128.5, 127.7, 126.4, 122.4, 120.6, 117.7, 117.3, 75.3, 21.7. **HRMS** (ESI-TOF) (m/z): calcd for $C_{22}H_{20}NaO_2$ ([M+Na]$^+$), 339.1356, found, 339.1359.

(*E*)-4-((4-phenylbut-3-en-2-yl)oxy)-1,1'-biphenyl (3q)

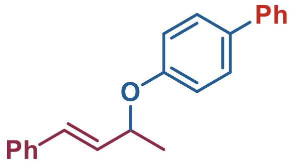

Condition A. Yellow oil, 92% yield (55.2 mg), >20:1 rr. **^{1}H NMR (600 MHz, CDCl$_3$)** δ 7.43 (d, *J* = 7.8 Hz, 2H), 7.39 (d, *J* = 8.4 Hz, 2H), 7.32 – 7.26(m, 4H), 7.22 – 716 m, 3H), 7.13 (d, *J* = 7.2 Hz, 1H), 6.92 (d, *J* = 9.0 Hz, 2H), 6.53 (d, *J* = 16.2 Hz, 1H), 6.20 (dd, *J* = 16.2, 6.0 Hz, 1H), 4.93 – 4.86 (m, 1H), 1.44 (d, *J* = 6.0 Hz, 3H). **^{13}C NMR (151 MHz, CDCl$_3$)** δ 157.7, 140.9, 136.6, 133.9, 130.8, 130.7, 128.8, 128.7, 128.2, 127.9, 126.8, 126.7, 126.6, 116.4, 74.7, 21.8. **HRMS** (ESI-TOF) (m/z): calcd for $C_{22}H_{20}NaO$ ([M+Na]$^+$), 323.1406, found, 323.1408.

(*E*)-4,4,5,5-tetramethyl-2-(4-((4-phenylbut-3-en-2-yl)oxy)phenyl)-1,3,2-dioxaborolane (3r)

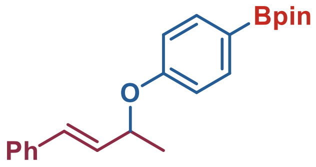

Condition A. Yellow oil, 92% yield (64.4 mg), >20:1 rr. **^{1}H NMR (600 MHz, $CDCl_3$)** δ 7.72 (d, *J* = 8.4 Hz, 2H), 7.35 (d, *J* = 7.2 Hz, 2H), 7.29 (t, *J* = 7.2 Hz, 2H), 7.22 (t, *J* = 7.2 Hz, 1H), 6.94 (d, *J* = 8.4 Hz, 2H), 6.60 (d, *J* = 16.2 Hz, 1H), 6.26 (dd, *J* = 16.2, 6.6 Hz, 1H), 5.07 – 5.00 (m, 1H), 1.52 (d, *J* = 6.0 Hz, 3H), 1.32 (s, 12H). **^{13}C NMR (151 MHz, $CDCl_3$)** δ 160.6, 136.4, 130.7, 130.3, 128.5, 127.7, 126.4, 115.3, 83.5, 74.1, 24.9, 24.8, 21.6. ^{11}B NMR (193 MHz, $CDCl_3$) δ 30.59. **HRMS** (ESI-TOF) (m/z): calcd for $C_{22}H_{27}BNaO_3$ ([M+Na]$^+$), 373.1945, found, 373.1949.

(*E*)-1-chloro-2-((4-(p-tolyl)but-3-en-2-yl)oxy)benzene (3s)

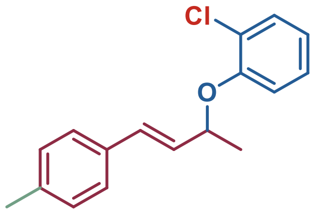

Condition A. Yellow oil, 62% yield (33.7 mg), >20:1 rr. **^{1}H NMR (600 MHz, $CDCl_3$)** δ 7.35 (d, *J* = 7.8 Hz, 1H), 7.26 (d, *J* = 8.4 Hz, 2H), 7.14 – 7.10 (m, 3H), 6.99 (d, *J* = 8.4 Hz, 1H), 6.87 (t, *J* = 7.8 Hz, 1H), 6.55 (d, *J* = 15.6 Hz, 1H), 6.24 (dd, *J* = 16.2, 6.6 Hz, 1H), 4.97 – 4.91 (m, 1H), 2.32 (s, 3H), 1.57 (d, *J* = 6.0 Hz, 3H). **^{13}C NMR (151 MHz, $CDCl_3$)** δ 153.7, 137.7, 133.6, 131.1, 130.3, 129.3, 129.1, 127.4, 126.4, 124.2, 121.8, 116.9, 76.9, 21.7, 21.2. **HRMS** (ESI-TOF) (m/z): calcd for $C_{17}H_{17}ClNaO$ ([M+Na]$^+$), 295.0860, found, 295.0864.

(*E*)-1-chloro-2-((4-(m-tolyl)but-3-en-2-yl)oxy)benzene (3t)

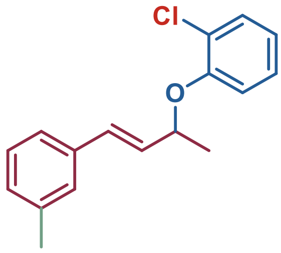

Condition A. Yellow oil, 80% yield (43.5 mg), >20:1 rr. **^{1}H NMR (600 MHz, $CDCl_3$)** δ 7.35 (d, *J* = 8.4 Hz, 1H), 7.21 – 7.16 (m, 3H), 7.13 (d, *J* = 7.8 Hz, 1H), 7.05 (d, *J* = 7.2 Hz, 1H), 6.99 (d, *J* = 8.4 Hz, 1H), 6.87 (t, *J* = 7.2 Hz, 1H), 6.56 (d, *J* = 15.6 Hz, 1H), 6.27 (dd, *J* = 16.2, 6.6 Hz, 1H), 4.98 – 4.92 (m, 1H), 2.33 (s, 3H), 1.58 (d, *J* = 6.6 Hz, 3H). **^{13}C NMR (151 MHz, $CDCl_3$)** δ 153.7, 138.1, 136.3, 131.2, 130.3, 129.9, 128.6, 128.5, 127.4, 127.2, 124.2, 123.7, 121.8, 116.7, 76.7, 21.7, 21.3. **HRMS** (ESI-TOF) (m/z): calcd for $C_{17}H_{17}ClNaO$ ([M+Na]$^+$), 295.0860, found, 295.0861.

(*E*)-1-((4-(3-(tert-butyl)phenyl)but-3-en-2-yl)oxy)-2-chlorobenzene (3u)

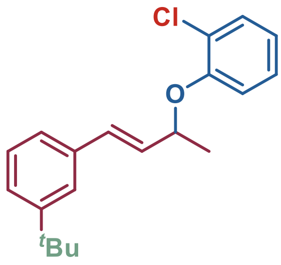

Condition A. Yellow oil, 70% yield (44.0 mg), >20:1 rr. **^{1}H NMR (600 MHz, $CDCl_3$)** δ 7.36 (d, *J* = 8.4 Hz, 2H), 7.28 (d, *J* = 7.8 Hz, 1H), 7.23 (d, *J* = 4.2 Hz, 1H), 7.21 (d, *J* = 7.8 Hz, 1H), 7.15 (t, *J* = 7.2 Hz, 1H), 7.01 (d, *J* = 8.4 Hz, 1H), 6.88 (t, *J* = 7.5 Hz, 1H), 6.60 (d, *J* = 15.6 Hz, 1H), 6.29 (dd, *J* = 16.2, 6.6 Hz, 1H), 4.99 – 4.93 (m, 1H), 1.59 (d, *J* = 6.0 Hz, 3H), 1.32 (s, 9H). **^{13}C NMR (151 MHz, $CDCl_3$)** δ 153.8, 151.4, 136.0, 133.0, 131.7, 130.3, 129.8, 128.3, 127.5, 125.0, 123.7, 123.5, 121.8, 116.8, 76.9, 34.6, 31.3, 21.8. **HRMS** (ESI-TOF) (m/z): calcd for $C_{20}H_{23}ClNaO$ ([M+Na]$^+$), 337.1330, found, 337.1333.

(*E*)-1-chloro-2-((4-(4-fluorophenyl)but-3-en-2-yl)oxy)benzene (3v)

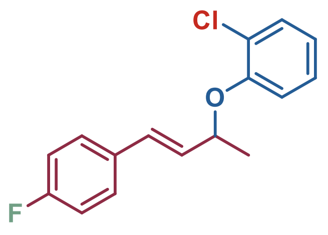

Condition A. Yellow oil, 74% yield (40.9 mg), >20:1 rr. **^{1}H NMR (600 MHz, $CDCl_3$)** δ 7.36 (dd, *J* = 8.4, 1.8 Hz, 1H), 7.34 – 7.30 (m, 2H), 7.17 – 7.13 (m, 1H), 6.99 (t, *J* = 9.0 Hz, 3H), 6.90 – 6.86 (m, 1H), 6.55 (d, *J* = 16.2 Hz, 1H), 6.20 (dd, *J* = 16.2, 6.6 Hz, 1H), 4.97 – 4.91 (m, 1H), 1.57 (d, *J* = 6.6 Hz, 3H). **^{13}C NMR (151 MHz, $CDCl_3$)** δ 162.4 (d, *J* = 246.7 Hz), 153.6, 132.5 (d, *J* = 3.5 Hz), 130.4, 129.9, 129.8 (d, *J* = 1.9 Hz), 128.1 (d, *J* = 7.4 Hz), 127.5, 124.2, 121.9, 116.7, 115.5 (d, *J* = 21.6 Hz), 76.6, 21.6. **^{19}F NMR (565 MHz, CDCl3)** δ = -114.04 ~ -114.11 (m, 1F). HRMS (ESI-TOF) (m/z): calcd for $C_{16}H_{14}ClFNaO$ ([M+Na]+), 299.0609, found, 299.0613.

(*E*)-1-((4-(4-bromophenyl)but-3-en-2-yl)oxy)-2-chlorobenzene (3w)

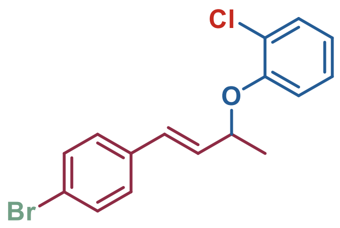

Condition A. Yellow oil, 73% yield (49.1 mg), >20:1 rr. **^{1}H NMR (600 MHz, $CDCl_3$)** δ 7.43 (d, *J* = 8.4 Hz, 2H), 7.36 (dd, *J* = 7.8, 1.8 Hz, 1H), 7.23 (d, *J* = 8.4 Hz, 2H), 7.19 – 7.13 (m, 1H), 6.97 (d, *J* = 8.4 Hz, 1H), 6.91 – 6.87 (m, 1H), 6.54 (d, *J* = 16.2 Hz, 1H), 6.29 (dd, *J* = 16.2, 6.6 Hz, 1H), 4.97 – 4.92 (m, 1H), 1.57 (d, *J* = 6.0 Hz, 3H). **^{13}C NMR (151 MHz, $CDCl_3$)** δ 153.6, 135.3, 131.7, 130.9, 130.4, 129.9, 128.1, 127.5, 124.3, 122.0, 121.6, 116.7, 76.5, 21.5. **HRMS** (ESI-TOF) (m/z): calcd for $C_{16}H_{14}BrClNaO$ ($[M+Na]^+$), 358.9809, found, 358.9807.

(*E*)-1-chloro-2-((4-(4-(trifluoromethyl)phenyl)but-3-en-2-yl)oxy)benzene (3x)

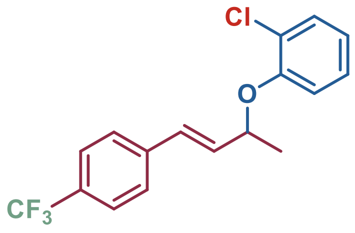

Condition A. Yellow oil, 71% yield (46.3 mg), >20:1 rr. **^{1}H NMR (600 MHz, $CDCl_3$)** δ 7.56 (d, *J* = 8.4 Hz, 2H), 7.46 (d, *J* = 8.4 Hz, 2H), 7.37 (dd, *J* = 9.0, 1.8 Hz, 1H), 7.19 – 7.12 (m, 1H), 6.98 (dd, *J* = 8.4, 1.2 Hz, 1H), 6.92 – 6.88 (m, 1H), 6.64 (d, *J* = 16.2 Hz, 1H), 6.40 (dd, *J* = 16.2, 6.0 Hz, 1H), 5.02 – 4.95 (m, 1H), 1.59 (d, *J* = 6.6 Hz, 3H).**^{13}C NMR (151 MHz, $CDCl_3$)** δ 153.5, 139.9, 132.8, 130.4, 129.7 (q, *J* = 33.1 Hz), 129.6, 127.5, 126.7, 125.5 (q, *J* = 3.5 Hz), 124.1 (q, *J* = 272.4 Hz), 124.3, 122.1, 116.7, 76.3, 21.5. **^{19}F NMR (565 MHz, CDCl3)** δ = -62.543 (s, $1CF_3$). **HRMS** (ESI-TOF) (m/z): calcd for $C_{17}H_{14}ClF_3NaO$ ($[M+Na]^+$), 349.0577, found, 349.0580.

(*E*)-3-(3-(2-chlorophenoxy)but-1-en-1-yl)-1,1'-biphenyl (3y)

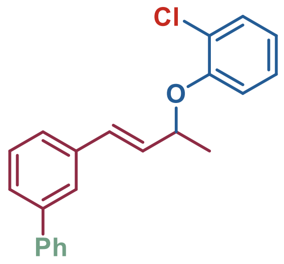

Condition A. Yellow oil, 74% yield (49.4 mg), >20:1 rr. **^{1}H NMR (600 MHz, $CDCl_3$)** δ 7.58 (d, *J* = 7.2 Hz, 3H), 7.49 – 7.41 (m, 3H), 7.41 – 7.32 (m, 4H), 7.18 – 7.13 (m, 1H), 7.01 (d, *J* = 7.8 Hz, 1H), 7.92 – 6.85 (m, 1H), 6.66 (d, *J* = 16.2 Hz, 1H), 6.37 (dd, *J* = 16.2, 6.6 Hz, 1H), 5.02 – 4.95 (m, 1H), 1.60 (d, *J* = 6.0 Hz, 3H). **^{13}C NMR (151 MHz, $CDCl_3$)** δ 153.7, 141.6, 141.0, 136.8, 131.0, 130.5, 130.4, 129.0, 128.8, 127.5, 127.4, 127.2, 126.7, 125.5, 125.4, 124.2, 121.9, 116.7, 76.6, 21.7. **HRMS** (ESI-TOF) (m/z): calcd for $C_{22}H_{19}ClNaO$ ($[M+Na]^+$), 357.1017, found, 357.1019.

(*E*)-1-chloro-4-(3-phenoxybut-1-en-1-yl)benzene (3z)

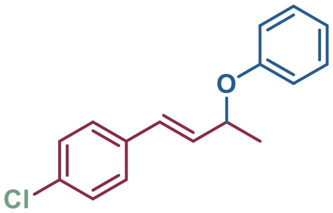

Condition A. Yellow oil, 78% yield (40.3 mg), >20:1 rr. **^{1}H NMR (500 MHz, $CDCl_3$)** δ 7.30 – 7.22 (m, 6H), 6.94 (d, *J* = 8.5 Hz, 3H), 6.55 (d, *J* = 16.0 Hz, 1H), 6.25 (dd, *J* = 16.0, 6.0 Hz, 1H), 5.00 – 4.91 (m, 1H), 1.51 (d, *J* = 6.5 Hz, 3H).**^{13}C NMR (151 MHz, $CDCl_3$)** δ 157.9, 135.0, 133.3, 131.3, 129.4, 129.3, 128.7, 127.7, 120.9, 116.0, 74.2, 21.6. **HRMS** (ESI-TOF) (m/z): calcd for $C_{16}H_{15}ClNaO$ ($[M+Na]^+$), 281.0704, found, 281.0703.

(*E*)-1-bromo-4-(3-(2-chlorophenoxy)but-1-en-1-yl)-2-methylbenzene （3aa)

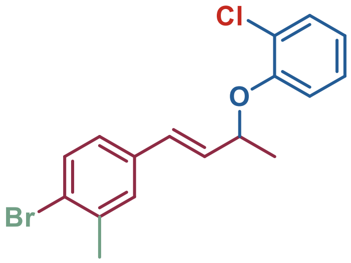

Condition A. Yellow oil, 64% yield (44.8 mg), >20:1 rr. **^{1}H NMR (600 MHz, $CDCl_3$)** δ 7.45 (d, *J* = 7.8 Hz, 1H), 7.36 (d, *J* = 7.8 Hz, 1H), 7.22 (s, 1H), 7.15 (t, *J* = 7.8 Hz, 1H), 7.05 (d, *J* = 7.8 Hz, 1H), 6.97 (d, *J* = 8.4 Hz, 1H), 6.89 (t, *J* = 7.8 Hz, 1H), 6.51 (d, *J* = 15.6 Hz, 1H), 6.28 (dd, *J* = 16.2, 6.6 Hz, 1H), 4.98 – 4.90 (m, 1H), 2.37 (s, 3H), 1.57 (d, *J* = 6.4 Hz, 3H). **^{13}C NMR (151 MHz, $CDCl_3$)** δ 153.6, 138.0, 135.6, 132.5, 130.6, 130.4, 130.1, 128.9, 127.5, 125.3, 124.3, 124.1, 121.9, 116.7, 76.5, 22.9, 21.6. **HRMS** (ESI-TOF) (m/z): calcd for $C_{17}H_{16}BrClNaO$ ($[M+Na]^+$), 372.9965, found, 372.9968.

(*E*)-1-chloro-2-((1-phenylpent-1-en-3-yl)oxy)benzene (3ab)

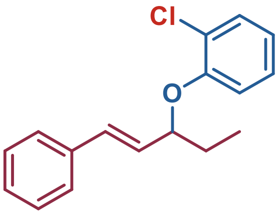

Condition A. Yellow oil, 99% yield (53.9 mg), >20:1 rr. **^{1}H NMR (600 MHz, CDCl3)** δ 7.38 – 7.34 (m, 3H), 7.30 (t, *J* = 7.2 Hz, 2H), 7.25 – 7.21 (m, 1H), 7.15 – 7.11 (m, 1H), 6.98 (d, *J* = 7.8 Hz, 1H), 6.88 – 6.84 (m, 1H), 6.57 (d, *J* = 10.2 Hz, 1H), 6.25 (dd, *J* = 16.2, 6.6 Hz, 1H), 4.73 – 4.68 (m, 1H), 2.02 – 1.94 (m, 1H), 1.90 – 1.82 (m, 1H), 1.08 (t, *J* = 7.8 Hz, 3H). **^{13}C NMR (151 MHz, CDCl3)** δ 154.0, 136.4, 131.9, 130.3, 128.9, 128.6, 127.8, 127.4, 126.5, 124.0, 121.6, 116.4, 82.0, 29.0, 9.7. **HRMS** (ESI-TOF) (m/z): calcd for $C_{17}H_{17}ClNaO$ ($[M+Na]^+$), 295.0860, found, 295.0857.

(*E*)-(2-methyl-3-phenoxybut-1-en-1-yl)benzene (3ac)

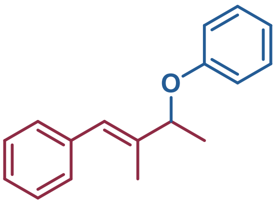

Condition A. Yellow oil, 63% yield (30.0 mg), >20:1 rr. **^{1}H NMR (500 MHz, $CDCl_3$)** δ 7.31 (t, *J* = 7.5 Hz, 2H), 7.28 – 7.22 (m, 4H), 7.20 (t, *J* = 7.5 Hz, 1H), 6.98 – 6.87 (m, 3H), 6.55 (s, 1H), 4.85 – 4.78 (m, 1H), 1.88 (s, 3H), 1.53 (d, *J* = 6.0 Hz, 3H). **^{13}C NMR (151 MHz, $CDCl_3$)** δ 158.3, 139.0, 137.6, 129.5, 129.1, 128.3, 126.7, 126.4, 120.9, 116.2, 79.4, 21.0, 13.3. **HRMS** (ESI-TOF) (m/z): calcd for $C_{17}H_{18}NaO$ ($[M+Na]^+$), 261.1250, found, 261.1054.

(3-(2-chlorophenoxy)but-1-ene-1,1-diyl)dibenzene (3ad)

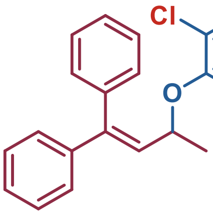

Condition A. Yellow oil, 99% yield (66.2 mg), >20:1 rr. **^{1}H NMR (500 MHz, $CDCl_3$)** δ 7.40 – 7.33 (m, 3H), 7.30 (dd, *J* = 8.0, 2.0 Hz, 1H), 7.25 – 7.21 (m, 3H), 7.20 – 7.16 (m, 2H), 7.12 – 7.05 (m, 2H), 7.03 – 7.98 (m, 1H), 6.85 – 6.80 (m, 1H), 6.60 – 6.56 (m, 1H), 6.14 (d, *J* = 9.0 Hz, 1H), 4.88 – 4.80 (m, 1H), 1.58 (d, *J* = 6.0 Hz, 3H).**^{13}C NMR (151 MHz, $CDCl_3$)** δ 153.4, 144.0, 141.2, 139.0, 130.1, 129.5, 129.4, 128.3, 128.2, 127.7, 127.6, 127.4, 127.2, 124.2, 121.7, 117.2, 73.7, 21.7. **HRMS** (ESI-TOF) (m/z): calcd for $C_{22}H_{19}ClNaO$ ($[M+Na]^+$), 357.1017, found, 357.1021.

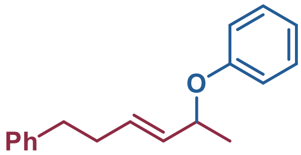

(*E*)-(5-phenoxyhex-3-en-1-yl)benzene (3ae)

Condition A. Yellow oil, 61% yield (30.8 mg), rr = 1.7:1. **^{1}H NMR (600 MHz, $CDCl_3$)** δ 7.28 – 7.21 (m, 4H), 7.17 (t, *J* = 7.2 Hz, 1H), 7.12 (d, *J* = 7.2 Hz, 2H), 6.94 – 6.85 (m, 3H), 5.75 – 5.66 (m, 1H), 5.56 – 5.50 (m, 1H), 4.78 – 4.70 (m, 1H), 2.66 (t, *J* = 7.8 Hz, 2H), 2.38 – 2.30 (m, 2H), 1.38 (d, *J* = 6.6 Hz, 3H). **^{13}C NMR (151 MHz, $CDCl_3$)** δ 158.4, 141.8, 130.8, 129.3 , 128.5, 128.4, 128.3, 125.8, 120.5, 116.1, 77.6, 37.4, 31.6, 17.8. **HRMS** (ESI-TOF) (m/z): calcd for $C_{18}H_{20}NaO$ ($[M+Na]^+$), 275.1406, found, 275.1409.

(*E*)-(3-phenoxyhex-4-en-1-yl)benzene (3ae')

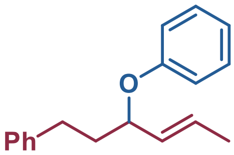

Condition A. Yellow oil, 35% yield (17.7 mg), rr = 1.7:1. **^{1}H NMR (600 MHz, $CDCl_3$)** δ 7.28 – 7.22 (m, 4H), 7.20 – 7.14 (m, 3H), 6.91 (t, *J* = 7.8 Hz, 1H), 6.87 (d, *J* = 7.8 Hz, 2H), 5.76 – 5.64 (m, 1H), 5.53 – 5.46 (m, 1H), 4.58 – 4.52 (m, 1H), 2.83 – 2.69 (m, 2H), 2.17 – 2.06 (m, 1H), 1.97 – 1.88 (m, 1H), 1.69 (d, *J* = 7.2 Hz, 3H). **^{13}C NMR (151 MHz, $CDCl_3$)** δ 158.0, 141.6, 131.7, 131.3, 129.3, 128.5, 128.6, 125.8, 120.5, 116.1, 74.3, 35.5, 34.0, 21.6. **HRMS** (ESI-TOF) (m/z): calcd for $C_{18}H_{20}NaO$ ($[M+Na]^+$), 275.1406, found, 275.1407.

(*E*)-1-chloro-2-((6-phenylhex-3-en-2-yl)oxy)benzene (3af)

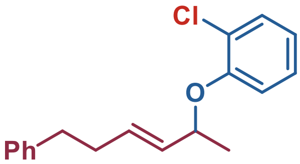

Condition A. Yellow oil, 54% yield (30.9 mg), rr = 1.2:1. **^{1}H NMR (600 MHz, $CDCl_3$)** δ 7.34 (dd, *J* = 7.8, 1.2 Hz, 1H), 7.25 (t, *J* = 7.8 Hz, 2H), 7.19 – 7.10 (m, 4H), 6.92 – 6.83 (m, 2H), 5.76 – 5.63 (m, 1H), 5.55 (dd, *J* = 15.6, 6.6 Hz, 1H), 4.76 – 4.70 (m, 1H), 2.68 – 2.63 (m, 2H), 2.38 – 2.30 (m, 2H), 1.44 (d, *J* = 6.6 Hz, 3H). **^{13}C NMR (151 MHz, $CDCl_3$)** δ 153.7, 141.5, 131.9, 131.2, 130.2, 128.4, 128.3, 127.3, 125.8, 124.2, 121.5, 116.8, 76.4, 35.4, 33.9, 21.6. **HRMS** (ESI-TOF) (m/z): calcd for $C_{18}H_{19}ClNaO$ ($[M+Na]^+$), 309.1017, found, 309.1021.

(*E*)-1-chloro-2-((1-phenylhex-4-en-3-yl)oxy)benzene (3af')

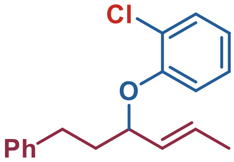

Condition A. Yellow oil, 44% yield (25.2 mg), rr = 1.2:1. **^{1}H NMR (500 MHz, $CDCl_3$)** δ 7.35 (d, *J* = 8.0 Hz, 1H), 7.25 (d, *J* = 7.5 Hz, 2H), 7.20 – 7.15 (m, 3H), 7.12 (t, *J* = 6.5 Hz, 1H), 6.88 – 6.83 (m, 2H), 5.73 – 5.64 (m, 1H), 5.56 – 5.49 (m, 1H), 4.58 – 4.52 (m, 1H), 2.89 – 2.74 (m, 2H), 2.26 – 2.14 (m, 1H), 2.04 – 1.91 (m, 1H), 1.69 (d, *J* = 6.5 Hz, 3H). **^{13}C NMR**

(151 MHz, $CDCl_3$) δ 153.9, 141.7, 130.4, 130.2, 128.7, 128.5, 128.4, 127.3, 125.8, 123.7, 121.3, 116.0, 79.4, 37.5, 31.4, 17.8. **HRMS** (ESI-TOF) (m/z): calcd for $C_{18}H_{19}ClNaO$ ($[M+Na]^+$), 309.1017, found, 309.1018.

(*E*)-1-chloro-2-((3-ethylhept-3-en-2-yl)oxy)benzene (3ag)

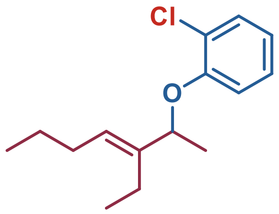

Condition A. Yellow oil, 65% yield (32.8 mg), rr = 1.9:1. **^{1}H NMR (600 MHz, $CDCl_3$)** δ 7.32 (dd, *J* = 7.8, 1.7 Hz, 1H), 7.15 – 7.09 (m, 1H), 6.91 (dd, *J* = 8.4, 1.8 Hz, 1H), 6.86 – 6.80 (m, 1H), 5.43 (t, *J* = 7.2 Hz, 1H), 4.77 – 4.71 (m, 1H), 2.17 – 2.11 (m, 2H), 2.05 – 1.97 (m, 2H), 1.49 (d, *J* = 6.6 Hz, 3H), 1.38 – 1.32 (m, 2H), 1.00 (t, *J* = 7.8 Hz, 3H), 0.85 (t, *J* = 7.2 Hz, 3H). **^{13}C NMR (151 MHz, $CDCl_3$)** δ 153.9, 140.5, 130.1, 128.0, 127.2, 123.7, 121.1, 116.0, 79.9, 29.4, 22.7, 21.0, 19.9, 14.2, 13.7. **HRMS** (ESI-TOF) (m/z): calcd for $C_{15}H_{21}ClNaO$ ($[M+Na]^+$), 275.1173, found, 275.1176.

(E)-1-chloro-2-((3-ethylhept-2-en-4-yl)oxy)benzene (3ag')

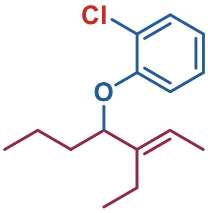

Condition A. Yellow oil, 34% yield (17.1 mg), rr = 1.9:1. **^{1}H NMR (600 MHz, $CDCl_3$)** δ 7.32 (d, *J* = 6.0 Hz, 1H), 7.11 (t, *J* = 7.8 Hz, 1H), 6.89 (d, *J* = 8.4 Hz, 1H), 6.81 (t, *J* = 7.8 Hz, 1H), 5.53 – 5.47 (m, 1H), 4.53 (t, *J* = 6.6 Hz, 1H), 2.16 – 2.09 (m, 2H), 1.90 – 1.83 (m, 1H), 1.71 – 1.65 (m, 1H), 1.63 (d, *J* = 6.6 Hz, 3H), 1.53 – 1.47 (m, 1H), 1.41 – 1.35 (m, 1H), 0.99 – 0.93 (m, 6H). **^{13}C NMR (151 MHz, $CDCl_3$)** δ 154.2, 140.2, 130.1, 127.2, 123.4, 122.7, 120.7, 115.3, 84.1, 36.8, 19.6, 19.1, 13.9, 13.6, 12.9. **HRMS** (ESI-TOF) (m/z): calcd for $C_{15}H_{21}ClNaO$ ($[M+Na]^+$), 275.1173, found, 275.1174.

1-chloro-2-(cyclohex-2-en-1-yloxy)benzene (3ah)

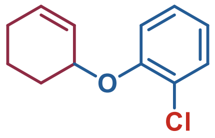

Condition A. Yellow oil, 98% yield (41.2 mg). **^{1}H NMR (600 MHz, $CDCl_3$)** δ 7.35 (dd,*J* = 7.8, 1.2 Hz, 1H), 7.19 – 7.16 (m, 1H), 7.05 – 6.98 (m, 1H), 6.99 (dd, *J* = 8.4, 1.2 Hz, 1H), 6.00 – 5.96 (m, 1H), 5.91 – 5.87 (m, 1H), 4.81 – 4.76 (m, 1H), 2.18 – 2.11 (m, 1H), 2.06 – 1.99 (m, 1H), 1.94 – 1.88 (m, 3H), 1.68 – 1.61 (m, 1H). **^{13}C NMR (151 MHz, $CDCl_3$)** δ 153.7, 132.5, 130.4, 127.5, 125.9, 124.4, 121.6, 116.2, 72.9, 28.4, 25.1, 18.9. **HRMS** (ESI-TOF) (m/z): calcd for $C_{12}H_{13}ClNaO$ ($[M+Na]^+$), 231.0547, found, 231.0551.

2-methyl-4-phenylchromane (4a)

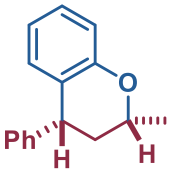

Condition B. Yellow oil, 83% yield (37.2 mg), 3.3:1 dr. **^{1}H NMR (600 MHz, $CDCl_3$)** δ 7.36 (d, *J* = 7.2 Hz, 0.3H), 7.31 (t, *J* = 7.8 Hz, 2.3H), 7.26 – 7.23 (m, 1.6H), 7.18 (d, *J* = 7.2 Hz, 2.3H), 7.08 (t, *J* = 6.6 Hz, 1.6H), 6.95 (d, *J* = 8.4 Hz, 0.3H), 6.90 (d, *J* = 8.4 Hz, 0.3H), 6.84 (d, *J* = 8.4 Hz, 1H), 6.75 – 6.68 (m, 2H), 4.33 – 4.26 (m, 1H), 4.23 – 4.19 (m, 0.3H), 4.19 – 4.14 (m, 1H), 4.14 – 4.09 (m, 0.3H), 2.21 – 2.15 (m, 1H), 2.13 – 2.06 (m, 0.3H), 2.02 – 1.97 (m, 0.3H), 1.97 – 1.89 (m, 1H), 1.42 (d, *J* = 6.0 Hz, 3H), 1.32 (d, *J* = 6.0 Hz, 0.9H). **^{13}C NMR (151 MHz, $CDCl_3$)** δ 155.5, 155.4, 146.7, 145.0, 130.9, 129.8, 129.4, 128.63, 128.58 , 128.5, 128.3, 127.9, 127.6, 126.6, 126.2, 125.7, 120.2, 116.8, 116.6, 116.1, 74.5, 72.3, 67.3, 43.1, 40.1, 37.7, 21.6, 21.1. **HRMS** (ESI-TOF) (m/z): calcd for $C_{16}H_{16}NaO$ ($[M+Na]^+$), 247.1093, found, 247.1090.

2-methyl-4-phenyl-8-propylchromane (4b)

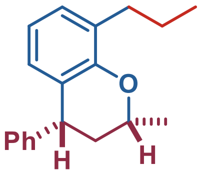

Condition B. Yellow oil, 81% yield (43.1 mg), 2:1 dr. **^{1}H NMR (500 MHz, $CDCl_3$)** δ 7.31 – 7.26 (m, 2.5H), 7.25 – 7.22 (m, 1.5H), 7.18 (d, J = 7.5 Hz, 2.5H), 7.08 (d, J = 7.0 Hz, 1H), 7.02 (dd, J = 7.0, 1.5 Hz, 0.5H), 6.95 (d, J = 7.0 Hz, 1H), 6.80 – 6.72 (m, 1H), 6.65 (t, J = 7.5 Hz, 1H), 6.54 (d, J = 8.0 Hz, 1H), 4.29 – 4.22 (m, 1H), 4.22 – 4.14 (m, 1.5H), 4.14 – 4.07 (m, 0.5H), 2.68 – 2.52 (m, 3H), 2.21 – 2.15 (m, 1H), 2.11 – 2.03 (m, 0.5H), 2.01 – 1.96 (m, 0.5H), 1.95 – 1.85 (m, 1H), 1.67 – 1.61 (m, 3H), 1.42 (d, J = 6.5 Hz, 3H), 1.32 (d, J = 6.5 Hz, 1.5H), 1.00 – 0.94 (m, 4.5H). **^{13}C NMR (151 MHz, $CDCl_3$)** δ 153.4, 153.3, 147.1, 145.5, 130.4, 130.2, 128.7, 128.6, 128.5, 128.4, 128.2, 128.1, 127.8, 127.4, 126.5, 126.1, 125.2, 119.4, 72.0, 67.2, 43.3, 40.4, 40.2, 37.8, 32.19, 32.15, 23.0, 22.9, 21.7, 21.2, 14.2. **HRMS** (ESI-TOF) (m/z): calcd for $C_{19}H_{22}NaO$ ($[M+Na]^+$), 289.1563, found, 289.1566.

8-isopropyl-2-methyl-4-phenylchromane (4c)

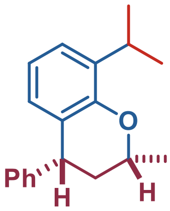

Condition B. Yellow oil, 73% yield (38.9 mg), 2:1 dr. **^{1}H NMR (500 MHz, $CDCl_3$)** δ 7.31 – 7.26 (m, 2.5H), 7.24 (d, J = 7.5 Hz, 1.5H), 7.20 – 7.17 (m, 2.5H), 7.11 – 7.07 (m, 1.5H), 7.03 (d, J = 7.0 Hz, 1H), 6.79 (d, J = 7.0 Hz, 1H), 6.69 (t, J = 7.5 Hz, 1H), 6.54 (d, J = 7.5 Hz, 1H), 4.31 – 4.23 (m, 1H), 4.23 – 4.15 (m, 1.5H), 4.14 – 4.08 (m, 0.5H), 3.38 – 3.30 (m, 1.5H), 2.22 – 2.16 (m, 1H), 2.12 – 2.04 (m, 0.5H), 2.02 – 1.96 (m, 0.5H), 1.96 – 1.86 (m, 1H), 1.43 (d, J = 6.0 Hz, 3H), 1.33 (d, J = 6.0 Hz, 1.5H), 1.27 – 1.20 (m, 9H). **^{13}C NMR (151 MHz, $CDCl_3$)** δ 152.7, 147.1, 145.5, 136.3, 136.1, 128.7, 128.6, 128.5, 128.2, 127.2, 126.5, 126.1, 125.2, 124.2, 124.0, 119.58, 119.56, 72.1, 67.2, 43.4, 40.5, 40.1, 37.7, 26.8, 26.8, 22.9, 22.7, 22.6, 22.4, 21.7, 21.2. **HRMS** (ESI-TOF) (m/z): calcd for $C_{19}H_{22}NaO$ ($[M+Na]^+$), 289.1563, found, 289.1559.

2,6-dimethyl-4-phenylchromane (4d)

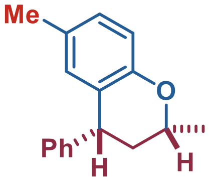

Condition B. Yellow oil, 95% yield (45.2 mg), 2.5:1 dr. **^{1}H NMR (500 MHz, $CDCl_3$)** δ 7.34 – 7.21 (m, 4.6H), 7.19 – 7.16 (m, 2.2H), 7.09 (d, J = 7.5 Hz, 1H), 6.96 (d, J = 8.0 Hz, 0.4H), 6.89 (d, J = 8.5 Hz, 1H), 6.80 (d, J = 8.0 Hz, 0.4H), 6.75 (d, J = 8.0 Hz, 1H), 6.51 (s, 1H), 4.28 – 4.20 (m, 1H), 4.18 – 4.08 (m, 1.8H), 2.19 (s, 1H), 2.18 – 2.13 (m, 1H), 2.10 (s, 3H), 2.09 – 2.04 (m, 0.4H), 1.99 – 1.85 (m, 1.4H), 1.40 (d, J = 6.5 Hz, 3H), 1.30 (d, J = 6.5 Hz, 1.2H). **^{13}C NMR (151 MHz, $CDCl_3$)** δ 153.3, 146.9, 145.1, 131.0, 130.0, 129.3, 129.2, 128.7, 128.6, 128.5, 128.3, 128.2, 126.5, 126.1, 125.2, 122.4, 116.5, 116.4, 72.2, 67.2, 43.1, 40.4, 40.2, 37.9, 21.6, 21.2, 20.5, 20.4. **HRMS** (ESI-TOF) (m/z): calcd for $C_{17}H_{18}NaO$ ($[M+Na]^+$), 261.1250, found, 261.1253.

6-isopropyl-2-methyl-4-phenylchromane (4e)

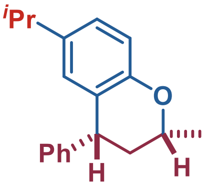

Condition B. Yellow oil, 82% yield (43.7 mg), 1.4:1 dr. **^{1}H NMR (500 MHz, $CDCl_3$)** δ 7.35 – 7.28 (m, 4H), 7.24 (d, J = 8.5 Hz, 1H), 7.21 – 7.16 (m, 2.4H), 7.09 (d, J = 7.5 Hz, 1.4H), 7.04 (d, J = 8.5Hz, 0.7H), 6.97 (d, J = 9.0 Hz, 1H), 6.84 (d, J = 8.5 Hz, 0.7H), 6.82 – 6.77 (m, 1.4H), 6.56 (s, 1H), 4.32 – 4.23 (m, 1H), 4.23 – 4.12 (m, 1.7H), 4.12 – 4.03 (m, 0.7H), 2.81 – 2.72 (m, 0.7H),

2.71 – 2.61 (m, 1H), 2.22 – 2.14 (m, 1H), 2.13 – 2.06 (m, 0.7H), 1.98 – 1.85 (m, 1.7H), 1.40 (d, *J* = 6.0 Hz, 3H), 1.30 (d, *J* = 6.5 Hz, 2.1H), 1.16 (t, *J* = 7.0 Hz, 4.2H), 1.11 – 1.03 (m, 6H).**^{13}C NMR (151 MHz, CDCl$_3$)** δ 153.5, 153.4, 146.9, 145.1, 140.5, 140.4, 128.7, 128.6, 128.5, 128.5, 128.2, 127.7, 126.5, 126.1, 125.9, 125.3, 125.0, 122.2, 116.5, 116.3, 72.2, 67.1, 43.2, 40.5, 40.3, 38.0, 33.2, 24.3, 24.1, 24.0, 21.6, 21.2. **HRMS** (ESI-TOF) (m/z): calcd for $C_{19}H_{22}NaO$ ([M+Na]$^+$), 289.1563, found, 289.1560.

6-(tert-butyl)-2-methyl-4-phenylchromane (4f)

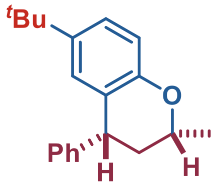

Condition B. Yellow oil, 90% yield (50.4 mg), 1.4:1 dr. **^{1}H NMR (500 MHz, CDCl$_3$)** δ 7.36 (d, *J* = 7.2 Hz, 0.7H), 7.33 – 7.29 (m, 2.1H), 7.27 – 7.25 (m, 0.7H), 7.25 – 7.23 (m, 1H), 7.23 – 7.21 (m, 1H), 7.21 – 7.20 (m, 0.7H), 7.19 – 7.16 (m, 2H), 7.12 (dd, *J* = 8.4, 1.8 Hz, 1H), 7.08 (d, *J* = 7.8 Hz, 1H), 6.95 (d, *J* = 2.4 Hz, 0.7H), 6.84 (d, *J* = 9.0 Hz, 0.7H), 6.78 (d, *J* = 8.4 Hz, 1H), 6.74 – 6.71 (m, 1H), 4.32 – 4.24 (m, 1H), 4.23 – 4.193 (m, 0.7H), 4.19 – 4.14 (m, 1H), 4.10 – 4.03 (m, 0.7H), 2.20 – 2.14 (m, 1H), 2.13 – 2.06 (m, 0.7H), 2.00 – 1.95 (m, 0.7H), 1.93 – 1.85 (m, 1H), 1.40 (d, *J* = 6.6 Hz, 3H), 1.30 (d, *J* = 6.0 Hz, 2.1H), 1.22 (s, 6.3H), 1.13 (s, 9H). **^{13}C NMR (151 MHz, CDCl$_3$)** δ 153.2, 146.9, 145.1, 142.8, 142.7, 128.6, 128.5, 128.4, 128.2, 127.5, 126.7, 126.5, 126.1, 126.0, 125.0, 124.5, 121.8, 116.1, 115.9, 115.4, 74.5, 72.2, 67.2, 43.3, 40.50, 40.47, 38.1, 34.0, 31.5, 31.4, 21.6 , 21.3. **HRMS** (ESI-TOF) (m/z): calcd for $C_{20}H_{24}NaO$ ([M+Na]$^+$), 303.1719, found, 303.1724.

2-methyl-4,6-diphenylchromane (4g)

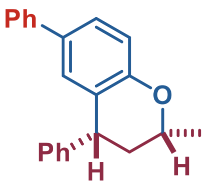

Condition B. Yellow oil, 88% yield (52.8 mg), 2.5:1 dr. **^{1}H NMR (500 MHz, CDCl$_3$)** δ 7.47 (d, *J* = 7.5 Hz, 1H), 7.44 – 7.41 (m, 0.4H), 7.37 – 7.32 (m, 4.2H), 7.32 – 7.26 (m, 5H), 7.25 – 7.23 (m, 1.2H), 7.22– 7.17 (m, 4H), 7.12 (d, *J* = 7.0 Hz, 1H), 7.00 – 6.96 (m, 0.4H), 6.96 – 6.89 (m, 2H), 4.36 – 4.29 (m,1H), 4.29 – 4.25 (m, 0.4H), 4.24 – 4.18 (m, 1H), 4.17 – 4.11 (m, 0.4H), 2.25 – 2.17 (m, 1H), 2.16 – 2.09 (m, 0.4H), 2.05 – 1.99 (m, 0.4H), 1.99 – 1.89 (m, 1H), 1.43 (dd, *J* = 6.5, 2.5 Hz, 3H), 1.34 (dd, *J* = 6.5, 2.5 Hz, 1.2H). **^{13}C NMR (151 MHz, CDCl$_3$)** δ 155.1, 146.5, 144.7, 140.9, 140.7, 133.2, 133.1, 129.4, 128.7, 128.6, 128.51, 128.50, 128.4, 128.3, 126.7, 126.61, 126.55, 126.5, 126.44, 126.39, 126.3, 125.8, 123.0, 117.2, 117.0, 72.5, 67.5, 43.1, 40.3, 40.2, 37.8, 21.6, 21.2. **HRMS** (ESI-TOF) (m/z): calcd for $C_{22}H_{20}NaO$ ([M+Na]$^+$), 323.1406, found, 323.1405.

2-methyl-6-phenoxy-4-phenylchromane (4h)

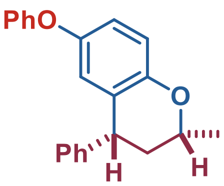

Condition B. Yellow oil, 99% yield (62.6 mg), 2.5:1 dr. **^{1}H NMR (500 MHz, CDCl$_3$)** δ 7.31 – 7.26 (m, 2.84H), 7.26 – 7.24 (m, 1H), 7.23 – 7.18 (m, 3.68H), 7.17 – 7.15 (m, 2H), 7.08 (d, *J* = 7.5 Hz, 1H), 7.01 – 6.93 (m, 1.42H), 6.91 – 6.87 (m, 1.42H), 6.84 – 6.81 (m, 2.84H), 6.78 (dd, *J* = 8.5, 2.5 Hz, 1H), 6.68 (d, *J* = 2.5 Hz, 0.42H), 6.48 – 6.46 (m, 1H), 4.33 – 4.25 (m, 1H), 4.19 – 4.08 (m, 1.84H), 2.20 – 2.15 (m, 1H), 2.12 – 2.06 (m, 0.42H), 2.01 – 1.96 (m, 0.42H), 1.96 – 1.87 (m, 1H), 1.42 (d, *J* = 6.5 Hz, 3H), 1.32 (d, *J* = 6.5 Hz, 1.26H). **^{13}C NMR (151 MHz, CDCl$_3$)** δ 158.6, 151.86, 151.81, 149.4, 149.1, 146.3, 144.5, 129.5, 129.4, 128.6, 128.5, 128.4, 128.3, 126.8, 126.7, 126.3, 123.9, 122.1, 122.0, 121.8, 121.4, 120.0, 119.6, 117.8, 117.5,

117.3, 117.0, 72.4, 67.5, 43.2, 40.3, 40.0, 37.6, 21.5, 21.1. **HRMS** (ESI-TOF) (m/z): calcd for $C_{22}H_{20}NaO_2$ ([M+Na]$^+$), 339.1356, found, 339.1359.

6-methoxy-2-methyl-4-phenylchromane (4i)

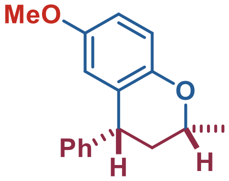

Condition B. Yellow oil, 94% yield (47.8 mg), 1.3:1 dr. **^{1}H NMR (600 MHz, $CDCl_3$)** δ 7.30 – 7.27 (m, 4H), 7.35 – 7.22 (m, 1.6H), 7.20 – 7.18 (m, 1.6H), 7.09 (d, *J* = 7.2 Hz, 1.8H), 6.83 (d, *J* = 8.4 Hz, 0.8H), 6.78 (d, *J* = 9.0 Hz, 1H), 6.77 – 6.74 (m, 0.8H), 6.69 – 6.65 (m, 1H), 6.47 (d, *J* = 3.0 Hz, 0.8H), 6.25 (d, *J* = 3.0 Hz, 1H), 4.25 – 4.19 (m, 1H), 4.18 – 4.15 (m, 0.8H), 4.15 – 4.10 (m, 1H), 4.10 – 4.04 (m, 1H), 3.65 (s, 2.4H), 3.57 (s, 3H), 2.18 – 2.13 m, 1H), 2.11 – 2.04 (m, 0.8H), 1.97 – 1.93 (m, 0.8H), 1.93 – 1.86 (m, 1H), 1.39 (d, *J* = 6.6 Hz, 3H), 1.29 (d, *J* = 6.0 Hz, 2.4H). **^{13}C NMR (151 MHz, $CDCl_3$)** δ 153.2, 153.1, 149.59, 149.55, 146.6, 144.8, 128.60 , 128.57, 128.5, 128.3, 126.6, 126.24, 126.19, 123.2, 117.4, 117.1, 114.7, 114.64, 114.60, 113.4, 72.1, 67.1, 55.6, 55.5, 43.3, 40.5, 40.2, 37.9, 21.6, 21.1. **HRMS** (ESI-TOF) (m/z): calcd for $C_{17}H_{18}NaO_2$ ([M+Na]$^+$), 277.1199, found, 277.1201.

6-fluoro-2-methyl-4-phenylchromane (4j)

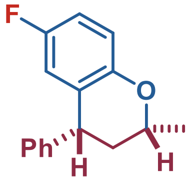

Condition B. Yellow oil, 99% yield (47.9 mg), 13:1 dr. **^{1}H NMR (500 MHz, $CDCl_3$)** δ 7.32 (t, *J* = 7.5 Hz, 2H), 7.26 (d, *J* = 7.5 Hz, 1H), 7.16 (d, *J* = 7.0 Hz, 2H), 6.81 – 6.74 (m, 2H), 6.40 (d, *J* = 8.5 Hz, 1H), 4.2 9 – 4.21 (m, 1H), 4.15 – 4.08 (m , 1H), 2.21 – 2.14 (m, 1H), 1.95 – 1.86 (m, 1H), 1.41 (d, *J* = 6.0 Hz, 3H). **^{13}C NMR (151 MHz, $CDCl_3$)** δ 156.7 (d, *J* = 237.1 Hz), 151.4, 144.2, 128.7, 128.4, 126.9, 126.8 (d, *J* = 6.9 Hz), 117.4 (d, *J* = 8.0 Hz), 115.6 (d, *J* = 23.1 Hz), 114.4 (d, *J* = 23.2 Hz), 72.4, 43.2, 39.6, 21.5. **^{19}F NMR (565 MHz, $CDCl_3$)** *δ* = -123.769 (s, 1F). **HRMS** (ESI-TOF) (m/z): calcd for $C_{16}H_{15}FNaO$ ([M+Na]$^+$), 265.0999, found, 265.0995.

6-chloro-2-methyl-4-phenylchromane (4k)

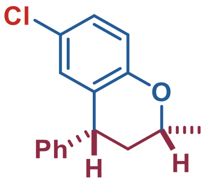

Condition B. Yellow oil, 97% yield (50.1 mg), >20:1 dr. **^{1}H NMR (500 MHz, $CDCl_3$)** δ 7.32 (d, *J* = 7.5 Hz, 2H), 7.27 (d, *J* = 7.5 Hz, 1H), 7.16 (d, *J* = 8.0 Hz, 2H), 7.03 (dd, *J* = 9.0, 3.0 Hz, 1H), 6.77 (d, *J* = 8.5 Hz, 1H), 6.69 – 6.65 (m, 1H), 4.32 – 4.22 (m, 1H), 4.15 – 4.07 (m, 1H), 2.21 – 2.14 (m, 1H), 1.96 – 1.84 (m, 1H), 1.41 (d, *J* = 6.0 Hz, 3H). **^{13}C NMR (151 MHz, $CDCl_3$)** δ 154.1, 144.0, 129.3, 128.8, 128.4, 127.6, 127.3, 126.9, 124.9, 118.0, 72.6, 43.0, 39.7, 21.5. **HRMS** (ESI-TOF) (m/z): calcd for $C_{16}H_{15}ClNaO$ ([M+Na]$^+$), 281.0704, found, 281.0701.

8-chloro-2-methyl-4-phenylchromane (4l)

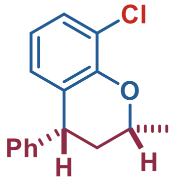

Condition B. Yellow oil, 85% yield (43.9 mg), >20:1 dr. **^{1}H NMR (500 MHz, $CDCl_3$)** δ 7.31 (d, *J* = 7.5 Hz, 2H), 7.26 (d, *J* = 8.5 Hz, 1H), 7.17 (t, *J* = 7.5 Hz, 3H), 6.65 (d, *J* = 7.5 Hz, 1H), 6.60 (d, *J* = 7.5 Hz, 1H), 4.41 – 4.32 (m, 1H), 4.21 – 4.14 (m, 1H), 2.26 – 2.19 (m, 1H), 2.01 – 1.92 (m, 1H), 1.50 (d, *J* = 6.5 Hz, 3H). **^{13}C NMR (151 MHz, $CDCl_3$)** δ 151.2, 144.4, 128.7, 128.5, 128.3, 128.2, 127.5, 126.8, 121.4, 120.1, 73.3, 43.2, 39.7, 21.4. **HRMS** (ESI-TOF) (m/z): calcd for $C_{16}H_{15}ClNaO$ ([M+Na]$^+$), 281.0704, found, 281.0707.

6-bromo-2-methyl-4-phenylchromane (4m)

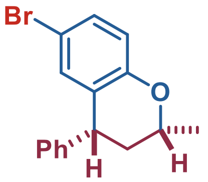

Condition B. Yellow oil, 99% yield (59.8 mg), >20:1 dr. **^{1}H NMR (500 MHz, $CDCl_3$)** δ 7.32 (t, J = 7.0 Hz, 2H), 7.27 (d, J = 7.5 Hz, 1H), 7.20 – 7.13 (m, 3H), 6.81 (s, 1H), 6.72 (d, J = 8.5 Hz, 1H), 4.30 – 4.21 (m, 1H), 4.14 – 4.07 (m, 1H), 2.20 – 2.13 (m, 1H), 1.94 – 1.84 (m, 1H), 1.41 (d, J = 6.5 Hz, 3H). **^{13}C NMR (151 MHz, $CDCl_3$)** δ 154.6, 144.0, 132.2, 130.5, 128.8, 128.4, 127.9, 126.9, 118.5, 112.3, 72.6, 42.9, 39.6, 21.5. **HRMS** (ESI-TOF) (m/z): calcd for $C_{16}H_{15}BrNaO$ ([M+Na]$^+$), 325.0198, found, 325.0201.

8-bromo-2-methyl-4-phenylchromane (4n)

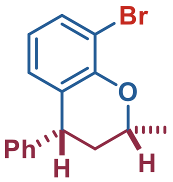

Condition B. Yellow oil, 60% yield (36.2 mg), >20:1 dr. **^{1}H NMR (500 MHz, $CDCl_3$)** δ 7.32 (t, J = 7.0 Hz, 2H), 7.27 (d, J = 7.5 Hz, 1H), 7.20 – 7.13 (m, 3H), 6.81 (s, 1H), 6.72 (d, J = 8.5 Hz, 1H), 4.30 – 4.21 (m, 1H), 4.14 – 4.07 (m, 1H), 2.20 – 2.13 (m, 1H), 1.94 – 1.84 (m, 1H), 1.41 (d, J = 6.5 Hz, 3H). **^{13}C NMR (151 MHz, $CDCl_3$)** δ 154.6, 144.0, 132.2, 130.5, 128.8, 128.4, 127.9, 126.9, 118.5, 112.3, 72.6, 42.9, 39.6, 21.5. **HRMS** (ESI-TOF) (m/z): calcd for $C_{16}H_{15}BrNaO$ ([M+Na]$^+$), 325.0198, found, 325.0199.

5,6,7-trifluoro-2-methyl-4-phenylchromane (4o)

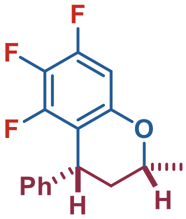

Condition B. Yellow oil, 47% yield (26.1 mg), >20:1 dr. **^{1}H NMR (600 MHz, $CDCl_3$)** δ 7.29 (t, J = 7.2 Hz, 2H), 7.25 – 7.20 (m, 1H), 7.12 (d, J = 6.6 Hz, 2H), 6.55 – 6.50 (m, 1H), 4.21 – 4.15 (m, 1H), 4.14 – 4.07 (m, 1H), 2.35 – 2.25 (m, 1H), 1.80 – 1.70 (m, 1H), 1.37 (d, J = 6.0 Hz, 3H). **^{13}C NMR (151 MHz, $CDCl_3$)** δ 151.8 (td, J = 11.3, 2.6 Hz), 150.9 (td, J = 11.2, 5.7 Hz), 149.2 (td, J = 15.9, 5.7 Hz), 144.4, 135.1 (dt, J = 243.4, 15.7 Hz), 128.7, 126.7, 126.6, 111.0 (d, J = 11.1 Hz), 100.6 (dd, J = 20.0, 3.5 Hz), 72.9, 40.9, 38.7, 20.9. **^{19}F NMR (565 MHz, $CDCl_3$)** δ = -130.86 ~ -130.98 (m, 1F), -136.65 ~ -136.85 (m, 1F), -170.79 ~ -170.01 (m, 1F). **HRMS** (ESI-TOF) (m/z): calcd for $C_{16}H_{13}F_3NaO$ ([M+Na]$^+$), 301.0811, found, 301.0807.

2-methyl-4-phenyl-6-(trifluoromethyl)chromane (4p)

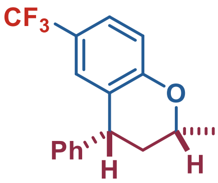

Condition B. Yellow oil, 67% yield (39.1 mg), >20:1 dr. **^{1}H NMR (500 MHz, $CDCl_3$)** δ 7.36 – 7.31 (m, 3H), 7.28 (d, J = 7.5 Hz, 1H), 7.16 (d, J = 7.0 Hz, 2H), 6.97 (s, 1H), 6.90 (d, J = 8.5 Hz, 1H), 4.38 – 4.30 (m, 1H), 4.19 – 4.12 (m, 1H), 2.26 – 2.18 (m, 1H), 1.98 – 1.88 (m, 1H), 1.44 (d, J = 6.5 Hz, 3H). **^{13}C NMR (151 MHz, $CDCl_3$)** δ 158.1, 143.7, 128.9, 128.4, 127.1, 126.0, 124.8 (q, J = 3.6 Hz), 124.4 (q, J = 271.8 Hz), 122.3 (q, J = 32.3 Hz), 117.1, 73.0, 42.9, 39.6, 21.4. **^{19}F NMR (565 MHz, $CDCl_3$)** δ = -61.410 (s, $1CF_3$). **HRMS** (ESI-TOF) (m/z): calcd for $C_{17}H_{15}F_3Na$ ([M+Na]$^+$), 315.0967, found, 315.0972.

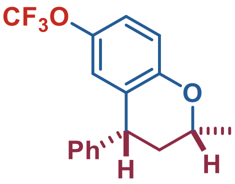

2-methyl-4-phenyl-6-(trifluoromethoxy)chromane (4q)

Condition B. Yellow oil, 83% yield (51.1 mg), >20:1 dr. **^{1}H NMR (500 MHz, $CDCl_3$)** δ 7.33 (t, J = 7.5 Hz, 2H), 7.27 (d, J = 7.0 Hz, 1H), 7.16 (d, J = 7.0 Hz, 2H), 6.95 (d, J = 10.5 Hz, 1H), 6.83 (d, J = 9.0 Hz, 1H), 6.56 (s, 1H), 4.34 – 4.25 (m, 1H), 4.18 – 4.10 (m, 1H), 2.25 – 2.15 (m, 1H), 1.95 –

1.85 (m, 1H), 1.42 (d, J = 6.0 Hz, 3H). **^{13}C NMR (151 MHz, CDCl$_3$)** δ 154.0, 143.9, 142.1 (d, J = 1.8 Hz), 128.8, 128.4, 127.0, 126.8, 122.6, 120.7, 120.4 (q, J = 255.9Hz), 117.5, 72.7, 43.1, 39.6, 21.5. **^{19}F NMR (565 MHz, CDCl$_3$)** δ = -58.43 (s, 1OCF$_3$). **HRMS** (ESI-TOF) (m/z): calcd for $C_{17}H_{15}F_3NaO_2$ ([M+Na]$^+$), 331.0916, found, 331.0911.

2-(2-methyl-4-phenylchroman-6-yl)acetonitrile (4r)

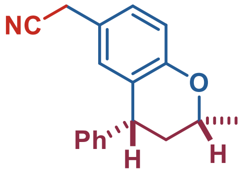

Condition B. Yellow oil, 84% yield (44.2 mg), 17:1 dr. **^{1}H NMR (500 MHz, CDCl$_3$)** δ 7.33 (t, J = 7.0 Hz, 2H), 7.27 (d, J = 6.5 Hz, 1H), 7.16 (d, J = 7.5 Hz, 2H), 7.05 (d, J = 8.0 Hz, 1H), 6.84 (d, J = 8.5 Hz, 1H), 6.60 (s, 1H), 4.34 – 4.24 (m, 1H), 4.18 – 4.08 (m, 1H), 3.53 – 3.42 (m, 2H), 2.24 – 2.14 (m, 1H), 1.96 – 1.86 (m, 1H), 1.42 (d, J = 6.0 Hz, 3H). **^{13}C NMR (151 MHz, CDCl$_3$)** δ 155.2, 144.3, 129.4, 128.7, 128.4, 127.2, 126.9, 126.4, 121.3, 118.2, 117.4, 72.5, 42.9, 39.8, 22.8, 21.5. **HRMS** (ESI-TOF) (m/z): calcd for $C_{18}H_{17}NNaO$ ([M+Na]$^+$), 286.1202, found, 286.1198.

4,4,5,5-tetramethyl-2-(2-methyl-4-phenylchroman-6-yl)-1,3,2-dioxaborolane (4s)

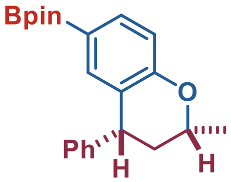

Condition B. White solid, 90% yield (63.0 mg), >20:1 dr. **^{1}H NMR (500 MHz, CDCl$_3$)** δ 7.57 (d, J = 8.0 Hz, 1H), 7.31 (t, J = 7.0 Hz, 2H), 7.26 – 7.22 (m, 2H), 7.17 (d, J = 7.5 Hz, 2H), 6.85 (d, J = 8.5 Hz, 1H), 4.31 – 4.22 (m, 1H), 4.20 – 4.13 (m, 1H), 2.25 – 2.15 (m, 1H), 1.92 – 1.80 (m, 1H), 1.41 (d, J = 6.5 Hz, 3H), 1.24 (s, 12H). **^{13}C NMR (151 MHz, CDCl$_3$)** δ 158.4, 145.1, 136.7, 134.6, 128.6, 128.5, 126.5, 124.8, 116.3, 83.3, 72.4, 42.8, 40.9, 24.8, 24.7, 21.5. ^{11}B NMR (193 MHz, CDCl$_3$) δ 30.42. **HRMS** (ESI-TOF) (m/z): calcd for $C_{22}H_{27}BNaO_3$ ([M+Na]$^+$), 373.1945, found, 373.1951.

2-methyl-6-(methylsulfonyl)-4-phenylchromane (4t)

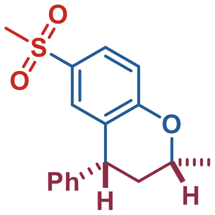

Condition B. Yellow oil, 35% yield (21.1 mg), >20:1 dr. **^{1}H NMR (600 MHz, CDCl$_3$)** δ 7.65 (dd, J = 9.0, 1.8 Hz, 1H), 7.34 (t, J = 7.2 Hz, 2H), 7.31 – 7.29 (m, 1H), 7.29 – 7.27 (m, 1H), 7.15 (d, J = 7.2 Hz, 2H), 6.96 (d, J = 8.4 Hz, 1H), 4.42 – 4.35 (m, 1H), 4.20 – 4.14 (m, 1H), 2.89 (s, 3H), 2.30 – 2.22 (m, 1H), 1.99 – 1.89 (m, 1H), 1.46 (d, J = 6.0 Hz, 3H). **^{13}C NMR (151 MHz, CDCl$_3$)** δ 159.9, 143.2, 129.7, 129.0, 128.3, 127.3, 127.2, 126.5, 117.6, 116.2, 73.4, 44.7, 42.8, 39.3, 21.3. **HRMS** (ESI-TOF) (m/z): calcd for $C_{17}H_{18}NaO_3S$ ([M+Na]$^+$), 325.0869, found, 325.0872.

methyl 2-methyl-4-phenylchromane-6-carboxylate (4u)

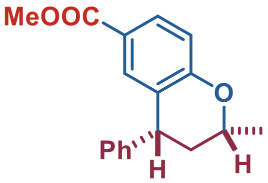

Condition B. Yellow oil, 75% yield (42.3 mg), >20:1 dr. **^{1}H NMR (600 MHz, CDCl$_3$)** δ 7.79 (dd, J = 8.4, 1.8 Hz, 1H), 7.45 (s, 1H), 7.33 (t, J = 7.2 Hz, 2H), 7.27 (d, J = 7.2 Hz, 1H), 7.16 (d, J = 7.2Hz, 2H), 6.86 (d, J = 8.4 Hz, 1H), 4.40 – 4.30 (m, 1H), 4.18 – 4.13 (m, 1H), 3.76 (s, 3H), 2.24 – 2.18 (m, 1H), 1.96 – 1.87 (m, 1H), 1.44 (d, J = 6.6 Hz, 3H). **^{13}C NMR (151 MHz, CDCl$_3$)** δ 166.9, 159.5, 144.1, 131.9, 129.4, 128.8, 128.4, 126.9, 125.5, 122.1, 116.7, 73.0, 51.7, 42.9, 39.8, 21.4. **HRMS** (ESI-TOF) (m/z): calcd for $C_{18}H_{18}NaO_3$ ([M+Na]$^+$), 305.1148, found, 305.1143.

N,N,2-trimethyl-4-phenylchromane-6-carboxamide (4v)

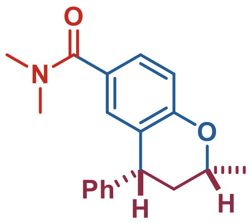

Condition B. Yellow oil, 88% yield (51.9 mg), >20:1 dr. **^{1}H NMR (600 MHz, $CDCl_3$)** δ 7.30 (t, *J* = 7.2 Hz, 2H), 7.26 – 7.23 (m, 1H), 7.22 – 7.19 (m, 1H), 7.17 (d, *J* = 7.2 Hz, 2H), 6.84 (d, *J* = 8.4 Hz, 1H), 6.81 – 6.79 (m, 1H), 4.36 – 4.28 (m, 1H), 4.19 – 4.12 (m, 1H), 2.96 (s, 3H), 2.83 (s, 3H), 2.24 – 2.18 (m, 1H), 1.96 – 1.89 (m, 1H), 1.43 (d, *J* = 6.0 Hz, 3H). **^{13}C NMR (151 MHz, $CDCl_3$)** δ 171.5, 156.5, 144.3, 129.3, 128.6, 128.4, 127.8, 127.2, 126.8, 125.3, 116.5, 72.7, 42.8, 39.7, 21.4. **HRMS** (ESI-TOF) (m/z): calcd for $C_{19}H_{21}NNaO_2$ ($[M+Na]^+$), 318.1465, found, 318.1470.

1-(2-methyl-4-phenylchroman-6-yl)ethan-1-one (4w)

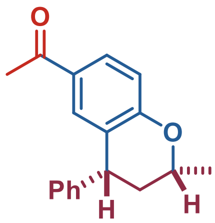

Condition B. Yellow oil, 75% yield (39.9 mg), >20:1 dr. **^{1}H NMR (600 MHz, $CDCl_3$)** δ 7.73 (dd, *J* = 8.4, 1.2 Hz, 1H), 7.37 (s, 1H), 7.32 (t, *J* = 7.2 Hz, 2H), 7.28 – 7.24 (m, 1H), 7.16 (d, *J* = 6.6 Hz, 2H), 6.87 (d, *J* = 8.4 Hz, 1H), 4.40 – 4.32 (m, 1H), 4.20 – 4.13 (m, 1H), 2.35 (s, 3H), 2.25 – 2.19 (m, 1H), 1.97 – 1.88 (m, 1H), 1.44 (d, *J* = 6.0 Hz, 3H). **^{13}C NMR (151 MHz, $CDCl_3$)** δ 196.8, 159.7, 143.9, 130.9, 128.8, 128.4, 128.3, 127.0, 125.5, 116.8, 115.4, 73.1, 42.8, 39.7, 26.2, 21.4. **HRMS** (ESI-TOF) (m/z): calcd for $C_{18}H_{18}NaO_2$ ($[M+Na]^+$), 289.1199, found, 289.1203.

2-methyl-4-phenylchromane-6-carbaldehyde (4x)

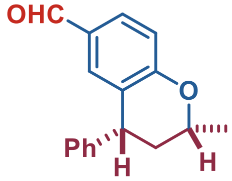

Condition B. Yellow oil, 70% yield (35.3 mg), >20:1 dr. **^{1}H NMR (600 MHz, $CDCl_3$)** δ 9.67 (s, 1H), 7.65 (dd, *J* = 8.4, 1.8 Hz, 1H), 7.34 (t, *J* = 7.2 Hz, 2H), 7.29 (d, *J* = 7.2 Hz, 1H), 7.26 – 7.23 (m, 1H), 7.17 (d, *J* = 7.2 Hz, 2H), 6.94 (d, *J* = 8.4 Hz, 1H), 4.44 – 4.36 (m, 1H), 4.22 – 4.14 (m, 1H), 2.28 – 2.20 (m, 1H), 2.01 – 1.91 (m, 1H), 1.46 (d, *J* = 6.0 Hz, 3H). **^{13}C NMR (151 MHz, $CDCl_3$)** δ 190.9, 160.8, 143.6, 133.0, 129.5, 129.2, 128.9, 128.4, 127.1, 126.3, 117.6, 73.4, 42.7, 39.4, 21.4. **HRMS** (ESI-TOF) (m/z): calcd for $C_{17}H_{16}NaO_2$ ($[M+Na]^+$), 275.1043, found, 275.1039.

2-methyl-4-phenylchromane-7-carbonitrile (*cis*-4y)

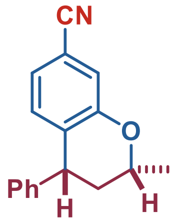

Method B , yellow oil, 45% yield, 1.7:1 dr. **^{1}H NMR (600 MHz, $CDCl_3$)** δ 7.26 (t, *J* = 7.2 Hz, 2H), 7.21 (d, *J* = 7.2 Hz, 1H), 7.07 (d, *J* = 6.6 Hz, 2H), 7.04 (d, *J* = 1.8 Hz, 1H), 6.91 (dd, *J* = 7.8, 1.2 Hz, 1H), 6.71 (dd, *J* = 7.8, 0.6 Hz, 1H), 4.29 – 4.22 (m, 1H), 4.11 – 4.05 (m, 1H), 2.20 – 2.12 (m, 1H), 1.91 – 1.82 (m, 1H), 1.37 (d, *J* = 6.0 Hz, 3H). **^{13}C NMR (151 MHz, $CDCl_3$)** δ 155.7, 143.4, 131.5, 130.7, 128.9, 128.4, 127.2, 123.5, 120.4, 118.8, 111.1, 73.0, 43.1, 39.1, 21.3. **HRMS** (ESI-TOF) (m/z): calcd for $C_{17}H_{15}NNaO$ ($[M+Na]^+$), 272.1046, found, 272.1049.

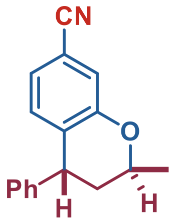

2-methyl-4-phenylchromane-7-carbonitrile (*trans*-4y)

Method B , yellow oil, 45% yield, 1.7:1 dr. **^{1}H NMR (600 MHz, $CDCl_3$)** δ 7.31 (t, *J* = 7.2 Hz, 2H), 7.25 (d, *J* = 3.6 Hz, 1H), 7.20 – 7.15 (m, 2H), 7.13 – 7.08 (m,

3H), 4.42 – 4.35 (m, 1H), 4.21 – 4.13 (m, 1H), 2.45 – 2.35 (m, 1H), 1.91 – 1.82 (m, 1H), 1.40 (d, *J* = 6.6 Hz, 3H). **^{13}C NMR (151 MHz, $CDCl_3$)** δ 157.1, 144.5, 128.7, 128.6, 128.2, 128.0, 127.0, 126.9, 122.1, 117.4, 113.8, 72.5, 42.0, 41.8, 21.0. **HRMS** (ESI-TOF) (m/z): calcd for $C_{17}H_{15}NNaO$ ($[M+Na]^+$), 272.1046, found, 272.1049.

6-allyl-2-methyl-4-phenylchromane (4z)

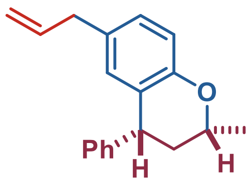

Condition B. Yellow oil, 77% yield (40.7 mg), 1.4:1 dr. **^{1}H NMR (600 MHz, $CDCl_3$)** δ 7.34 – 7.29 (m, 3.4H), 7.26 – 7.24 (m, 1.7H), 7.17 (d, *J* = 7.2 Hz, 2H), 7.08 (d, *J* = 7.8 Hz, 1.4H), 6.98 (d, *J* = 8.4 Hz, 0.7H), 6.91 (d, *J* = 7.8 Hz, 1H), 6.84 (d, *J* = 8.4 Hz, 0.7H), 6.81 – 6.75 (m, 1.7H), 6.52 (s, 1H), 5.94 – 5.86 (m, 0.7H), 5.86 – 5.78 (m, 1H), 5.03 – 4.96 (m, 1.4H), 4.96 – 4.89 (m, 2H), 4.32 – 4.22 (m, 1H), 4.20 – 4.17 (m, 0.7H), 4.17 – 4.13 (m, 1H), 4.13 – 4.06 (m, 0.7H), 3.28 – 3.21 (m, 1.4H), 3.19 – 3.09 (m, 2H), 2.22 – 2.14 (m, 1H), 2.12 – 2.04 (m, 0.7H), 1.98 – 1.94 (m, 0.7H), 1.94 – 1.85 (m, 1H), 1.41 (d, *J* = 6.6 Hz, 3H), 1.31 (d, *J* = 6.0 Hz, 2.1H). **^{13}C NMR (151 MHz, $CDCl_3$)** δ 153.8, 146.8, 145.1, 137.9, 131.6, 131.5, 130.7, 129.8, 128.7, 128.6, 128.5, 128.3, 128.2, 127.8, 126.6, 126.2, 125.4, 122.6, 116.7, 116.6, 115.3, 115.1, 72.3, 67.2, 43.1, 40.4, 40.3, 39.3, 37.9, 21.6, 21.2. **HRMS** (ESI-TOF) (m/z): calcd for $C_{19}H_{20}NaO$ ($[M+Na]^+$), 287.1406, found, 287.1408.

6-methyl-8-phenyl-7,8-dihydro-6H-[1,3]dioxolo[4,5-g]chromene (4aa)

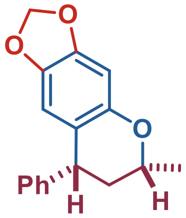

Condition B. White solid, 90% yield (48.3 mg), 1.3:1 dr. **^{1}H NMR (500 MHz, $CDCl_3$)** δ 7.44 – 7.35 (m, 7.27H), 7.34–7.28 (m, 1.80H), 6.72 (s, 1H), 6.59 (s, 0.8H), 6.45 (d, *J* = 10.0 Hz, 1.8H), 5.89 – 5.84 (m, 3.6H), 5.03 (dd, *J* = 9.0, 2.0 Hz, 0.8H), 4.97 (dd, *J* = 9.5, 1.0 Hz, 2H), 3.14 – 3.05 (m, 1H), 2.91 – 2.83 (m, 0.8H), 2.21 – 2.11 (m, 1.8H), 1.90 – 1.84 (m, 0.8H), 1.80 – 1.72 (m, 1H), 1.37 (d, *J* = 6.0 Hz, 2.4H), 1.30 (d, *J* = 5.5 Hz, 3H). **^{13}C NMR (151 MHz, $CDCl_3$)** δ 149.5, 149.0, 146.4, 146.3, 141.7, 141.64, 141.58, 141.5, 128.5, 128.4, 127.9, 127.7, 126.0, 118.8, 118.7, 107.7, 106.8, 100.8, 100.7, 98.7, 98.6, 78.1, 73.5, 40.1, 37.1, 30.1, 28.6, 24.0, 20.7. **HRMS** (ESI-TOF) (m/z): calcd for $C_{17}H_{16}NaO_3$ ($[M+Na]^+$), 291.0992, found, 291.0994.

3-methyl-1-phenyl-2,3-dihydro-1H-benzo[f]chromene (4ab)

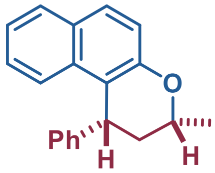

Condition B. Yellow solid, 79% yield (43.3 mg), 1.3:1 dr. **^{1}H NMR (500 MHz, $CDCl_3$)** δ 7.92 (d, *J* = 8.5 Hz, 0.84H), 7.87 (d, *J* = 8.5 Hz, 1H), 7.77 (t, *J* = 7.5 Hz, 1.8H), 7.63 (dd, *J* = 9.0, 3.5 Hz, 1.8H), 7.53 – 7.44 (m, 5.4H), 7.44 – 7.37 (m, 3.6H), 7.37 – 7.29 (m, 3.6H), 7.18 – 7.10 (m, 1.8H), 5.29 (dd, *J* = 12.0, 2.0 Hz, 0.8H), 4.96 (dd, *J* = 10.5, 2.5 Hz, 1H), 3.75 – 3.66 (m, 1H), 3.66 – 3.59 (m, 0.8H), 2.67 – 2.60 (m, 1H), 2.29 – 2.20 (m, 0.8H), 2.13 – 2.04 (m, 1.8H), 1.59 (d, *J* = 7.0 Hz, 2.4H), 1.34 (d, *J* = 7.0 Hz, 3H). **^{13}C NMR (151 MHz, $CDCl_3$)** δ 153.3, 151.7, 141.7, 141.5, 132.5, 132.4, 129.9, 129.3, 128.69, 128.67, 128.6, 128.5, 128.1, 127.9, 127.8, 127.7, 126.3, 126.2, 126.0, 125.8, 123.5, 123.1, 123.0, 122.2, 120.1, 119.5, 119.3, 118.3, 77.3, 73.0, 41.3, 37.1, 27.1, 25.9, 23.1, 22.7. **HRMS** (ESI-TOF) (m/z): calcd for $C_{20}H_{18}NaO$ ($[M+Na]^+$), 297.1250, found, 297.1254.

8-bromo-3-methyl-1-phenyl-2,3-dihydro-1H-benzo[f]chromene (4ac)

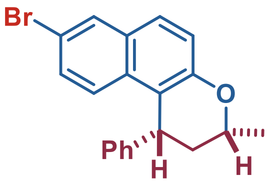

Condition B. Yellow solid, 99% yield (69.7 mg), 1:1 dr. **^{1}H NMR (500 MHz, $CDCl_3$)** δ 7.90 (dd, J = 7.5, 2.0 Hz, 2H), 7.76 (d, J = 9.0 Hz, 1H), 7.71 (d, J = 9.0 Hz, 1H), 7.55 – 7.48 (m, 5H), 7.48 – 7.44 (m, 3H), 7.44 – 7.37 (m, 4H), 7.37 – 7.30 (m, 2H), 7.18 – 7.11 (m, 2H), 5.27 (dd, J = 12.0, 2.0 Hz, 1H), 4.94 (dd, J = 10.5, 2.5 Hz, 1H), 3.68 – 3.58 (m, 1H), 3.58 – 3.51 (m, 1H), 2.66 – 2.57 (m, 1H), 2.29 – 2.18 (m, 1H), 2.10 – 2.02 (m, 2H), 1.54 (d, J = 7.0 Hz, 3H), 1.30 (d, J = 6.5 Hz, 3H). **^{13}C NMR (151 MHz, $CDCl_3$)** δ 153.6, 152.0, 141.4, 141.2, 131.1, 131.03, 130.96, 130.54, 130.52, 129.4, 128.9, 128.6, 128.5, 128.0, 127.8, 127.1, 126.9, 126.2, 125.9, 125.3, 124.0, 120.6, 120.4, 120.3, 118.5, 116.7, 116.6, 77.3, 73.1, 41.1, 36.8, 27.1, 25.9, 23.1, 22.7. **HRMS** (ESI-TOF) (m/z): calcd for $C_{20}H_{17}BrNaO$ ([M+Na]$^+$), 375.0355, found, 375.0358.

8-chloro-4-(4-fluorophenyl)-2-methylchromane (4ad)

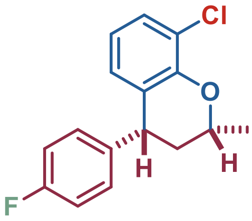

Condition B. Yellow oil, 78% yield (43.1 mg), >20:1 dr. **^{1}H NMR (600 MHz, $CDCl_3$)** δ 7.18 (d, J = 8.0 Hz, 1H), 7.14 – 7.10 (m, 2H), 7.05 – 6.98 (m, 2H), 6.66 (t, J = 7.5 Hz, 1H), 6.58 (d, J = 7.5 Hz, 1H), 4.41 – 4.31 (m, 1H), 4.21 – 4.13 (dd, 1H), 2.25 – 2.17 (m, 1H), 1.97 – 1.86 (m, 1H), 1.50 (d, J = 6.0 Hz, 3H). **^{13}C NMR (151 MHz, $CDCl_3$)** δ 161.7 (d, J = 245.2 Hz), 151.2, 140.2, 129.9 (d, J = 7.8 Hz), 128.4, 128.2, 127.3, 121.5, 120.2, 115.6 (d, J = 21.3 Hz), 73.3, 42.5, 39.8, 21.4. **^{19}F NMR (565 MHz, $CDCl_3$)** δ = -115.94 ~ -115.99 (m, 1F). **HRMS** (ESI-TOF) (m/z): calcd for $C_{16}H_{14}ClFNaO$ ([M+Na]$^+$), 299.0609, found, 299.0613.

8-chloro-4-(4-chlorophenyl)-2-methylchromane (4ae)

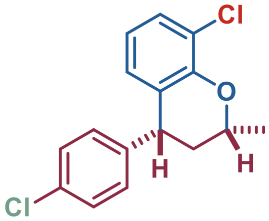

Condition B. Yellow oil, 74% yield (43.2 mg), >20:1 dr. **^{1}H NMR (500 MHz, $CDCl_3$)** δ 7.29 (d, J = 8.0 Hz, 2H), 7.19 (d, J = 7.5 Hz, 1H), 7.09 (d, J = 8.8 Hz, 2H), 6.66 (t, J = 7.5 Hz, 1H), 6.57 (d, J = 8.0 Hz, 1H), 4.39 – 4.30 (m, 1H), 4.19 – 4.13 (m, 1H), 2.23 – 2.16 (m, 1H), 1.98 – 1.84 (m, 1H), 1.50 (d, J = 6.5 Hz, 3H). **^{13}C NMR (151 MHz, $CDCl_3$)** δ 151.2, 143.0, 132.5, 129.8, 128.9, 128.4, 128.1, 126.9, 121.5, 120.2, 73.2, 42.6, 39.6, 21.4. **HRMS** (ESI-TOF) (m/z): calcd for $C_{16}H_{14}Cl_2NaO$ ([M+Na]$^+$), 315.0314, found, 315.0311.

4-(4-bromophenyl)-8-chloro-2-methylchromane (4af)

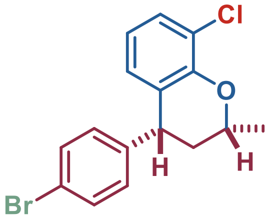

Condition B. Yellow oil, 76% yield (51.1 mg), >20:1 dr. **^{1}H NMR (500 MHz, $CDCl_3$)** δ 7.44 (d, J = 8.0 Hz, 2H), 7.18 (d, J = 8.0 Hz, 1H), 7.04 (d, J = 8.5 Hz, 2H), 6.66 (t, J = 8.0 Hz, 1H), 6.57 (d, J = 8.0 Hz, 1H), 4.38 – 4.30 (m, 1H), 4.18 – 4.11 (m, 1H), 2.24 – 2.16 (m, 1H), 1.94 – 1.84 (m, 1H), 1.50 (d, J = 6.0 Hz, 3H). **^{13}C NMR (151 MHz, $CDCl_3$)** δ 151.2, 143.5, 131.8, 130.2, 128.4, 128.1, 126.8, 121.6, 120.6, 120.2, 73.2, 42.7, 39.6, 21.4. **HRMS** (ESI-TOF) (m/z): calcd for $C_{16}H_{14}BrClNaO$ ([M+Na]$^+$), 358.9809, found, 358.9811.

8-chloro-2-methyl-4-(p-tolyl)chromane (4ag)

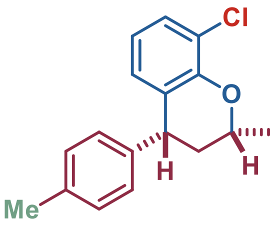

Condition B. Yellow oil, 60% yield (32.7 mg), >20:1 dr. **^{1}H NMR (500 MHz, $CDCl_3$)** δ 7.16 (d, *J* = 7.5 Hz, 1H), 7.13 (d, *J* = 7.5 Hz, 2H), 7.05 (d, *J* = 8.0 Hz, 2H), 6.66 – 6.58 (m, 2H), 4.39 – 4.31 (m, 1H), 4.17 – 4.09 (m, 1H), 2.34 (s, 3H), 2.21 – 2.16 (m, 1H), 1.99 – 1.89 (m, 1H), 1.49 (d, *J* = 6.5 Hz, 3H). **^{13}C NMR (151 MHz, $CDCl_3$)** δ 151.2, δ 141.4, 136.4, 129.4, 128.33, 128.27, 128.1, 127.7, 121.3, 120.1, 73.3, 42.8, 39.7, 21.4, 21.0. **HRMS** (ESI-TOF) (m/z): calcd for $C_{17}H_{17}ClNaO$ ($[M+Na]^+$), 295.0860, found, 295.0858.

8-chloro-4-(4-methoxyphenyl)-2-methylchromane (4ah)

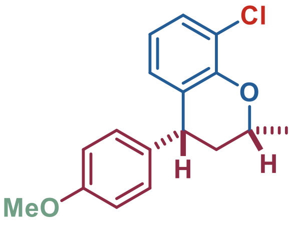

Condition B. Yellow oil, 42% yield (24.2 mg), >20:1 dr. **^{1}H NMR (500 MHz, $CDCl_3$)** δ 7.17 (d, *J* = 7.5 Hz, 1H), 7.08 (d, *J* = 8.5 Hz, 2H), 6.86 (d, *J* = 9.0 Hz, 2H), 6.68 – 6.59 (m, 2H), 4.40 – 4.31 (m, 1H), 4.16 – 4.09 (m, 1H), 3.80 (s, 3H), 2.22 – 2.14 (m, 1H), 1.98 – 1.88 (m, 1H), 1.50 (d, *J* = 6.0 Hz, 3H). **^{13}C NMR (151 MHz, $CDCl_3$)** δ 158.4, 151.1, 136.4, 129.4, 128.2, 128.1, 127.9, 121.3, 120.1, 114.1, 73.4, 55.3, 42.4, 39.7, 21.4. **HRMS** (ESI-TOF) (m/z): calcd for $C_{17}H_{17}ClNaO_2$ ($[M+Na]^+$), 311.0809, found, 311.0814.

4-(4-(tert-butyl)phenyl)-8-chloro-2-methylchromane (4ai)

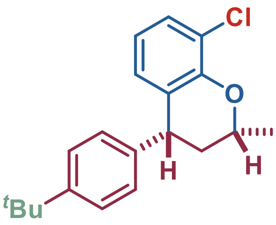

Condition B. Yellow oil, 50% yield (31.4 mg), >20:1 dr. **^{1}H NMR (500 MHz, $CDCl_3$)** δ 7.33 (d, *J* = 8.0 Hz, 2H), 7.17 (d, *J* = 7 Hz, 1H), 7.08 (d, *J* = 8.0 Hz, 2H), 6.69 – 6.61 (m, 2H), 4.40 – 4.30 (m, 1H), 4.18 – 4.10 (m, 1H), 2.24 – 2.16 (m, 1H), 2.00 – 1.90 (m, 1H), 1.49 (d, *J* = 6.5 Hz, 3H), 1.32 (s, 9H). **^{13}C NMR (151 MHz, $CDCl_3$)** δ 149.6, 141.2, 128.4, 128.1, 128.0, 127.7, 125.5, 121.3, 120.1, 73.4, 42.7, 39.7, 34.4, 31.4, 21.4. **HRMS** (ESI-TOF) (m/z): calcd for $C_{20}H_{23}ClNaO$ ($[M+Na]^+$), 337.1330, found, 337.1333.

8-chloro-2-methyl-4-(m-tolyl)chromane (4aj)

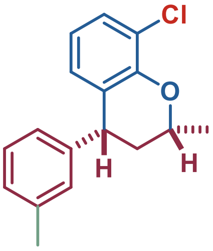

Condition B. Yellow oil, 80% yield (43.5 mg), >20:1 dr. **^{1}H NMR (500 MHz, $CDCl_3$)** δ 7.23 – 7.15 (m, 2H), 7.07 (d, *J* = 7.5 Hz, 1H), 6.96 (d, *J* = 10.5 Hz, 2H), 6.68 – 6.59 (m, 2H), 4.40 – 4.30 (m, 1H), 4.16 – 4.11 (m, 1H), 2.32 (s, 3H), 2.24 – 2.17 (m, 1H), 2.01 – 1.91 (m, 1H), 1.50 (d, *J* = 6.5 Hz, 3H). **^{13}C NMR (151 MHz, $CDCl_3$)** δ 151.2, 144.4, 138.3, 129.2, 128.5, 128.3, 128.1, 127.6, 127.6, 125.6, 121.3, 120.1, 73.3, 43.1, 39.7, 21.4. **HRMS** (ESI-TOF) (m/z): calcd for $C_{17}H_{17}ClNaO$ ($[M+Na]^+$), 295.0860, found, 295.0862.

4-(3-(tert-butyl)phenyl)-8-chloro-2-methylchromane (4ak)

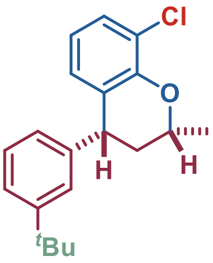

Condition B. Yellow oil, 70% yield (44.0 mg), >20:1 dr. **^{1}H NMR (600 MHz, CDCl$_3$)** δ 7.28 (d, *J* = 8.4 Hz, 1H), 7.24 – 7.22 (m, 1H), 7.19 (s, 1H), 7.16 (d, *J* = 7.8 Hz, 1H), 6.96 – 6.93 (m, 1H), 6.66 – 6.59 (m, 2H), 4.39 – 4.33 (m, 1H), 4.19 – 4.14 (m, 1H), 2.24 – 2.19 (m, 1H), 2.01 – 1.92 (m, 1H), 1.50 (d, *J* = 6.0 Hz, 3H), 1.30 (s, 9H). **^{13}C NMR (151 MHz, CDCl$_3$)** δ 151.6, 151.2, 144.0, 128.33, 128.29 , 128.1, 127.7, 125.7, 125.4, 123.7, 121.3, 120.0, 73.4, 43.5, 39.7, 34.7, 31.4, 21.4. **HRMS** (ESI-TOF) (m/z): calcd for $C_{20}H_{23}ClNaO$ ([M+Na]$^+$), 337.1330, found, 337.1332.

8-chloro-4-(3-methoxyphenyl)-2-methylchromane (4al)

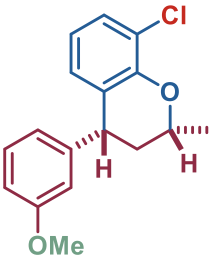

Condition B. Yellow oil, 68% yield (39.2 mg), >20:1 dr. **^{1}H NMR (600 MHz, CDCl$_3$)** δ 7.16 (t, *J* = 9.6 Hz, 1H), 7.10 (d, *J* = 7.2 Hz, 1H), 6.72 (d, *J* = 7.8 Hz, 1H), 6.68 (d, *J* = 7.8 Hz, 1H), 6.62 (s, 1H), 6.59 (d, *J* = 7.8 Hz, 1H), 6.57 (d, *J* = 7.8 Hz, 1H), 4.30 – 4.24 (m, 1H), 4.09 – 4.05 (m, 1H), 3.69 (s, 3H), 2.17 – 2.12 (m, 1H), 1.92 – 1.85 (m, 1H), 1.43 (d, *J* = 6.6 Hz, 3H). **^{13}C NMR (151 MHz, CDCl$_3$)** δ 159.9, 151.2, 146.0, 129.7, 128.3, 128.2, 127.3, 121.4, 120.9, 120.2, 114.2, 112.1, 73.3, 55.2, 43.2, 39.5, 21.4. **HRMS** (ESI-TOF) (m/z): calcd for $C_{17}H_{17}ClNaO_2$ ([M+Na]+), 311.0809, found, 311.0805.

6-bromo-4-(3-methoxyphenyl)-2-methylchromane (4am)

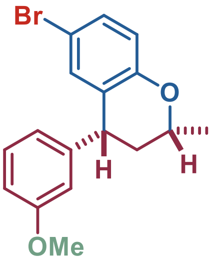

Condition B. Yellow oil, 73% yield (48.5 mg), >20:1 dr. **^{1}H NMR (600 MHz, CDCl$_3$)** δ 7.17 (t, *J* = 7.8 Hz, 1H), 7.09 (d, *J* = 8.4 Hz, 1H), 6.76 (s, 1H), 6.73 (d, *J* = 8.4 Hz, 1H), 6.67 (d, *J* = 7.2 Hz, 1H), 6.64 (s, 1H), 6.62 (d, *J* = 4.8 Hz, 1H), 4.21 – 4.13 (m, 1H), 4.03 – 3.90 (m 1H), 3.71 (s, 3H), 2.12 – 2.05 (m, 1H), 1.86 – 1.77 (m, 1H), 1.33 (d, *J* = 6.0 Hz, 3H). **^{13}C NMR (151 MHz, CDCl$_3$)** δ 159.9, 154.5, 145.6, 132.2, 130.5, 129.8, 127.7, 120.8, 118.5, 114.3, 112.3, 112.1, 72.6, 55.2, 43.0, 39.4, 21.4. **HRMS** (ESI-TOF) (m/z): calcd for $C_{17}H_{17}BrNaO_2$ ([M+Na]$^+$), 355.0304, found, 355.0309.

6-(tert-butyl)-4-(4-chlorophenyl)-2-methylchromane (4an)

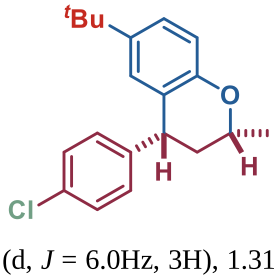

Condition B. Yellow oil, 91% yield (57.2 mg), 2:1 dr. **^{1}H NMR (600 MHz, CDCl$_3$)** δ 7.28 (d, *J* = 6.6 Hz, 2H), 7.26 – 7.24 (m, 2H), 7.12 (d, *J* = 9.0 Hz, 2.5H), 7.01 (d, *J* = 8.4 Hz, 1H), 6.91 (s, 0.5H), 6.84 (d, *J* = 8.4 Hz, 0.5H), 6.78 (d, *J* = 9.0 Hz, 1H), 6.69 (s, 1H), 4.30 – 4.22 (m, 1H), 4.20 – 4.12 (m, 1.5H), 4.06 – 3.99 (m, 0.5H), 2.18 – 2.13 (m, 1H), 2.12 – 2.05 (m, 0.5H), 1.94 – 1.90 (m, 0.5H), 1.87 – 1.79 (m, 1H), 1.40 (d, *J* = 6.0Hz, 3H), 1.31 (d, *J* = 6.0 Hz, 1.5H), 1.22 (s, 4.5H), 1.14 (s, 9H). **^{13}C NMR (151 MHz, CDCl$_3$)** δ 153.22, 153.17, 145.4, 143.8, 142.9, 132.2, 131.9, 130.99, 129.94, 129.8, 128.7, 128.4, 127.3, 126.4, 126.1, 125.3, 124.8, 124.0, 116.3, 116.1, 72.1, 67.1, 42.7, 40.6, 40.0, 38.0, 34.0, 31.49, 31.45, 31.4, 21.6, 21.2. **HRMS** (ESI-TOF) (m/z): calcd for $C_{20}H_{23}ClNaO$ ([M+Na]$^+$), 337.1330, found, 337.1336.

4-([1,1'-biphenyl]-4-yl)-8-chloro-2-methylchromane (4ao)

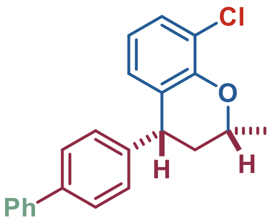

Condition B. White solid, 53% yield (35.4 mg), >20:1 dr. **^{1}H NMR (500 MHz, $CDCl_3$)** δ 7.58 (d, *J* = 7.0 Hz, 2H), 7.55 (d, *J* = 8.0 Hz, 2H), 7.43 (t, *J* = 7.5 Hz, 2H), 7.34 (d, *J* = 7.0 Hz, 1H), 7.23 (d, *J* = 8.5 Hz, 2H), 7.20 – 7.17 (m, 1H), 6.67 (d, *J* = 5.0 Hz, 2H), 4.42 – 4.32 (m, 1H), 4.26 – 4.18 (m, 1H), 2.28 –2.21 (m, 1H), 2.04 – 1.94 (m, 1H), 1.51 (d, *J* = 6.5 Hz, 3H). **^{13}C NMR (151 MHz, $CDCl_3$)** δ 151.2, 143.5, 140.7, 139.8, 128.9, 128.8, 128.33, 128.26, 127.4, 127.2, 127.0, 121.5, 120.2, 73.3, 42.9, 39.7, 21.4. **HRMS** (ESI-TOF) (m/z): calcd for $C_{22}H_{19}ClNaO$ ($[M+Na]^+$), 357.1017, found, 357.1019.

4-([1,1'-biphenyl]-3-yl)-8-chloro-2-methylchromane (4ap)

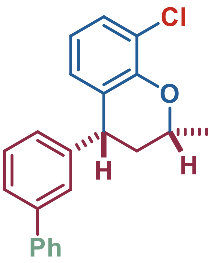

Condition B. White solid, 71% yield (47.4 mg), >20:1 dr. **^{1}H NMR (600 MHz, $CDCl_3$)** δ 7.56 (d, *J* = 7.8 Hz, 2H), 7.49 (d, *J* = 6.0 Hz, 1H), 7.42 (t, *J* = 7.8 Hz, 2H), 7.38 (d, *J* = 7.8 Hz, 2H), 7.34 (d, *J* = 7.2 Hz, 1H), 7.19 (dd, *J* = 6.0, 3.0 Hz, 1H), 7.14 (d, *J* = 7.2 Hz, 1H), 6.67 (d, *J* = 6.5 Hz, 2H), 4.42 – 4.35 (m, 1H), 4.27 – 4.22 (m, 1H), 2.30 – 2.24 (m, 1H), 2.06 – 1.98 (m, 1H), 1.52 (d, *J* = 6.2 Hz, 3H). **^{13}C NMR (151 MHz, $CDCl_3$)** δ 151.2, 145.0, 141.7, 140.9, 129.1, 128.8, 128.33, 128.27, 127.40, 127.37, 127.3, 127.1, 125.7, 121.5, 120.2, 73.3, 43.3, 39.8, 21.4. **HRMS** (ESI-TOF) (m/z): calcd for $C_{22}H_{19}ClNaO$ ($[M+Na]^+$), 357.1017, found, 357.1021.

8-chloro-2-methyl-4-(4-(trifluoromethyl)phenyl)chromane (4aq)

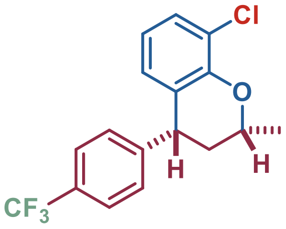

Condition B. Yellow oil, 73% yield (47.6 mg), >20:1 dr. **^{1}H NMR (600 MHz, $CDCl_3$)** δ 7.58 (d, *J* = 8.4 Hz, 2H), 7.28 (d, *J* = 8.4 Hz, 2H), 7.21 (d, *J* = 8.4 Hz, 1H), 6.67 (t, *J* = 7.8 Hz, 1H), 6.55 (d, *J* = 7.8 Hz, 1H), 4.39 – 4.32 (m, 1H), 4.30 – 4.22 (m, 1H), 2.26 – 2.20 (m, 1H), 1.98 – 1.88 (m, 1H), 1.51 (d, *J* = 6.0 Hz, 3H). **^{13}C NMR (151 MHz, $CDCl_3$)** δ 151.3, 148.7, 129.2 (q, *J* = 32.8 Hz), 128.8, 128.6, 128.1, 126.4, 125.7 (q, *J* = 3.5 Hz), 124.1 (d, *J* = 272.4 Hz), 121.7, 120.3, 73.1, 43.1, 39.7, 21.3. **^{19}F NMR (565 MHz, $CDCl_3$)** *δ* = -62.43 (s, $1CF_3$). **HRMS** (ESI-TOF) (m/z): calcd for $C_{17}H_{14}ClF_3NaO$ ($[M+Na]^+$), 349.0577, found, 349.0581.

2-(4-(8-chloro-2-methylchroman-4-yl)phenyl)-4,4,5,5-tetramethyl-1,3,2-dioxaborolane (4ar)

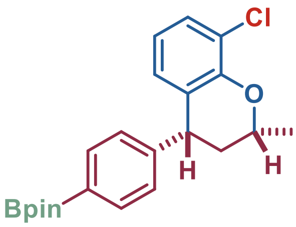

Condition B. White solid, 75% yield (57.6 mg), >20:1 dr. **^{1}H NMR (600 MHz, $CDCl_3$)** δ 7.77 (d, *J* = 7.2 Hz, 2H), 7.18 (d, *J* = 7.8 Hz, 3H), 6.63 (t, *J* = 7.8 Hz, 1H), 6.57 (d, *J* = 7.8 Hz, 1H), 4.38 – 4.32 (m, 1H), 4.19 (dd, *J* = 12.0, 6.0 Hz, 1H), 2.21 (dd, *J* = 14.4, 6.0 Hz, 1H), 2.01 – 1.89 (m, 1H), 1.50 (d, *J* = 6.0 Hz, 3H), 1.34 (s, 12H). **^{13}C NMR (151 MHz, $CDCl_3$)** δ 151.2, 147.7, 135.2, 128.32, 128.26, 128.0, 127.3, 121.5, 120.1, 83.8, 73.3, 43.4, 39.5,24.9, 24.8, 21.4. ^{11}B NMR (193 MHz, $CDCl_3$) δ 30.62. **HRMS** (ESI-TOF) (m/z): calcd for $C_{22}H_{26}BClNaO_3$ ($[M+Na]^+$), 407.1556, found, 407.1559.

8-chloro-4-(3,5-dimethylphenyl)-2-methylchromane (4as)

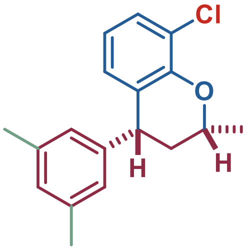

Condition B. Yellow oil, 56% yield (32.0 mg), >20:1 dr. **^{1}H NMR (600 MHz, $CDCl_3$)** δ 7.17 (d, *J* = 6.6 Hz, 1H), 6.89 (s, 1H), 6.77 (s, 2H), 6.67 – 6.62 (m, 2H), 4.38 – 4.30 (m, 1H), 4.11 – 4.03 (m, 1H), 2.28 (s, 6H), 2.21 – 2.16 (m, 1H), 2.00 – 1.90 (m, 1H), 1.49 (d, *J* = 6.6 Hz, 3H). **^{13}C NMR (151 MHz, $CDCl_3$)** δ 151.2, 144.3, 138.2, 128.5, 128.4, 128.1, 127.8, 126.3, 121.3, 120.1, 73.4, 43.1, 39.7, 21.4, 21.3. **HRMS** (ESI-TOF) (m/z): calcd for $C_{18}H_{19}ClNaO$ ($[M+Na]^+$), 309.1017, found, 309.1021 .

4-(4-bromo-3-methylphenyl)-8-chloro-2-methylchromane (4at)

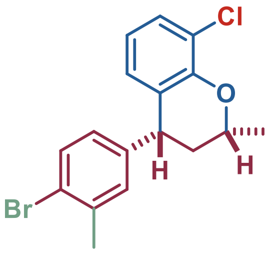

Condition B. Yellow oil, 64% yield (44.8 mg), >20:1 dr. **^{1}H NMR (600 MHz, $CDCl_3$)** δ 7.45 (d, *J* = 8.4 Hz, 1H), 7.17 (d, *J* = 7.8 Hz, 1H), 7.02 (s, 1H), 6.83 (d, *J* = 7.8 Hz, 1H), 6.65 (t, *J* = 7.8 Hz, 1H), 6.58 (d, *J* = 7.8 Hz, 1H), 4.36 – 4.27 (m, 1H), 4.14 – 4.06 (m, 1H), 2.35 (s, 3H), 2.18 – 2.14 (m, 1H), 1.93 – 1.85 (m, 1H), 1.48 (d, *J* = 6.0 Hz, 3H). **^{13}C NMR (151 MHz, $CDCl_3$)** δ 151.2, 143.7, 138.1, 132.6, 130.9, 128.3, 128.2, 127.4, 127.0, 123.0, 121.5, 120.2, 73.2, 42.6, 39.6, 22.9, 21.4. **HRMS** (ESI-TOF) (m/z): calcd for $C_{17}H_{16}BrClNaO$ ($[M+Na]^+$), 372.9965, found, 372.9971.

8-chloro-2-ethyl-4-phenylchromane (4au)

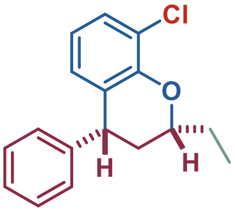

Condition B. Yellow oil, 99% yield (53.9 mg), >20:1 dr. **^{1}H NMR (600 MHz, $CDCl_3$)** δ 7.32 (t, *J* = 7.8 Hz, 2H), 7.25 (t, *J* = 6.8 Hz, 1H), 7.16 (d, *J* = 7.2 Hz, 3H), 6.64 (t, *J* = 7.2 Hz, 1H), 6.60 (d, *J* = 7.8 Hz, 1H), 4.19 – 4.10 (m, 2H), 2.27 – 2.20 (m, 1H), 1.98 – 1.90 (m, 1H), 1.90 – 1.84 (m, 1H), 1.78 – 1.70 (m, 1H), 1.11 (t, *J* = 7.8 Hz, 3H). **^{13}C NMR (151 MHz, $CDCl_3$)** δ 151.2, 144.5, 128.7, 128.5, 128.20, 128.16 , 127.8, 126.8, 121.6, 120.0, 78.2, 43.2, 37.6, 28.6, 9.6. **HRMS** (ESI-TOF) (m/z): calcd for $C_{17}H_{17}ClNaO$ ($[M+Na]^+$), 295.0860, found, 295.0864.

4-(benzofuran-5-yl)-8-chloro-2-methylchromane (4av)

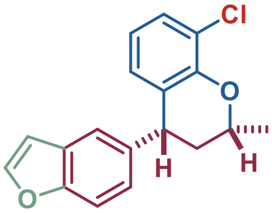

Condition B. Yellow oil, 42% yield (25.0 mg), >20:1 dr. **^{1}H NMR (600 MHz, $CDCl_3$)** δ 7.56 (d, *J* = 7.8 Hz, 2H), 7.49 (d, *J* = 7.8 Hz, 1H), 7.42 (t, *J* = 7.2 Hz, 1H), 7.34 (d, *J* = 7.2 Hz, 1H), 7.20 – 7.16 (m, 1H), 7.14 (d, *J* = 7.8 Hz, 1H), 6.69 – 6.50 (m, 1H), 4.42 – 4.34 (m, 1H), 4.28 – 4.22 (m, 1H), 2.30 – 2.24 (m, 1H), 2.06 – 1.98 (m, 1H), 1.51 (d, *J* = 6.0 Hz, 3H). **^{13}C NMR (151 MHz, $CDCl_3$)** δ 151.3, 145.0, 141.7, 141.0, 129.2, 128.8, 128.4, 128.3, 127.4, 127.3, 127.2, 125.7, 121.5, 120.2, 73.4, 43.4, 39.9, 21.5. **HRMS** (ESI-TOF) (m/z): calcd for $C_{18}H_{15}ClNaO_2$ ($[M+Na]^+$), 321.0653, found, 321.0654.

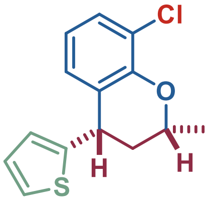

8-chloro-2-methyl-4-(thiophen-2-yl)chromane (4aw)

Condition B. Yellow oil, 54% yield (28.5 mg), >20:1 dr. **^{1}H NMR (600 MHz, $CDCl_3$)** δ 7.23 – 7.17 (m, 2H), 6.99 – 6.93 (m, 2H), 6.81 (d, *J* = 7.8 Hz, 1H), 6.69 (t, *J* = 7.8 Hz, 1H), 4.53 (dd, *J* = 12.0, 6.0 Hz, 1H), 4.39 – 4.33 (m, 1H), 2.37 – 2.31 (m, 1H), 2.09 – 2.01 (m, 1H), 1.51 (d, *J* = 6.6 Hz, 3H).

^{13}C NMR (151 MHz, CDCl$_3$) δ 150.5, 147.2, 128.6, 127.8, 126.9, 126.6, 125.7, 124.2, 121.4, 120.2, 73.4, 40.2, 38.1, 21.3. **HRMS** (ESI-TOF) (m/z): calcd for $C_{14}H_{13}ClNaOS$ ([M+Na]$^+$), 287.0268, found, 287.0266.

4-(2-methyl-4-phenylchroman-6-yl)butan-2-one (5a)

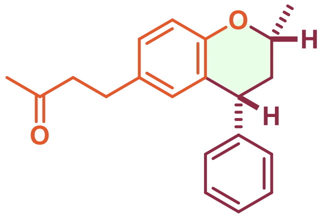

Condition B. Yellow oil, 91% yield (53.5 mg), 2.5:1 dr. **^{1}H NMR (600 MHz, CDCl$_3$)** δ 7.32 (t, *J* = 7.2 Hz, 2H), 7.29 – 7.23 (m, 1.84H), 7.20 (t, *J* = 7.8 Hz, 0.42H), 7.16 (d, *J* = 7.2 Hz, 2H), 7.07 (d, *J* = 7.8 Hz, 0.84H), 6.97 (d, *J* = 7.8 Hz, 0.42H), 6.90 (d, *J* = 8.4 Hz, 1H), 6.82 (d, *J* = 8.4 Hz, 0.42H), 6.78 – 6.74 (m, 1.42H), 6.50 (s, 1H), 4.29 – 4.22 (m, 1H), 4.19 – 4.16 (m, 0.42H), 4.15 – 4.11 (m, 1H), 4.11 – 4.06 (m, 0.42H), 2.74 (t, *J* = 7.2 Hz, 1H), 2.70 – 2.62 (m, 2.88H), 2.60 – 2.53 (m, 1.84H), 2.20 – 2.14 (m, 1H), 2.11 – 2.05 (m, 1.68H), 2.04 (s, 3H), 1.99 – 1.94 (m, 0.42H), 1.95 – 1.85 (m, 1H), 1.40 (d, *J* = 6.0 Hz, 3H), 1.30 (d, *J* = 6.6 Hz, 1.26H). **^{13}C NMR (151 MHz, CDCl$_3$)** δ 208.1, 153.8, 146.7, 144.9, 132.45, 132.42, 130.3, 129.3, 128.59, 128.57, 128.5, 128.3, 127.9, 127.4, 126.6, 126.2, 125.5, 122.7, 116.8, 116.6, 72.2, 67.3, 45.4, 43.0, 40.24, 40.18, 37.8, 30.03, 29.96, 29.0, 28.9, 21.6, 21.1. **HRMS** (ESI-TOF) (m/z): calcd for $C_{20}H_{22}NaO_2$ ([M+Na]$^+$), 317.1512, found, 317.1516.

4,8-dimethyl-6-phenyl-7,8-dihydro-2H,6H-pyrano[3,2-g]chromen-2-one (5b)

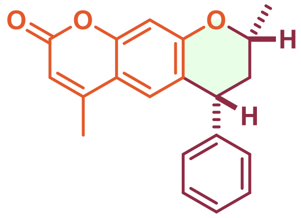

Condition B. Yellow oil, 40% yield (24.5 mg), 5:1 dr. **^{1}H NMR (600 MHz, CDCl$_3$)** δ 7.40 (d, *J* = 9.0 Hz, 0.2H), 7.35 (t, *J* = 7.2 Hz, 2H), 7.30 – 7.27 (m, 1.2H), 7.25 – 7.22 (m, 0.8H), 7.19 (d, *J* = 7.2 Hz, 2H), 7.13 (d, *J* = 7.2 Hz, 1H), 7.06 (d, *J* = 7.2 Hz, 0.2H), 6.91 – 6.87 (m, 1.2H), 6.79 (s, 1H), 6.03 (s, 1H), 5.95 (s, 0.2H), 4.44 – 4.35 (m, 1.2H), 4.22 – 4.14 (m, 1.2H), 2.47 – 2.43 (m, 0.2H), 2.33 (s, 0.6H), 2.28 – 2.20 (m, 1H), 2.10 (s, 3H), 2.00 – 1.91 (m, 1H), 1.90 – 1.86 (m, 0.2H), 1.46 (d, *J* = 6.0 Hz, 3H), 1.42 (d, *J* = 6.6 Hz, 0.6H). **^{13}C NMR (151 MHz, CDCl$_3$)** δ 161.4, 158.7, 153.5, 152.4, 144.0 , 128.9, 128.5, 128.4, 127.1, 126.9, 126.2, 125.7, 123.5, 123.0, 113.8, 113.6, 111.9, 111.5, 104.1, 73.4, 73.0, 42.7, 41.4, 39.4, 38.4, 21.4, 21.0, 18.7, 18.5. **HRMS** (ESI-TOF) (m/z): calcd for $C_{20}H_{18}NaO_3$ ([M+Na]$^+$), 329.1148, found, 329.1152.

8-chloro-4-(4-(((2-isopropyl-5-methylcyclohexyl)oxy)methyl)phenyl)-2-methylchromane (5c)

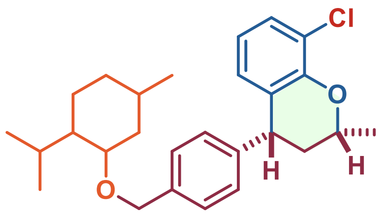

Condition B. Yellow oil, 66% yield (56.3 mg), >20:1 dr. **^{1}H NMR (600 MHz, CDCl$_3$)** δ 7.30 (d, *J* = 7.8 Hz, 2H), 7.17 (d, *J* = 7.8 Hz, 1H), 7.13 (d, *J* = 8.4 Hz, 2H), 6.67 – 6.57 (m, 2H), 4.64 (d, *J* = 10.8 Hz, 1H), 4.40 – 4.32 (m, 2H), 4.19 – 4.12 (m, 1H), 3.25 – 3.10 (m, 1H), 2.35 – 2.25 (m, 1H), 2.22 – 2.15 (m, 2H), 1.98 – 1.89 (m, 1H), 1.70 – 1.60 (m, 2H), 1.49 (d, *J* = 6.6 Hz, 3H), 1.43 – 1.33 (m, 2H), 1.32 – 1.26 (m, 1H), 0.99 – 0.96 (m, 1H), 0.94 (d, *J* = 6.6 Hz, 3H), 0.89 (d, *J* = 7.2 Hz, 3H), 0.88 – 0.85 (m, 1H), 0.71 (t, *J* = 6.6 Hz, 3H). **^{13}C NMR (151 MHz, CDCl$_3$)** δ 151.2, 143.7, 137.8, 128.5, 128.39, 128.38, 128.2, 127.6, 121.4, 120.1, 78.9, 73.3, 70.2, 48.3, 43.0, 40.4, 39.7, 34.6, 31.6, 25.5, 23.3, 22.4, 21.4, 21.0, 16.1. **HRMS** (ESI-TOF) (m/z): calcd for $C_{27}H_{35}ClNaO_2$ ([M+Na]$^+$), 449.2218, found, 449.2224.

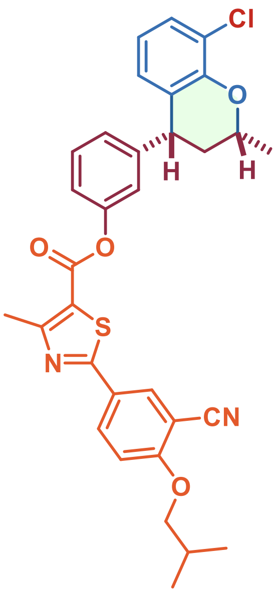

3-(8-chloro-2-methylchroman-4-yl)phenyl 2-(3-cyano-4-isobutoxyphenyl)-4-methylthiazole-5-carboxylate (5d)

Condition B. White solid, 45% yield (51.5 mg), >20:1 dr. **^{1}H NMR (600 MHz, CDCl$_3$)** δ 8.21 (d, *J* = 2.4 Hz, 1H), 8.12 (dd, *J* = 9.0, 2.4 Hz, 1H), 7.39 (t, *J* = 7.8 Hz, 1H), 7.19 (d, *J* = 7.8 Hz, 1H), 7.14 – 7.09 (m, 2H), 7.03 (d, *J* = 9.0 Hz, 2H), 6.72 – 6.64 (m, 2H), 4.40 – 4.32 (m, 1H), 4.26 – 4.20 (m, 1H), 3.91 (d, *J* = 6.6 Hz, 2H), 2.82 (s, 3H), 2.30 – 2.25 (m, 1H), 2.24 – 2.18 (m, 1H), 2.02 – 1.93 (m, 1H), 1.51 (d, *J* = 6.6 Hz, 3H), 1.10 (d, *J* = 6.6s Hz, 6H). **^{13}C NMR (151 MHz, CDCl$_3$)** δ 168.2, 163.1, 162.7, 160.3, 151.2, 150.5, 146.4, 132.7, 132.2, 129.8, 128.4, 128.2, 126.8, 126.3, 125.8, 121.6, 120.5, 120.3, 120.1, 115.3, 112.7, 103.1, 75.8, 73.2, 43.0, 39.6, 28.1, 21.4, 19.0, 17.7. **HRMS** (ESI-TOF) (m/z): calcd for $C_{32}H_{29}ClN_2NaO_4S$ ([M+Na]$^+$), 595.1429, found, 595.1433.

8-chloro-2-methylchroman-4-yl)phenyl 2-(4-isobutylphenyl)propanoate (5e)

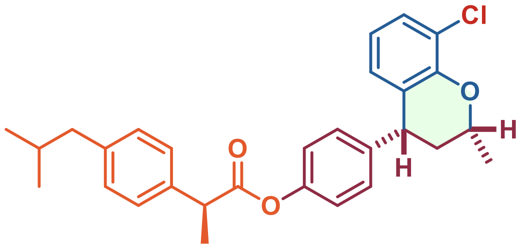

Condition B. White solid, 82% yield (75.8 mg), >20:1 dr. **^{1}H NMR (600 MHz, CDCl$_3$)** δ 7.29 (d, *J* = 7.8 Hz, 2H), 7.16 (d, *J* = 7.8 Hz, 1H), 7.15 – 7.10 (m, 4H), 6.95 (d, *J* = 8.4 Hz, 2H), 6.63 (t, *J* = 7.8 Hz, 1H), 6.57 (d, *J* = 7.2 Hz, 1H), 4.36 – 4.30 (m, 1H), 4.18 – 4.13 (m, 1H), 3.95 – 3.91 (m, 1H), 2.46 (d, *J* = 7.2 Hz, 2H), 2.22 – 2.14 (m, 1H), 1.93 – 1.83 (m, 2H), 1.60 (d, *J* = 7.2 Hz, 3H), 1.48 (d, *J* = 6.6 Hz, 3H), 0.90 (d, *J* = 6.6 Hz, 6H). **^{13}C NMR (151 MHz, CDCl$_3$)** δ 173.3, 151.2, 149.6, 141.8, 140.8, 137.2, 129.5, 129.3, 128.29, 128.25, 127.22, 127.18, 121.6, 120.2, 73.3, 45.2, 45.0, 42.6, 39.7, 30.2, 22.4, 21.4, 18.5. **HRMS** (ESI-TOF) (m/z): calcd for $C_{29}H_{31}ClNaO_3$ ([M+Na]$^+$), 485.1859, found, 485.1861.

4-(8-chloro-2-methylchroman-4-yl)phenyl 2-(7-methoxynaphthalen-2-yl)propanoate (5f)

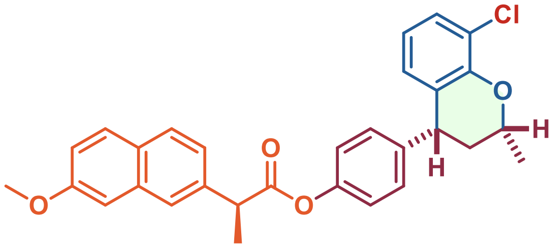

Condition B. White solid, 60% yield (58.3 mg), >20:1 dr. **^{1}H NMR (600 MHz, CDCl$_3$)** δ 7.76 – 7.72 (m, 3H), 7.51 – 7.48 (m, 1H), 7.17 – 7.13 (m, 3H), 7.10 (d, *J* = 9.0 Hz, 2H), 6.94 (d, *J* = 8.4 Hz, 2H), 6.65 – 6.60 (m, 1H), 6.56 (d, *J* = 7.8 Hz, 1H), 4.35 – 4.29 (m, 1H), 4.17 – 4.13 (m, 1H), 4.12 – 4.06 (m, 1H), 3.92 (s, 3H), 2.20 – 2.14 (m, 1H), 1.92 – 1.85 (m, 1H), 1.69 (d, *J* = 7.2 Hz, 3H), 1.48 (d, *J* = 6.0 Hz, 3H). **^{13}C NMR (151 MHz, CDCl$_3$)** δ 173.2, 157.8, 151.2, 149.6, 141.9, 135.1, 133.8, 129.3, 129.0, 128.30, 128.25, 127.4, 127.2, 126.13, 126.08, 121.6, 121.4, 120.2, 119.1, 105.6, 73.3, 55.3, 45.6, 42.6, 39.7, 21.4, 18.5. **HRMS** (ESI-TOF) (m/z): calcd for $C_{30}H_{27}ClNaO_4$ ([M+Na]$^+$), 509.1490, found, 509.1494.

VI. Structure Analysis X-Ray Crystallography of 4ao

For the stereochemistry configuration of the dihydrofunctionalization compounds, the X-ray crystallography of **4ao** at low temperature showed that the structure of compound, X-ray quality crystals were grown from PE/hexane. Crystallographic data (excluding structure factors) for the structure has been deposited with the Cambridge Crystallographic Data Center as supplementary publication number **(CCDC 2351059)**. These data can be obtained free of charge from the Cambridge Crystallographic Data Centre via www.ccdc.cam.ac.uk/data_request/cif

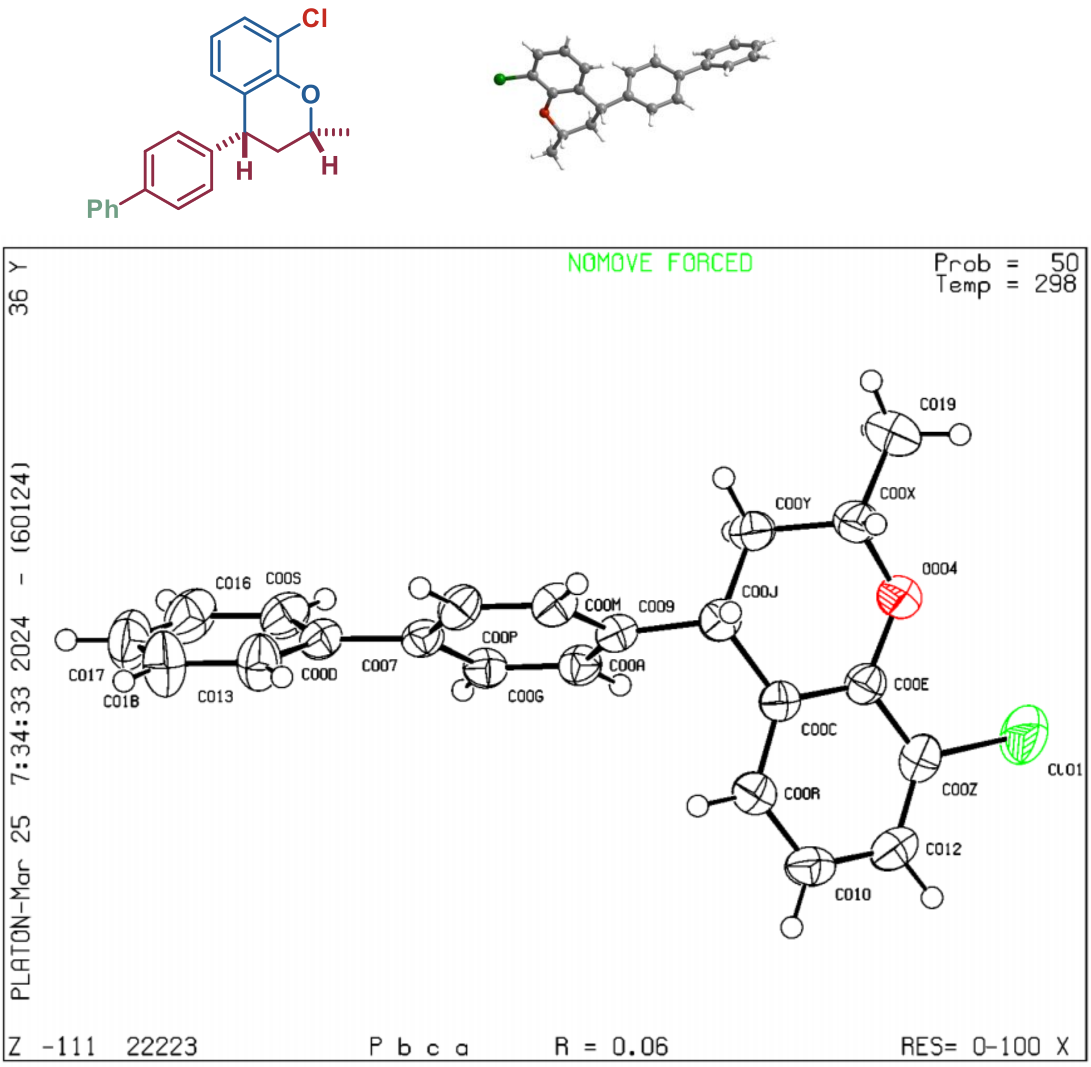

Fig S7 X-Ray Crystallography of compound **4ao.**

Table S6. Crystallographic data for compound 4ao.

Bond precision:	C-C = 0.0024 A	Wavelength=1.54178	
Cell:	a=11.8872(5) alpha=90	b=10.1609(5) beta=90	c=28.5346(12) gamma=90
Temperature:	298 K		
	Calculated	Reported	
Volume	3446.5(3)	3446.5(3)	
Space group	P b c a	P b c a	
Hall group	-P 2ac 2ab	-P 2ac 2ab	
Moiety formula	C22 H19 Cl O	C22 H19 Cl O	
Mr	334.82	334.82	
Dx,g cm-3	1.291	1.291	
Z	8	8	
Mu (mm-1)	1.981	1.981	
F000	1408.0	1408.0	
F000’	1414.36		
h,k,lmax	14,12,34	14,12,34	
Nref	3169	3159	
Tmin,Tmax	0.788,0.820	0.520,0.753	
Correction method= # Reported T Limits: Tmin=0.520 Tmax=0.753 AbsCorr = NONE			
Data completeness= 0.997	Theta(max)= 68.358		
R(reflections)= 0.0608(2818)	wR2(reflections)=0.1663(3159)		
S = 1.075	Npar= 218		

VII. Mechanistic Experiments

1. Intermediate conversion experiment

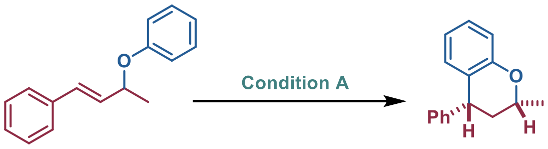

(*E*)-**3a** → **4a**, dr = 3.3:1

standard condition	92%
w/o Co	0%
w/o Ir(ppy)$_3$	0%
under dark	0%
w/o HX-1	0%

Procedure: Under condition A, (*E*)-**3a** (0.2 mmol) were added to the reaction mixture. Yield was determined by ^{1}H NMR spectroscopy using CH_2Br_2 as an internal standard.

We achieved **4a** with 92% yield under standard conditions. While **4a** cannot be obtained by reducing the control variable of any catalytic component. These phenomena suggest that the results showed that light, cobalt catalyst, photosensitizer and Brønsted acid are all essential for the formation of **4a**, which rules out the possibility of Lewis acid or Brønsted acid promoting intramolecular Friedel-Crafts acylation.

2. Kinetic profile of the hydrofunctionalization of 1,3-diene (1a) with phenol (2a).

time/h	1a	3a	4a
0	100%	0%	0%
3	90%	10%	0%
6	72%	28%	0%
9	59%	41%	0%
12	48%	52%	0%
15	40%	59%	0%
18	30%	69%	0%
21	23%	76%	0%
24	15%	80%	0%
27	0%	72%	28%
30	0%	60%	40%
33	0%	46%	54%
36	0%	35%	60%
39	0%	24%	69%
42	0%	16%	78%
45	0%	5%	80%
48	0%	0%	83%

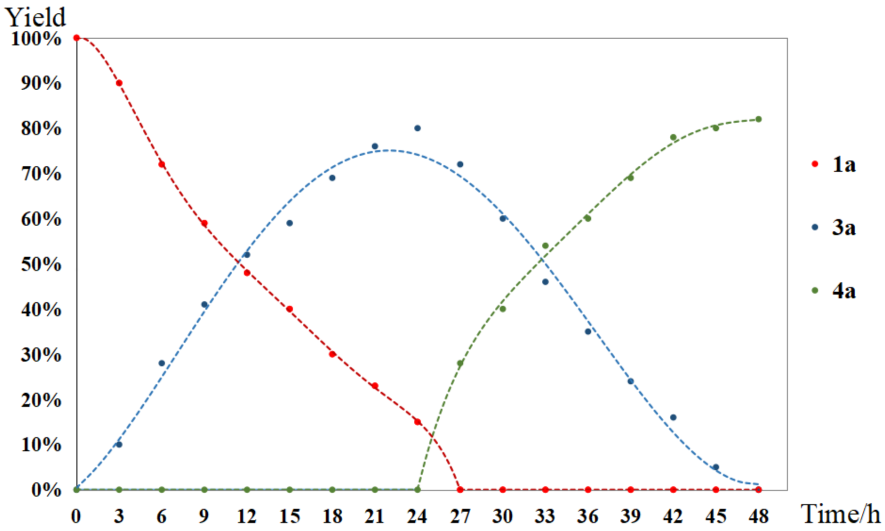

Fig S8 Kinetic profile of the hydrofunctionalization of 1,3-diene 1a with phenol 2a

Procedure: Under standard condition, **1a** (0.2 mmol) and **2a** (0.4 mmol) were added to the reaction mixture. 17 reactions were set in parallel and quenched at the corresponding time. Yield was determined by ^{1}H NMR spectroscopy using CH_2Br_2 as an internal standard.

3. Radical inhibition experiment

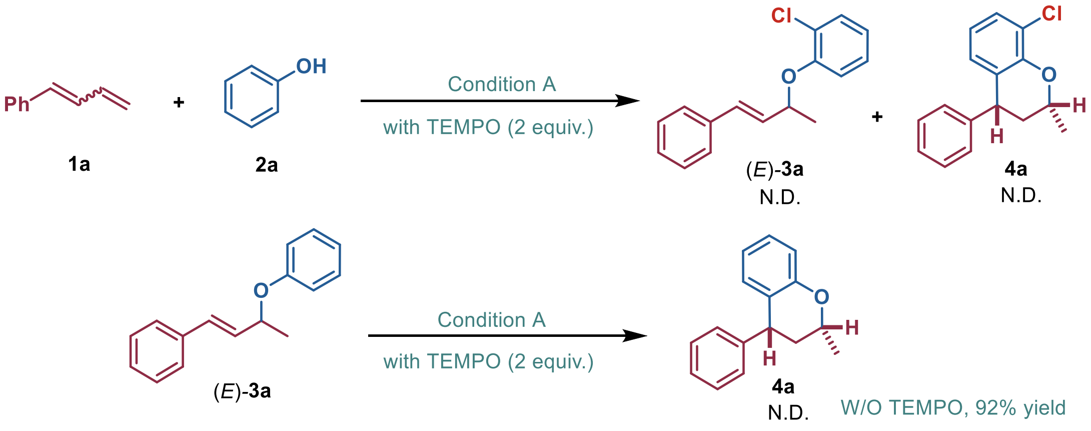

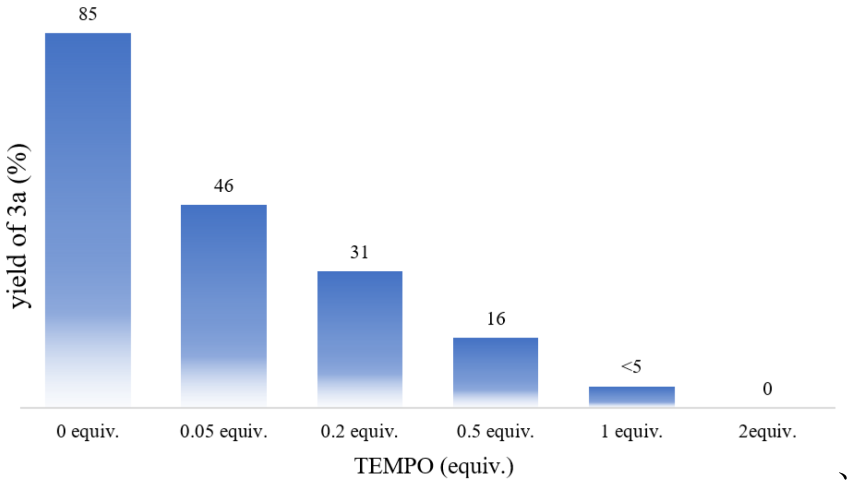

Procedure: Under condition A, using 1,3-diene **1a** as substrate, radical inhibitor 2,2,6,6-tetramethyl-1-piperidinyloxy (TEMPO) was added into the model reaction and the formation of **3a** and **4a** was progressively inhibited. In additon, under conditon A, using (*E*)-**3a** as substrate, TEMPO was added into the reaction and the formation of **4a** was also inhibited, compared with the 92% yield of **4a** without TEMPO.

This result suggested that radical intermediates may be involved in both transformations, consistent with the speculated metal-hydride HAT process.

4. Radical clock experiment

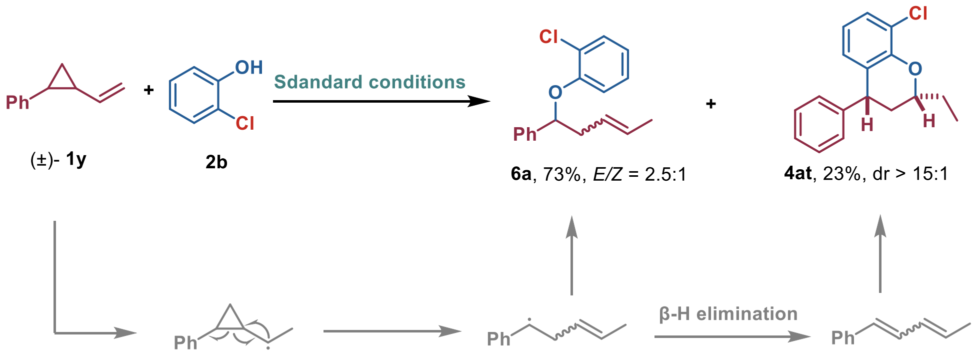

Procedure: Under condition A, (±)-**1y** (0.4 mmol) and **2b** (0.2 mmol) were added to the reaction mixture. Crude products were purified with silica gel column chromatography to afford pure compound.

The ring-opening product **6a** and dihydrofunctionalization product **4at** were detected in 73% and 23%, respectively. These phenomena suggest that an alkyl radical intermediate is probably involved in this transformation, in line with the speculated CoH-mediated HAT process.

1-chloro-2-((1-phenylpent-3-en-1-yl)oxy)benzene (6a)

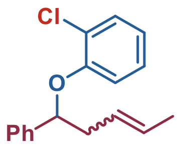

Colorless oil, 73% yield (39.7 mg), 2.5:1 *E/Z*. **^{1}H NMR (600 MHz, $CDCl_3$)** δ 7.38 – 7.32 (m, 5.6H), 7.27 – 7.23 (m, 2.8H), 7.00 (t, *J* = 7.2 Hz, 1.4H), 6.79 (t, *J* = 7.2 Hz, 1.4H), 6.71 (t, *J* = 7.2 Hz, 1.4H), 5.62 – 5.47 (m, 2.8H), 5.20 – 5.16 (m, 0.4H), 5.14 – 5.10 (m, 1H), 2.86 – 2.80 (m, 0.4H), 2.79 – 2.72 (m, 1H), 2.69 – 2.63 (m, 0.4H), 2.59 – 2.51 (m, 1H), 1.64 (d, *J* = 4.2 Hz, 3H), 1.55 (d, *J* = 6.6 Hz, 1H). **^{13}C NMR (151 MHz, $CDCl_3$)** δ 153.7, 153.6, 141.0, 140.9, 130.23, 130.20, 128.50, 128.49, 127.70, 127.67, 127.3, 127.0, 126.2, 126.14, 126.12, 125.1, 123.59, 123.57, 121.30, 121.26, 115.56, 115.49, 81.6, 81.0, 41.7, 35.9, 18.0, 12.9. **HRMS** (ESI-TOF) (m/z): calcd for $C_{17}H_{17}ClNaO$ ($[M+Na]^+$), 295.0860, found, 295.0864.

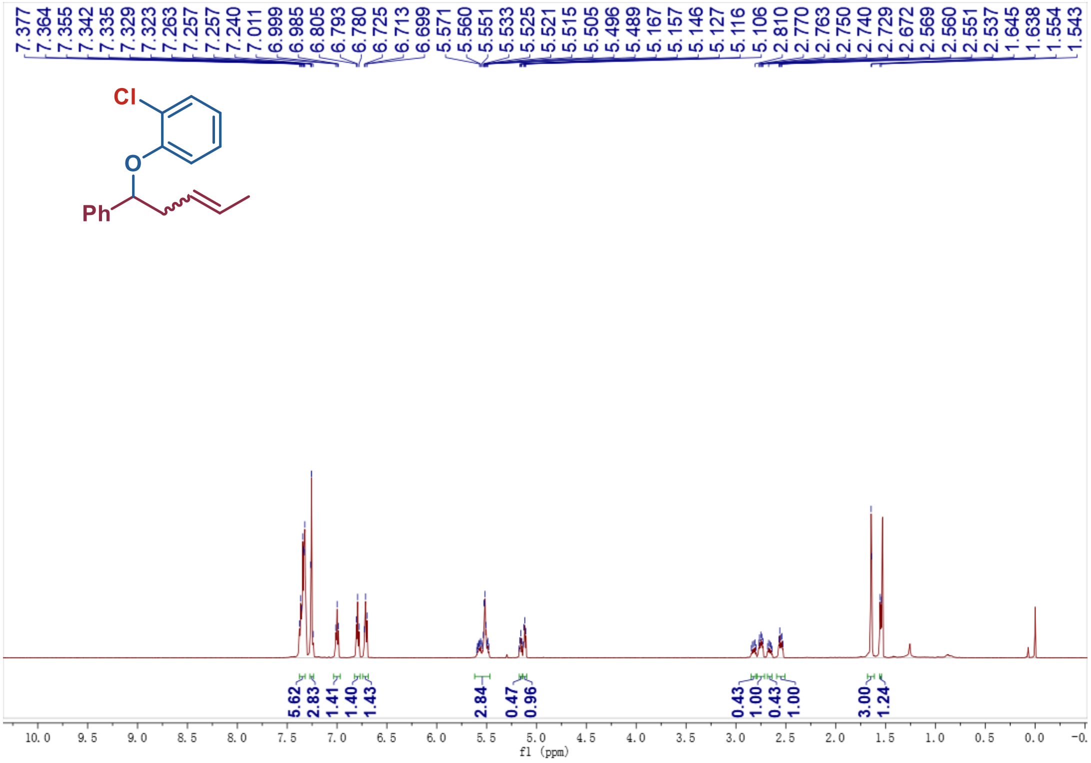

Fig. S9 ^{1}H NMR of ring-opening product 6a.

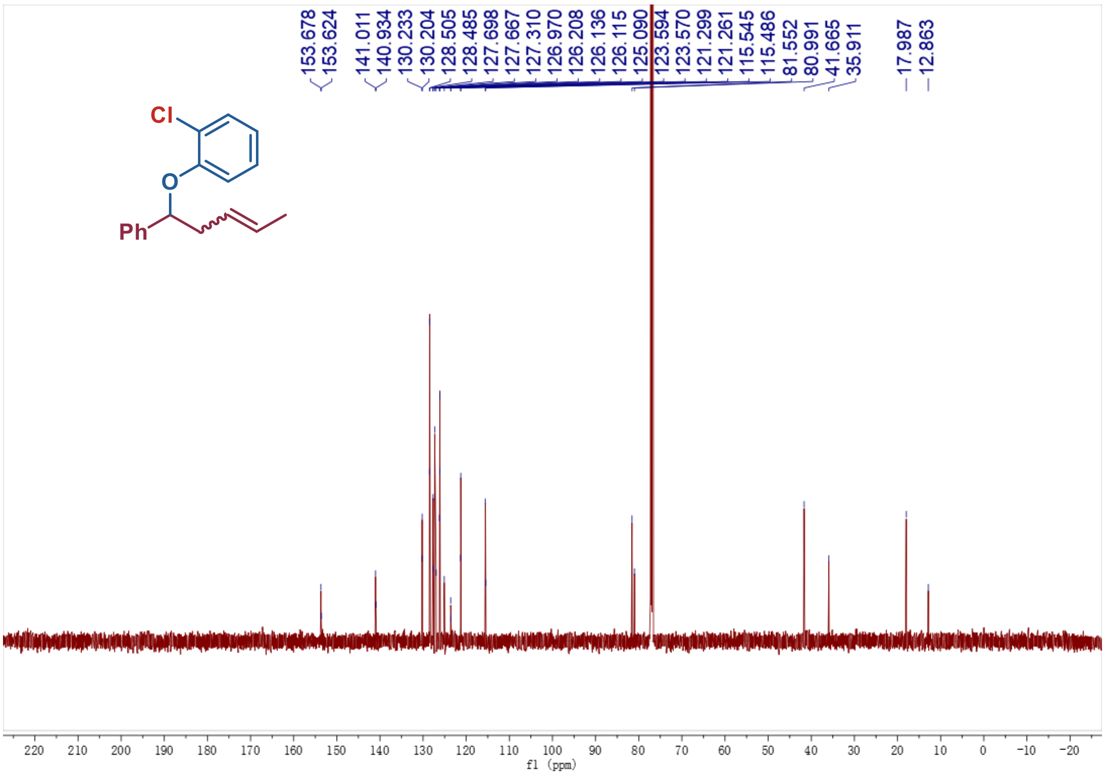

Fig. S10 ^{13}C NMR of ring-opening product 6a.

5. Stern-Volmer Luminescence Quenching Analysis

Several Stern-Volmer luminescence quenching experiments were carried out.

(1) Quencher: cobalt-salen complex Co-**1** (Fig. S11.)

Procedure: In a glovebox, to a screw-top 1.0 cm quartz cuvette was added $1*10^{-5}$ M solution of **Ir(ppy)$_3$** in degassed DCM and cobalt-salen complex **Co-1** (quencher) of appropriate concentration in degassed DCM (quencher concentration = 10 µM, 20 µM, 30 µM, 40 µM，50µM). The fluorescence intensity was measured at λ = 400 nm after excitation at λ = 375 nm in the quartz cuvette.

Co(µM)	I0/I	I0	I
10	1.107493104	430.827	389.011
20	1.291753743	430.827	333.521
30	1.48267567	430.827	290.574
40	1.72988155	430.827	249.05
50	1.927784216	430.827	223.483

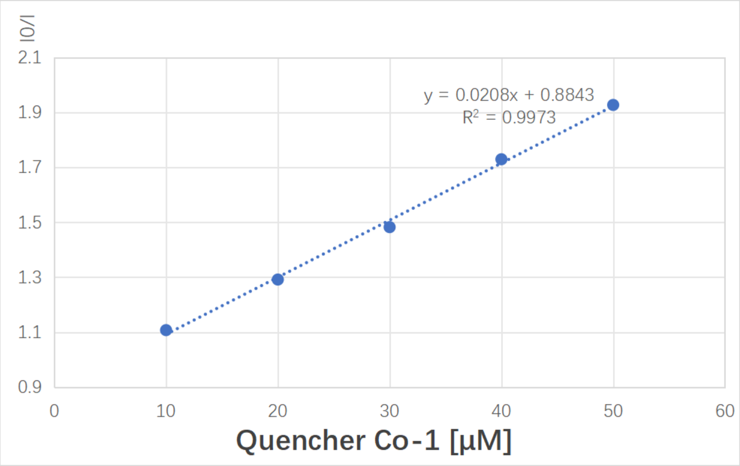

Fig. S11

(2) Quencher: colldinium salt HX-**1**(Fig. S12.)

Procedure: In a glovebox, to a screw-top 1.0 cm quartz cuvette was added $1*10^{-5}$ M solution of **Ir(ppy)$_3$** in degassed DCM and collidinium salt (quencher) of appropriate concentration in degassed DCM (quencher concentration = 10 µM, 20 µM, 30 µM, 40 µM，50µM). The fluorescence intensity was measured at λ = 400 nm after excitation at λ = 375 nm in the quartz cuvette.

HX-1(µM)	I0/I	I0	I
10	1.002298301	460.962	459.905
20	1.060104363	460.962	434.827
30	1.026556832	460.962	449.037
40	0.99987853	460.962	461.018
50	1.035925533	460.962	444.976

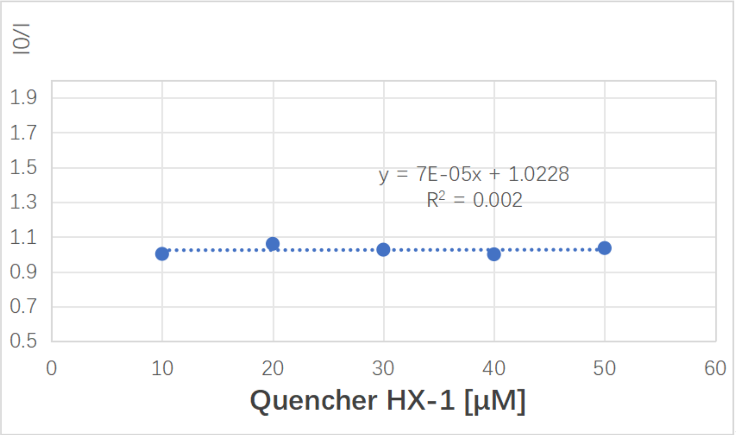

Fig. S12

(3) Quencher: 1,3-dienes (Fig. S13.)

Procedure: In a glovebox, to a screw-top 1.0 cm quartz cuvette was added 1*10^{-5} M solution of **Ir(ppy)$_3$** in degassed DCM and 1,3-dienes (quencher) of appropriate concentration in degassed DCM (quencher concentration = 10 μM, 20 μM, 30 μM, 40 μM， 50μM). The fluorescence intensity was measured at λ = 400 nm after excitation at λ = 375 nm in the quartz cuvette.

1,3-dienes(μM)	I0/I	I0	I
10	1.023523803	467.995	457.239
20	1.022403548	467.995	457.74
30	1.014889543	467.995	461.129
40	1.030871404	467.995	453.98
50	0.983579511	467.995	475.808

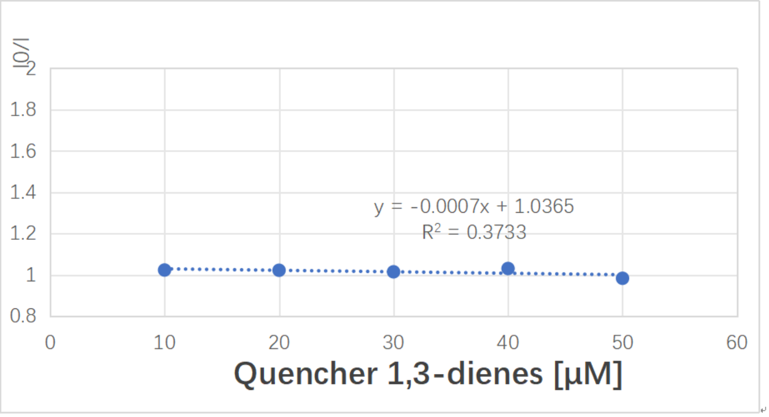

Fig. S13

(4) Quencher: Phenols (Fig. S14.)

Procedure: In a glovebox, to a screw-top 1.0 cm quartz cuvette was added 1*10^{-5} M solution of **Ir(ppy)$_3$** in degassed DCM and Phenols (quencher) of appropriate concentration in degassed DCM (quencher concentration = 10 μM, 20 μM, 30 μM, 40 Mm, 50μM). The fluorescence intensity was measured at λ = 400 nm after excitation at λ = 375 nm in the quartz cuvette.

Phenols(μM)	I0/I	I0	I
10	1.015761021	459.963	452.826
20	0.995491791	459.963	462.046
30	1.018942784	459.963	451.412
40	1.029919324	459.963	446.601
50	0.977093804	459.963	470.746

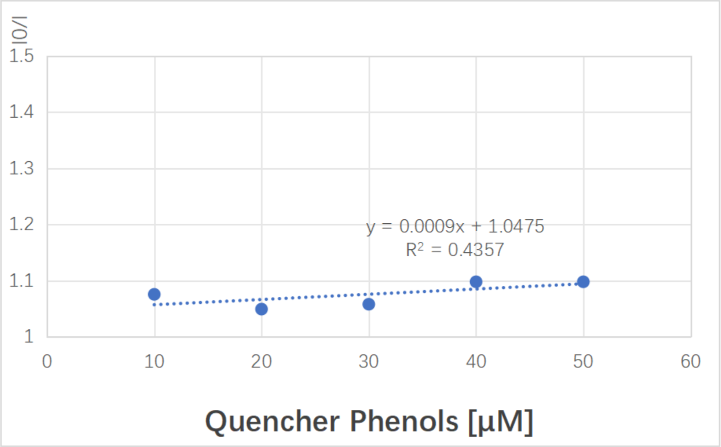

Fig. S14

The Stern-Volmer experiments revealed that the fluorescence of **Ir(ppy)$_3$** is quenched by Co-**1**, but not by HX-**1, 1,3-dienes** and **Phenols**.

6. Deuterium experiment

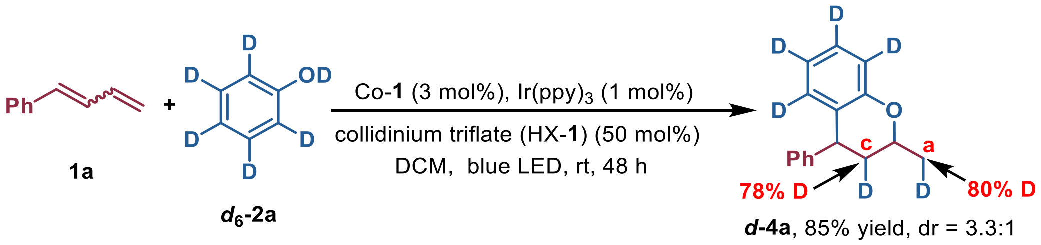

*Procedure:*Under condition B, ***d*$_6$-2a** (0.2 mmol) and **1a** (0.4 mmol) were added to the reaction mixture. Crude products were purified with silica gel column chromatography to afford pure compound. Crude products were purified with silica gel column chromatography to afford pure compound ***d*-4a** in 85% yield.

2-(methyl-*d*)-4-phenylchromane-3,5,6,7,8-*d*$_5$ (*d*-4a)

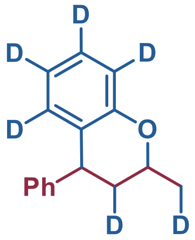

colorless oil, 85% yield, 3.3:1 dr. ^{1}**H NMR** (600 MHz, CDCl$_3$) δ 7.31 (t, *J* = 7.2 Hz, 1.5H), 7.29 – 7.21 (m, 1.5H), 7.17 (d, *J* = 6.6 Hz, 1.5H), 7.08 (d, *J* = 7.2 Hz, 0.5H), 4.33 – 4.19 (m, 1H), 4.19 – 4.09 (m, 1H), 2.22 – 2.16 (m, 0.45H), 2.13 – 2.06 (m, 0.16H), 2.01 – 1.97 (m, 0.15H), 1.97 – 1.88 (m, 0.46H), 1.42 (d, *J* = 6.0 Hz, 1.7H), 1.32 (d, *J* = 6.0 Hz, 0.5H). ^{13}**C NMR** (151 MHz, CDCl$_3$) δ 155.4, 146.7, 145.0, 129.6 – 129.2 (m), 128.62, 128.58, 128.5, 128.3, 127.5 – 126.9 (m), 126.6, 126.2, 125.6, 122.8, 119.9 – 119.4 (m), 116.6 – 115.6 (m), 72.3 (d, *J* = 7.2 Hz), 67.3 (d, *J* = 6.9 Hz), 43.0 (d, *J* = 13.1 Hz), 40.1 (d, *J* = 3.3 Hz), 34.0 , 37.8 , 21.6 (d, *J* = 3.4 Hz), 21.1 (d, *J* = 3.5 Hz).

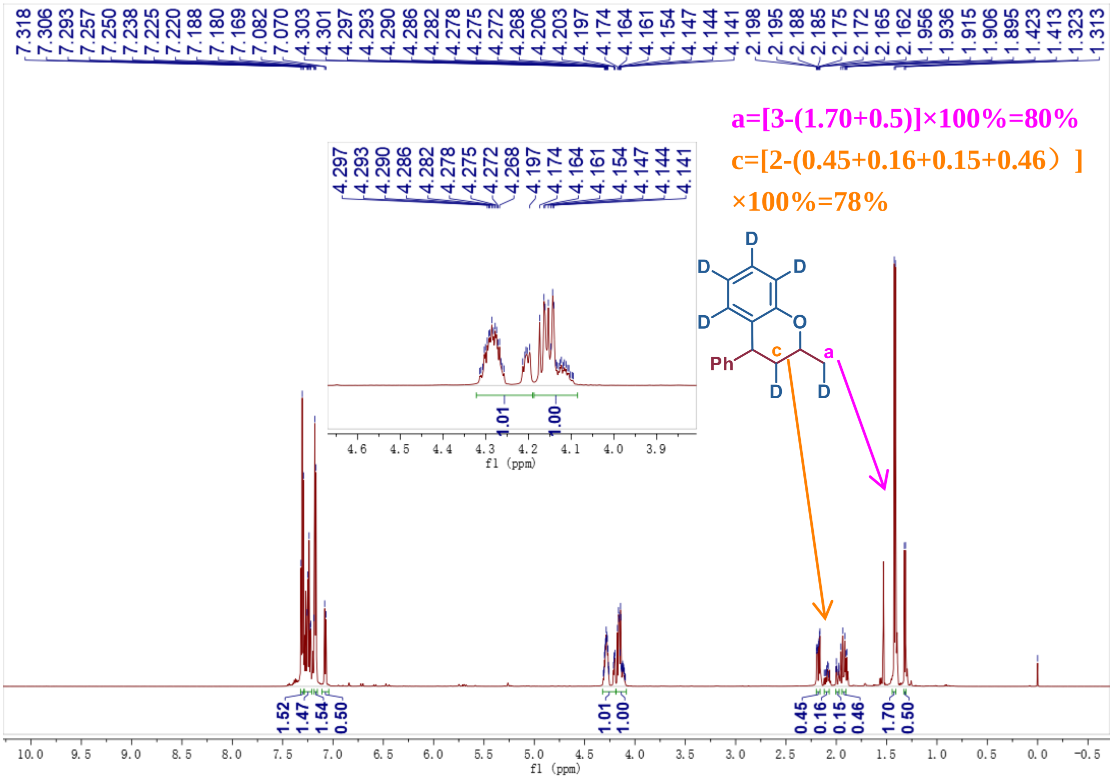

Fig. S15 ^{1}H NMR data of product *d*-4a.

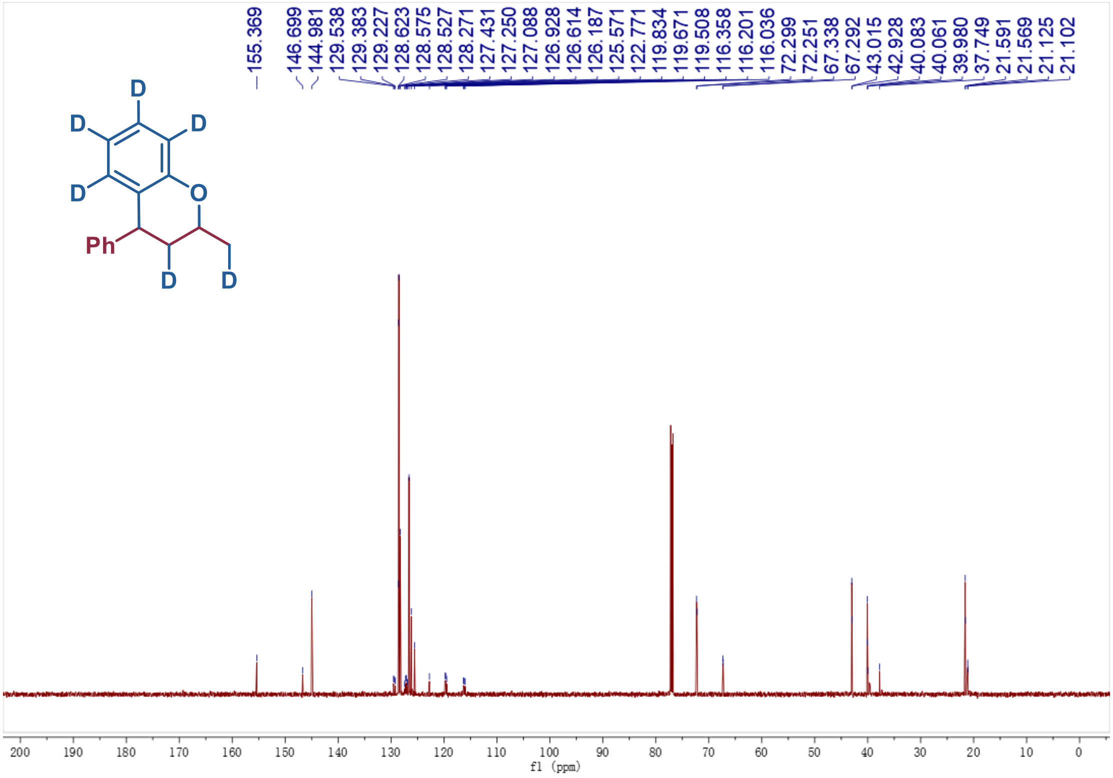

Fig. S16 ^{13}C NMR data of product *d*-4a.

7. Turn on/off profile experiment

Procedure: In a glovebox, to an oven-dried vial with a stirring bar was added photoredox catalyst Ir(ppy)$_3$ (1 mol%), Co-**1** (3 mol%), HX-**1** (20 mol%), DCM (1.0 mL). Then, **2a** (1.0 equiv) and **1a** (2.0 equiv) were added to the reaction mixture. Bibromomethane (0.2 mmol) was then added and the reaction mixture was stirred for 3 min. After sealing the vial with a cap and removal from the glove box, the reaction was stirred and irradiated with 40W 448 nm Blue LEDs with the temperature around rt. 50 microliters were sampled each time, and two samples were taken in parallel. Yield through determined by ^{1}H NMR spectroscopy. It was found that the formation of **3a** needed continuous irradiation of light, instead of getting the product through a radical chain reaction.

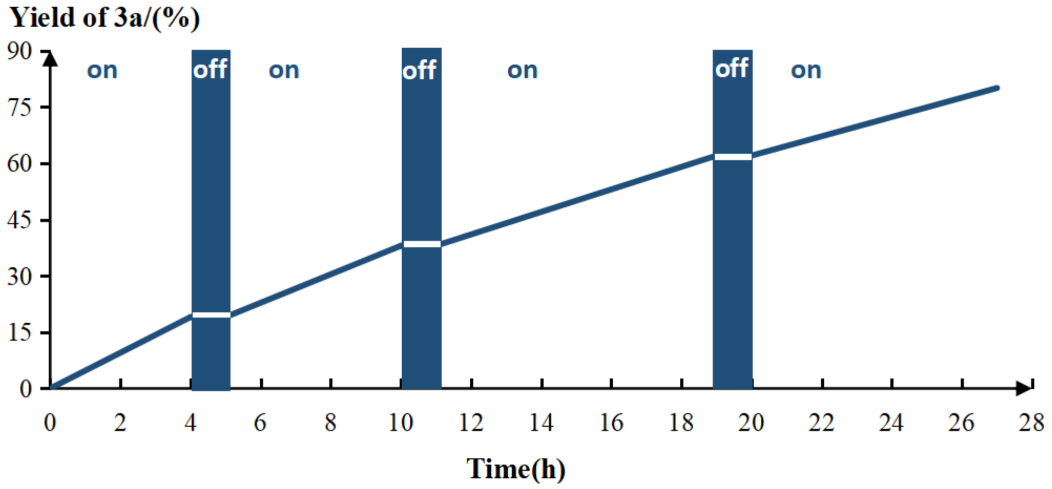

Fig. S17 Light on-off Experiment.

VIII. Reference

(1) D. F. Pünner, D. A. Schmidt, G. Hilt, *Angew. Chem. Int. Ed.* **2019,** *58*, 17103–17103.

(2) K. Pak S. Cheung, D. Kurandina, T. Yata, V. Gevorgyan, *J. Am. Chem. Soc.* **2020**, *142*, 9932–9937.

(3) M. Mendel, T. M. Karl, J, Hamm, S. J. Kaldas, T. Sperger, B. Mondal, F. Schoenebeck, *Nature*. **2024**, 631, 80–86.

(4) Y. Tu, B. Xu, Q. Wang, H. Dong, Z. Zhang, J. Zhang, *J. Am. Chem. Soc.* **2023**, *145*, 4378–4383.

(5) M. Nakagawa, Y. Matsuki, K. Nagao, H. Ohmiya, *J. Am. Chem. Soc.* **2022**. *144*, 7953–7959.

IX. NMR spectra

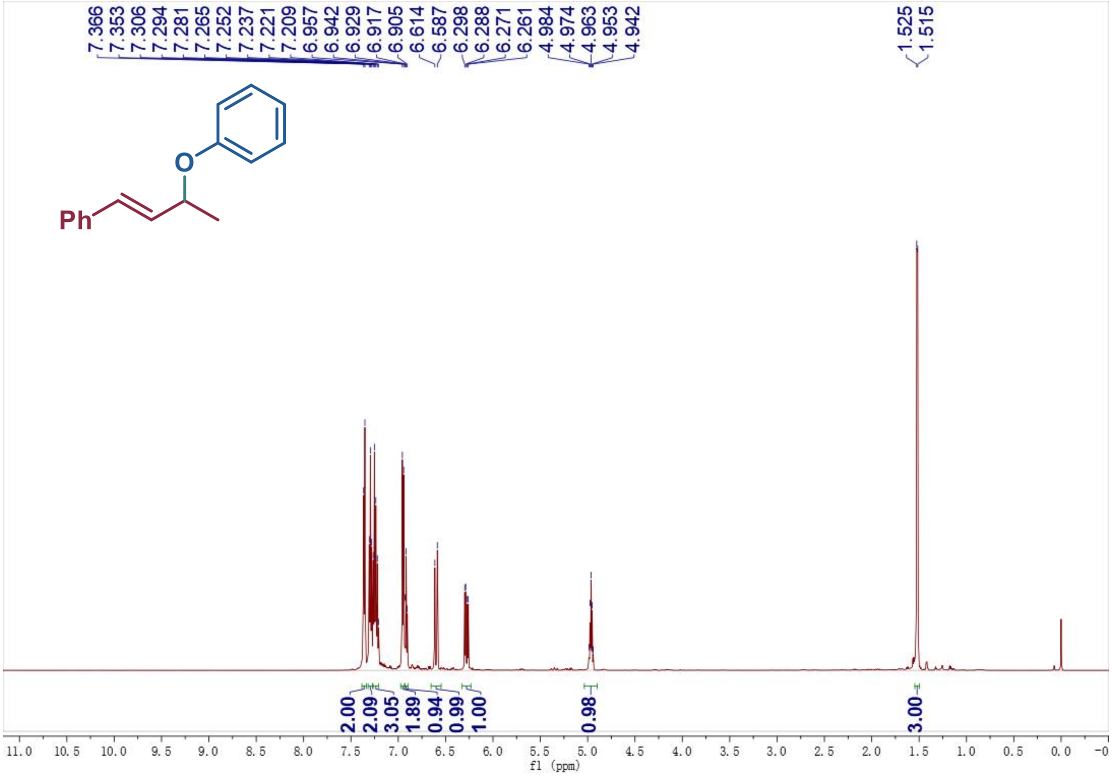

Fig. S18 ^{1}H NMR data of product 3a.

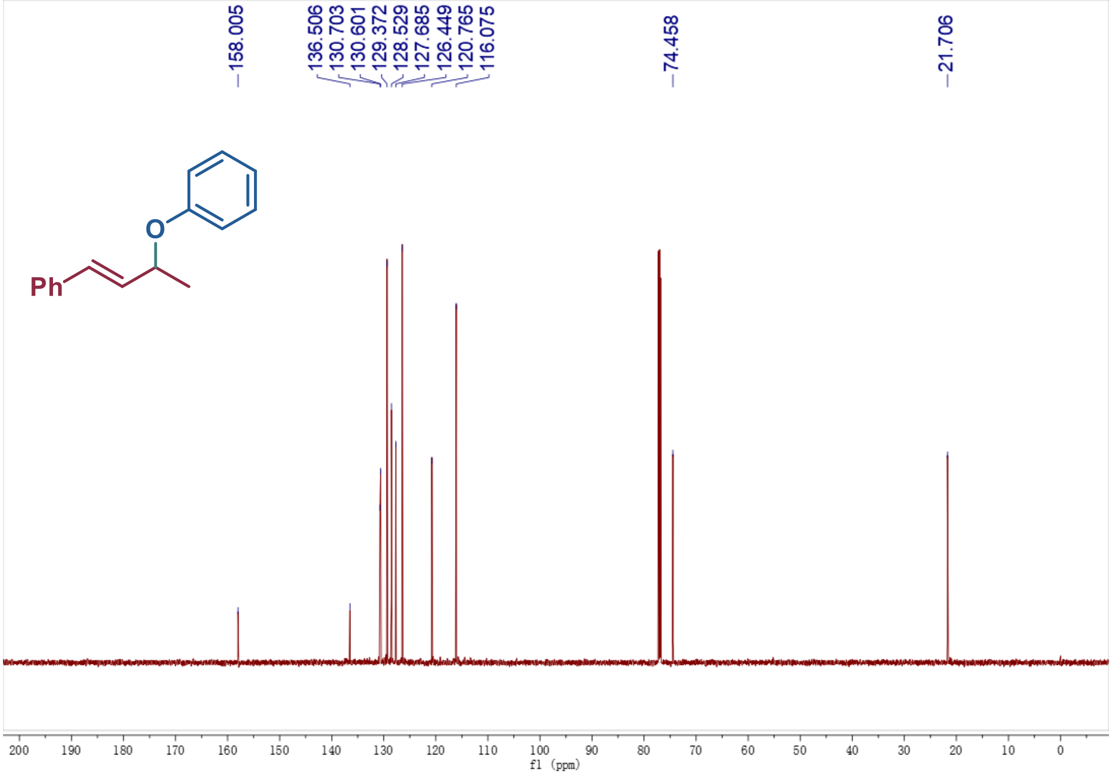

Fig. S19 ^{13}C NMR data of product 3a.

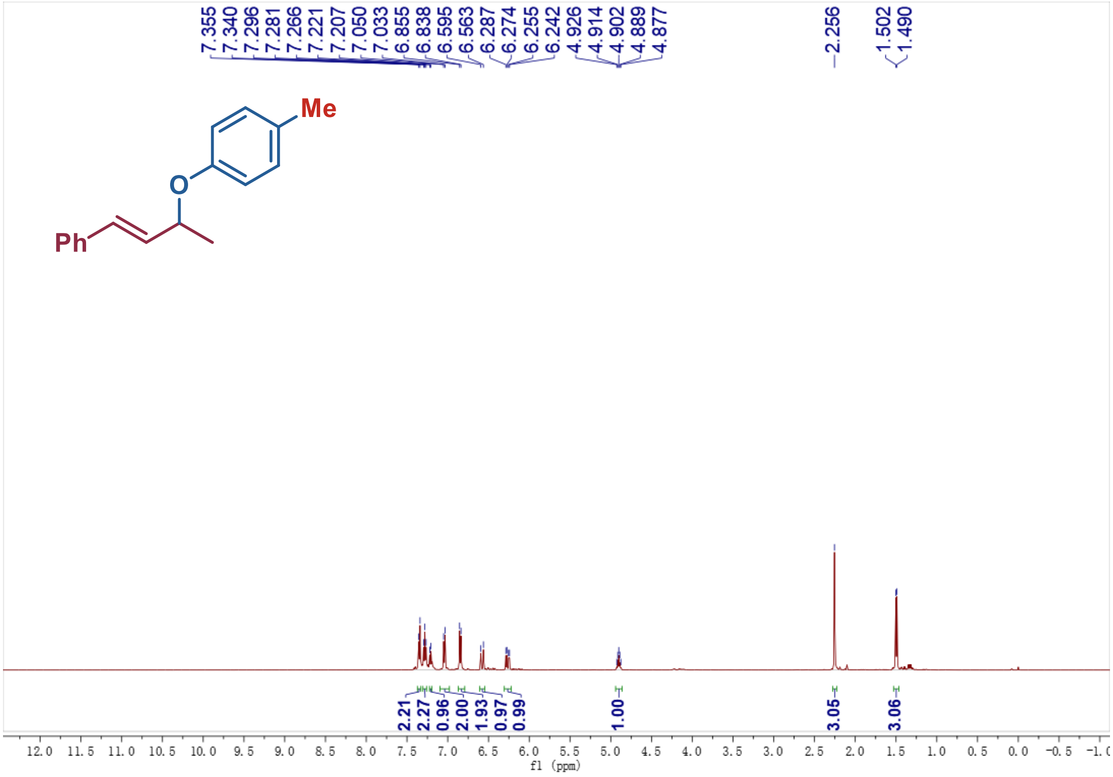

Fig. S20 ^{1}H NMR data of product 3b.

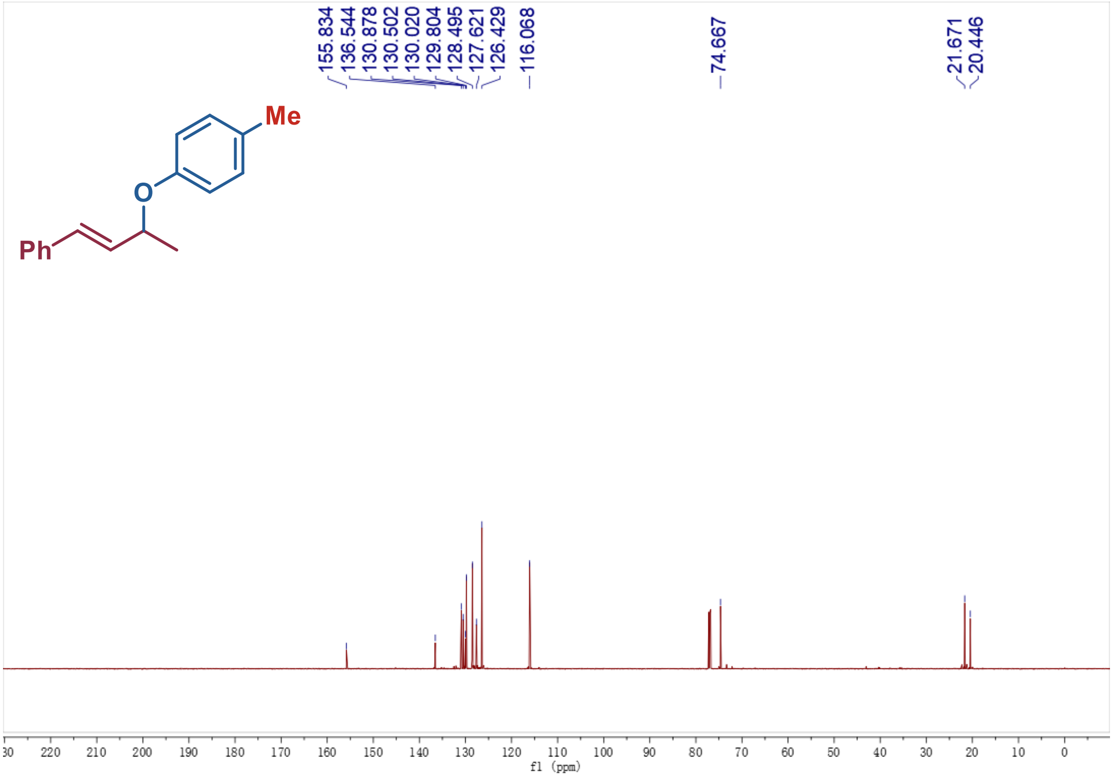

Fig. S21 ^{13}C NMR data of product 3b.

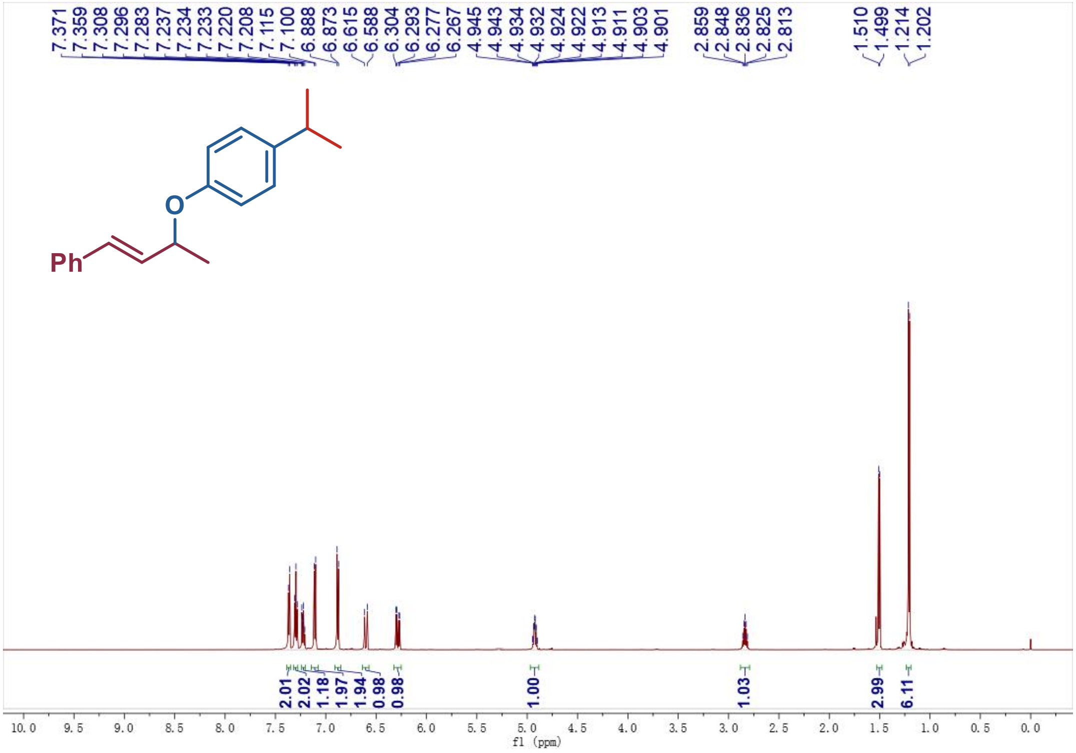

Fig. S22 ^{1}H NMR data of product 3c.

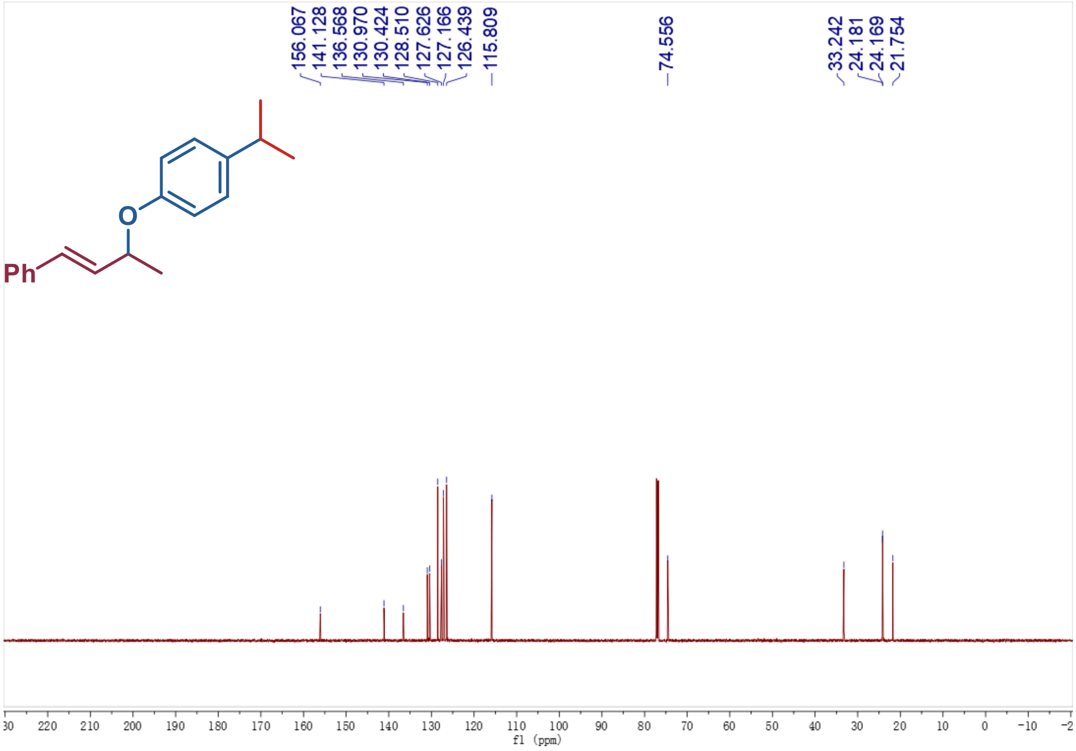

Fig. S23 ^{13}C NMR data of product 3c.

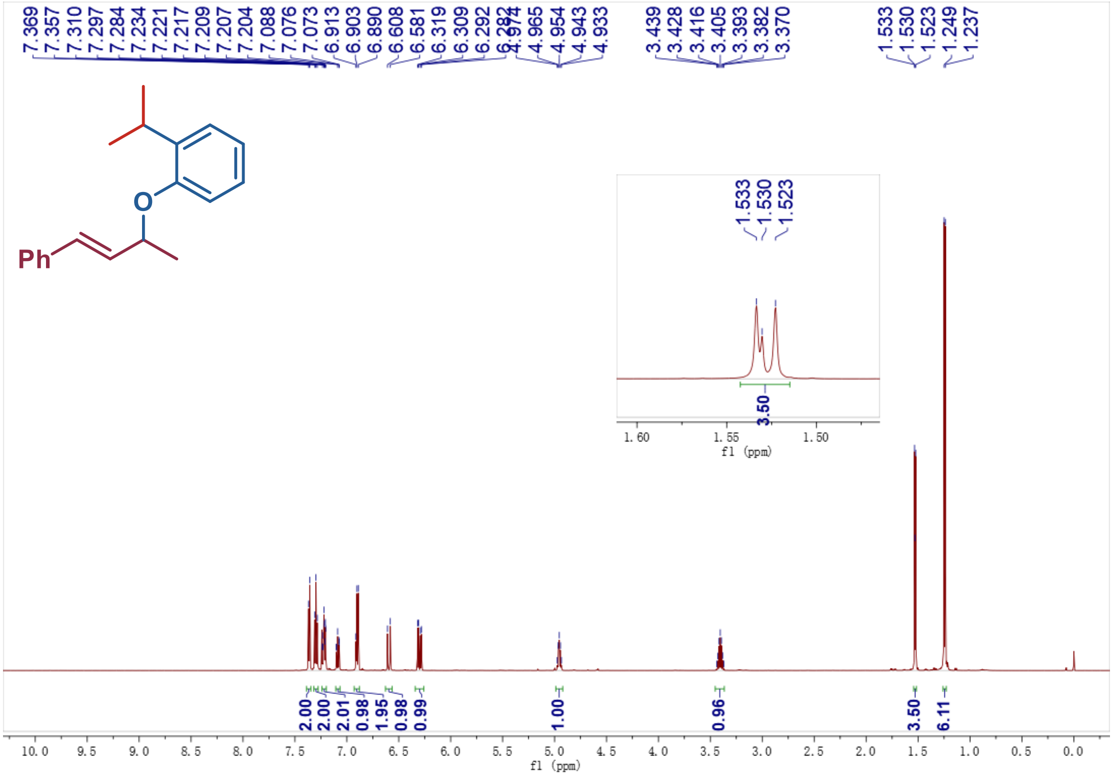

Fig. S24 ^{1}H NMR data of product 3d.

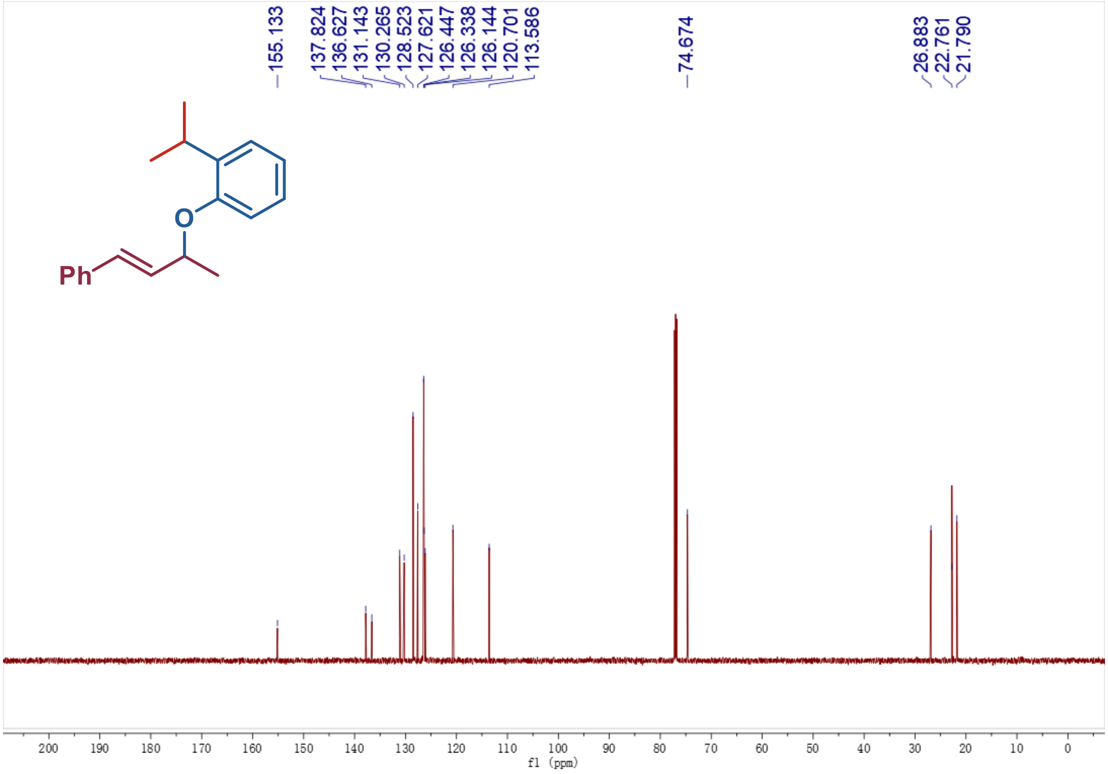

Fig. S25 ^{13}C NMR data of product 3d.

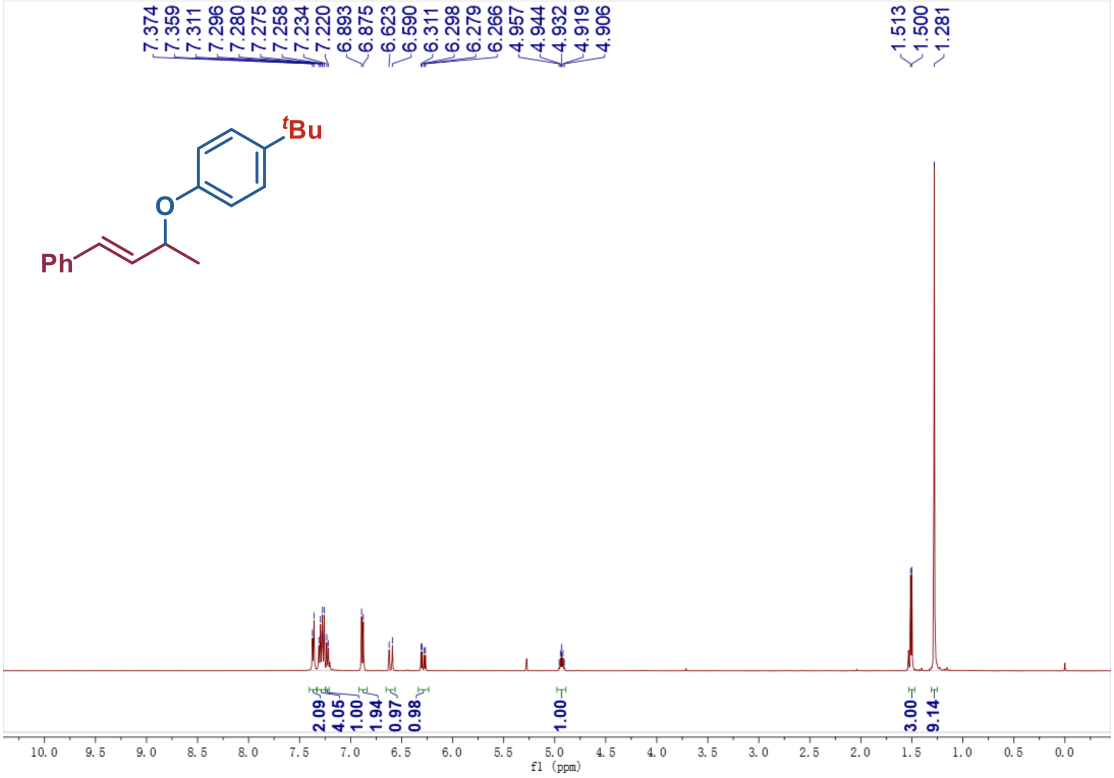

Fig. S26 ^{1}H NMR data of product 3e.

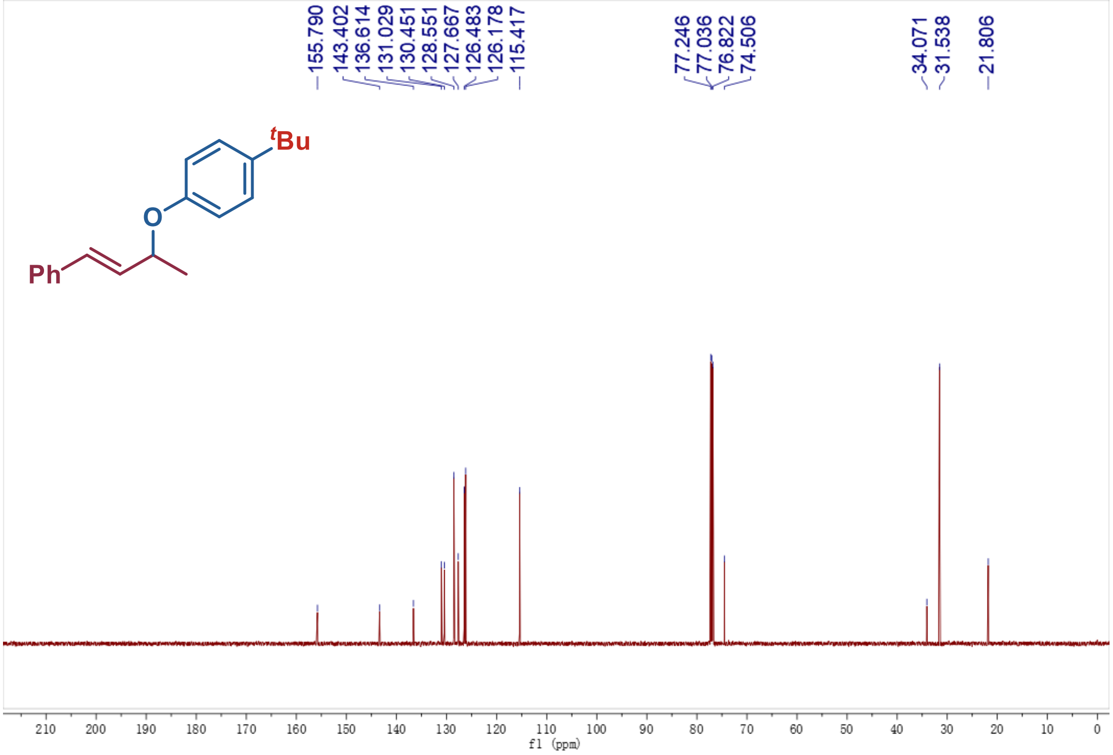

Fig. S27 ^{13}C NMR data of product 3e.

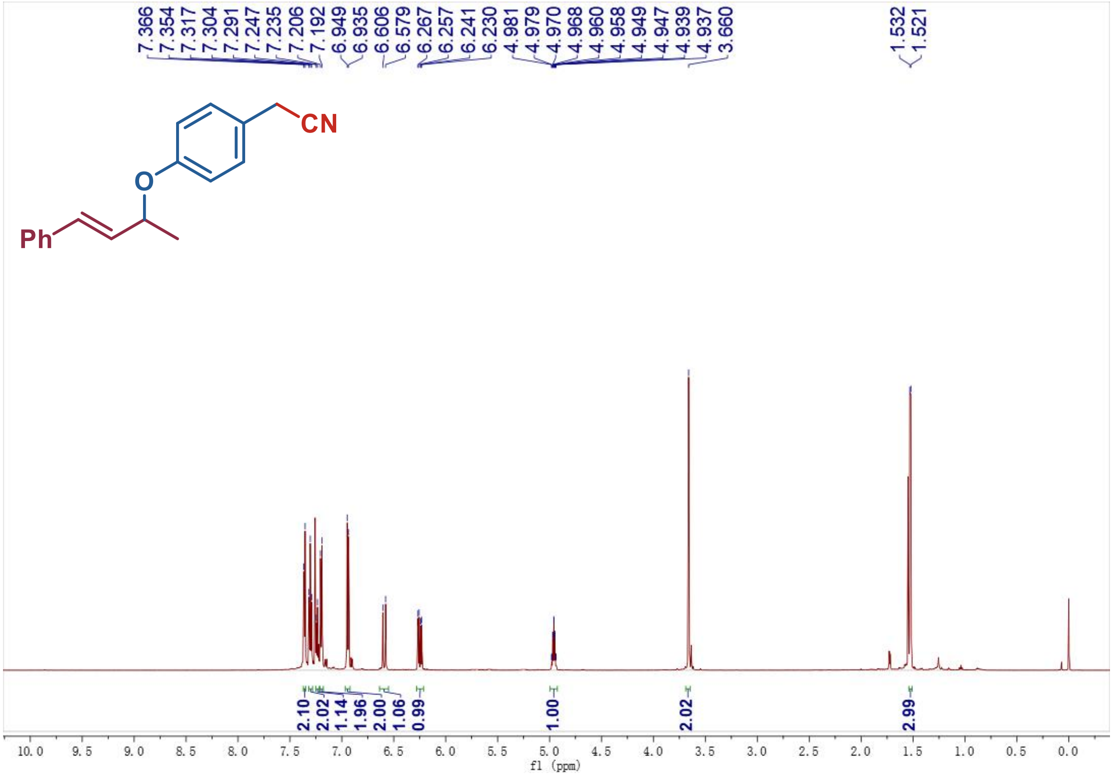

Fig. S28 ^{1}H NMR data of product 3f.

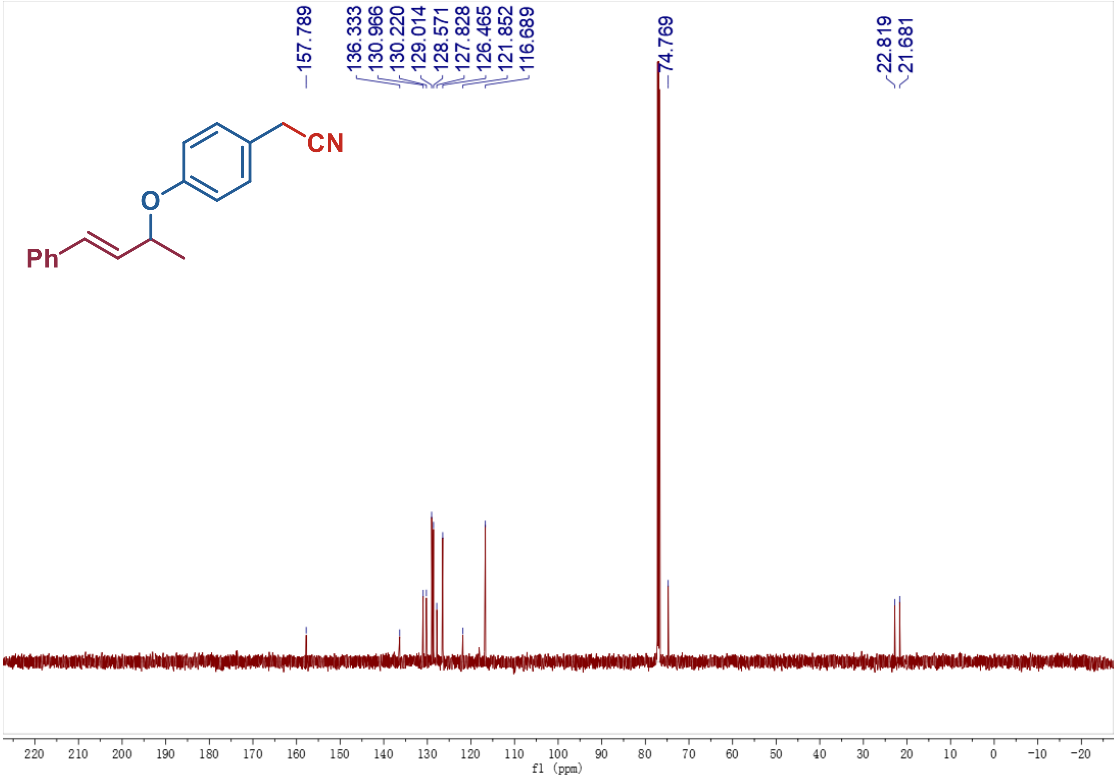

Fig. S29 ^{13}C NMR data of product 3f.

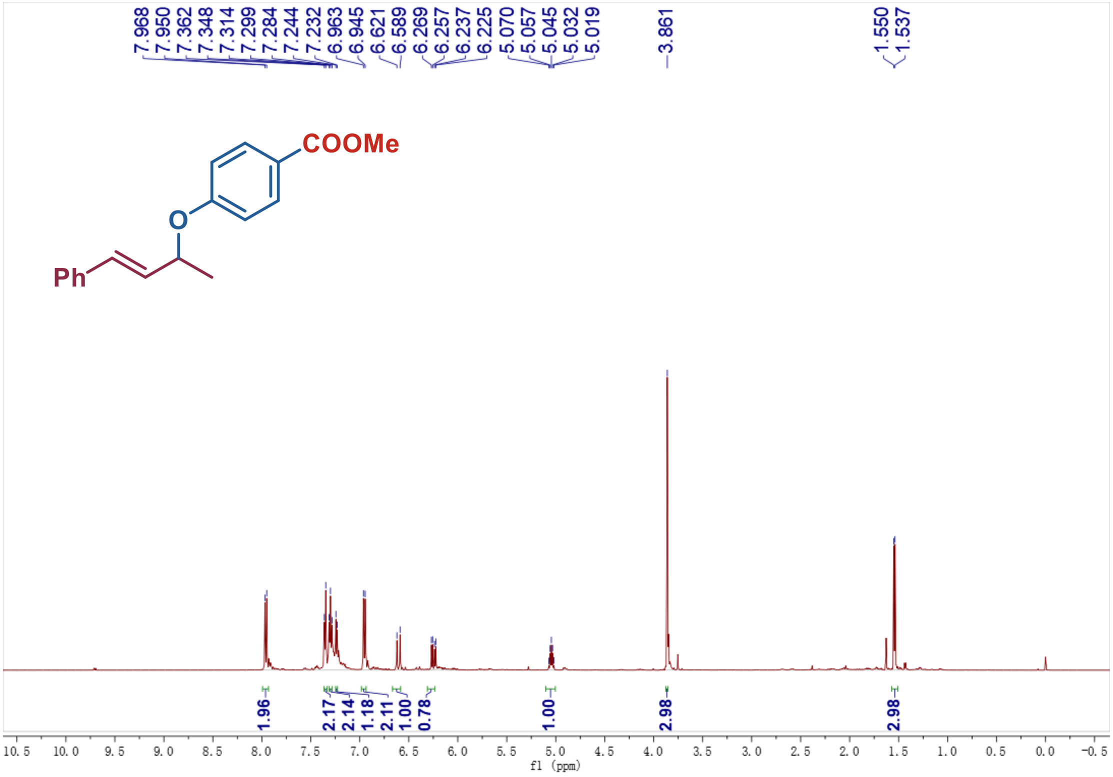

Fig. S30 ^{1}H NMR data of product 3g.

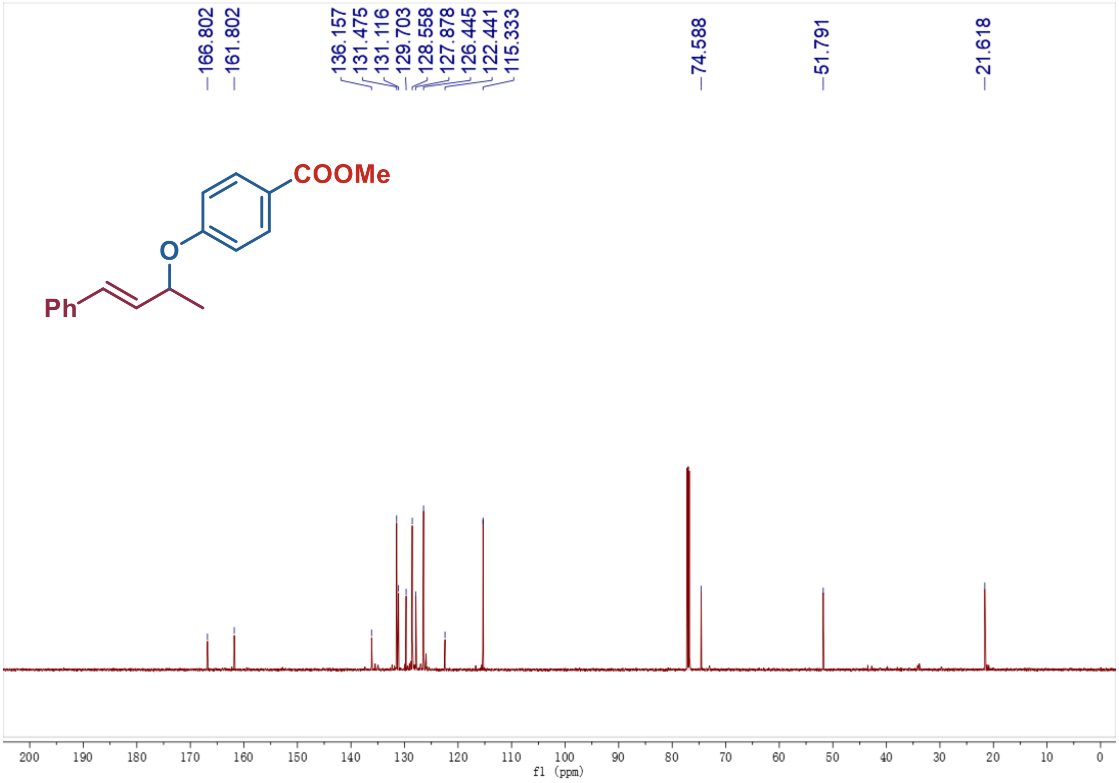

Fig. S31 ^{13}C NMR data of product 3g.

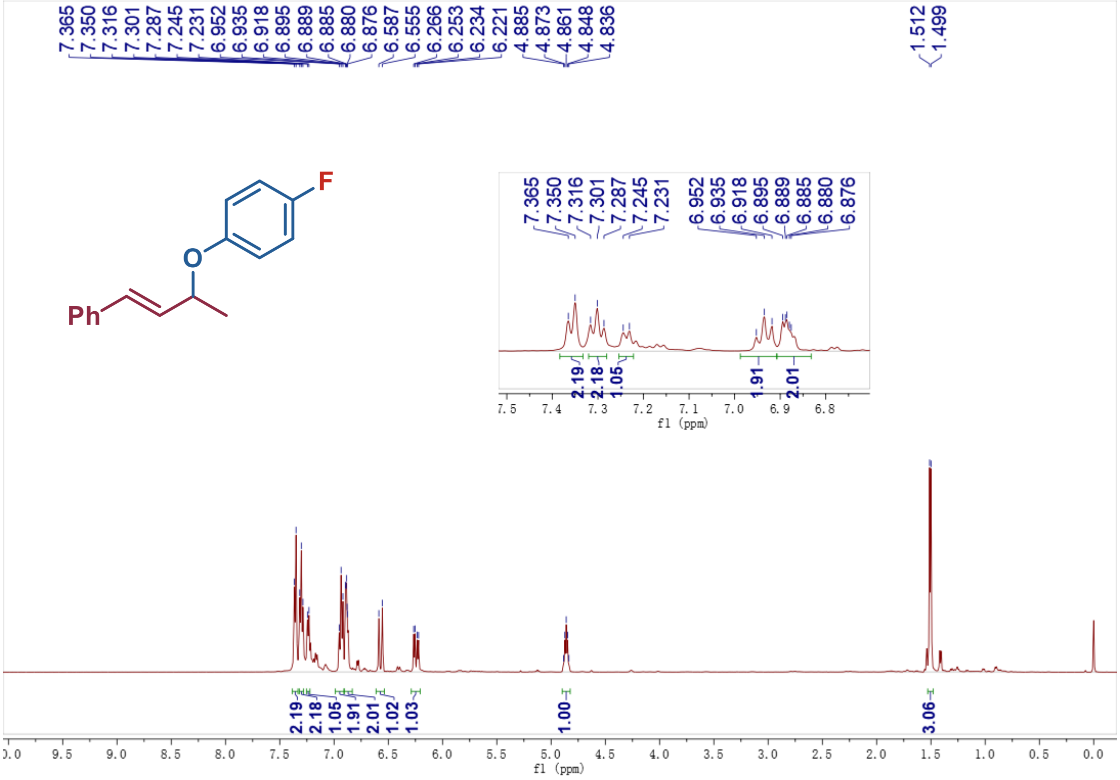

Fig. S32 ^{1}H NMR data of product 3h.

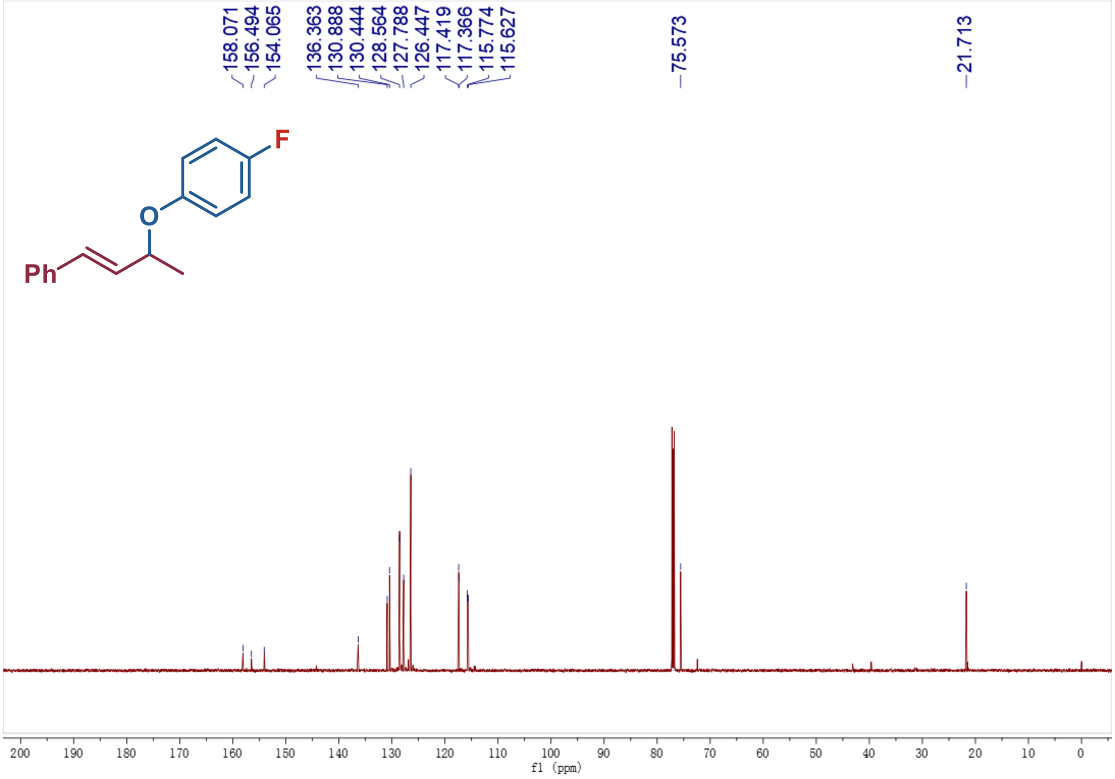

Fig. S33 ^{13}C NMR data of product 3h.

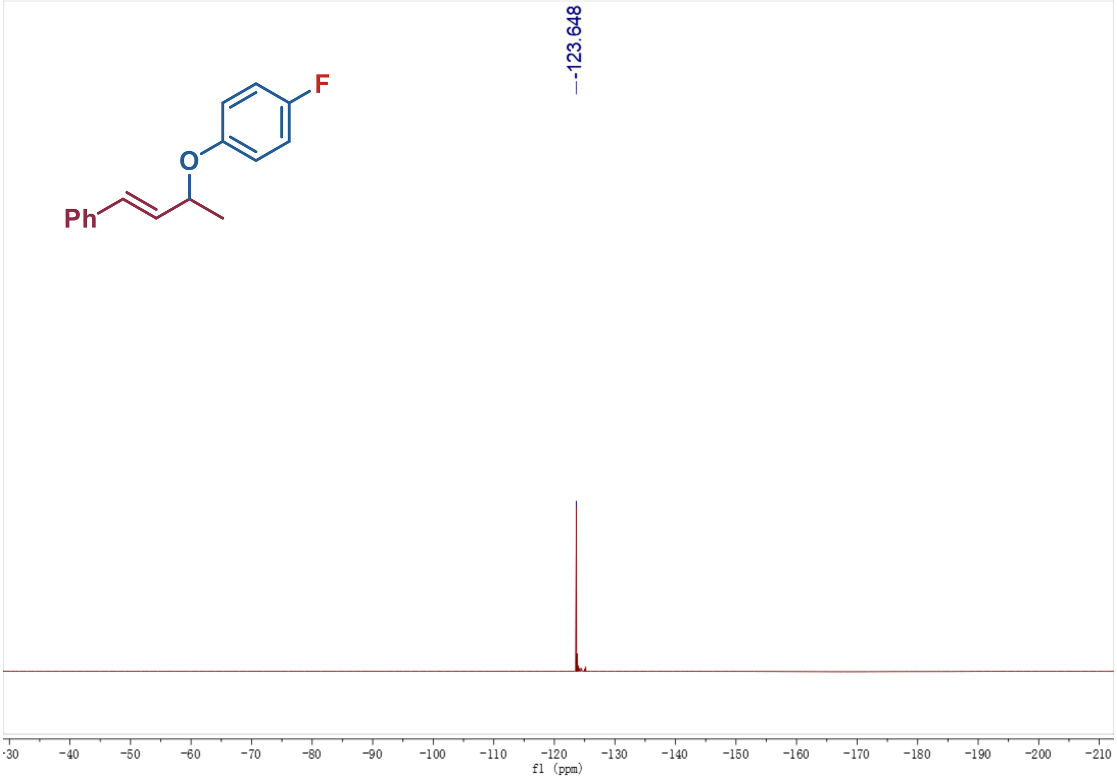

Fig. S34 ^{19}F NMR data of product 3h.

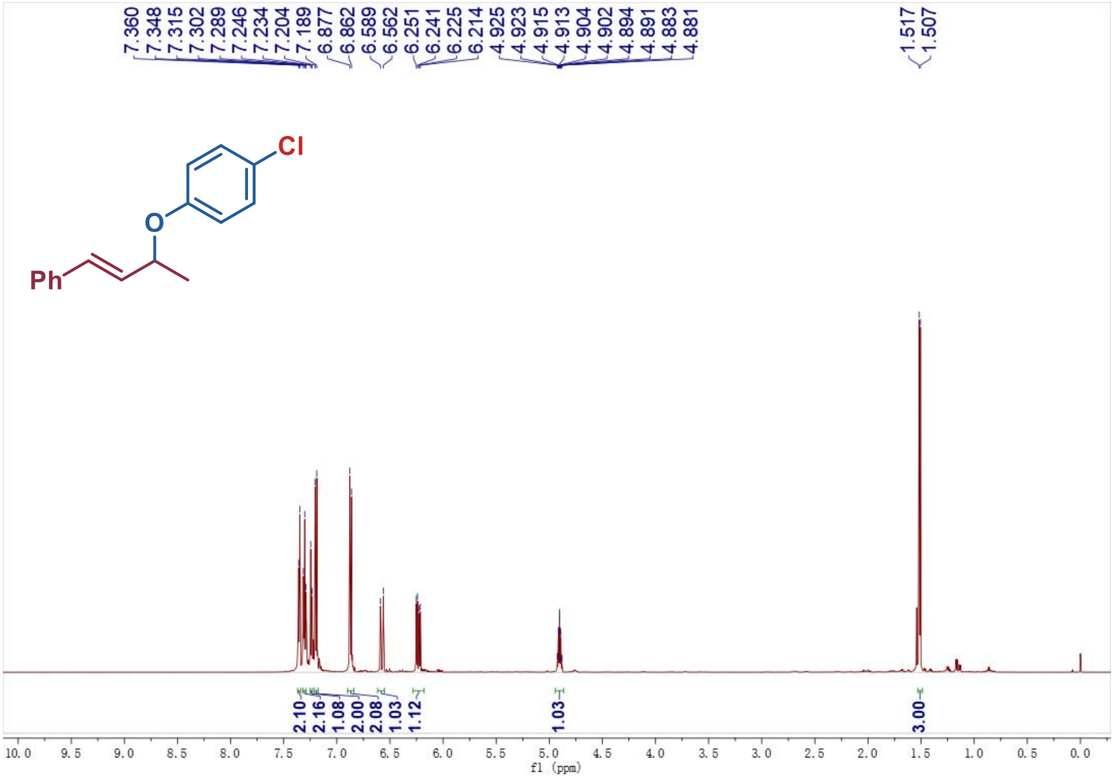

Fig. S35 ^{1}H NMR data of product 3i.

.

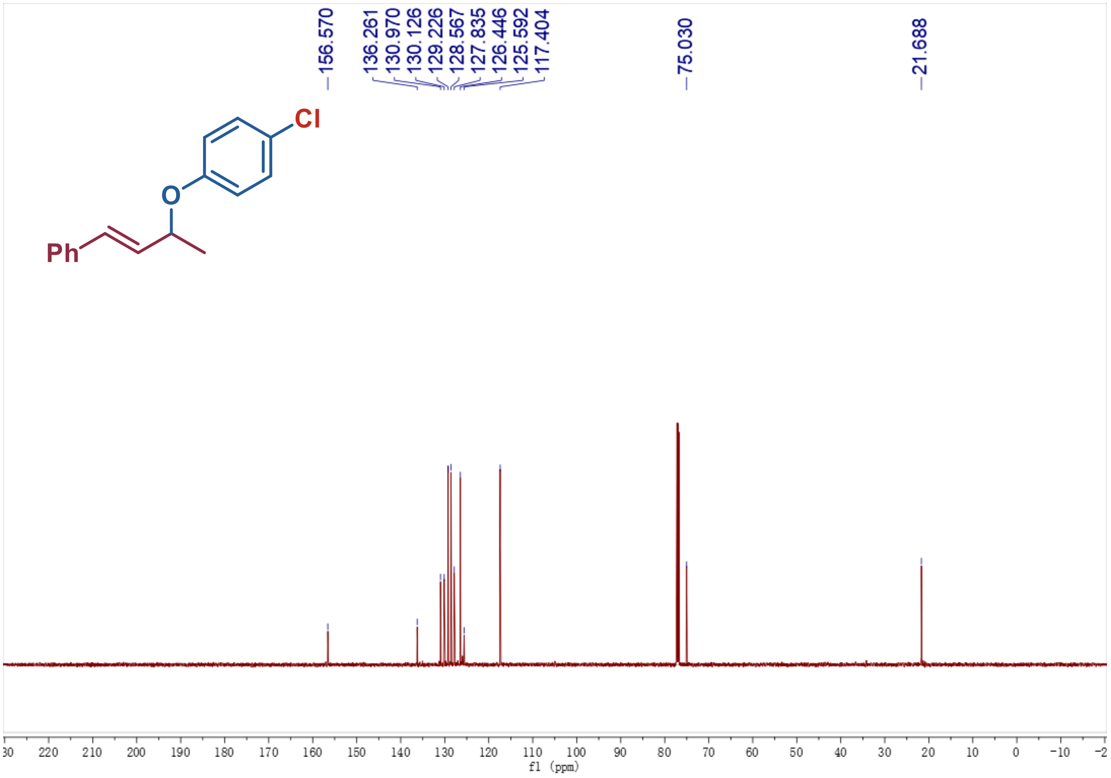

Fig. S36 ^{13}C NMR data of product 3i.

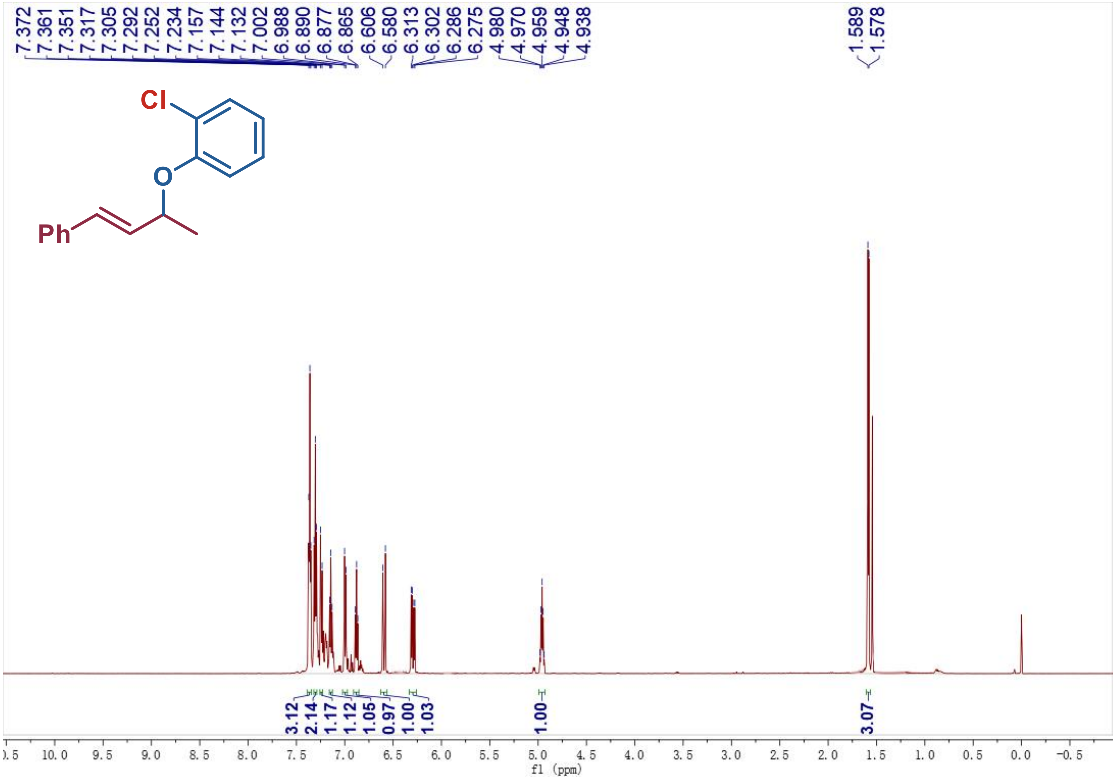

Fig. S37 ^{1}H NMR data of product 3j.

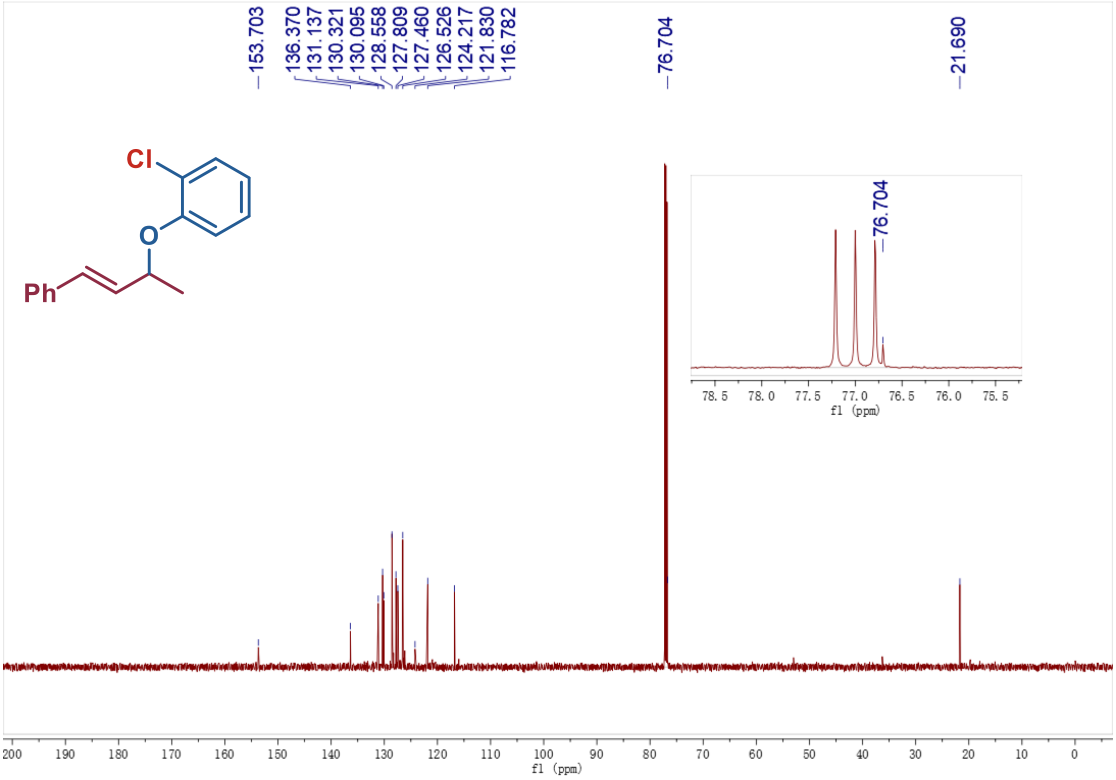

Fig. S38 ^{13}C NMR data of product 3j.

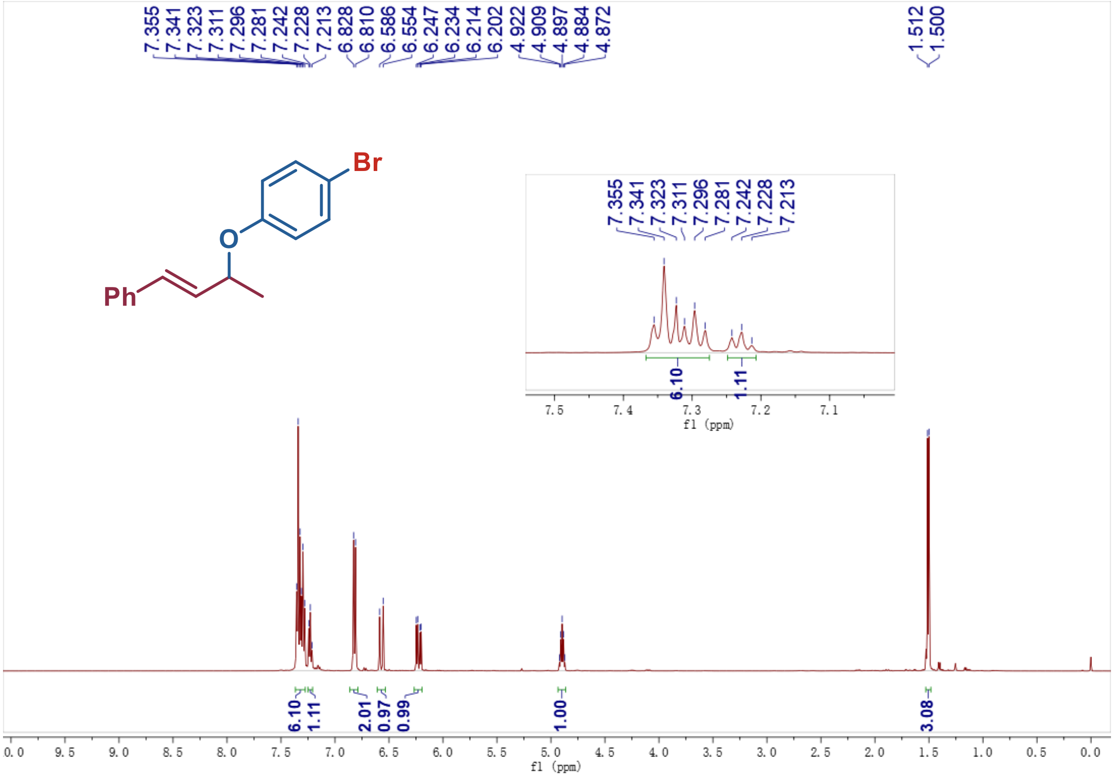

Fig. S39 ^{1}H NMR data of product 3k.

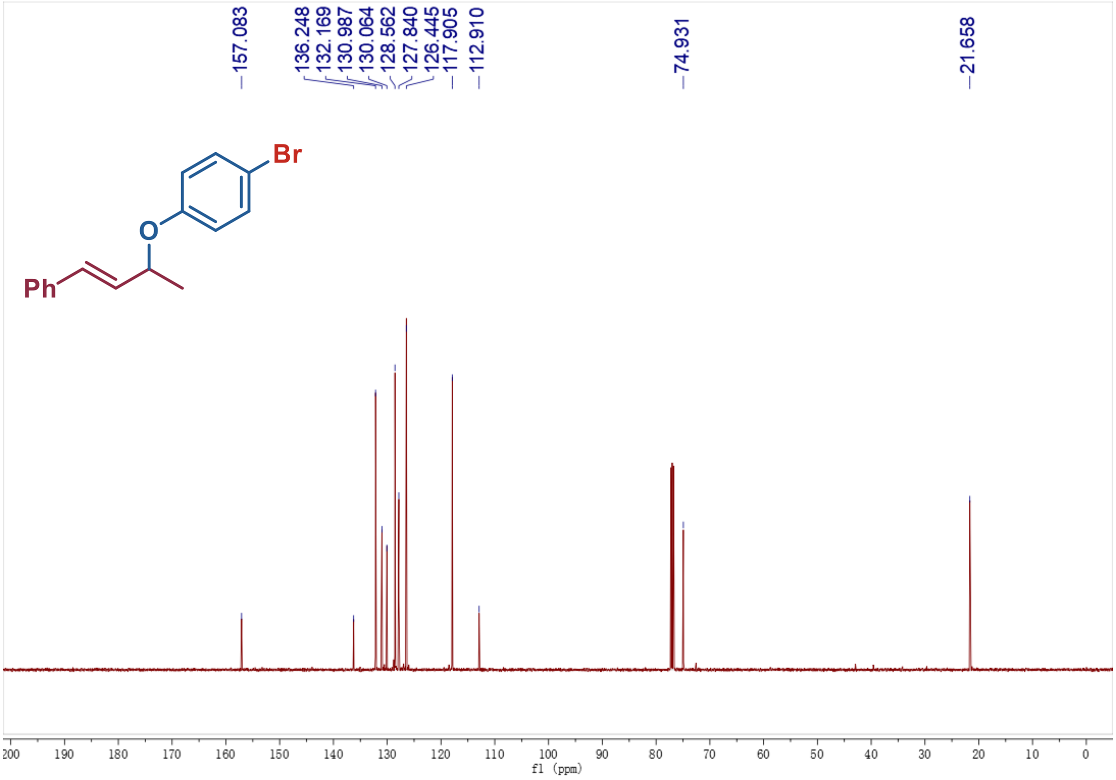

Fig. S40 ^{13}C NMR data of product 3k.

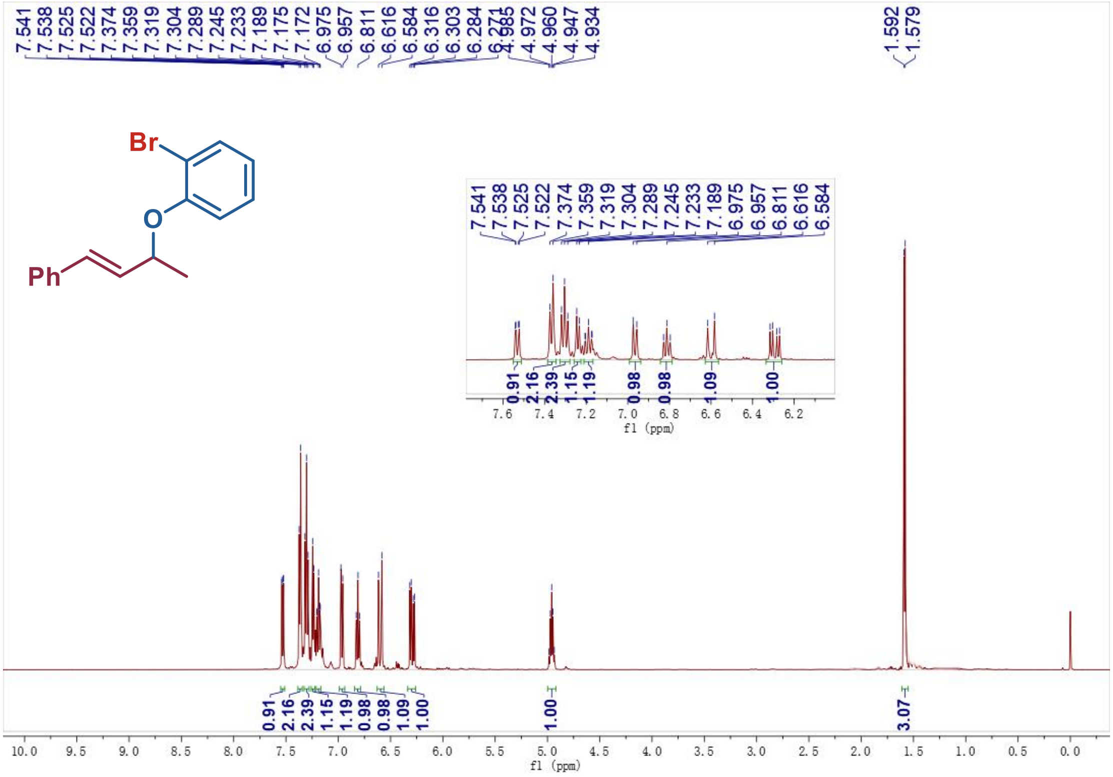

Fig. S41 ^{1}H NMR data of product 3l.

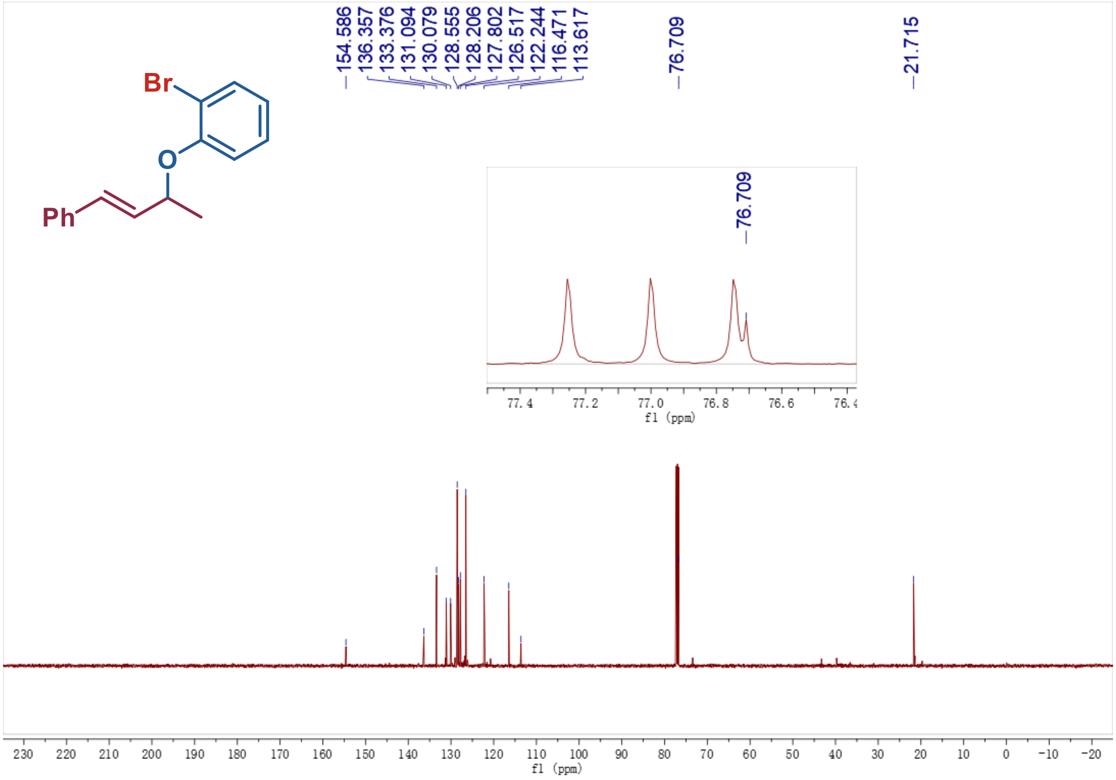

Fig. S42 ^{13}C NMR data of product 3l.

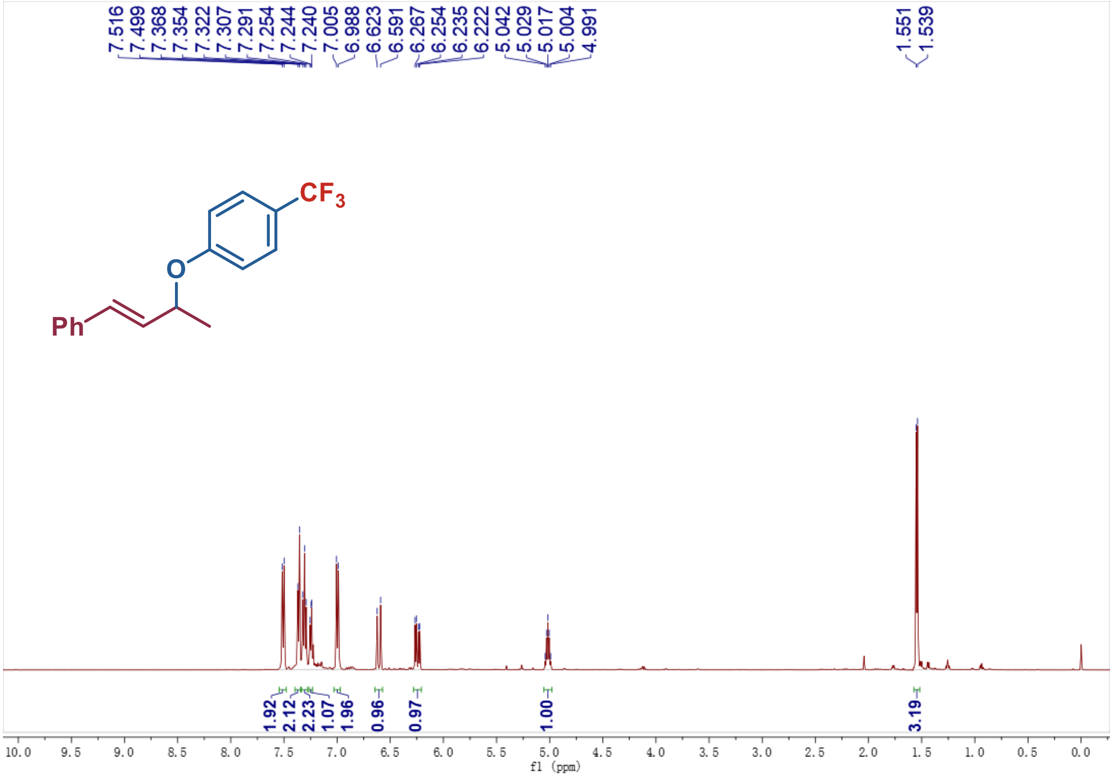

Fig. S43 ^{1}H NMR data of product 3m.

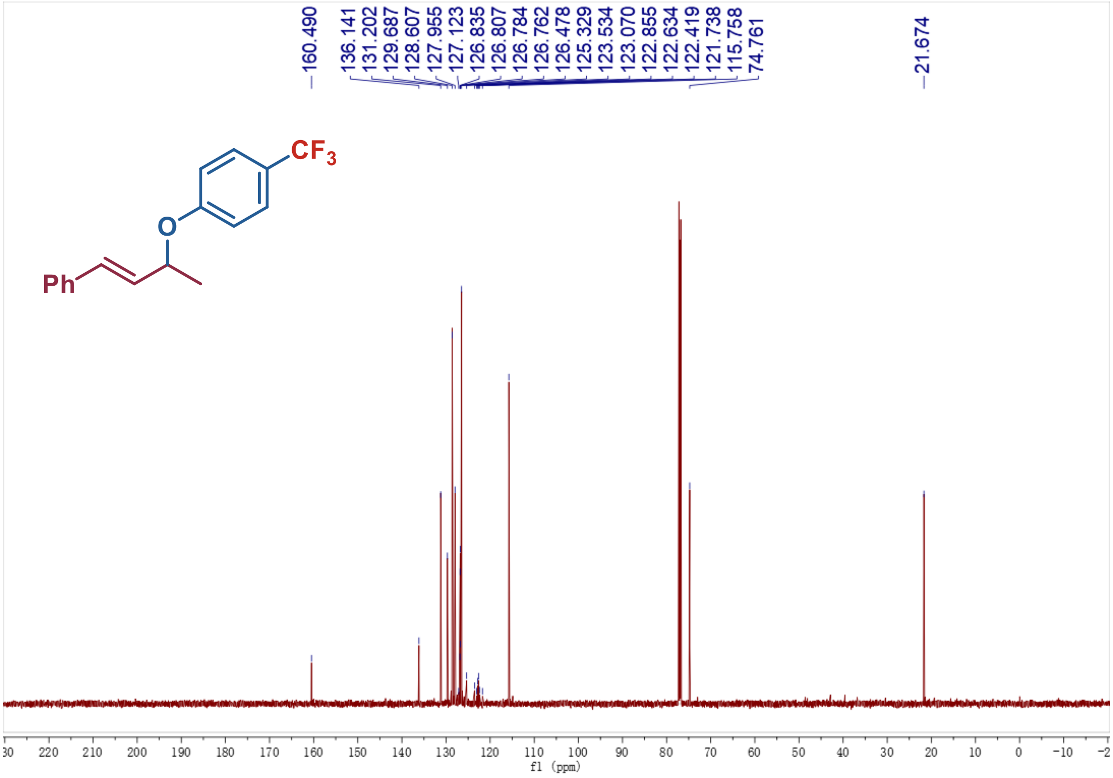

Fig. S44 ^{13}C NMR data of product 3m.

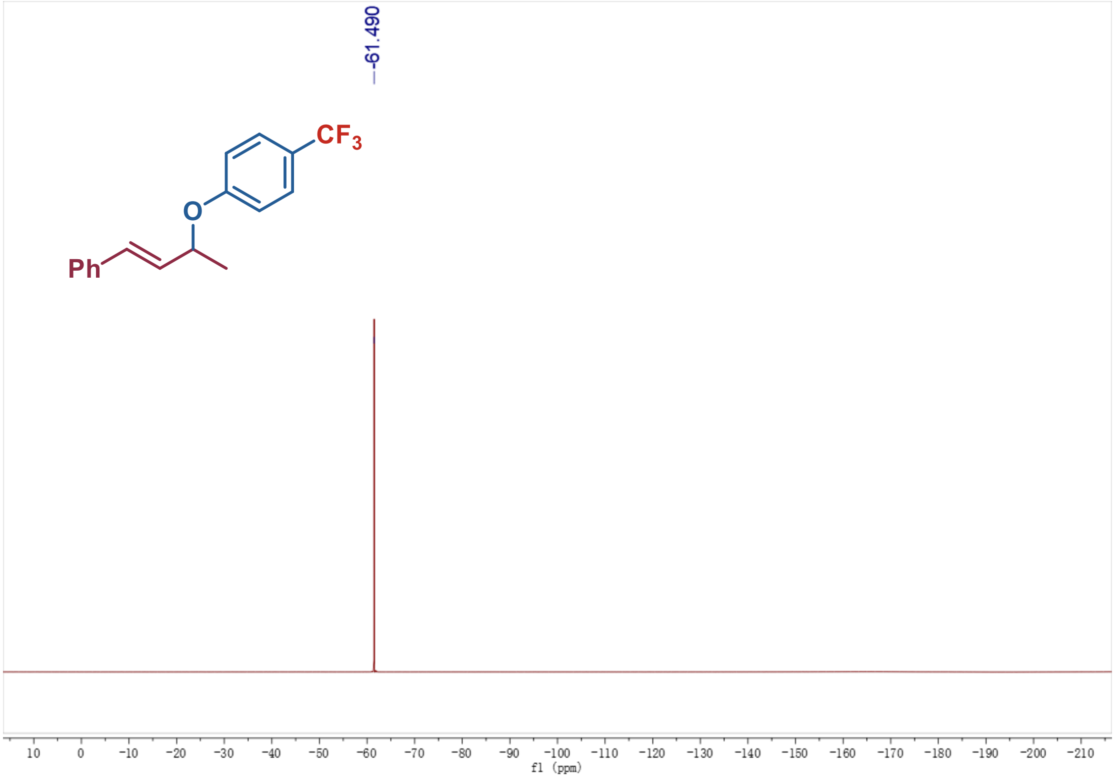

Fig. S45 ^{19}F NMR data of product 3m.

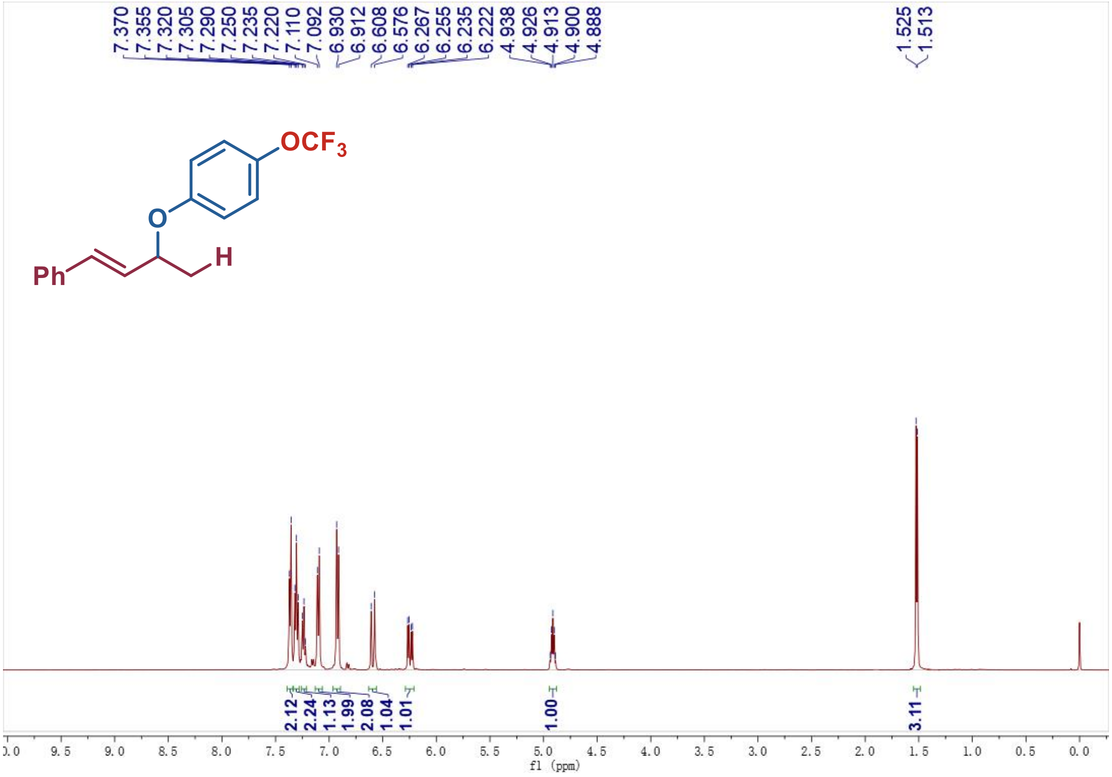

Fig. S46 ^{1}H NMR data of product 3n.

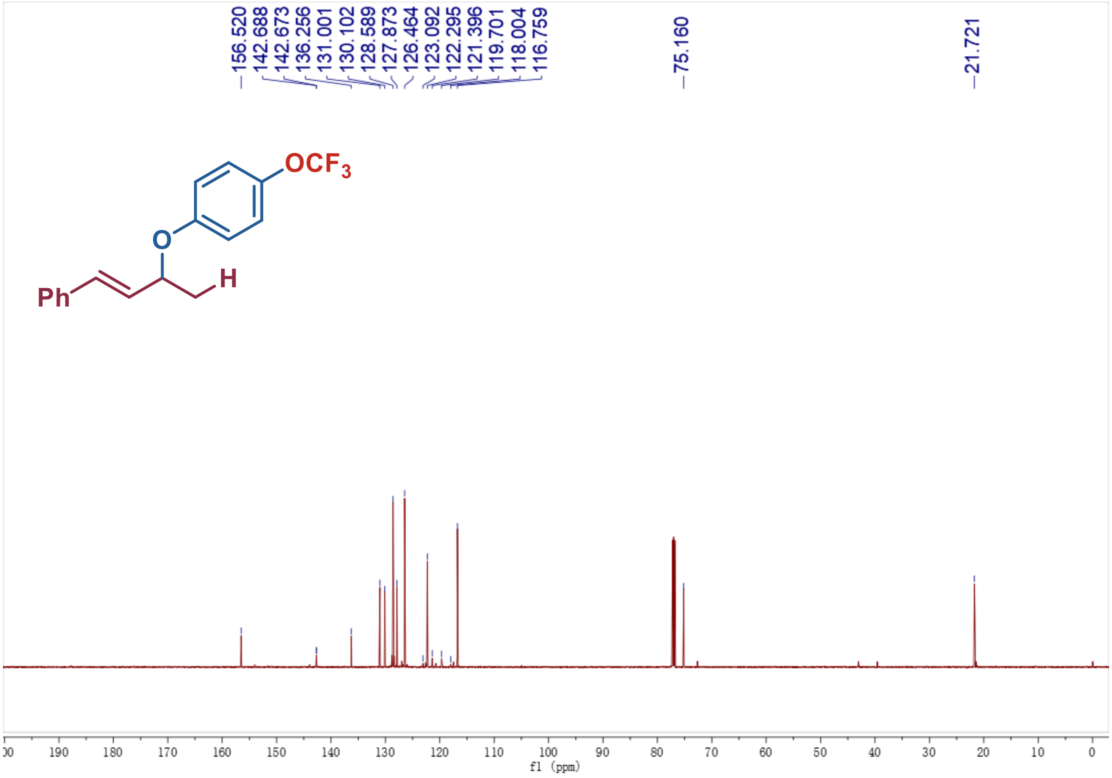

Fig. S47 ^{13}C NMR data of product 3n.

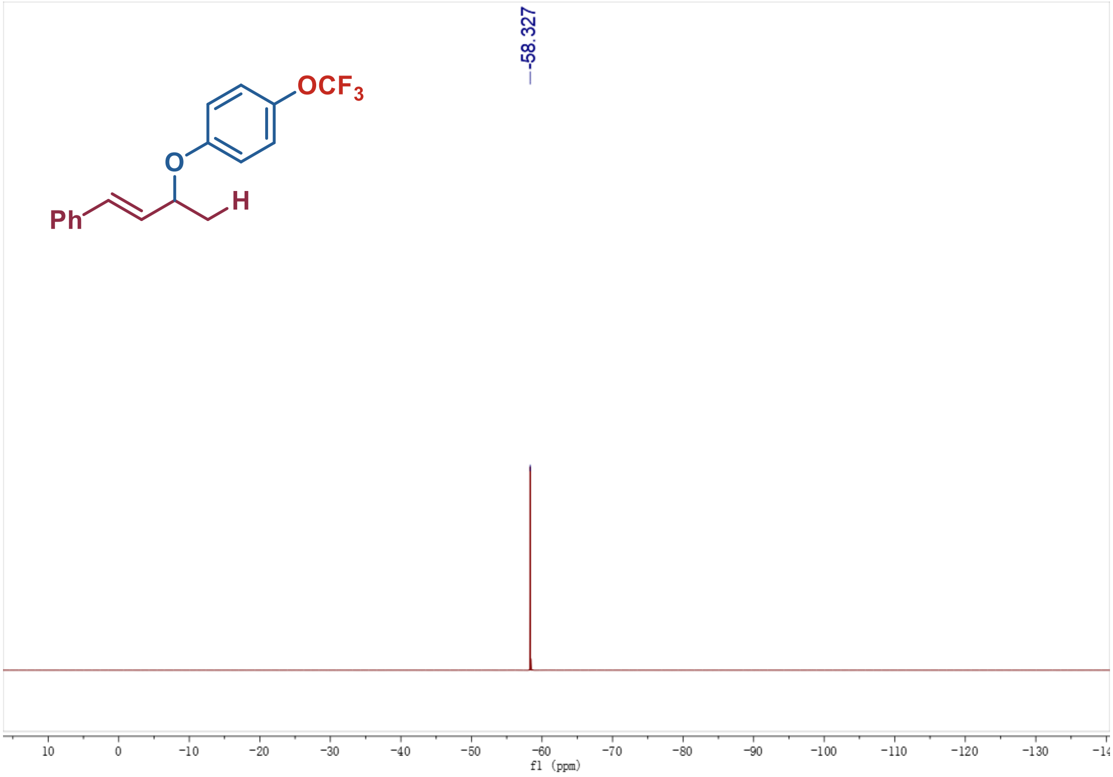

Fig. S48 ^{19}F NMR data of product 3n.

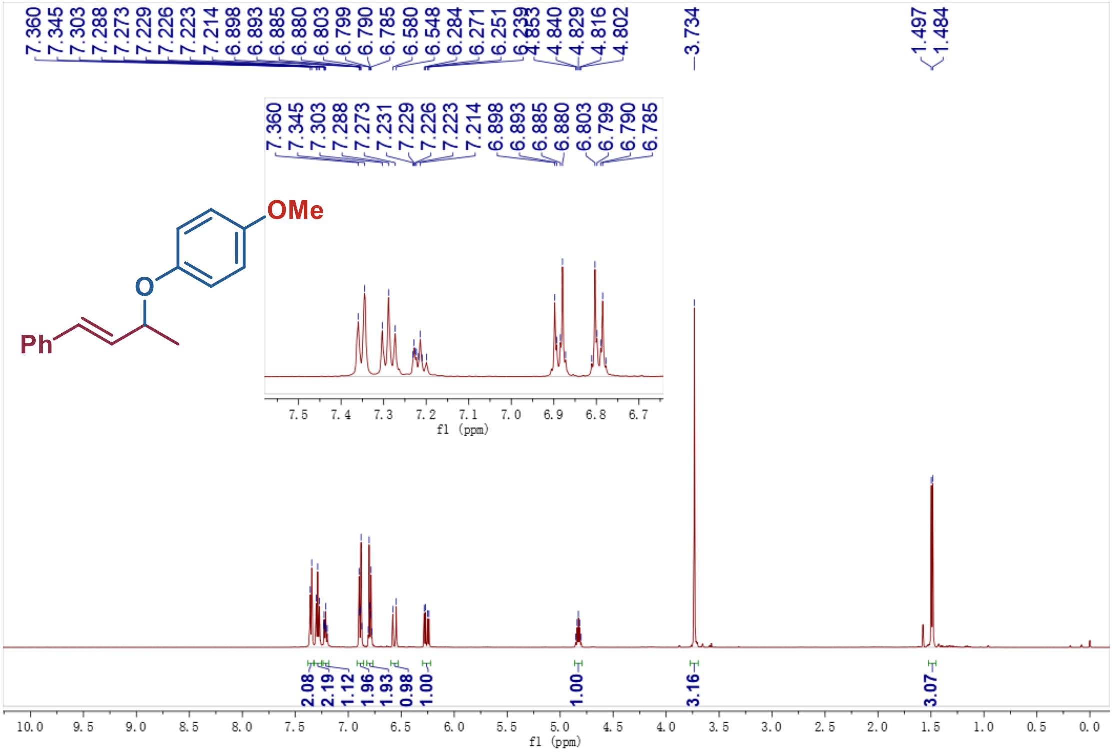

Fig. S49 ^{1}H NMR data of product 3o.

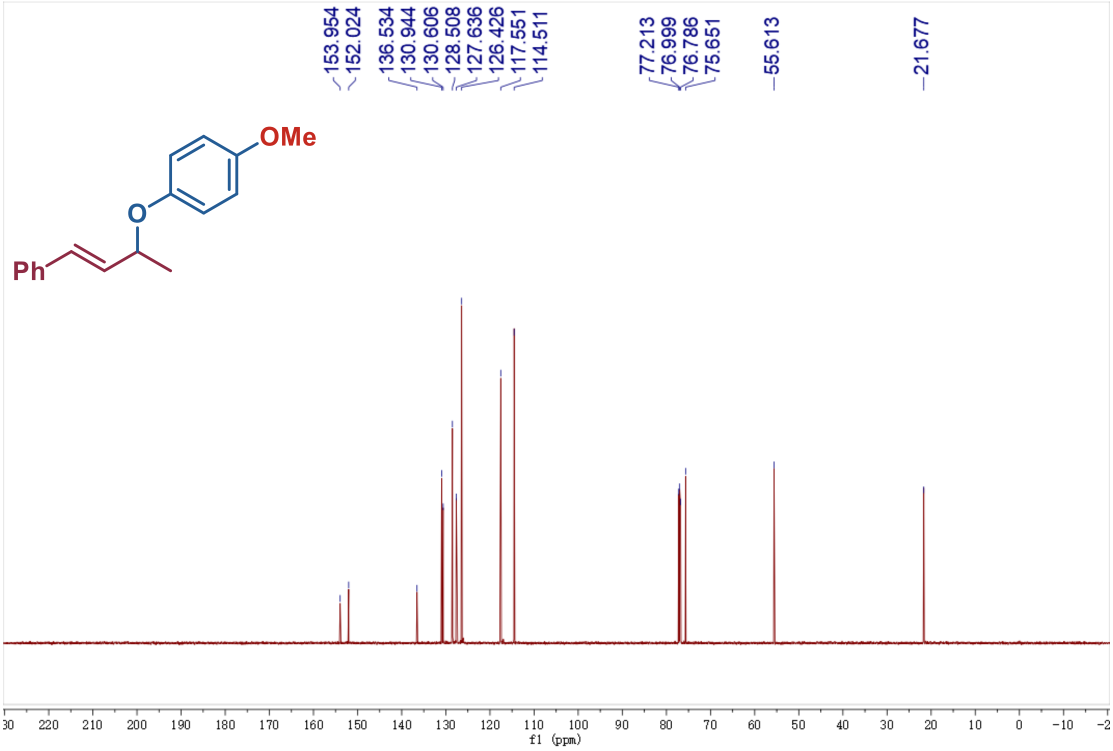

Fig. S50 ^{13}C NMR data of product 3o.

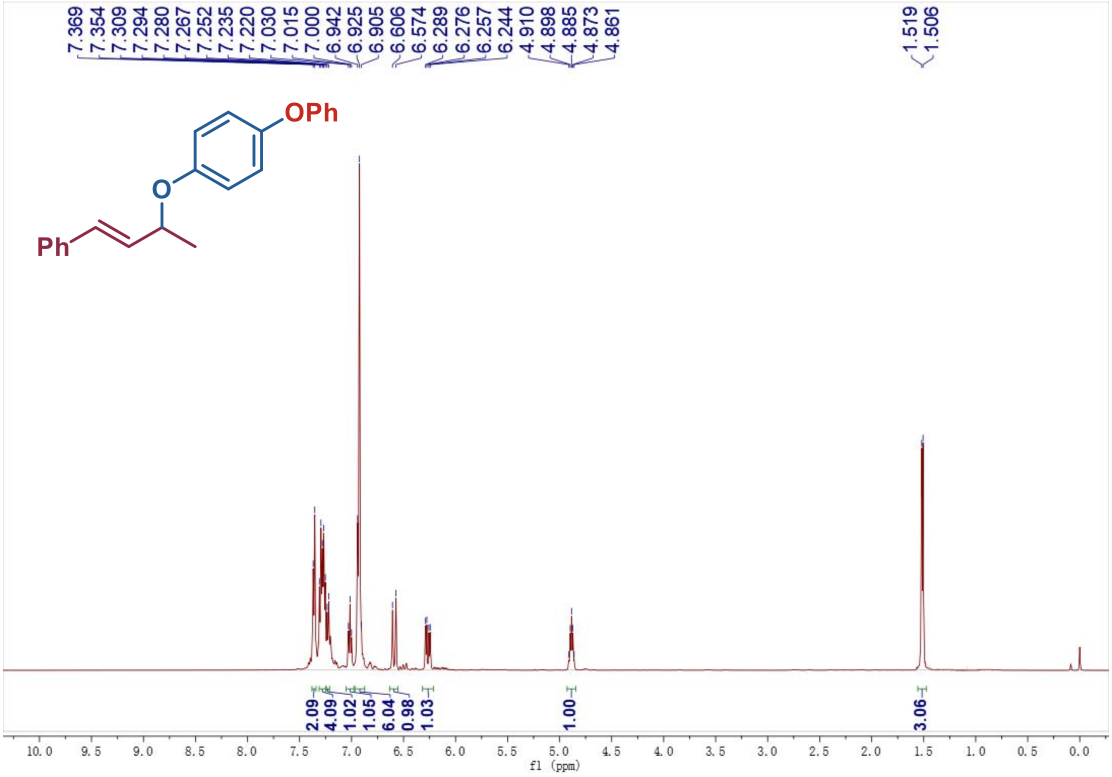

Fig. S51 ^{1}H NMR data of product 3p.

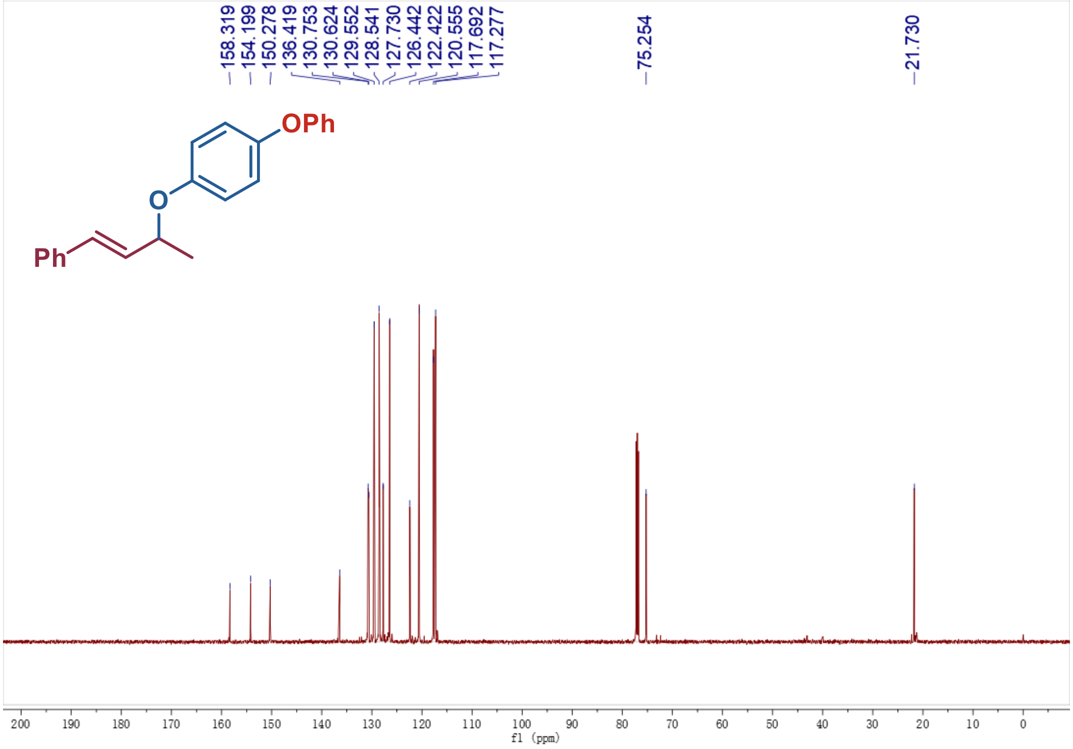

Fig. S52 ^{13}C NMR data of product 3p.

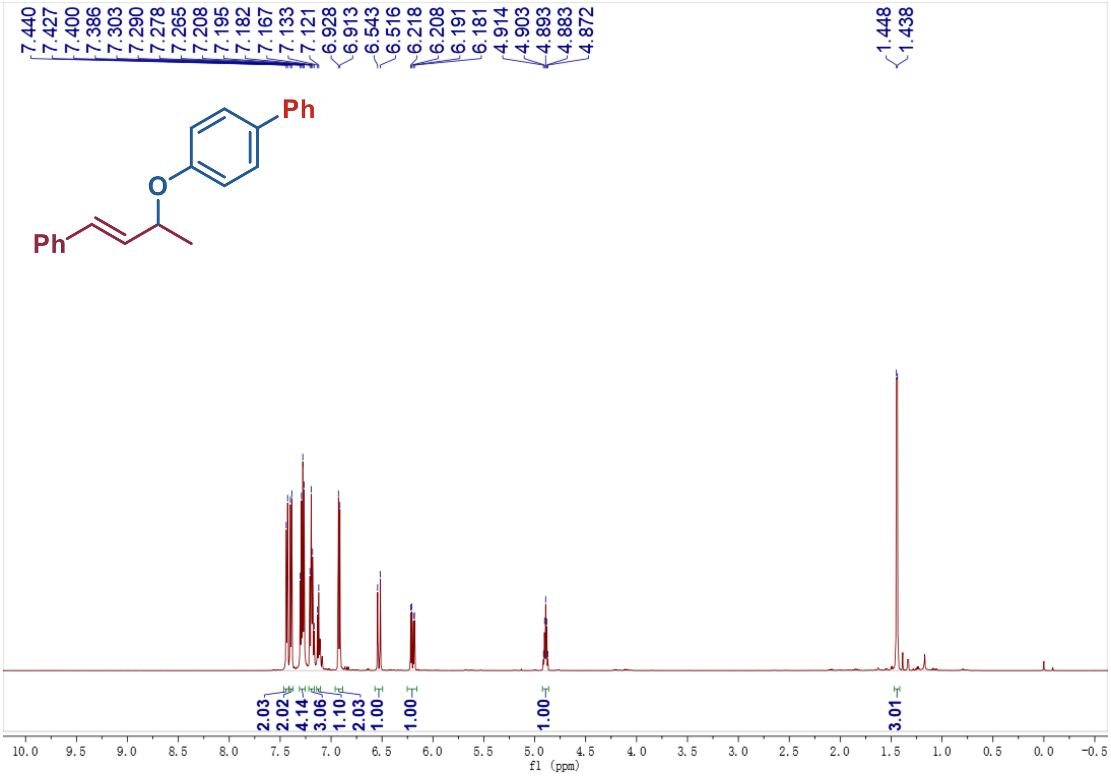

Fig. S53 ^{1}H NMR data of product 3q.

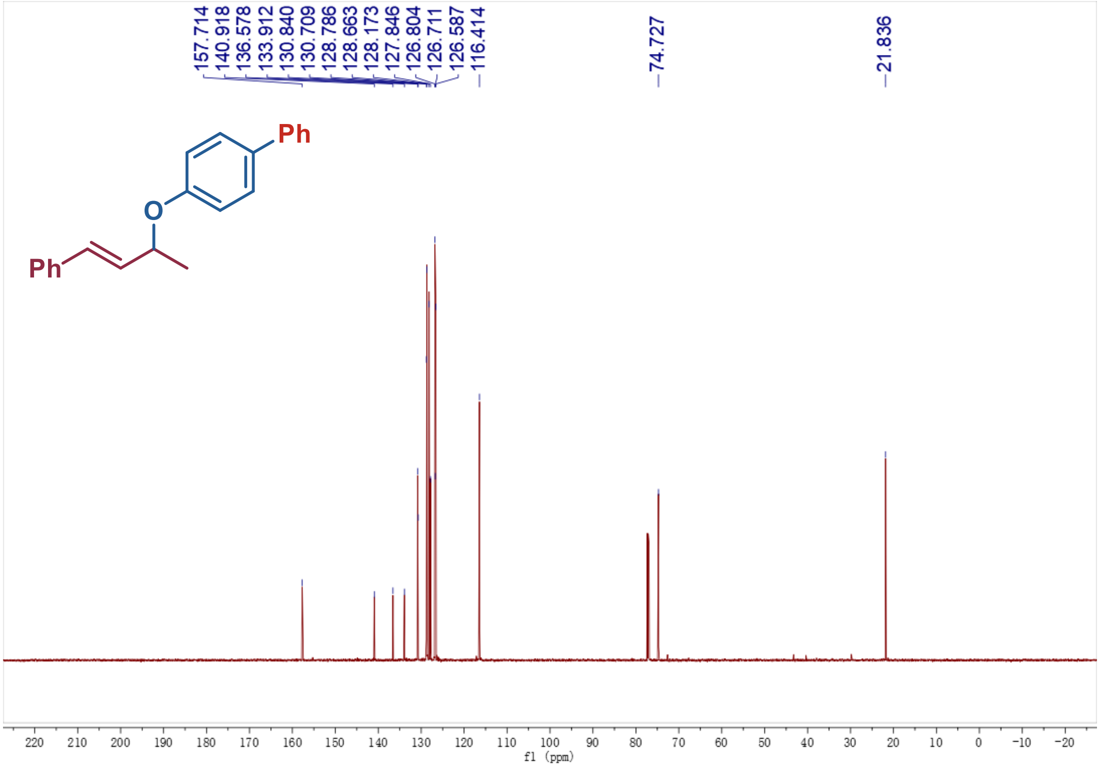

Fig. S54 ^{13}C NMR data of product 3q.

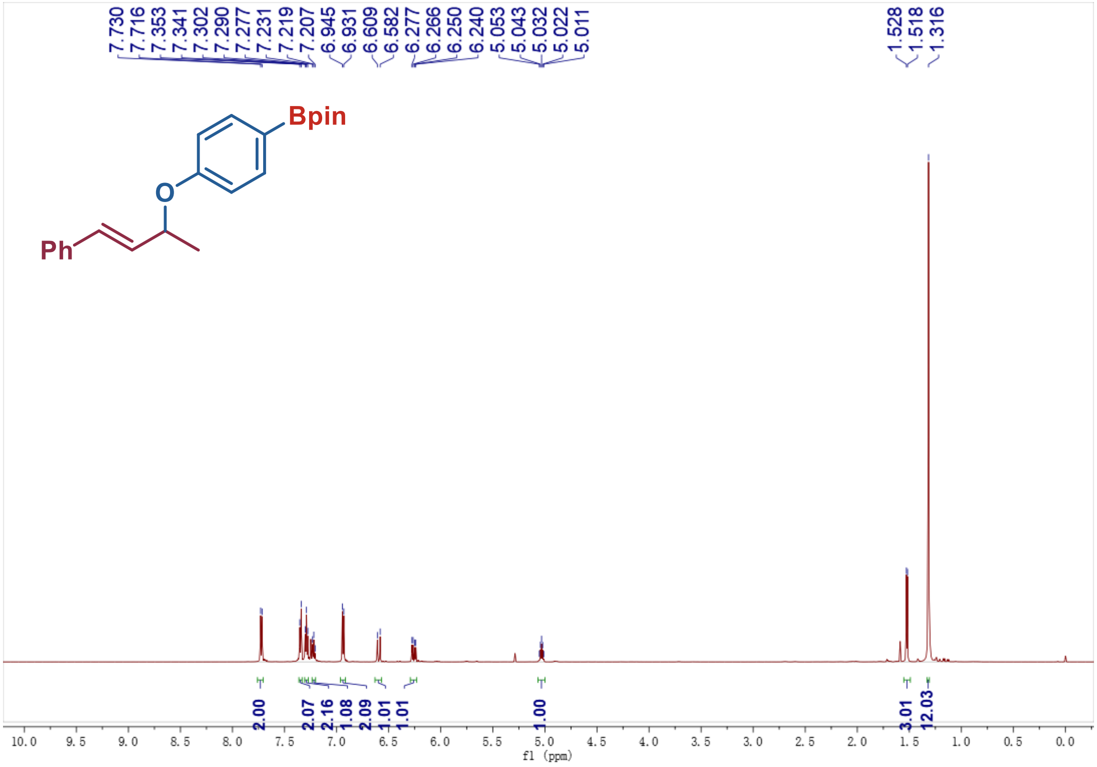

Fig. S55 ^{1}H NMR data of product 3r.

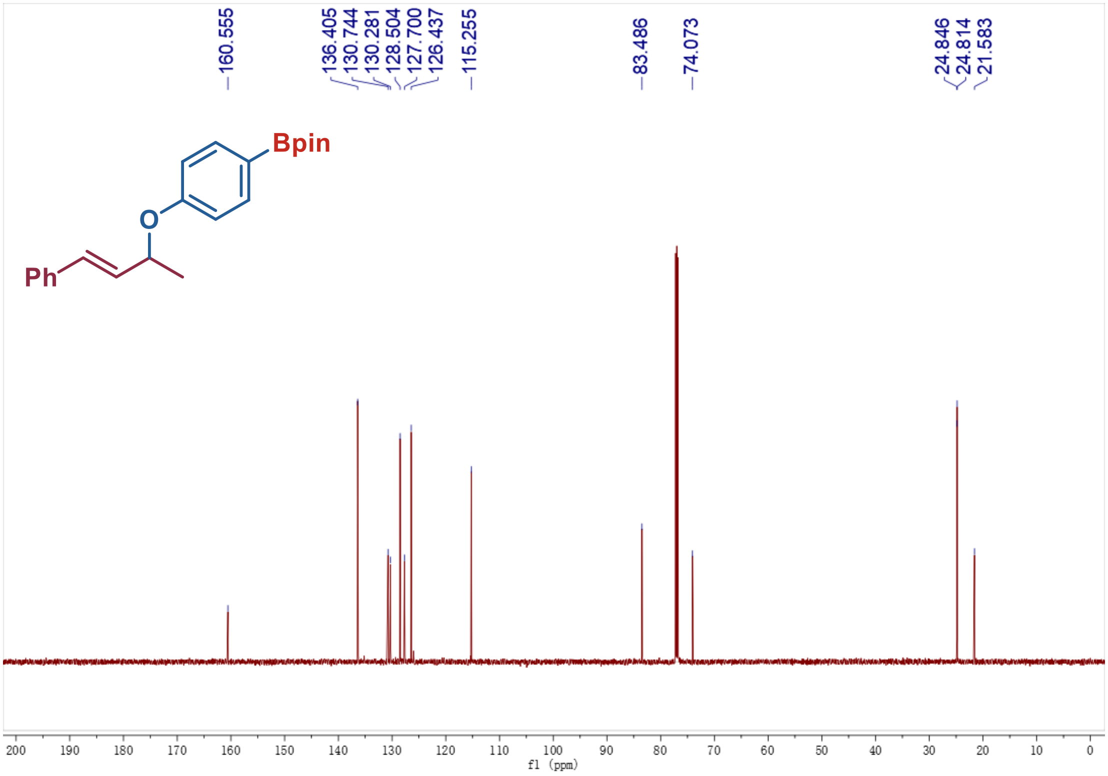

Fig. S56 ^{13}C NMR data of product 3r.

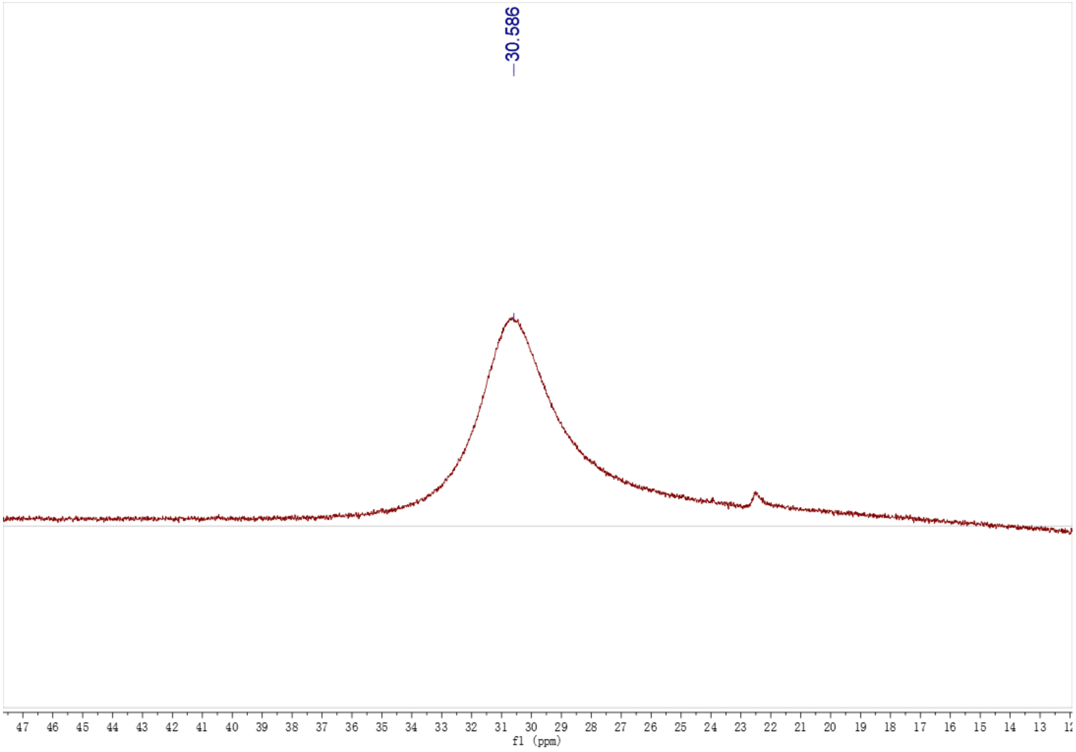

Fig. S57 ^{11}B NMR data of product 3r.

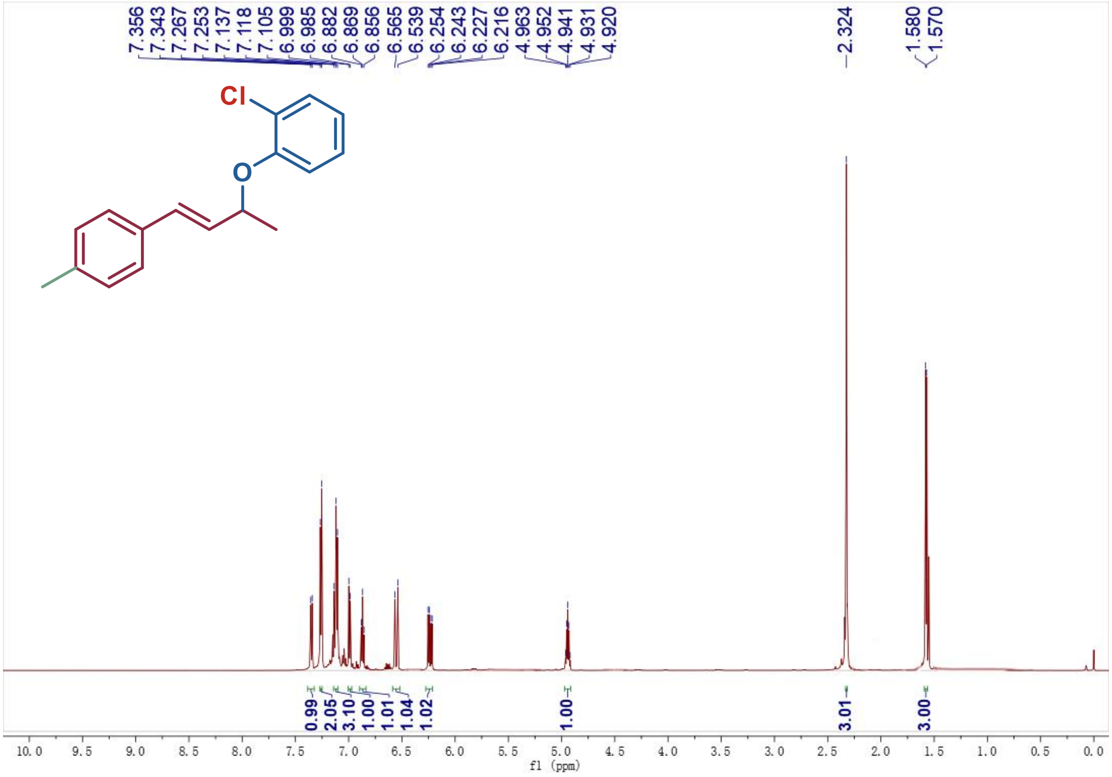

Fig. S58 ^{1}H NMR data of product 3s.

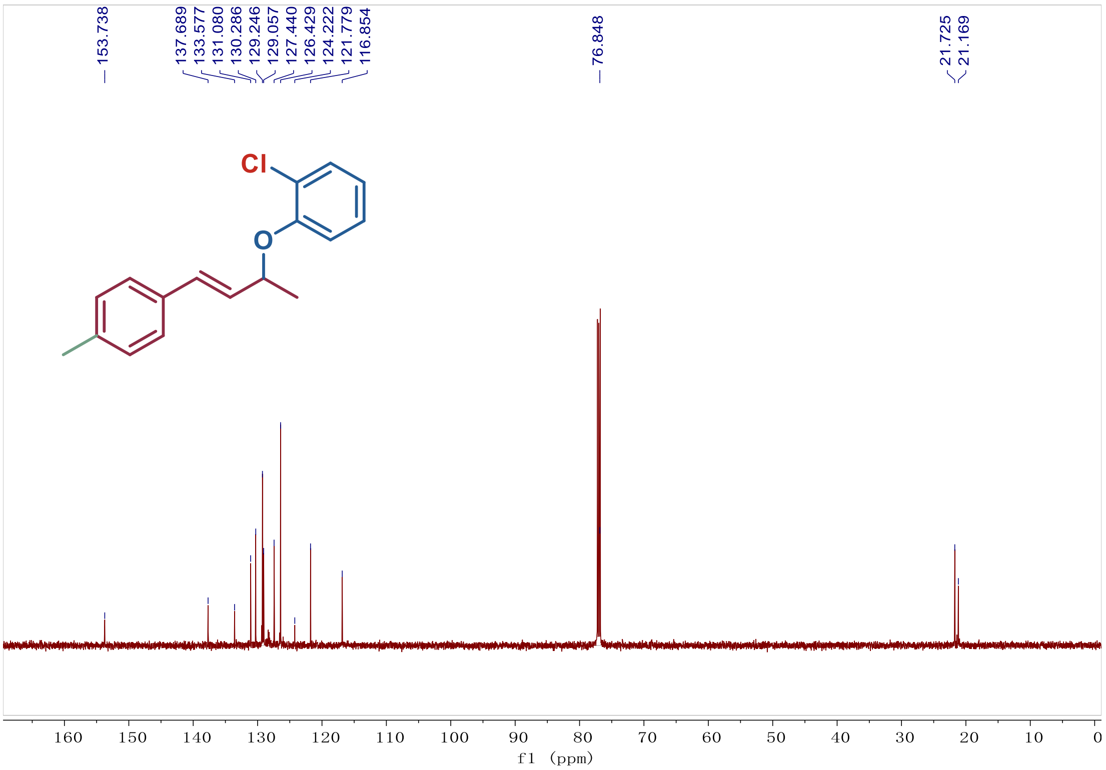

Fig. S59 ^{13}C NMR data of product 3s.

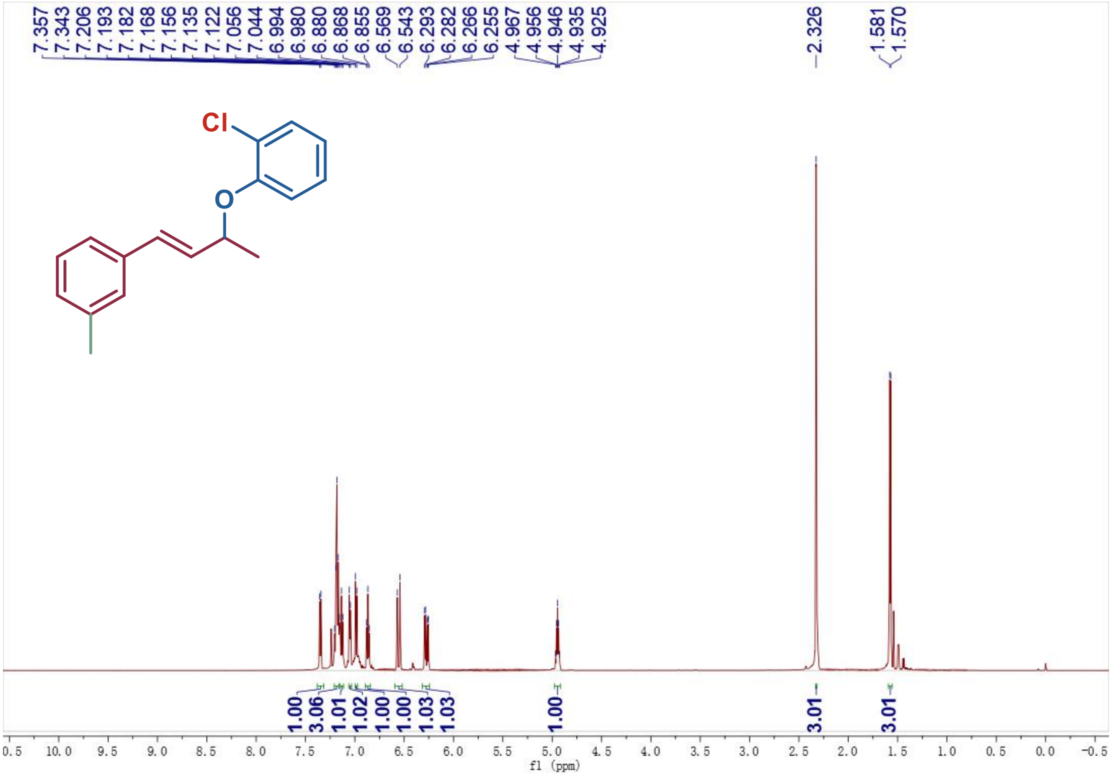

Fig. S60 ^{1}H NMR data of product 3t.

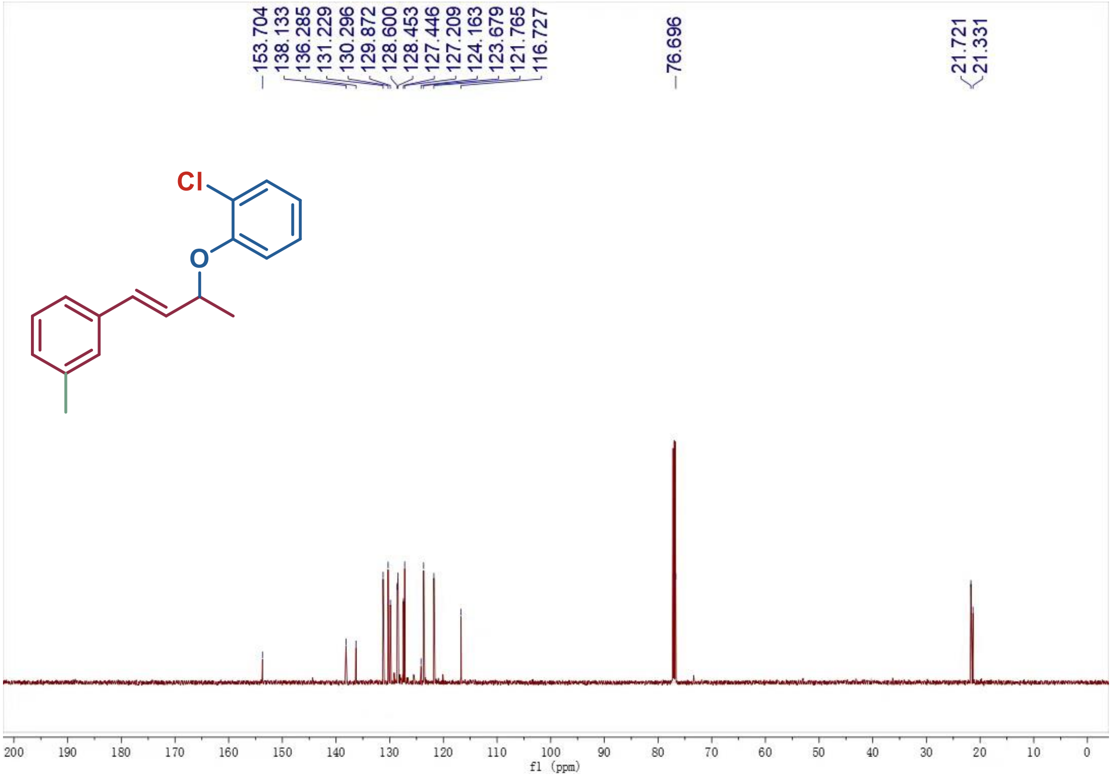

Fig. S61 ^{13}C NMR data of product 3t.

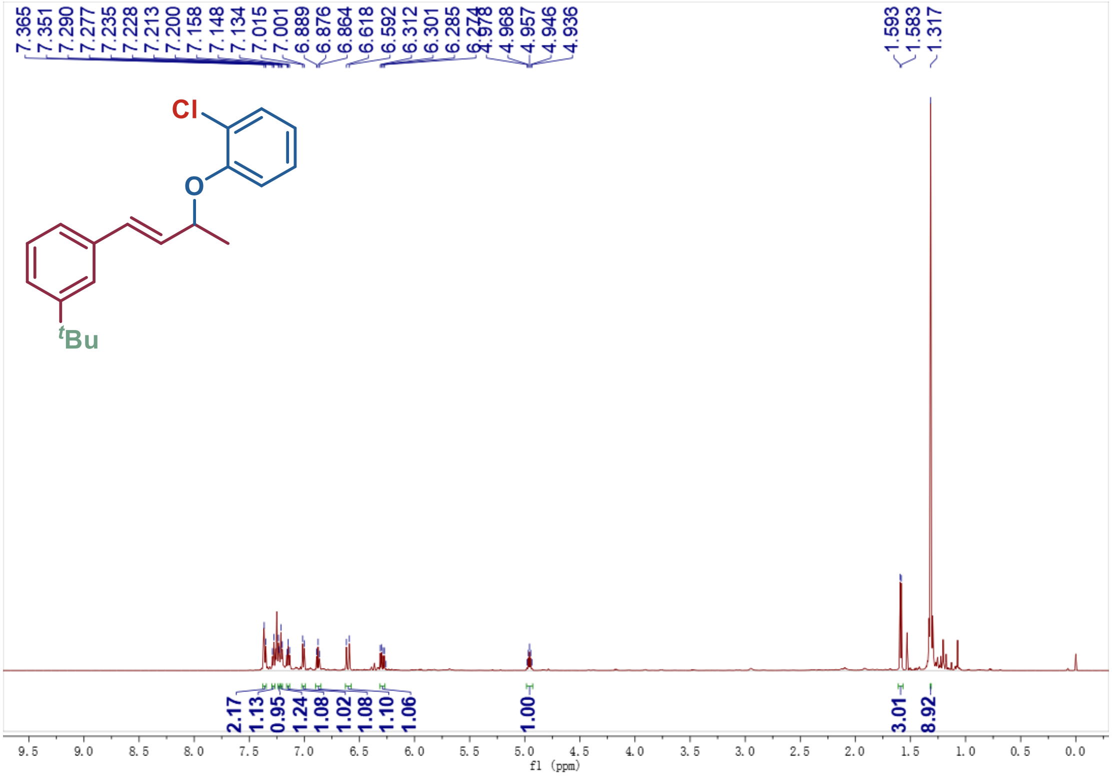

Fig. S62 ^{1}H NMR data of product 3u.

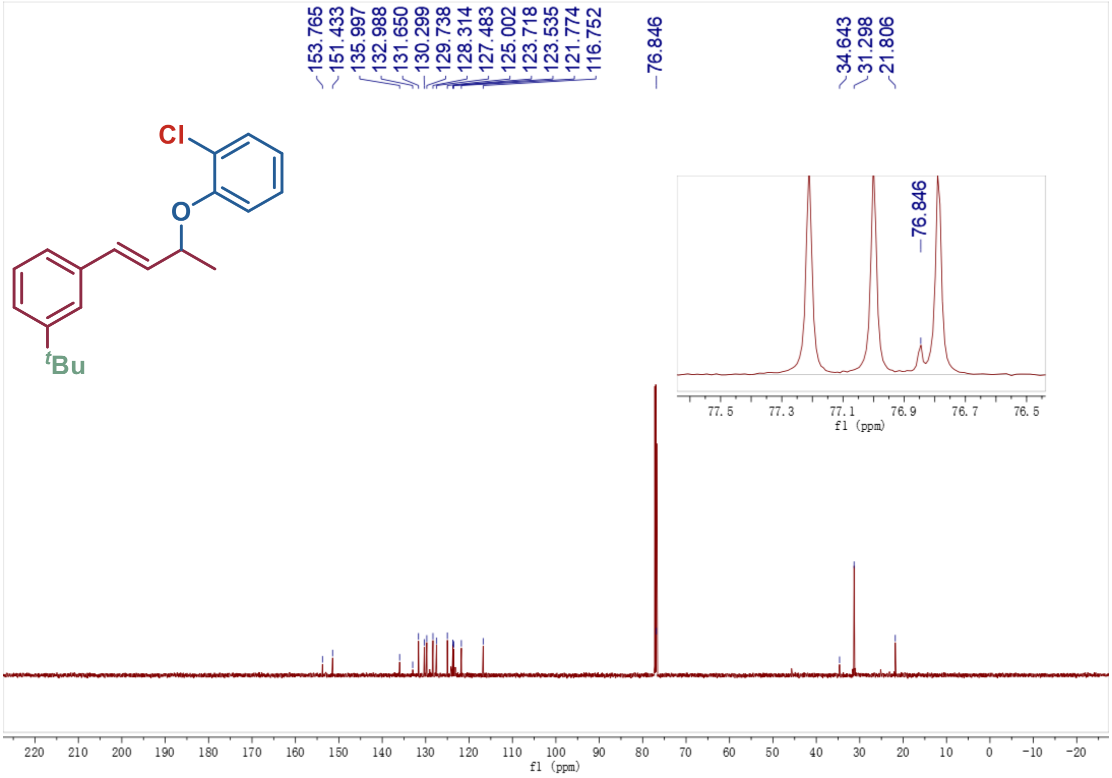

Fig. S 63 ^{13}C NMR data of product 3u.

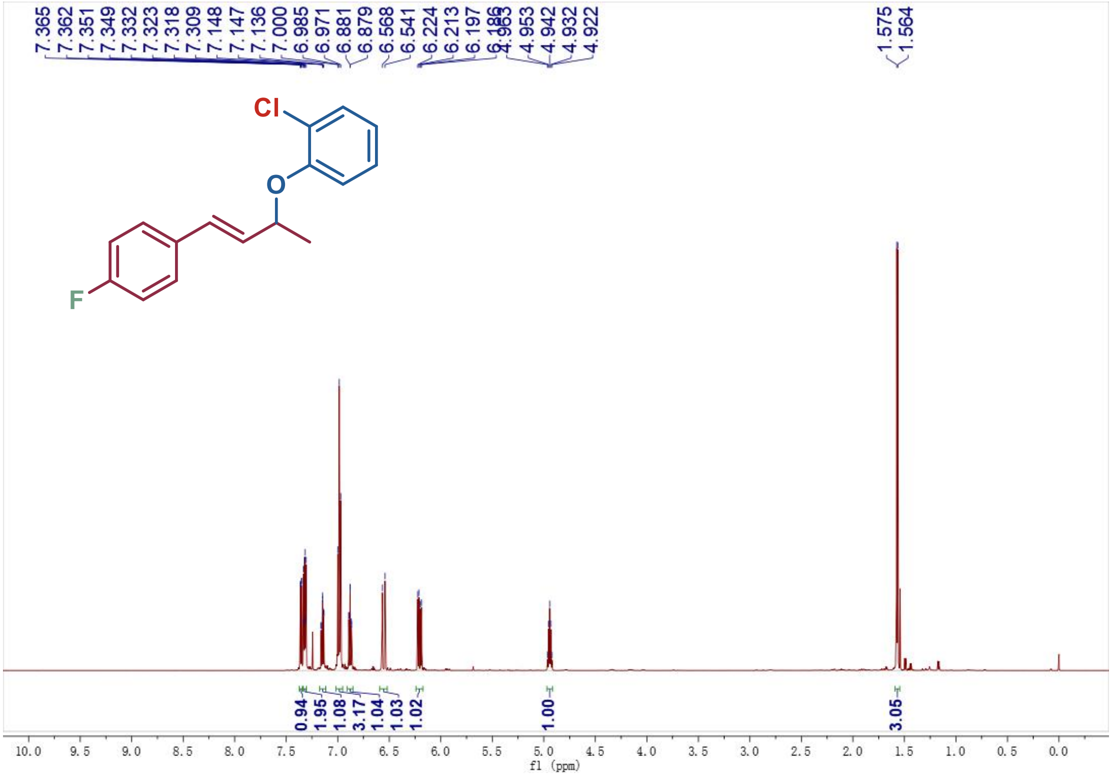

Fig. S64 ^{1}H NMR data of product 3v.

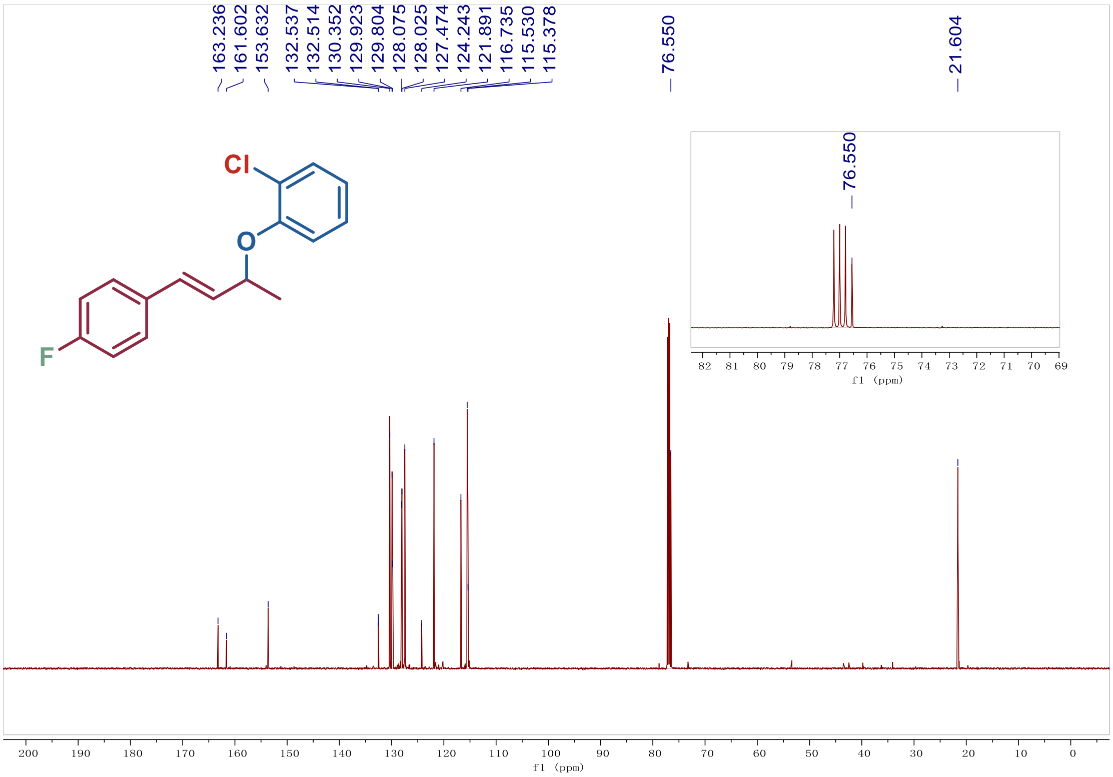

Fig. S65 ^{13}C NMR data of product 3v.

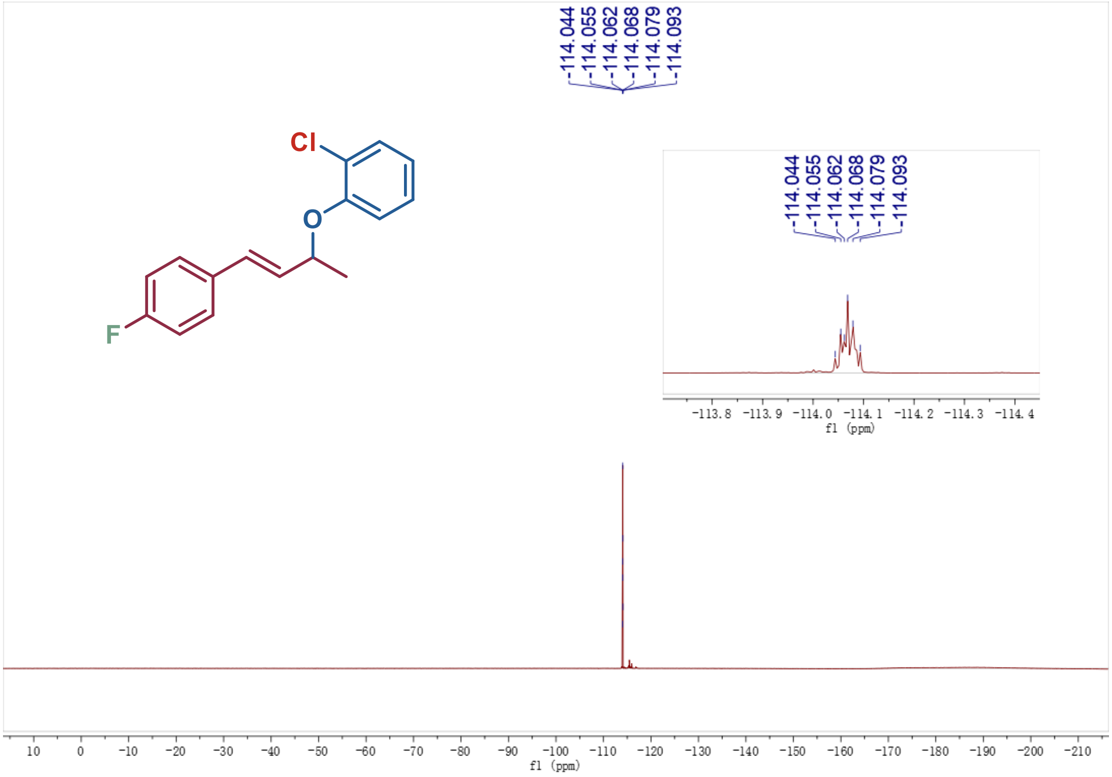

Fig. S66 ^{19}F NMR data of product 3v.

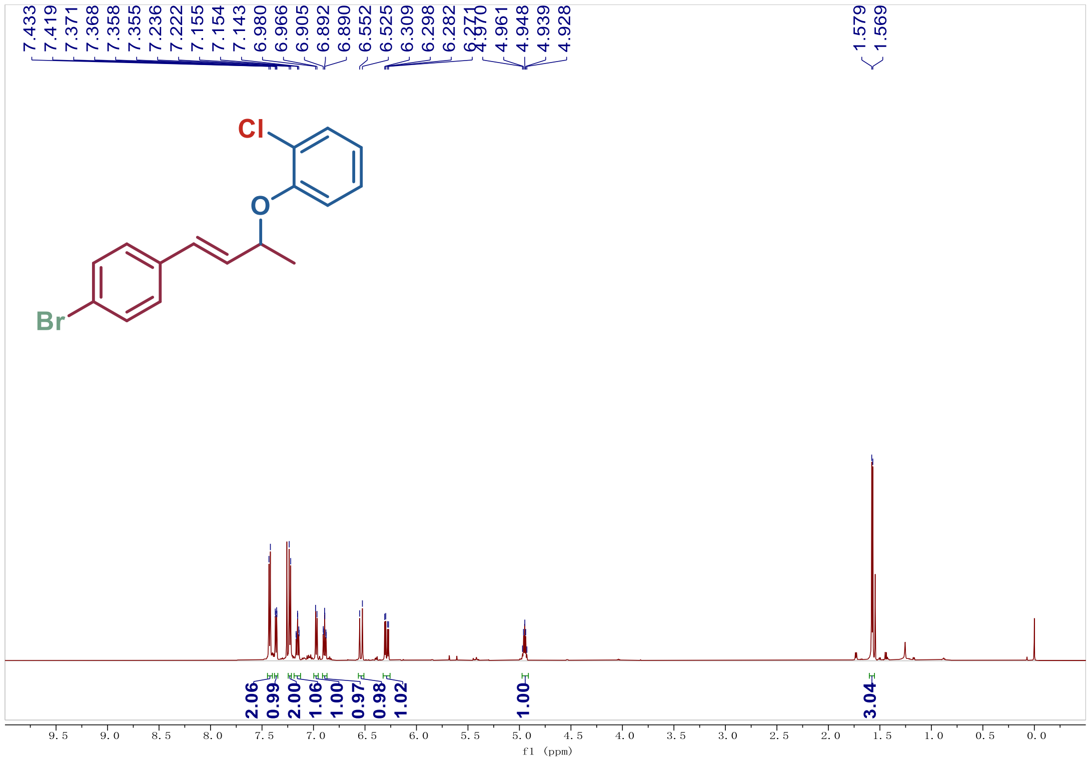

Fig. S67 ^{1}H NMR data of product 3w.

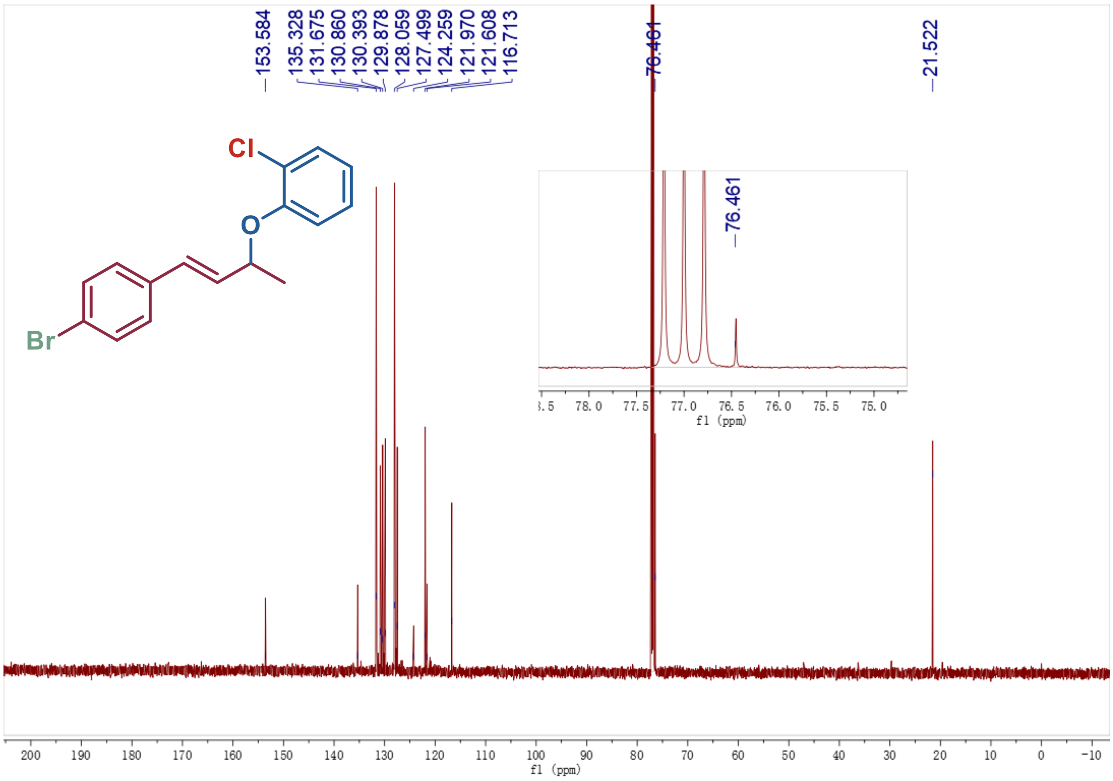

Fig. S68 ^{13}C NMR data of product 3w.

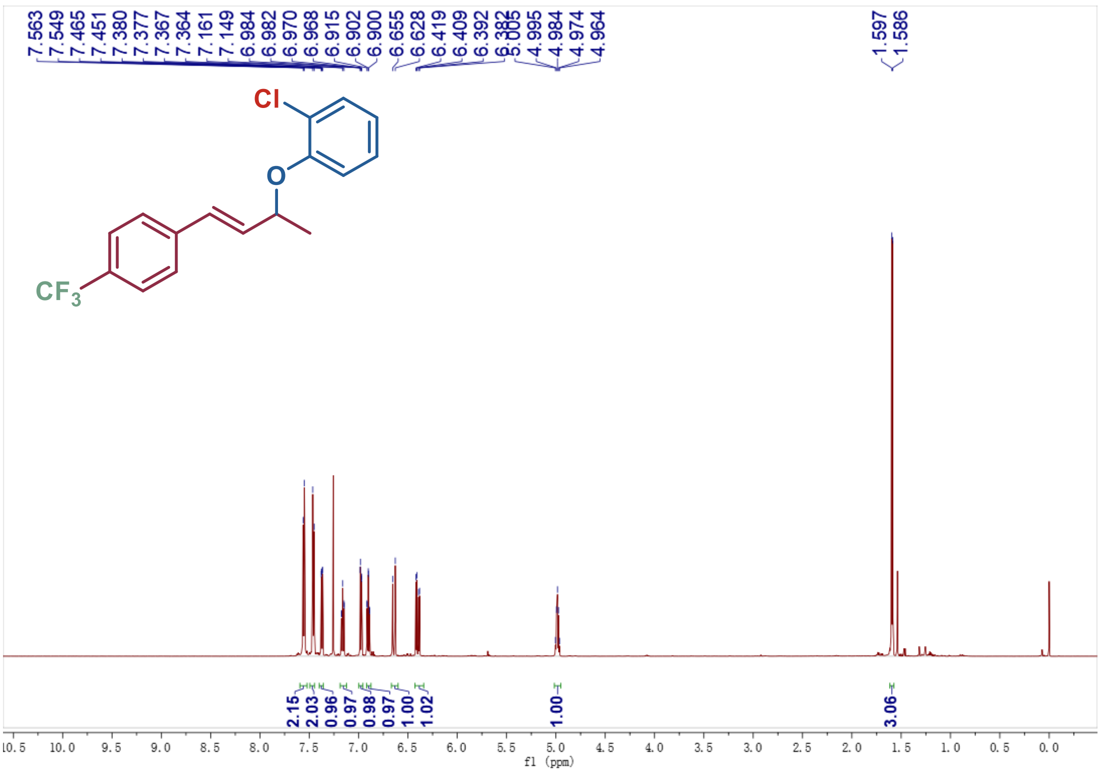

Fig. S69 ^{1}H NMR data of product 3x.

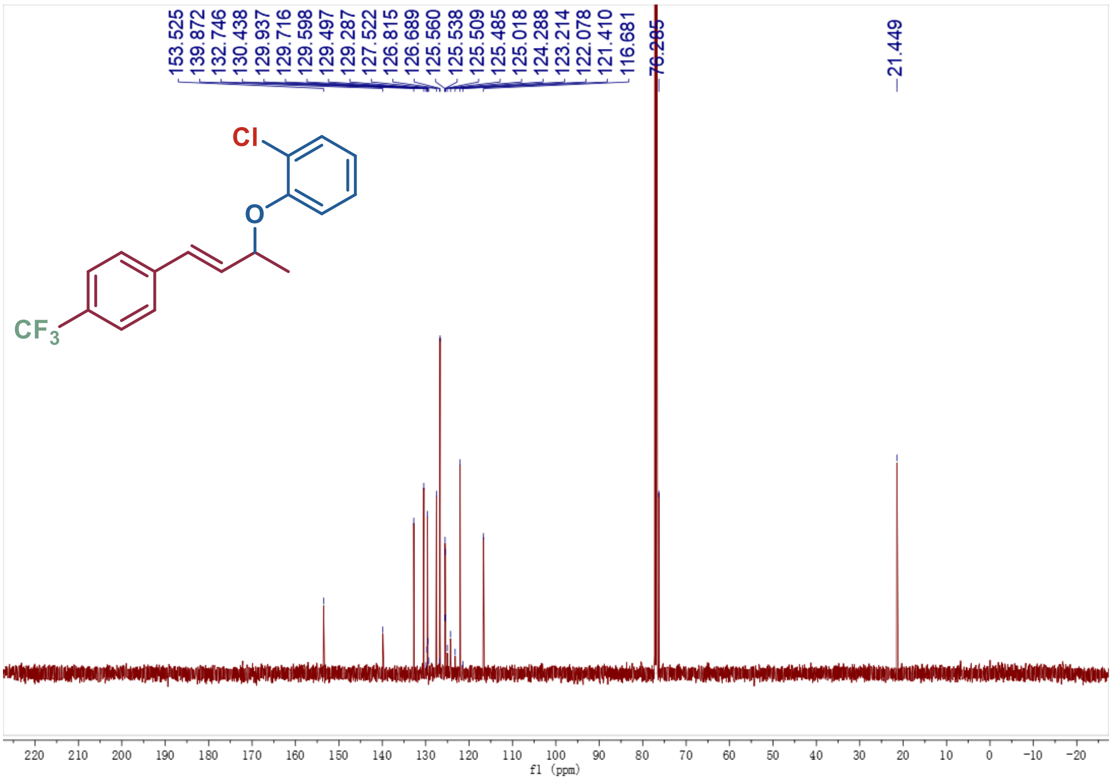

Fig. S70 ^{13}C NMR data of product 3x.

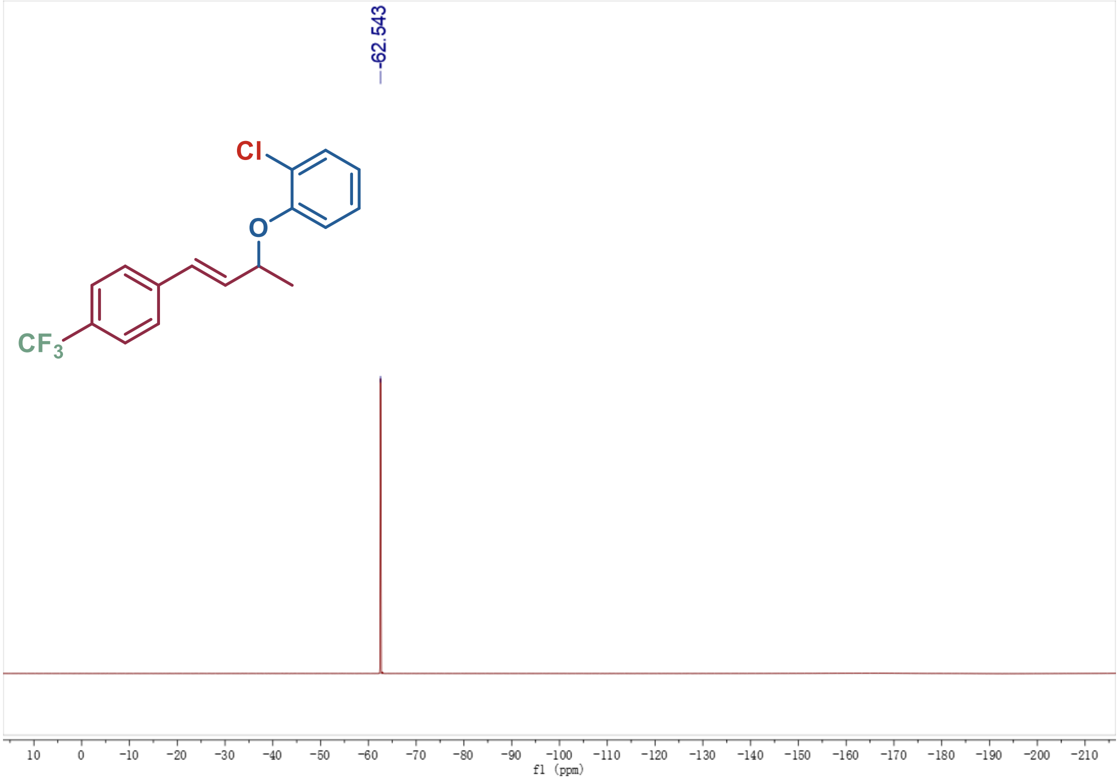

Fig. S71 ^{19}F NMR data of product 3x.

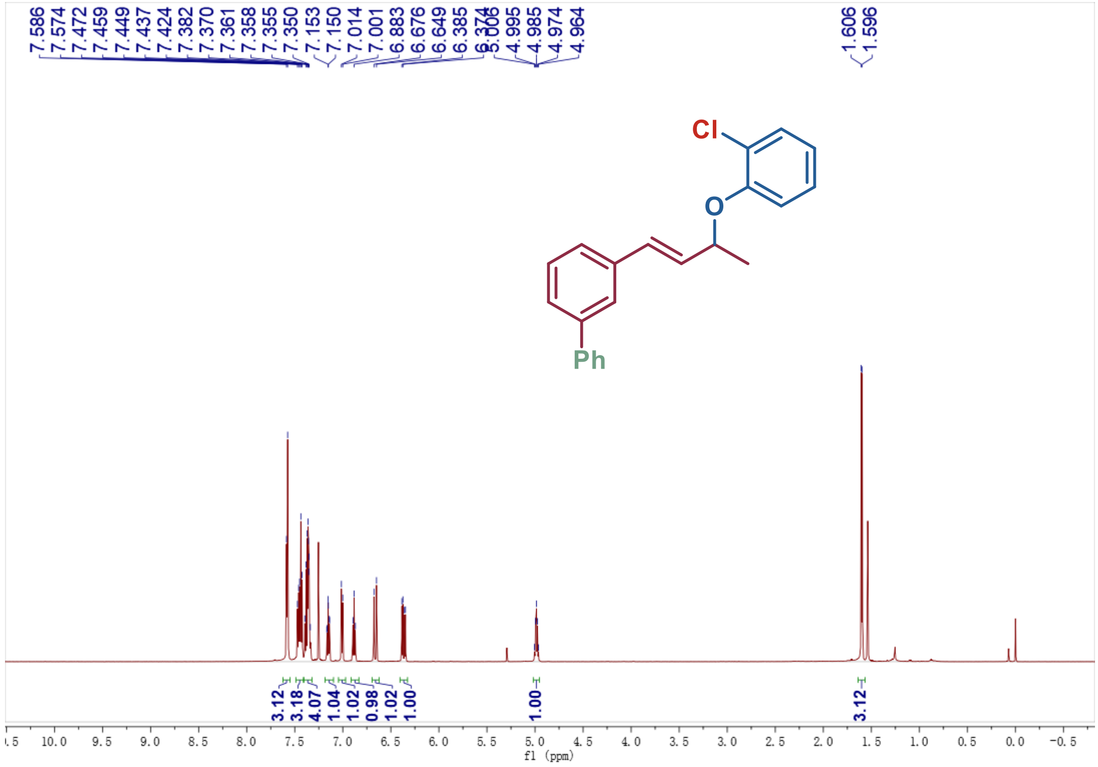

Fig. S72 ^{1}H NMR data of product 3y.

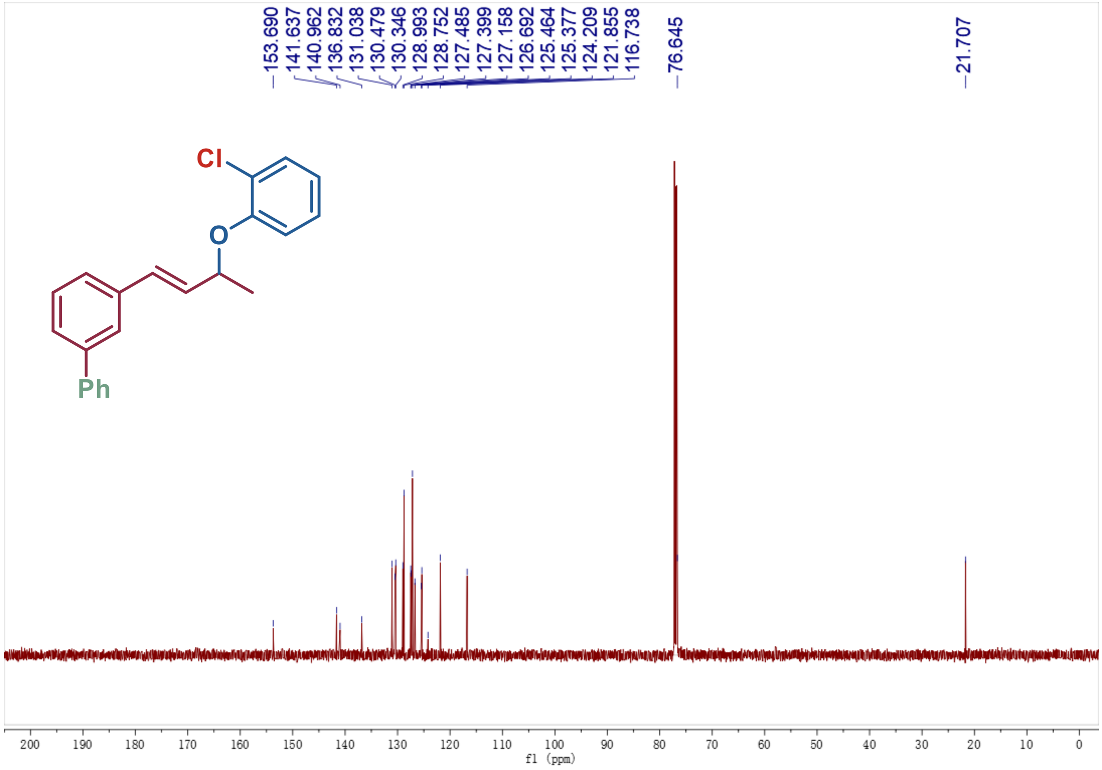

Fig. S73 ^{13}C NMR data of product 3y.

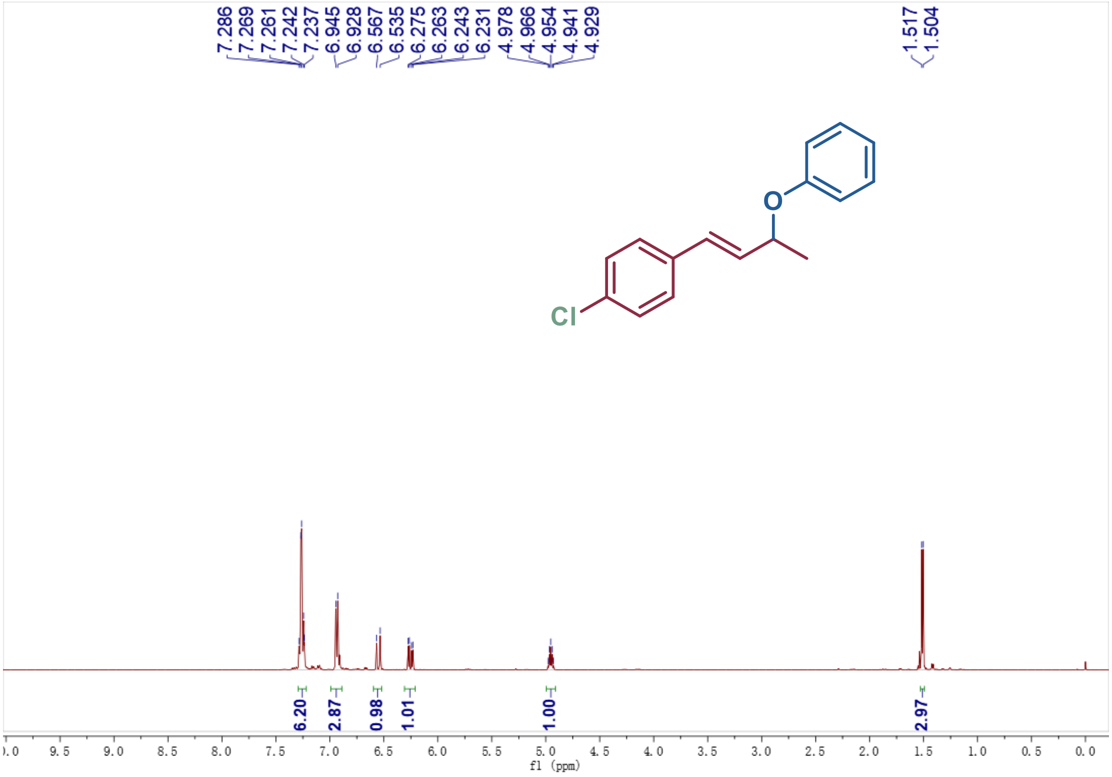

Fig. S74 ^{1}H NMR data of product 3z.

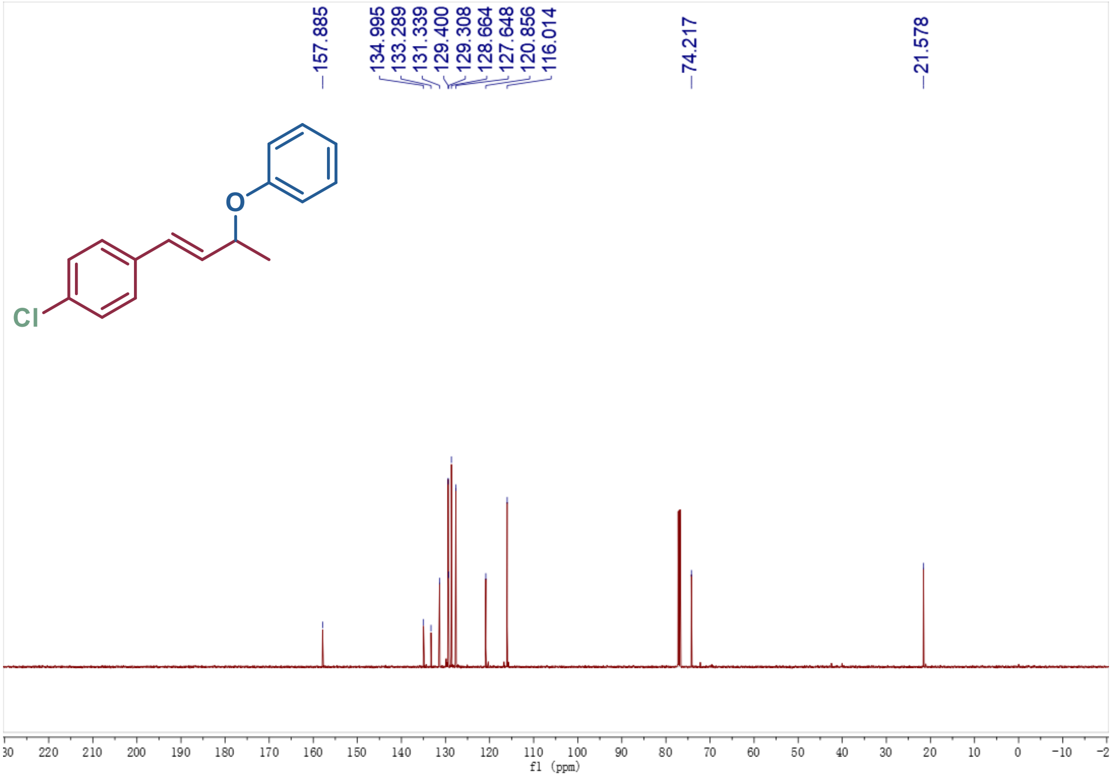

Fig. S75 ^{13}C NMR data of product 3z.

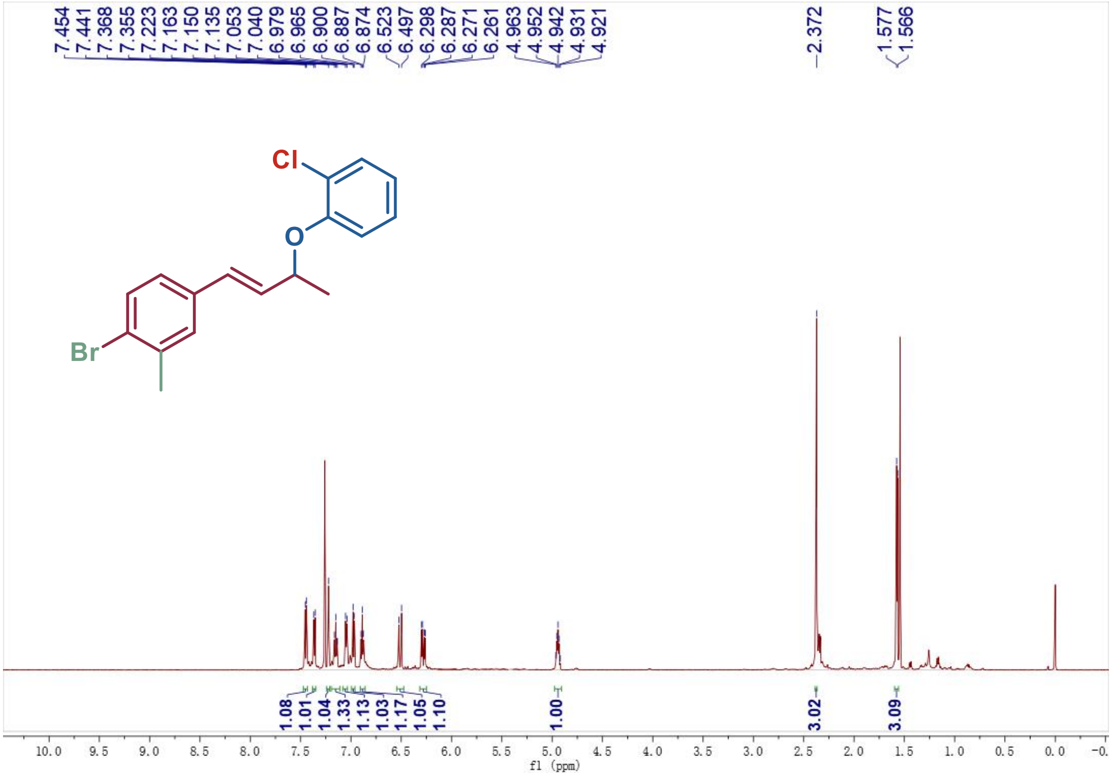

Fig. S76 ^{1}H NMR data of product 3aa.

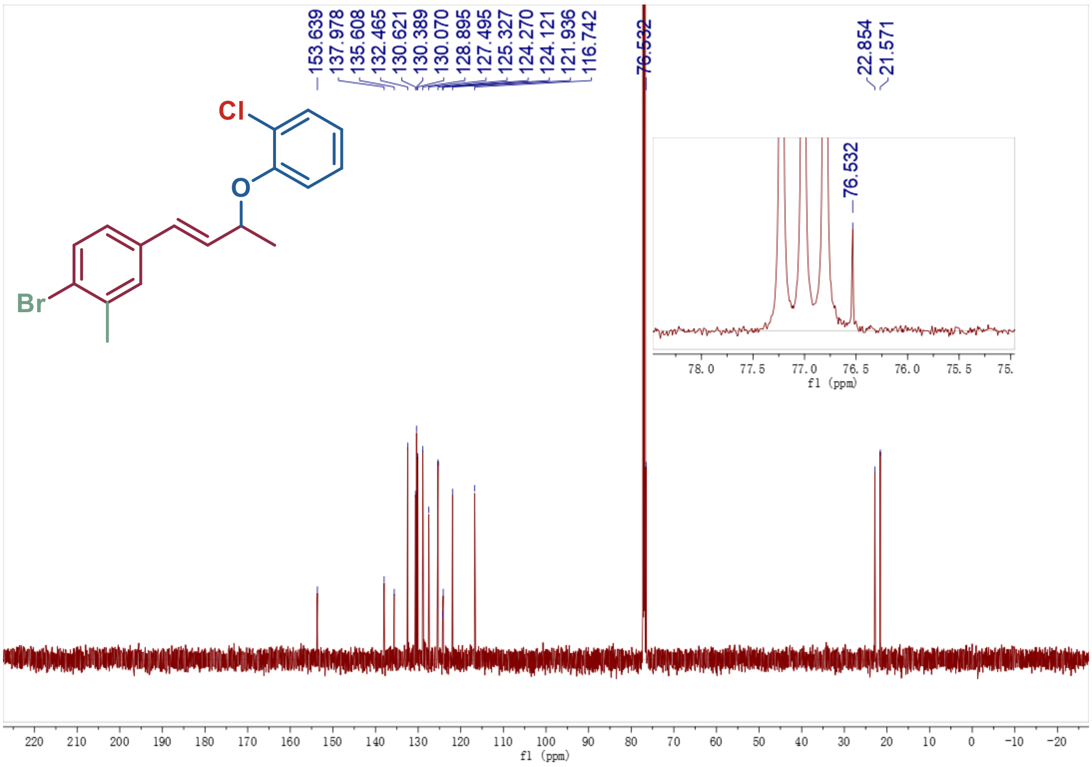

Fig. S77 ^{13}C NMR data of product 3aa.

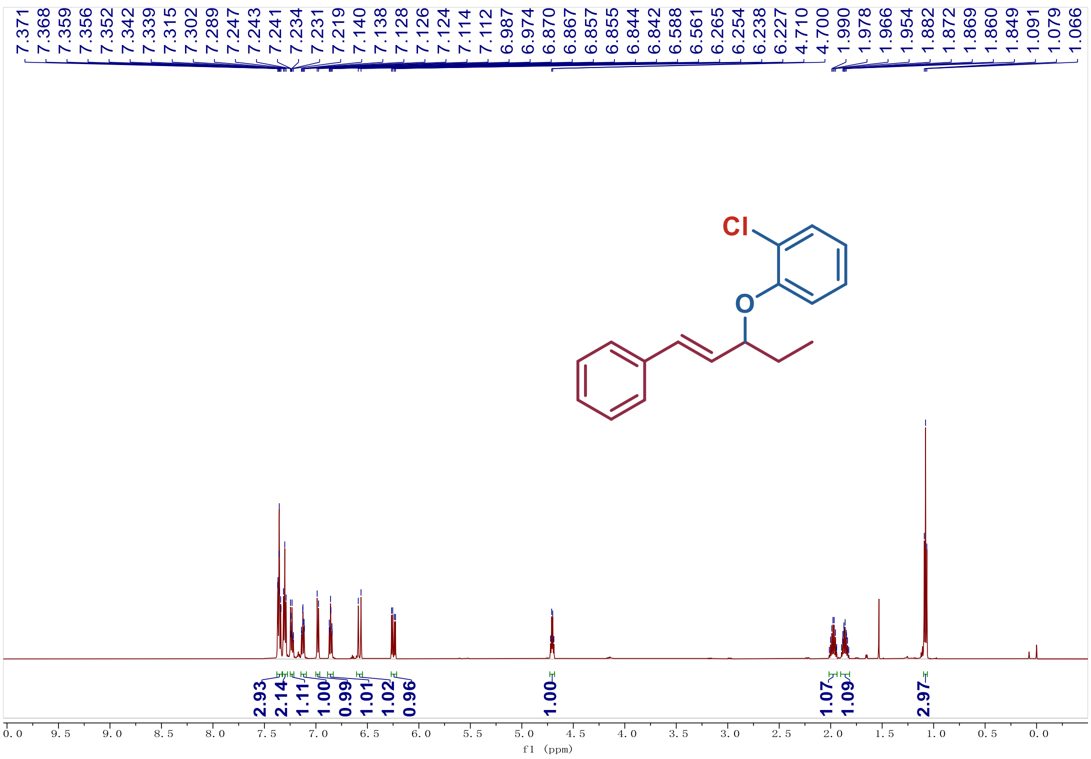

Fig. S78 ^{1}H NMR data of product 3ab.

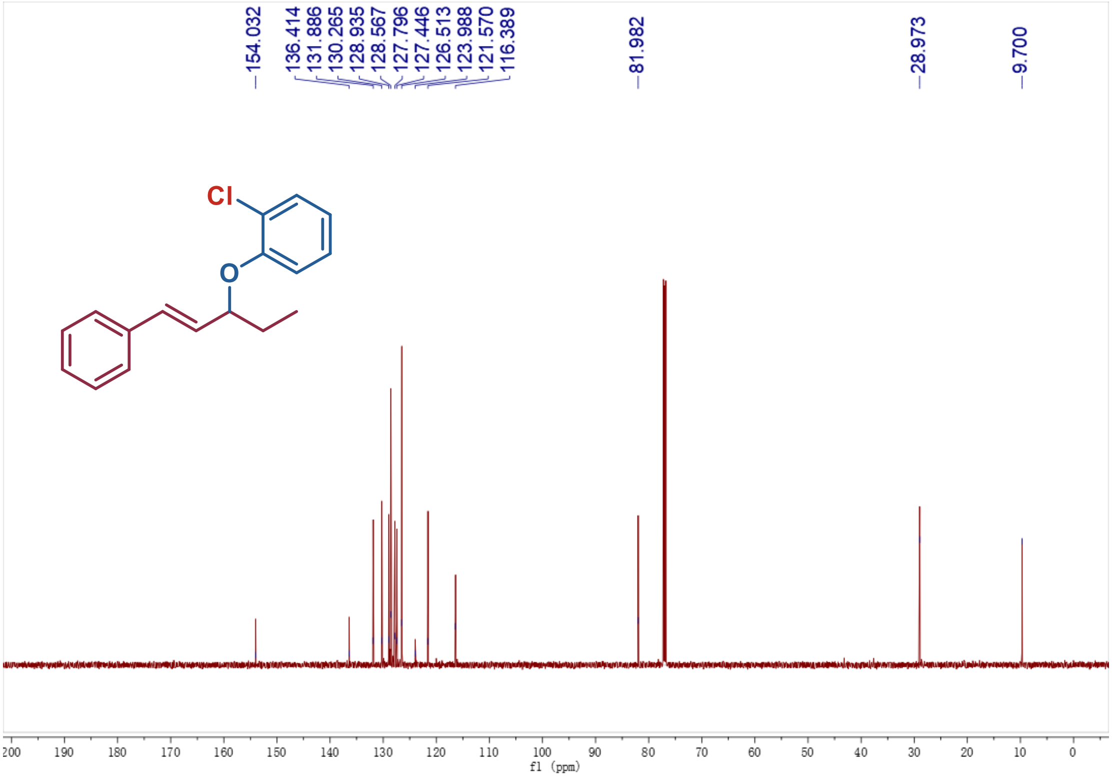

Fig. S79 ^{13}C NMR data of product 3ab.

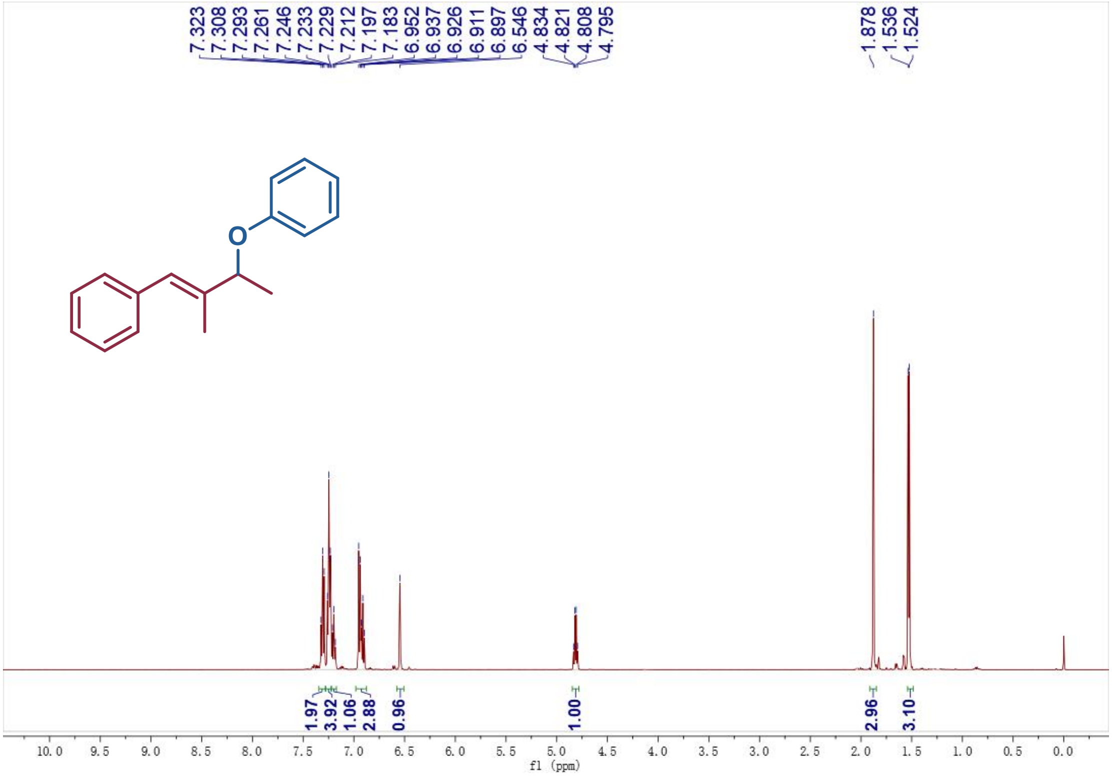

Fig. S80 ^{1}H NMR data of product 3ac.

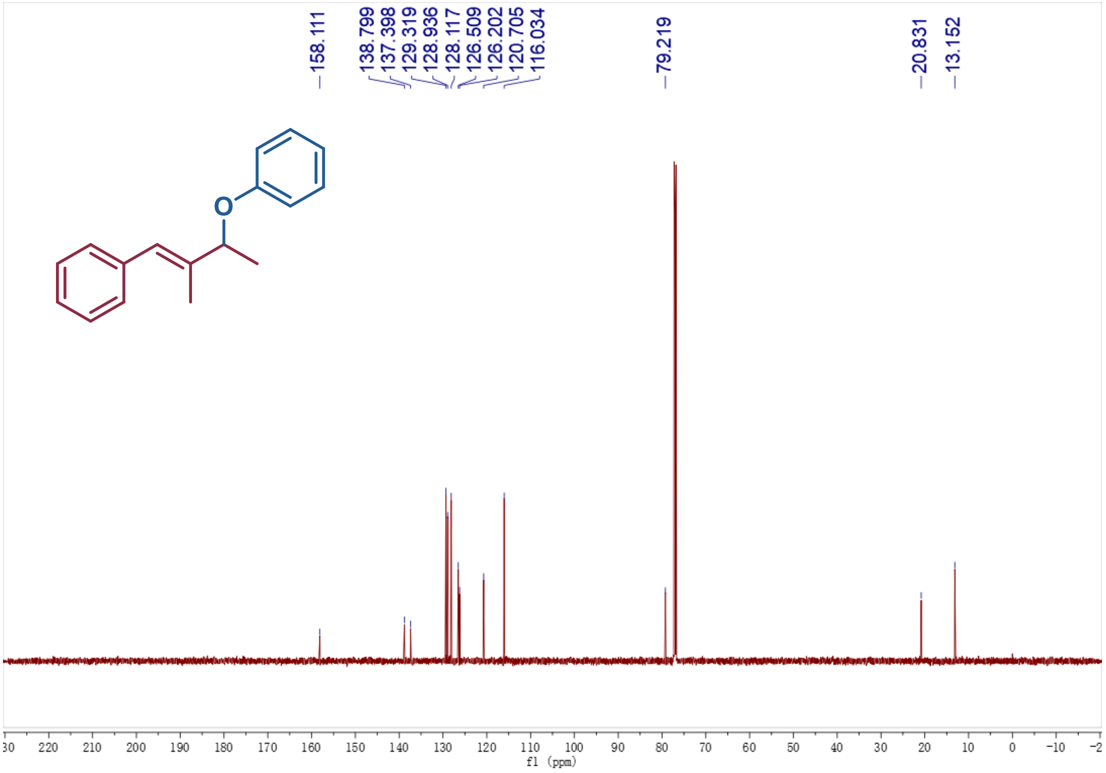

Fig. S81 ^{13}C NMR data of product 3ac.

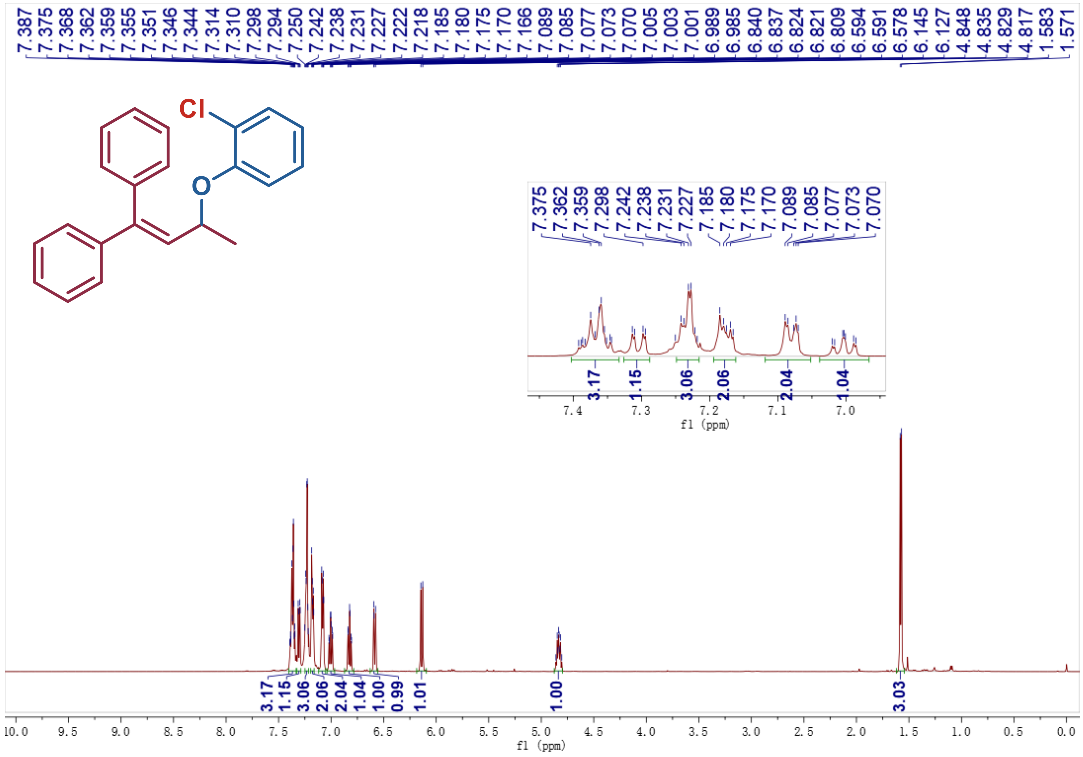

Fig. S82 ^{1}H NMR data of product 3ad.

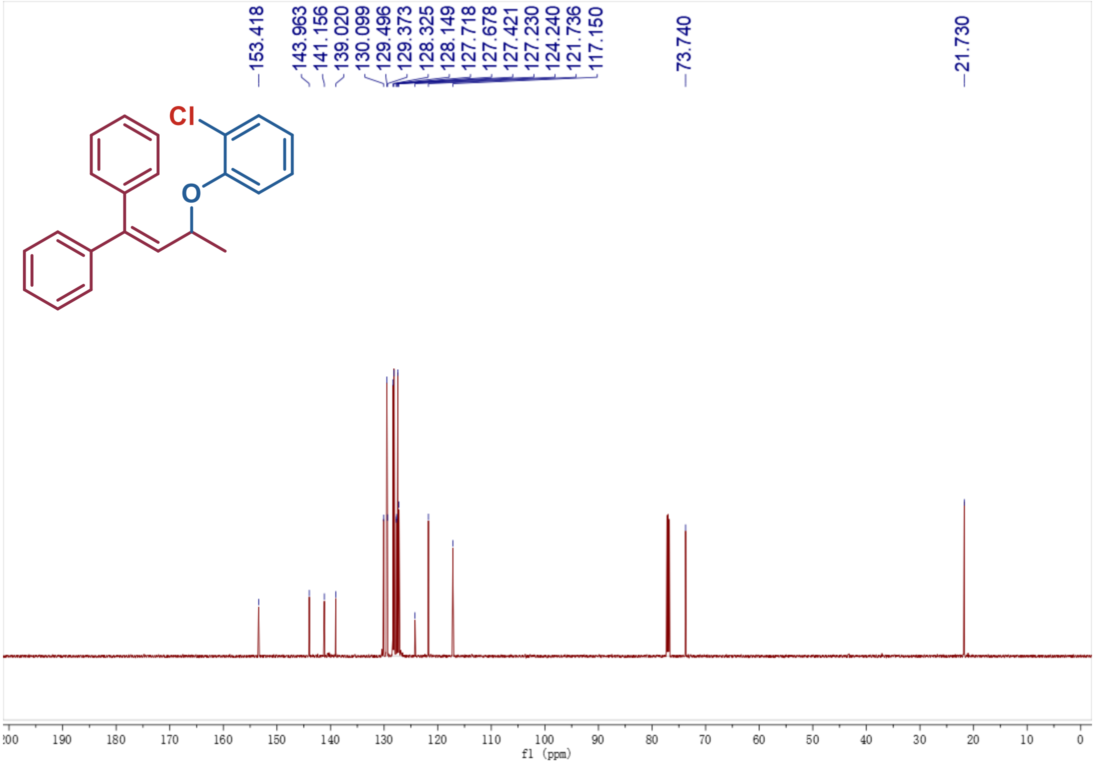

Fig. S83 ^{13}C NMR data of product 3ad.

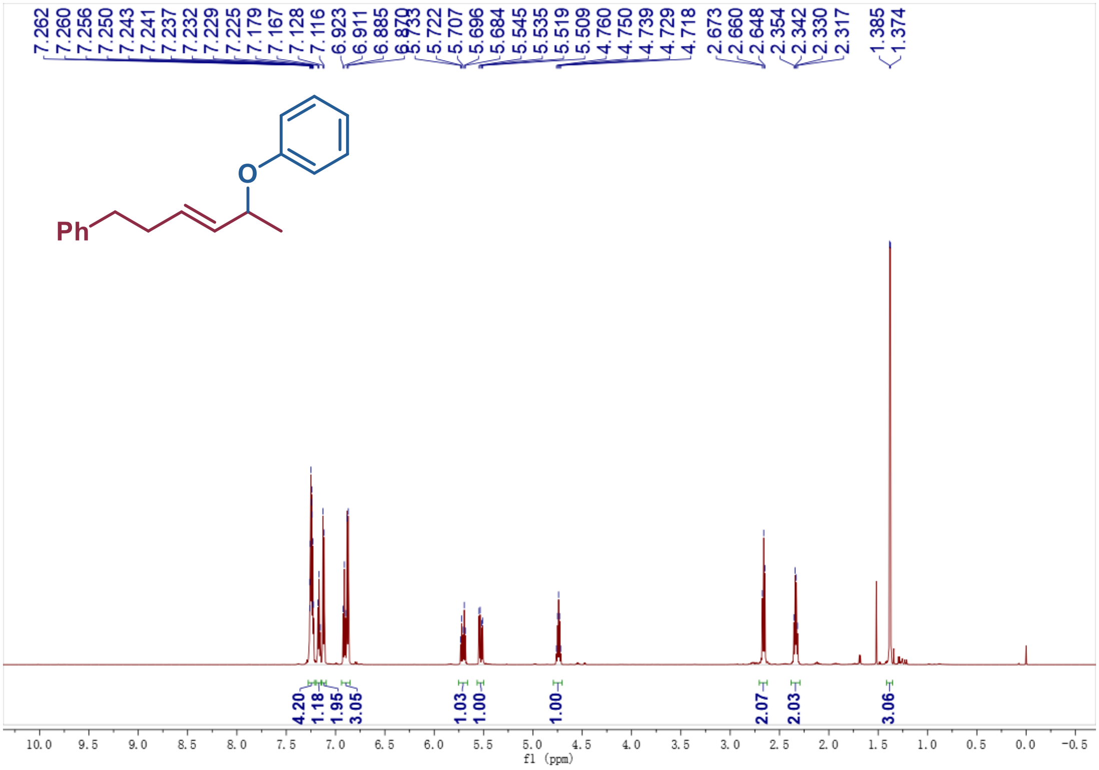

Fig. S84 ¹H NMR data of product 3ae.

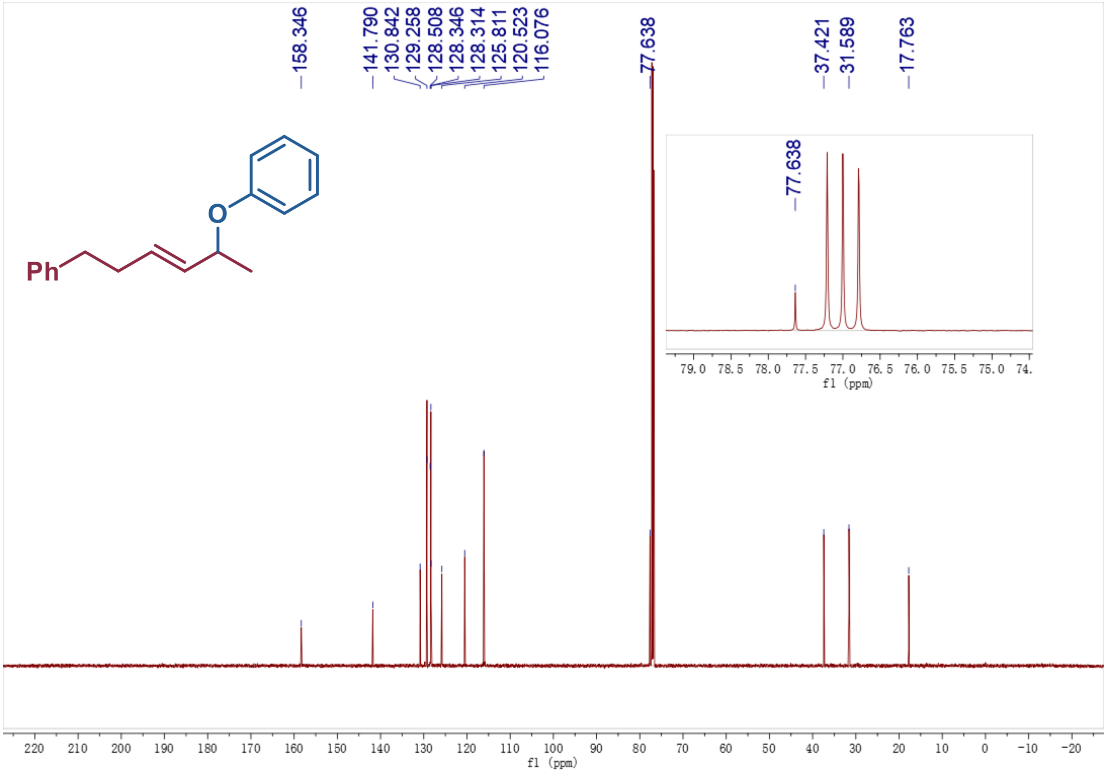

Fig. S85 ¹³C NMR data of product 3ae.

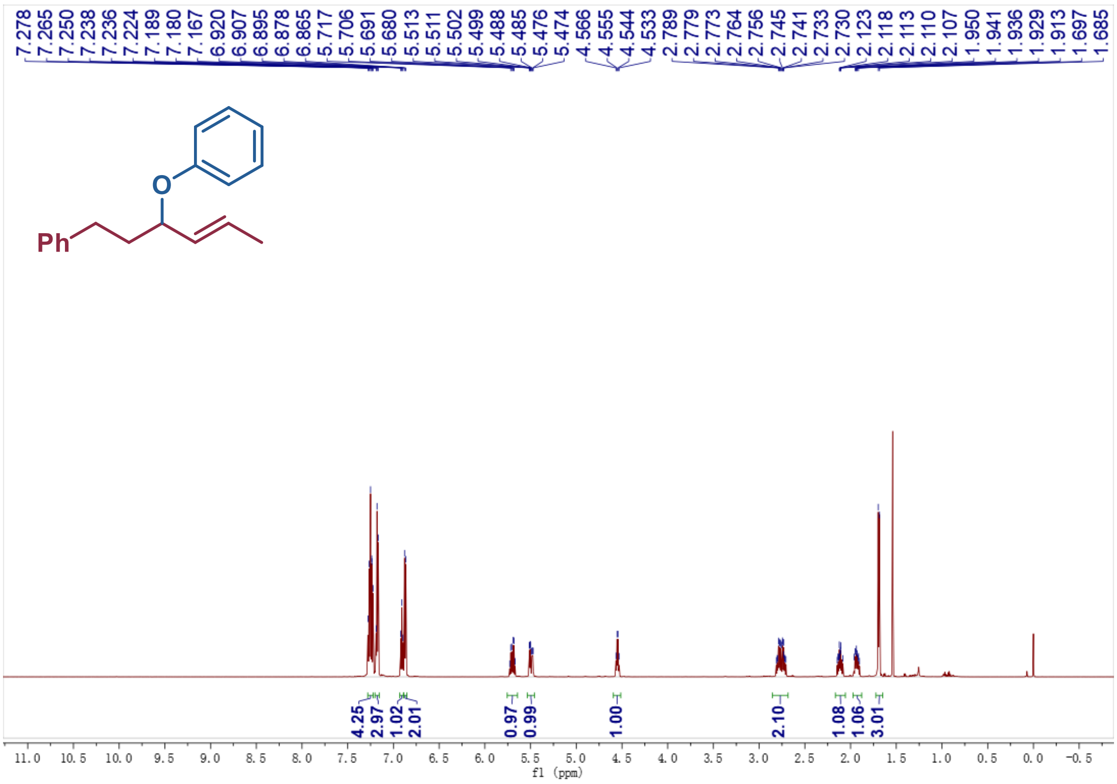

Fig. S86 ^{1}H NMR data of product 3ae’.

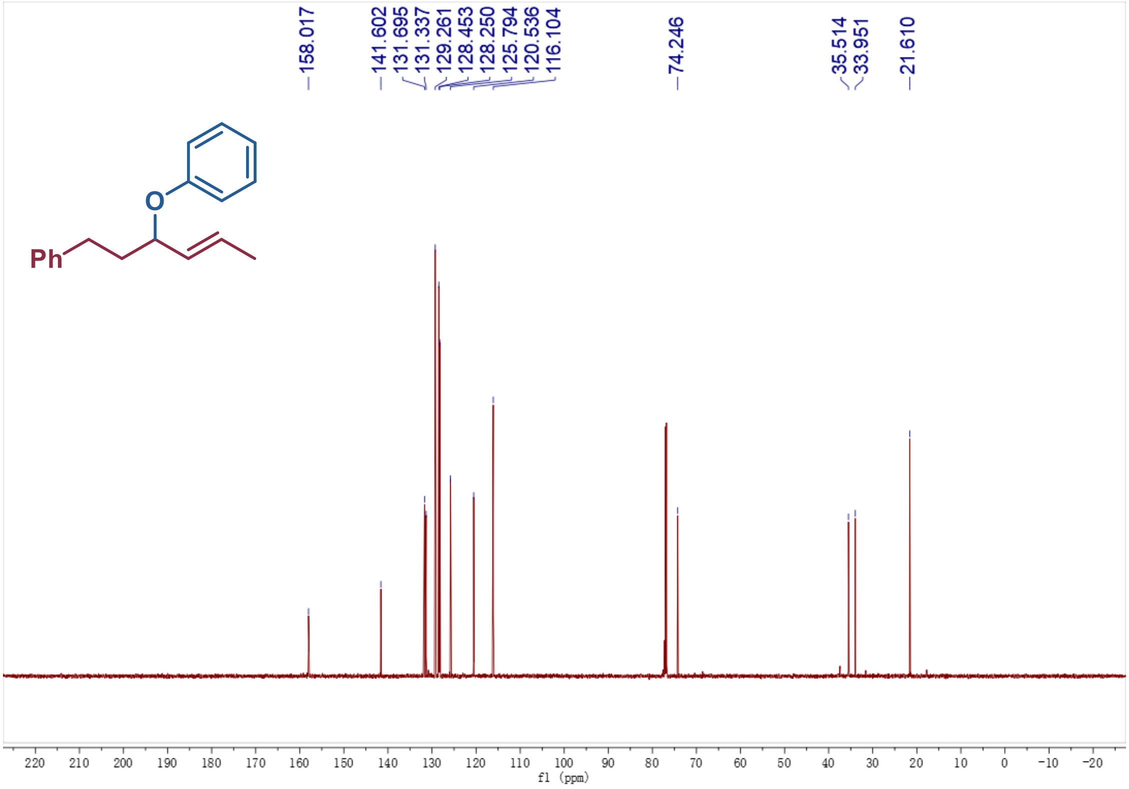

Fig. S87 ^{13}C NMR data of product 3ae’.

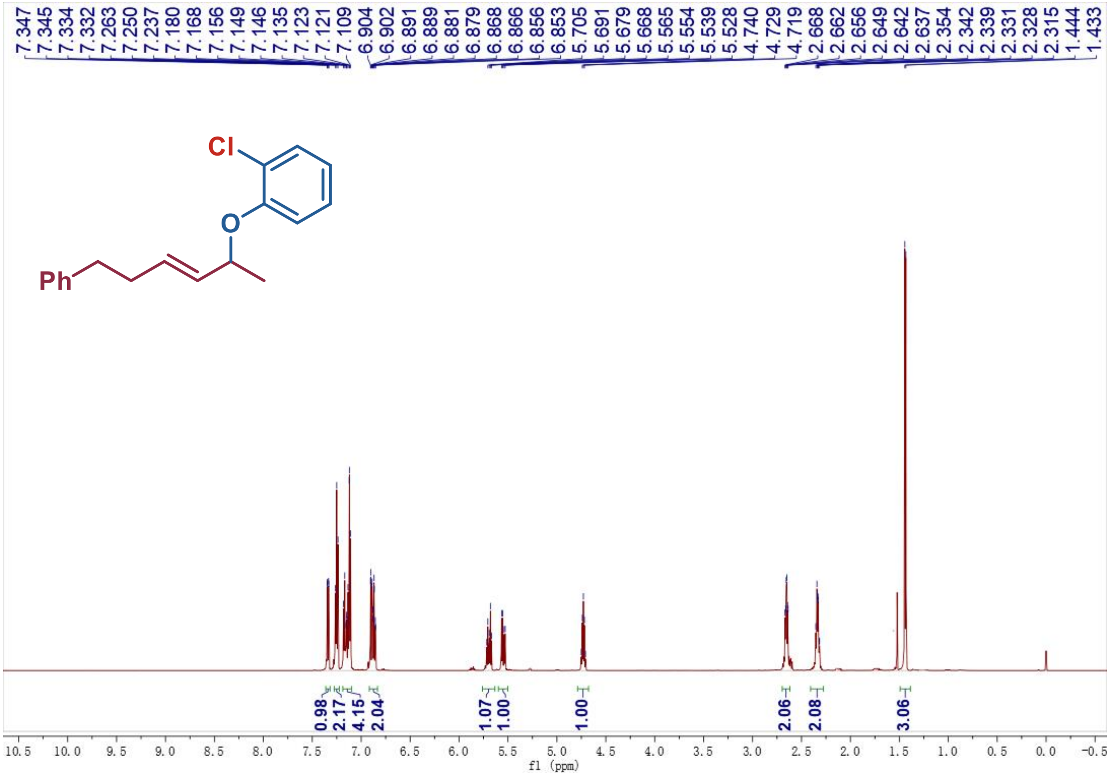

Fig. S88 ^{1}H NMR data of product 3af.

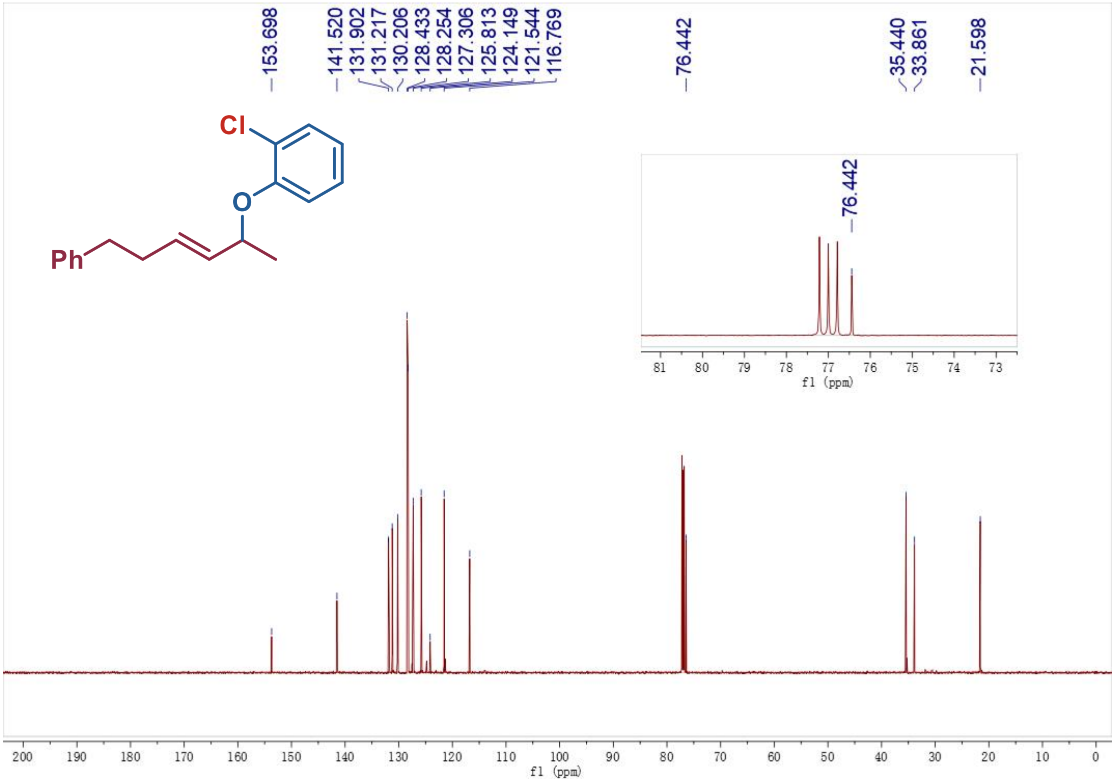

Fig. S89 ^{13}C NMR data of product 3af.

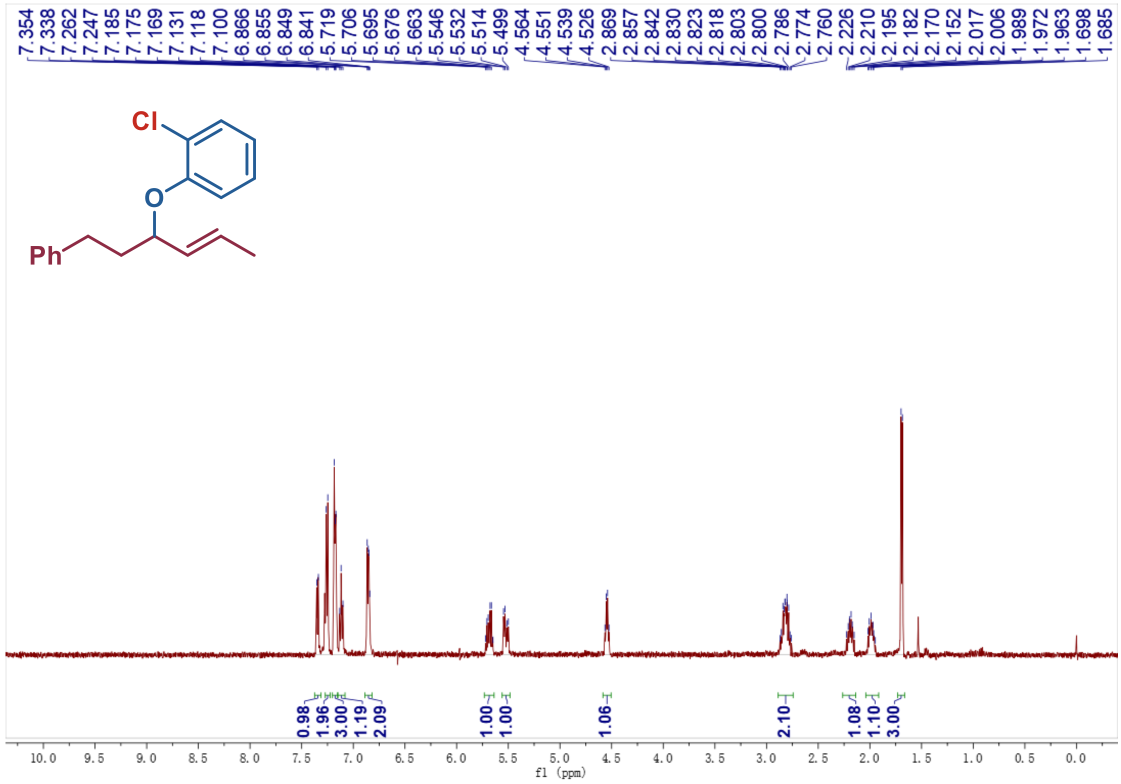

Fig. S90 ^{1}H NMR data of product 3af'.

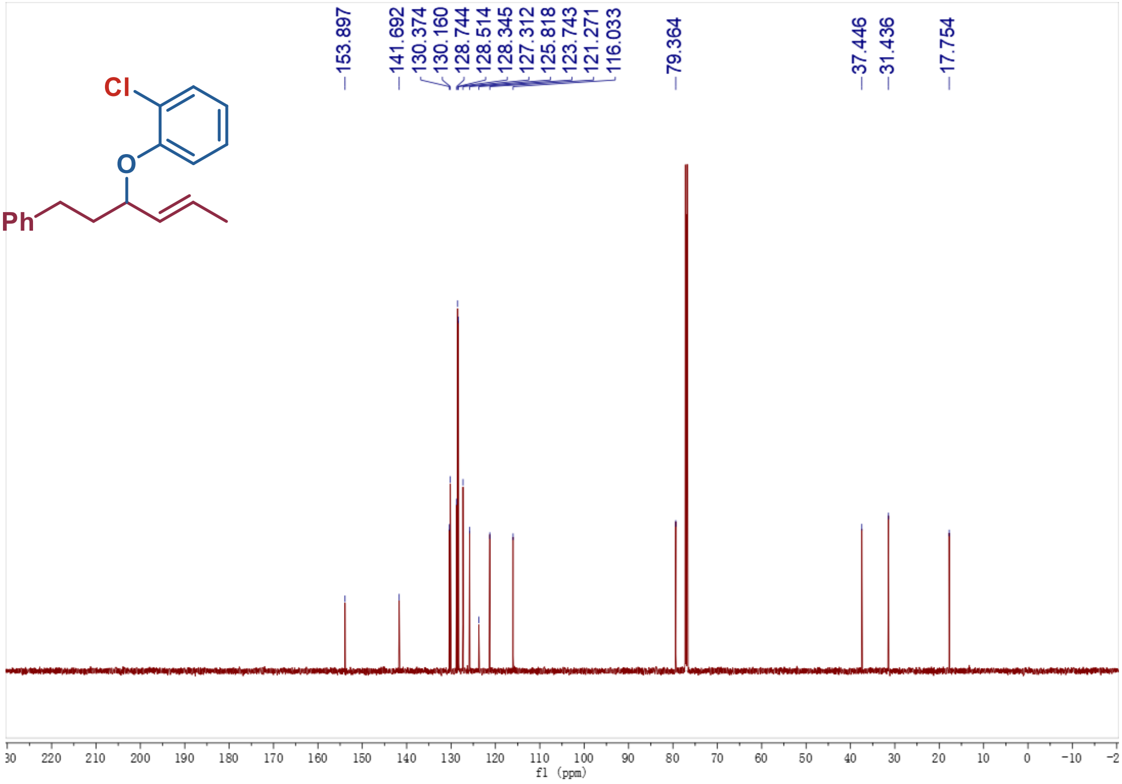

Fig. S91 ^{13}C NMR data of product 3af'.

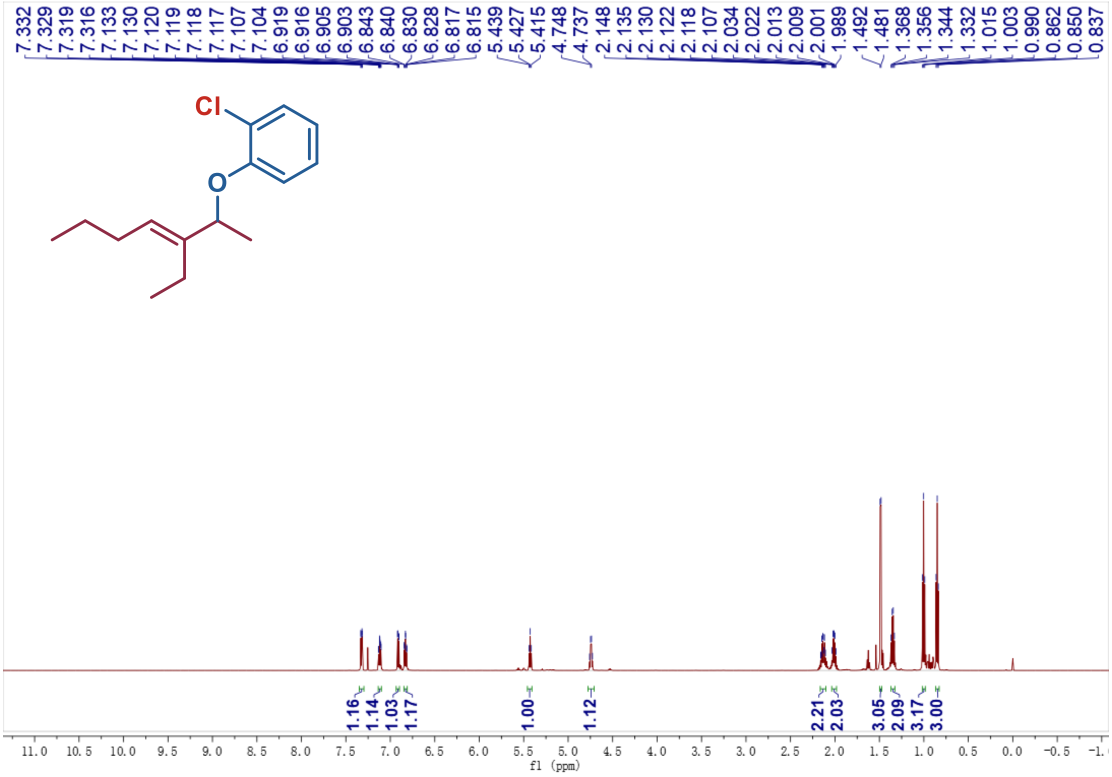

Fig. S92 ^{1}H NMR data of product 3ag.

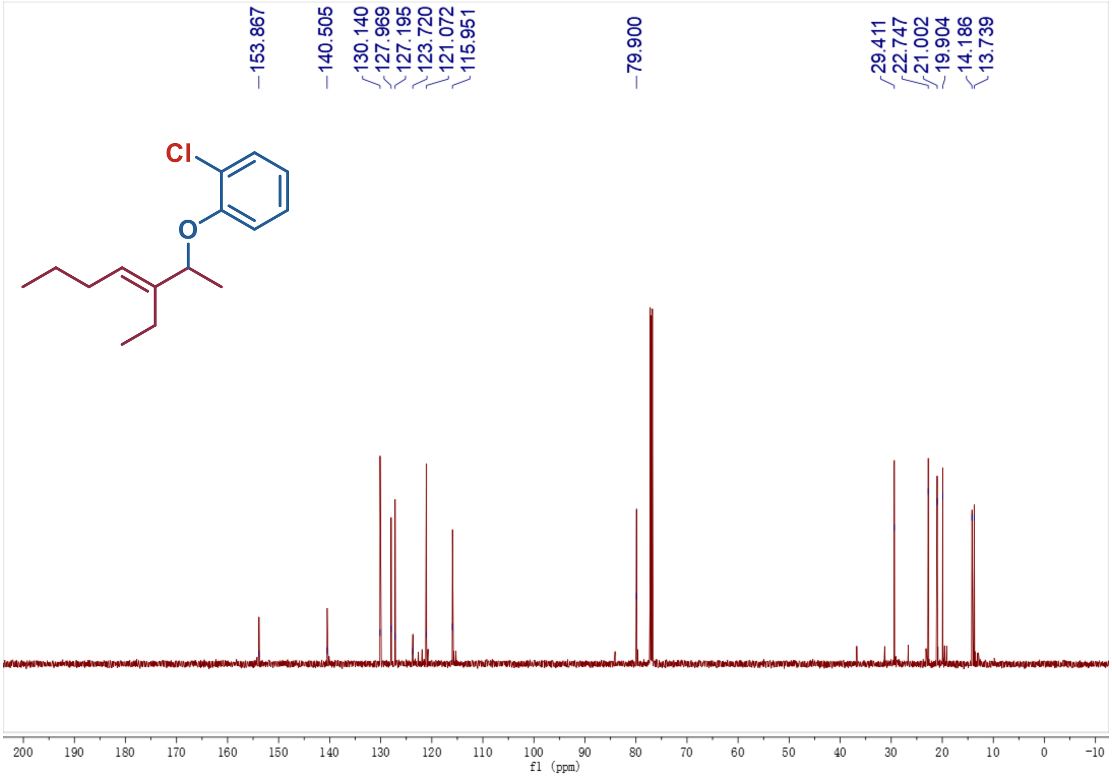

Fig. S93 ^{13}C NMR data of product 3ag.

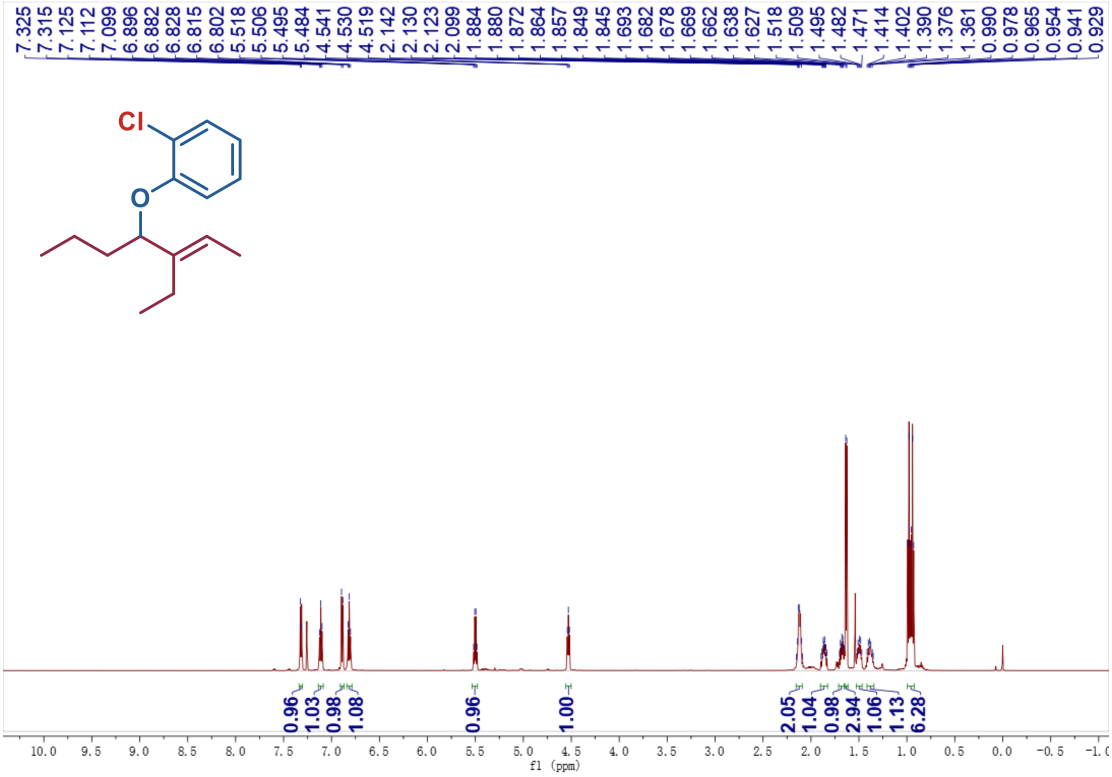

Fig. S94 ^{1}H NMR data of product 3ag’.

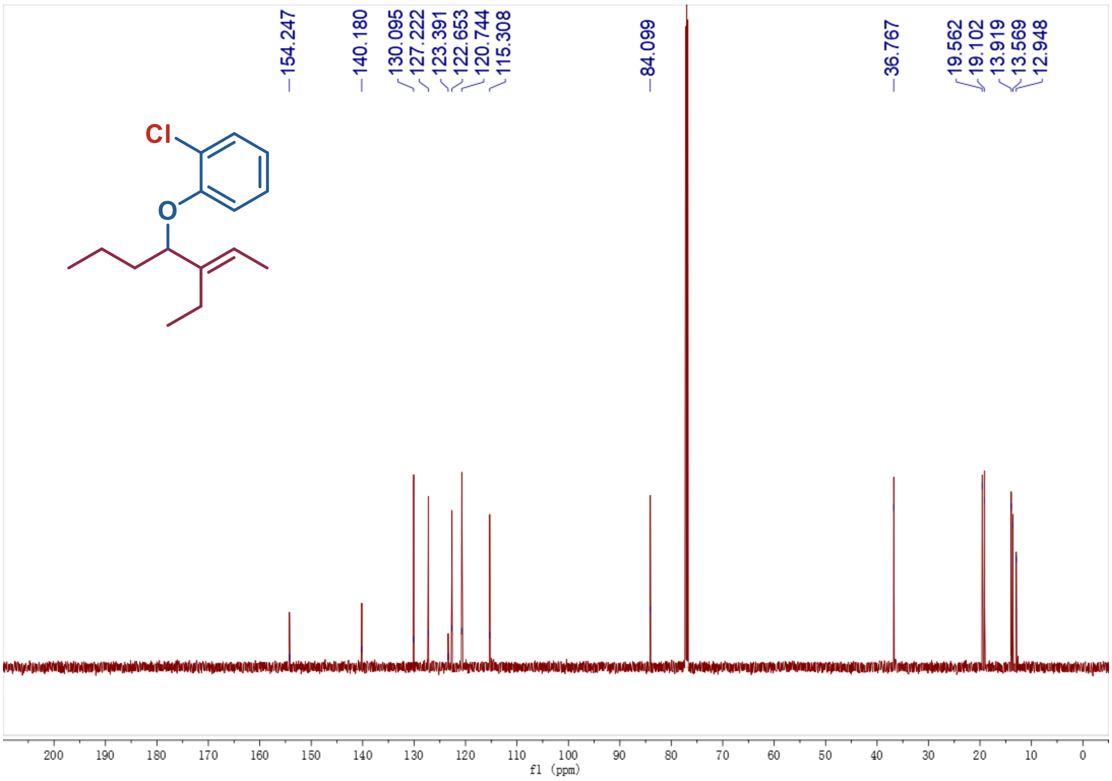

Fig. S95 ^{13}C NMR data of product 3ag’.

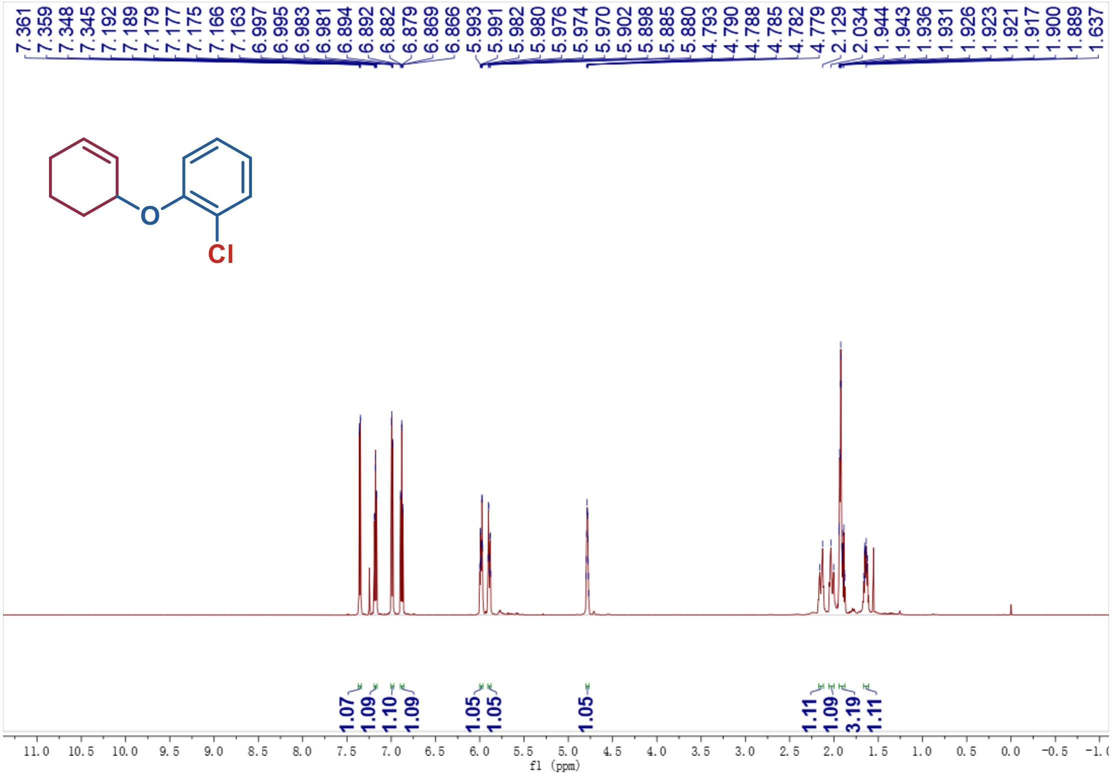

Fig. S96 ^{1}H NMR data of product 3ah.

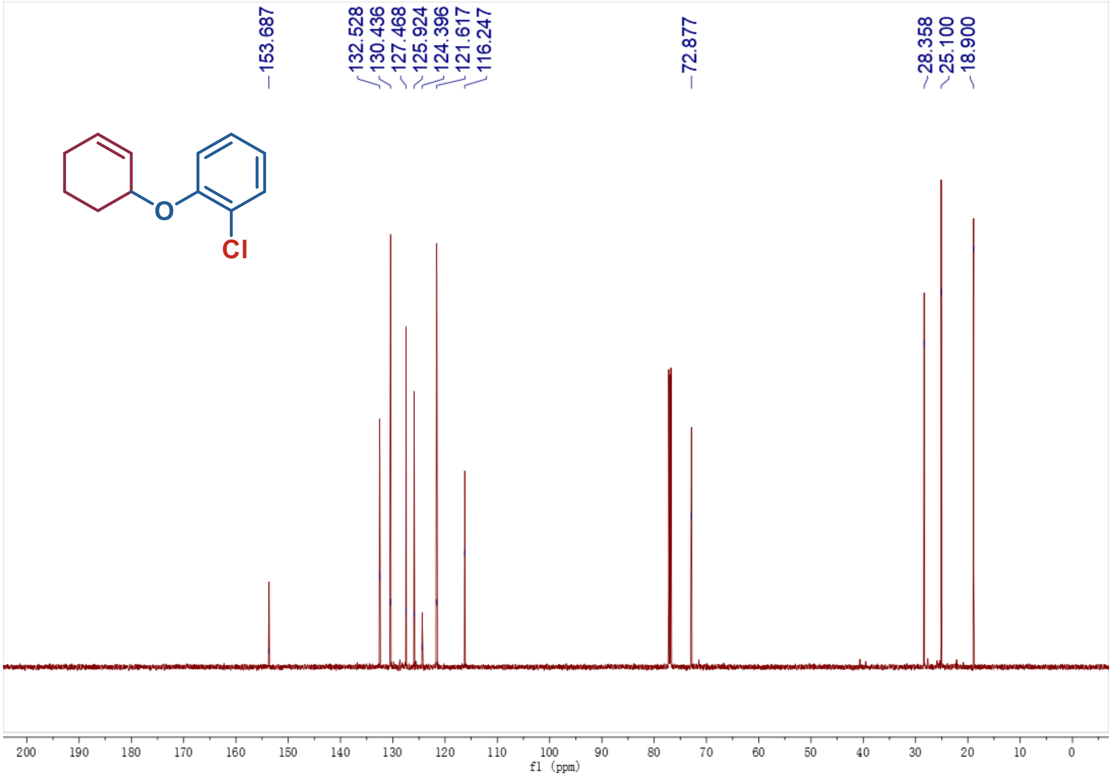

Fig. S97 ^{13}C NMR data of product 3ah.

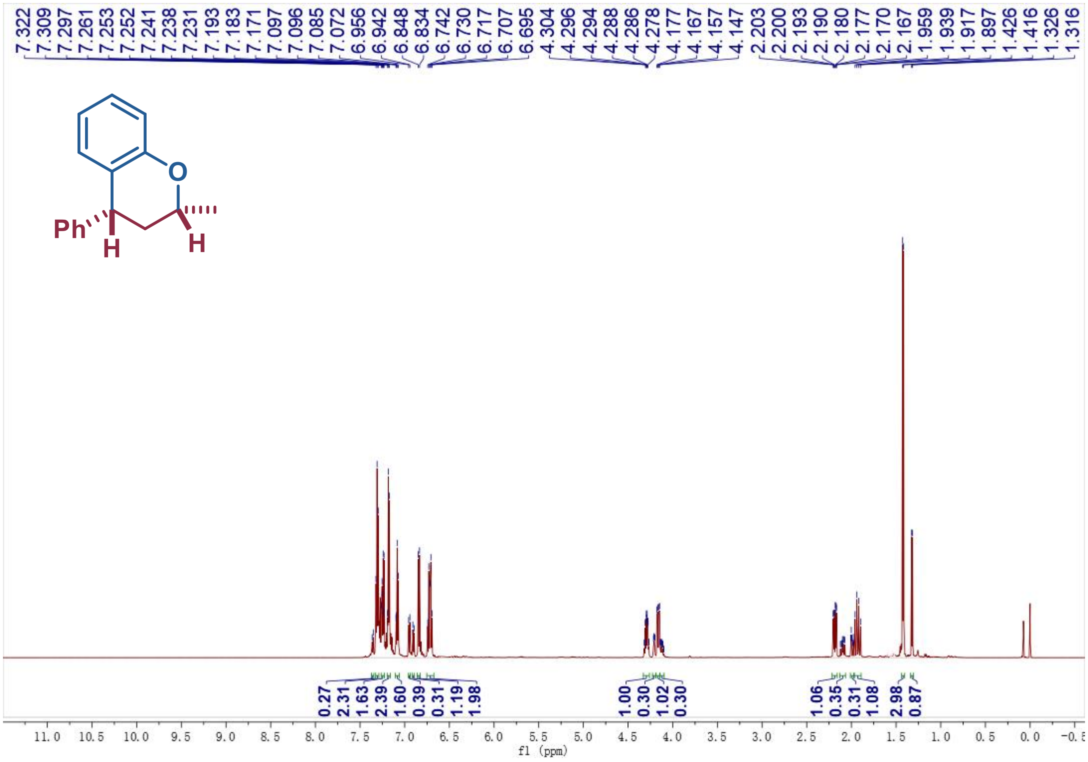

Fig. S98 ^{1}H NMR data of product 4a.

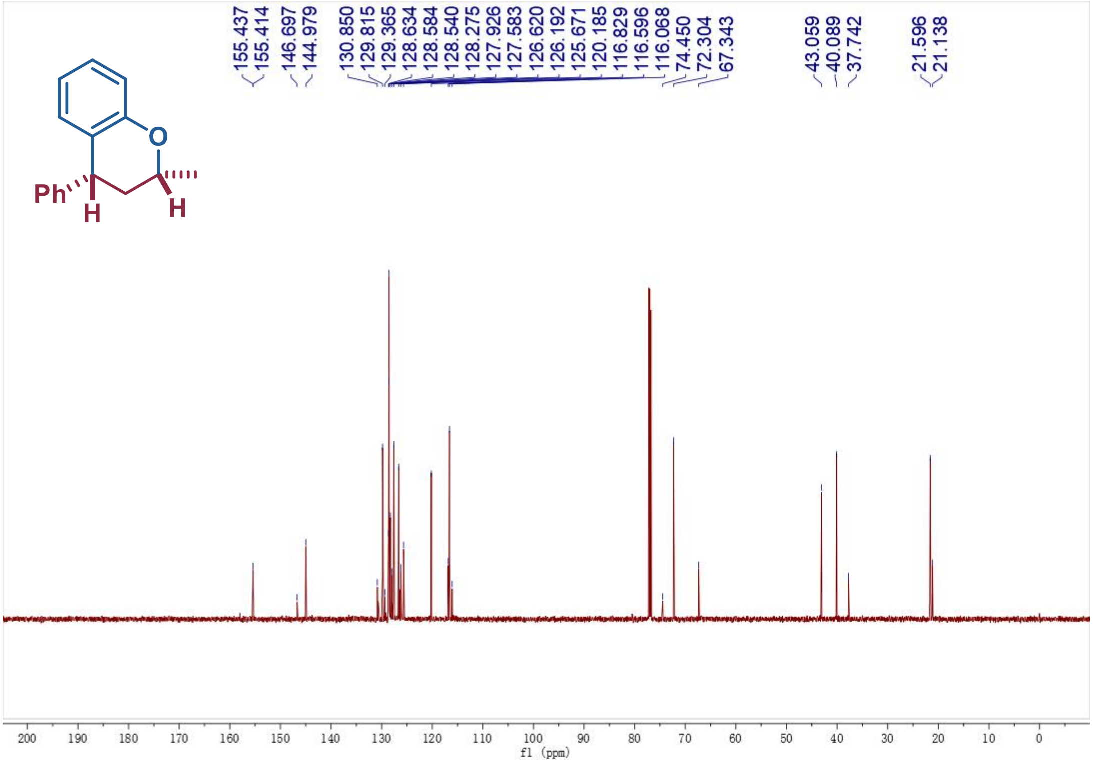

Fig. S99 ^{13}C NMR data of product 4a.

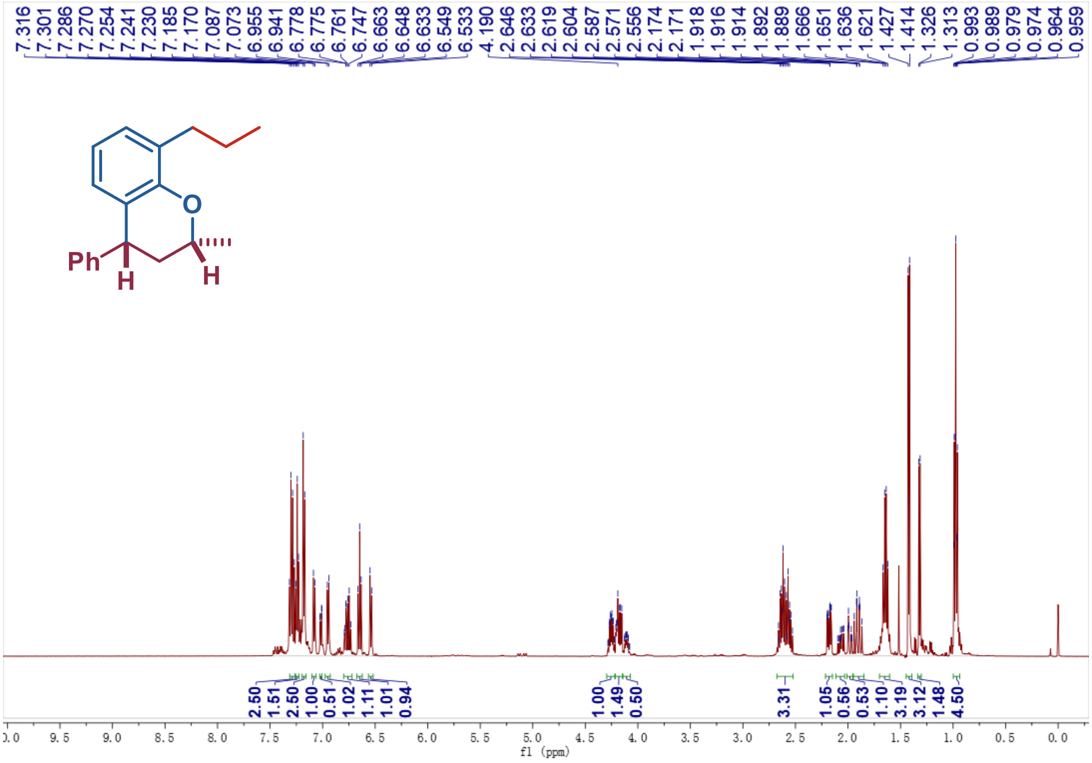

Fig. S100 ^{1}H NMR data of product 4b.

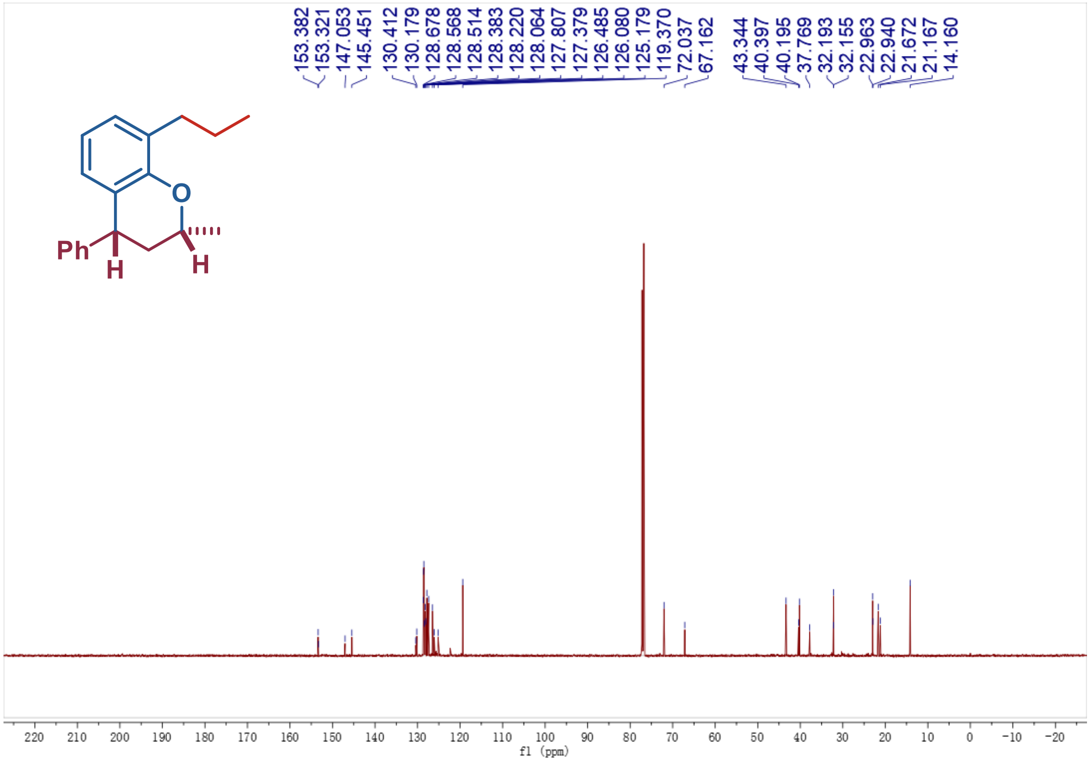

Fig. S101 ^{13}C NMR data of product 4b.

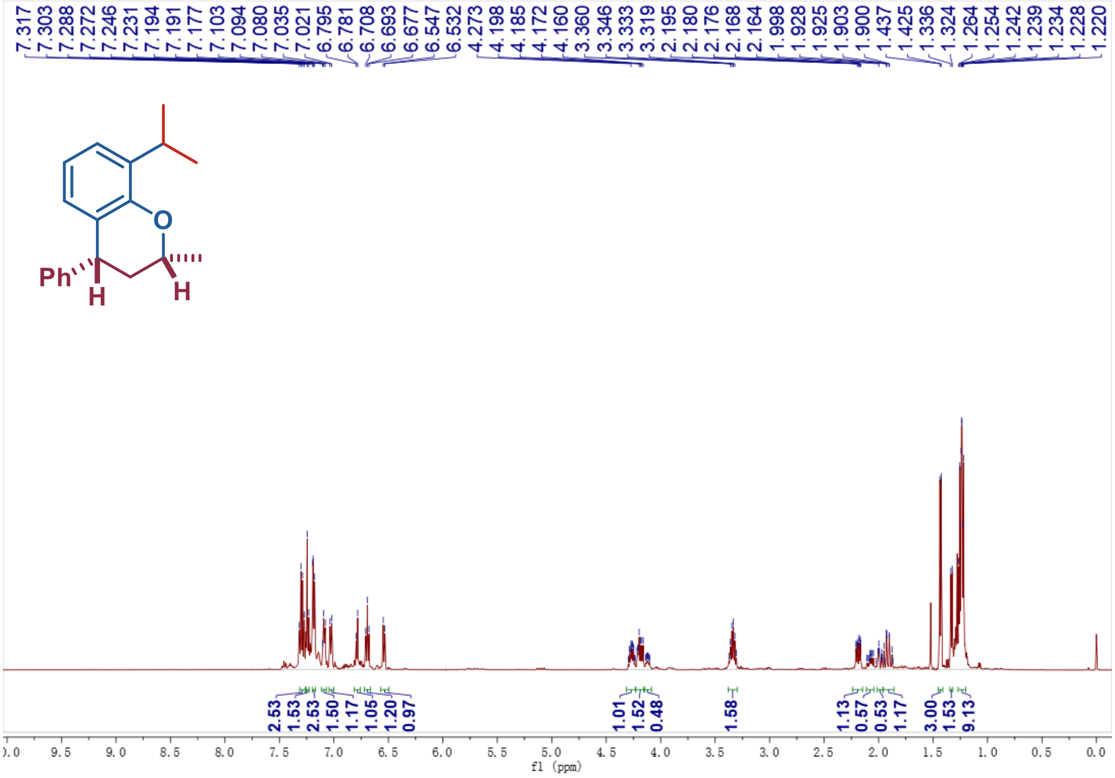

Fig. S102 ^{1}H NMR data of product 4c.

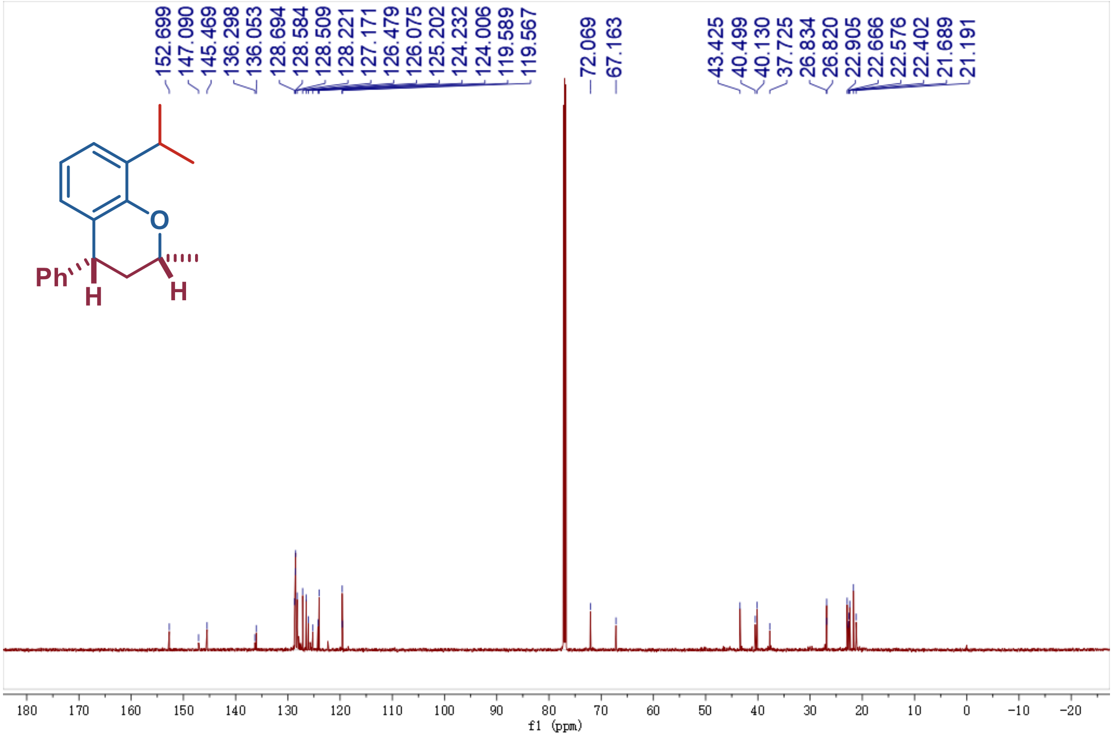

Fig. S103 ^{13}C NMR data of product 4c.

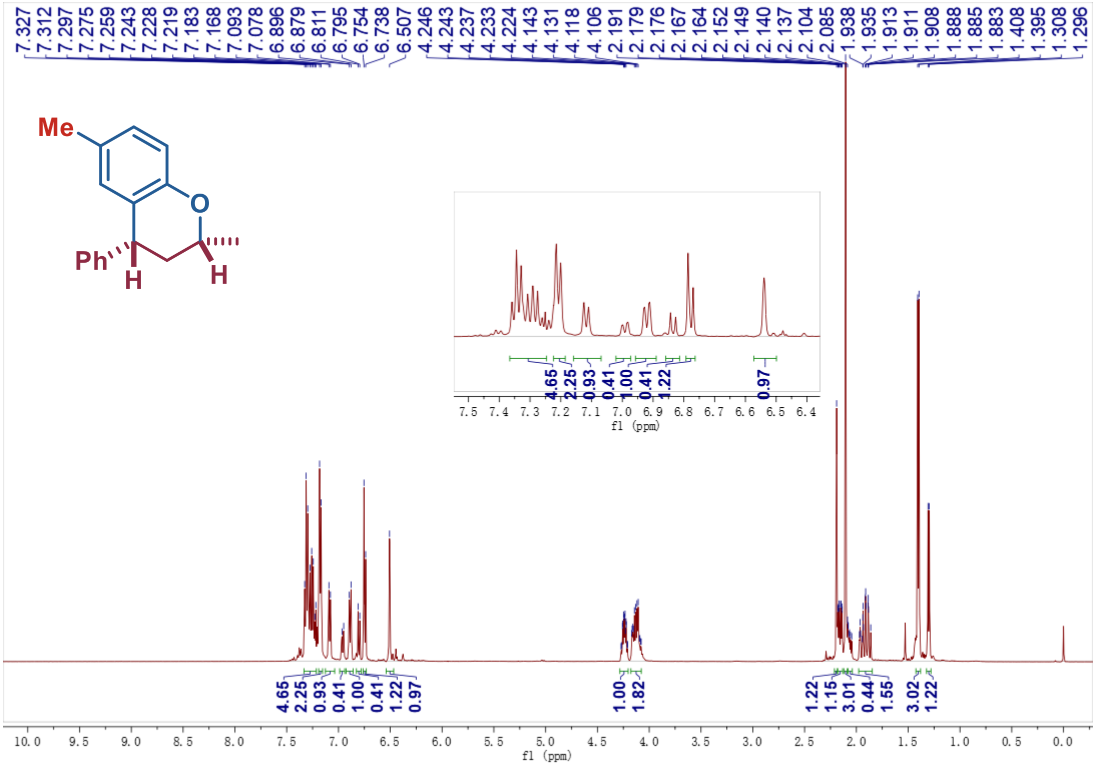

Fig. S104 ^{1}H NMR data of product 4d.

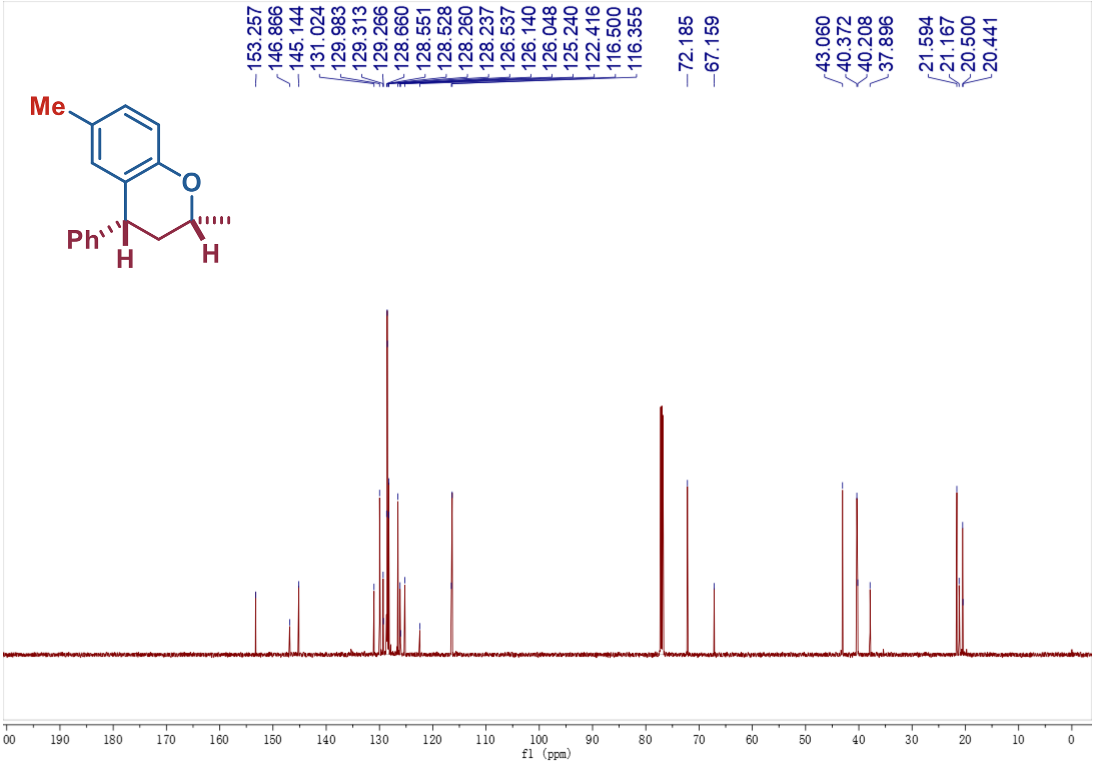

Fig. S105 ^{13}C NMR data of product 4d.

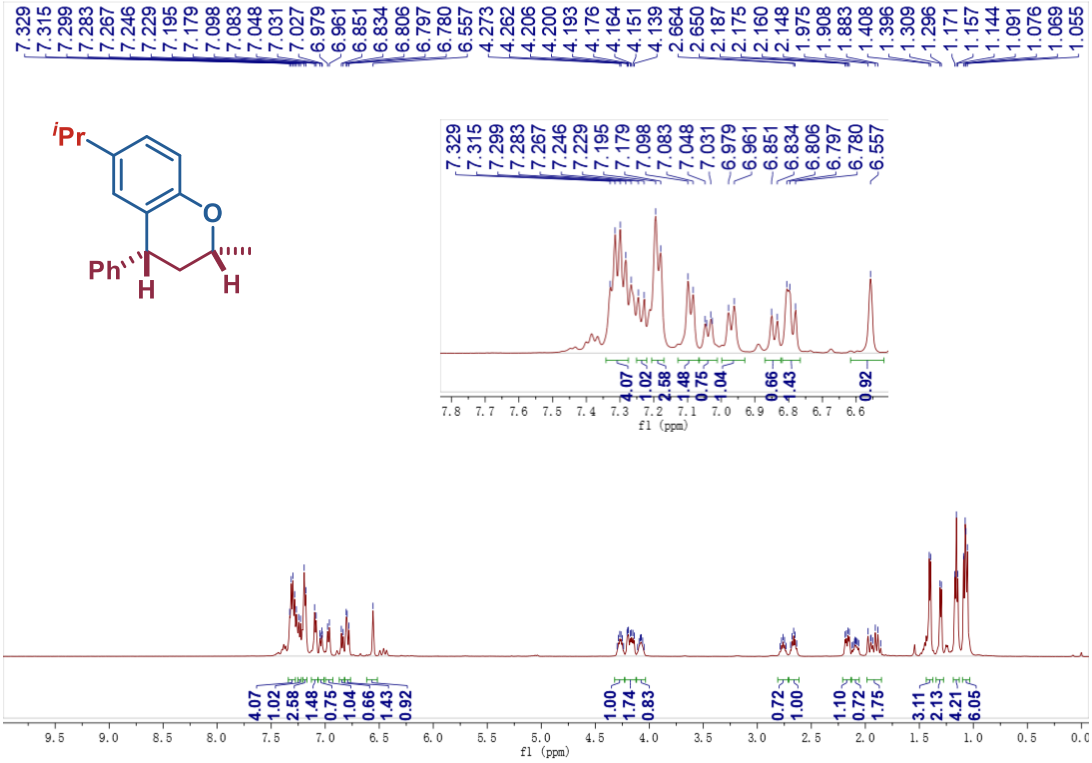

Fig. S106 ^{1}H NMR data of product 4e.

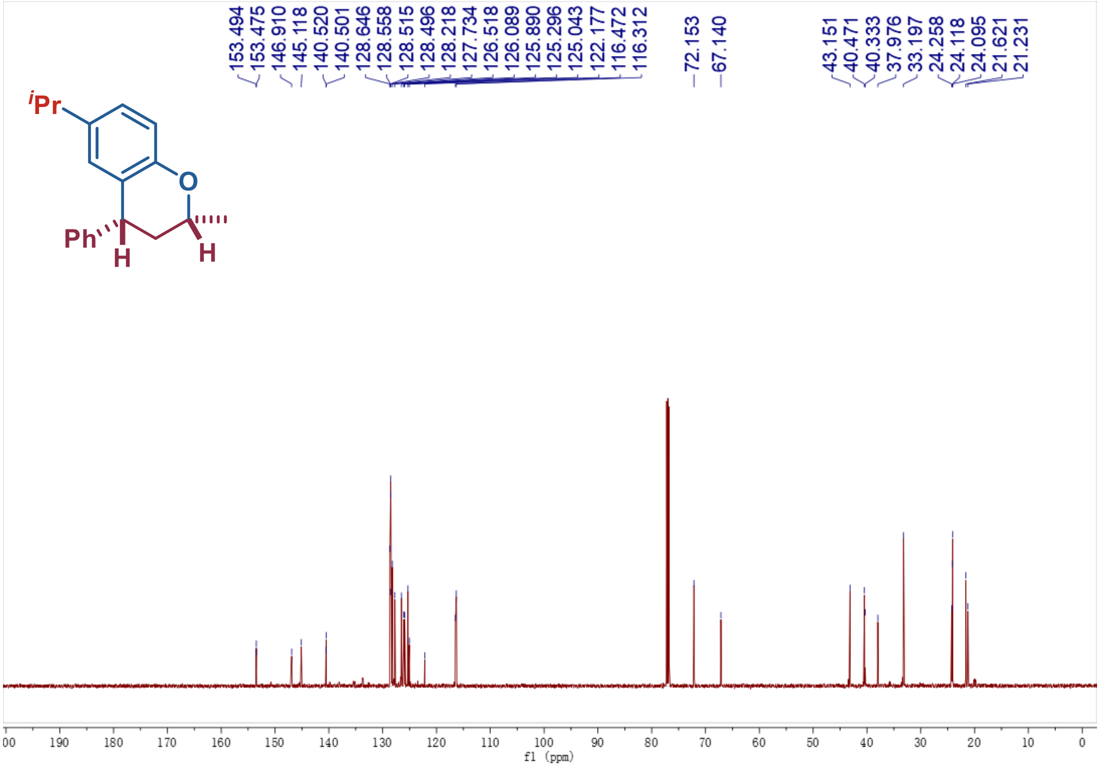

Fig. S107 ^{13}C NMR data of product 4e.

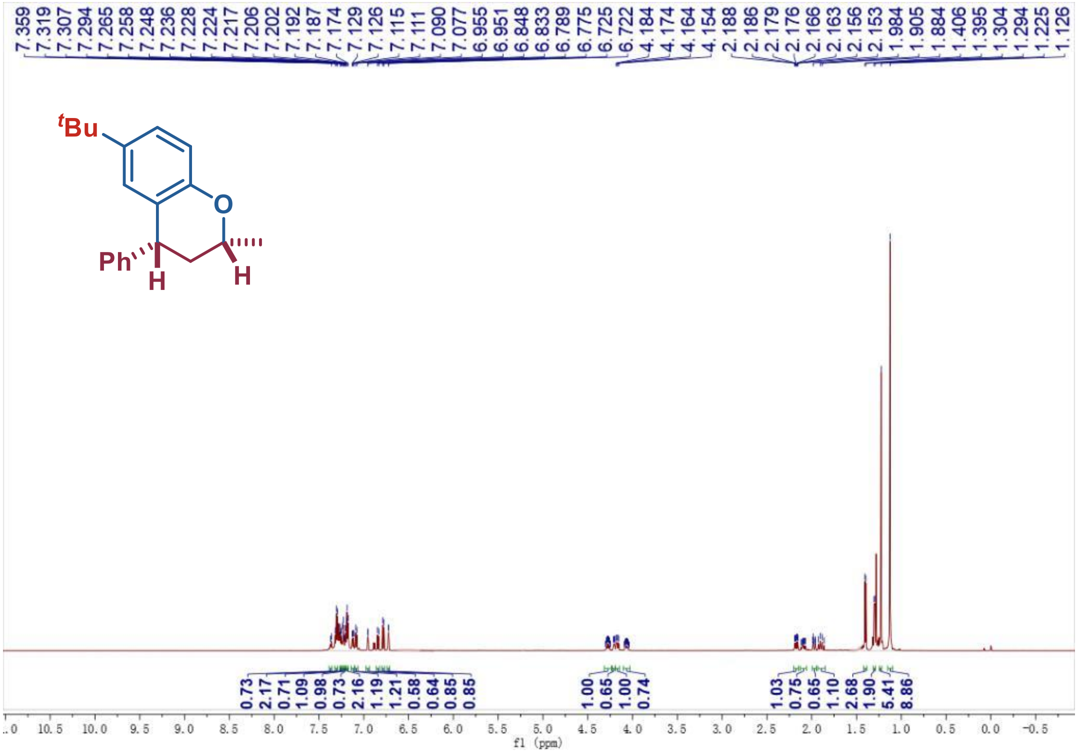

Fig. S108 ^{1}H NMR data of product 4f.

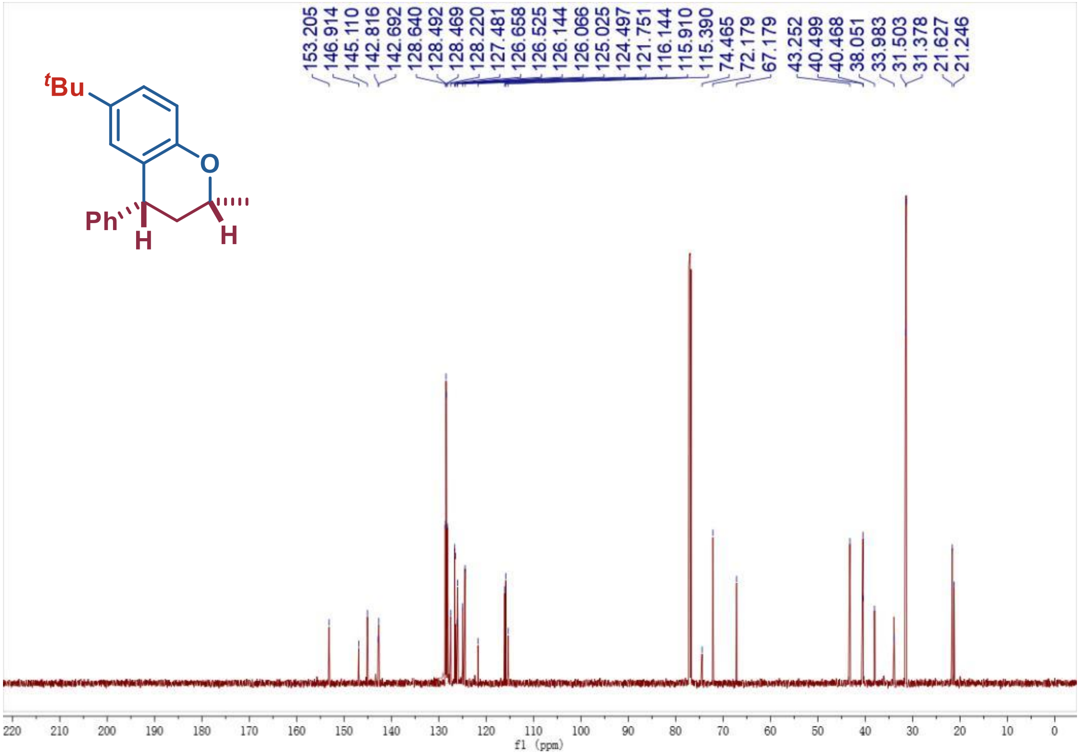

Fig. S109 ^{13}C NMR data of product 4f.

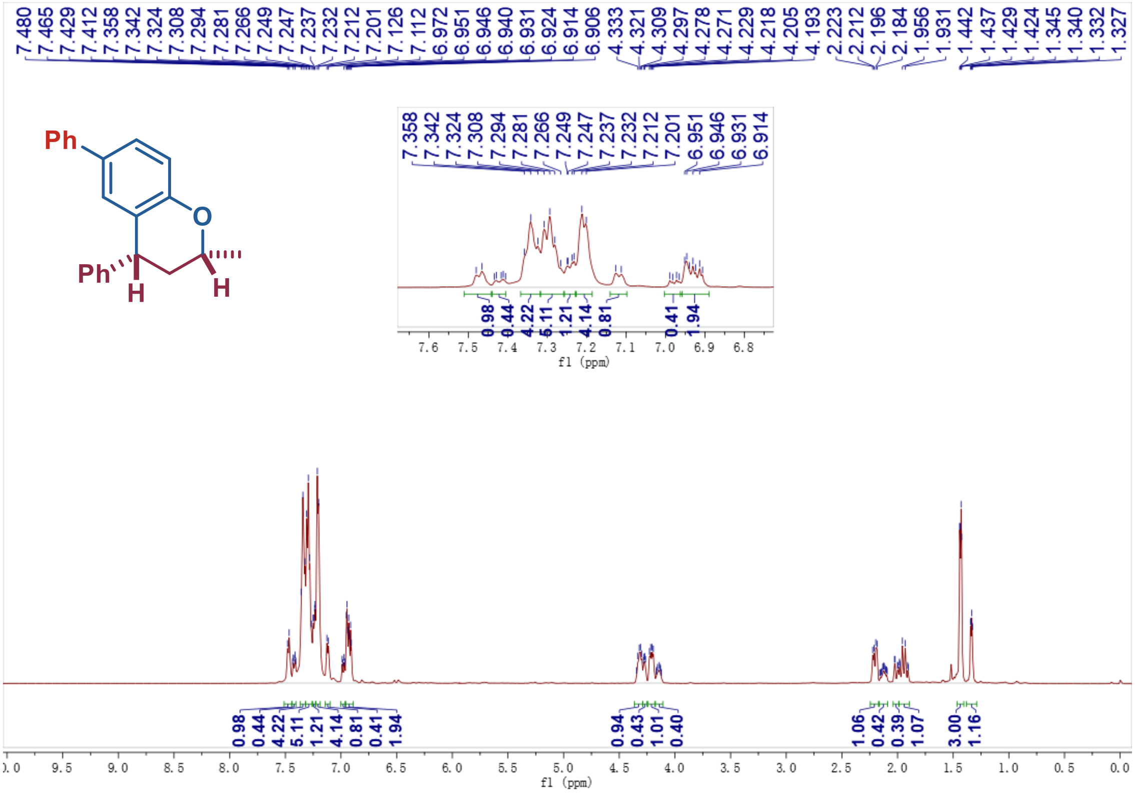

Fig. S110 ^{1}H NMR data of product 4g.

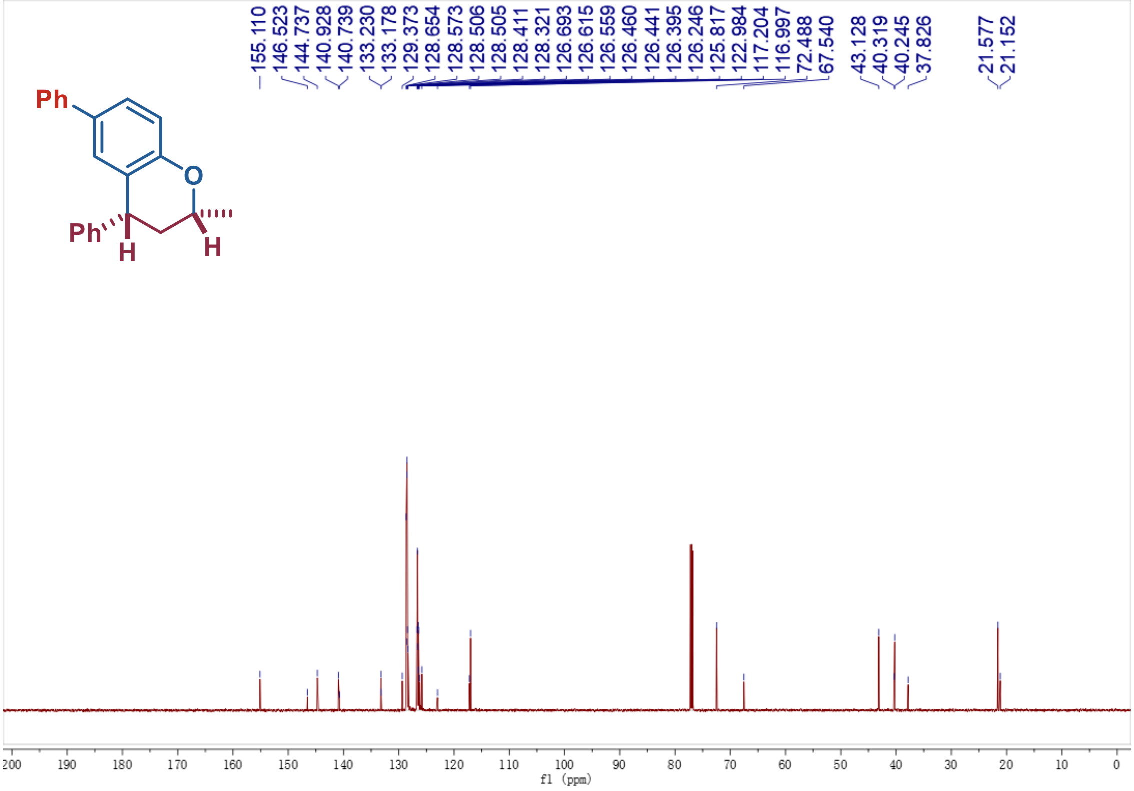

Fig. S111 ^{13}C NMR data of product 4g.

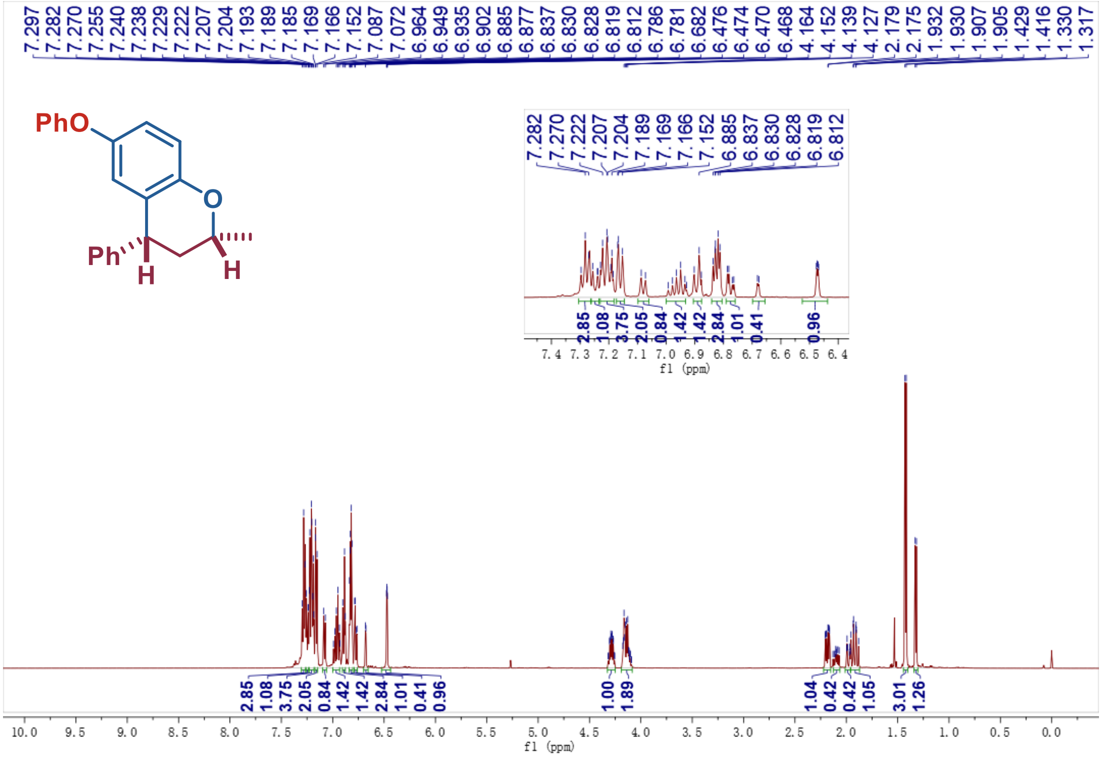

Fig. S112 ^{1}H NMR data of product 4h.

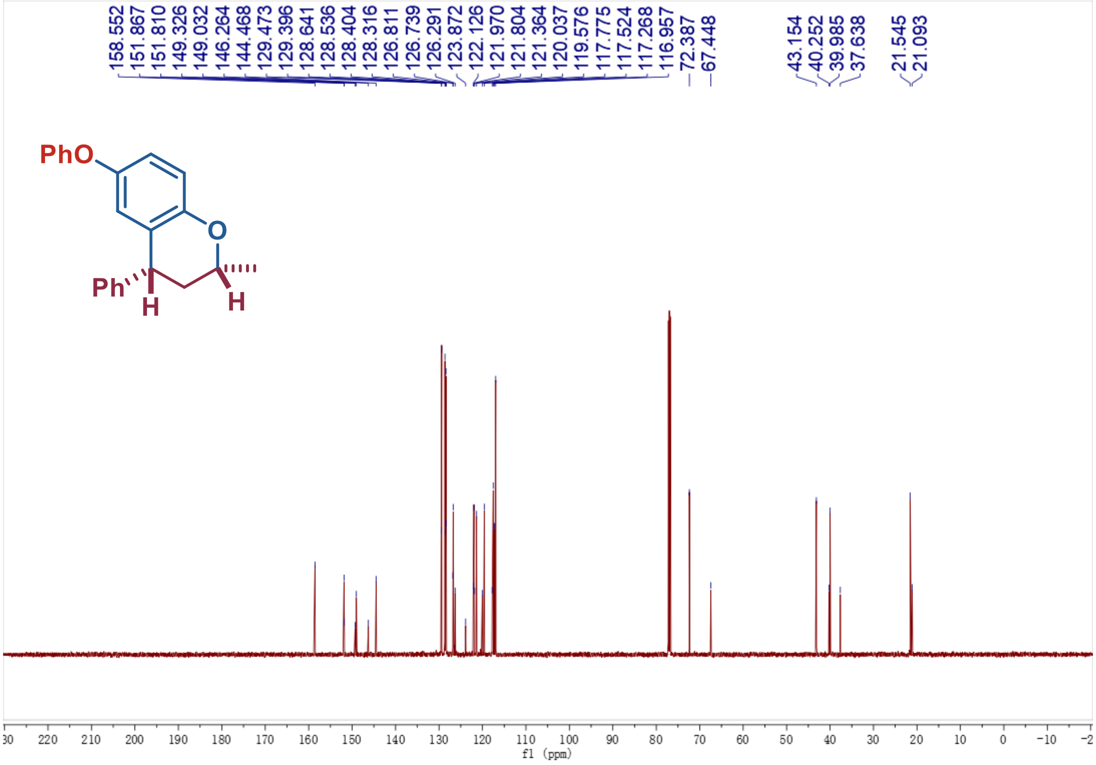

Fig. S113 ^{13}C NMR data of product 4h.

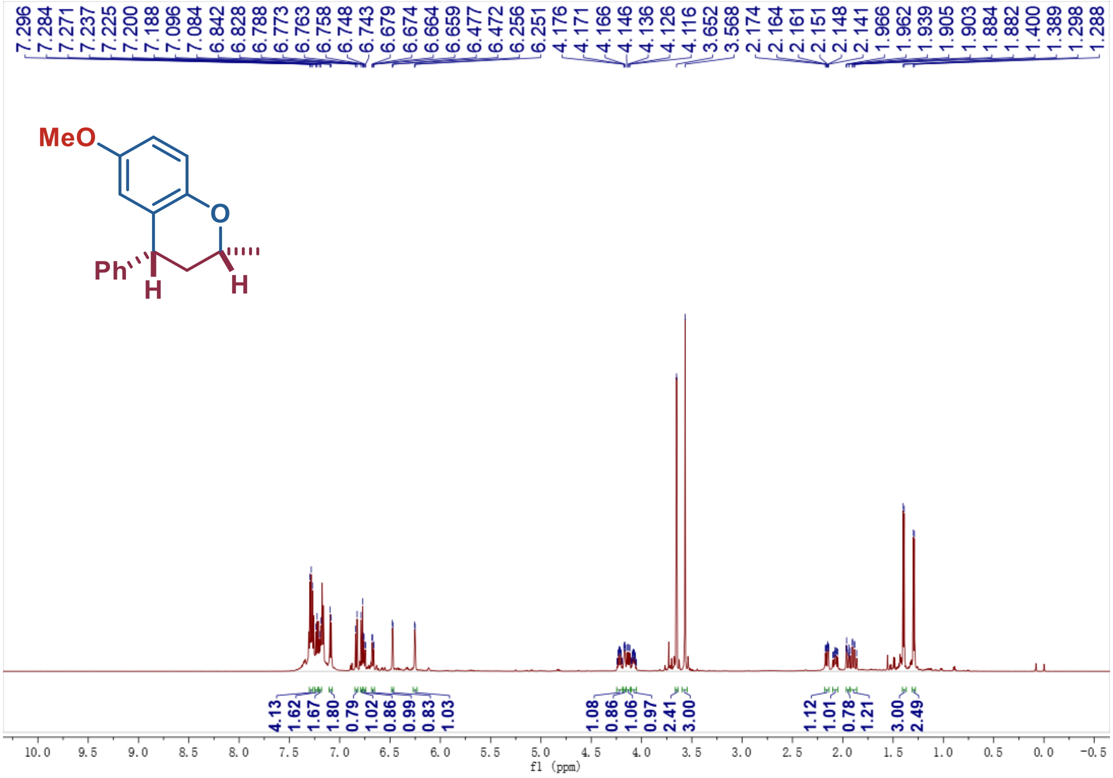

Fig. S114 ^{1}H NMR data of product 4i.

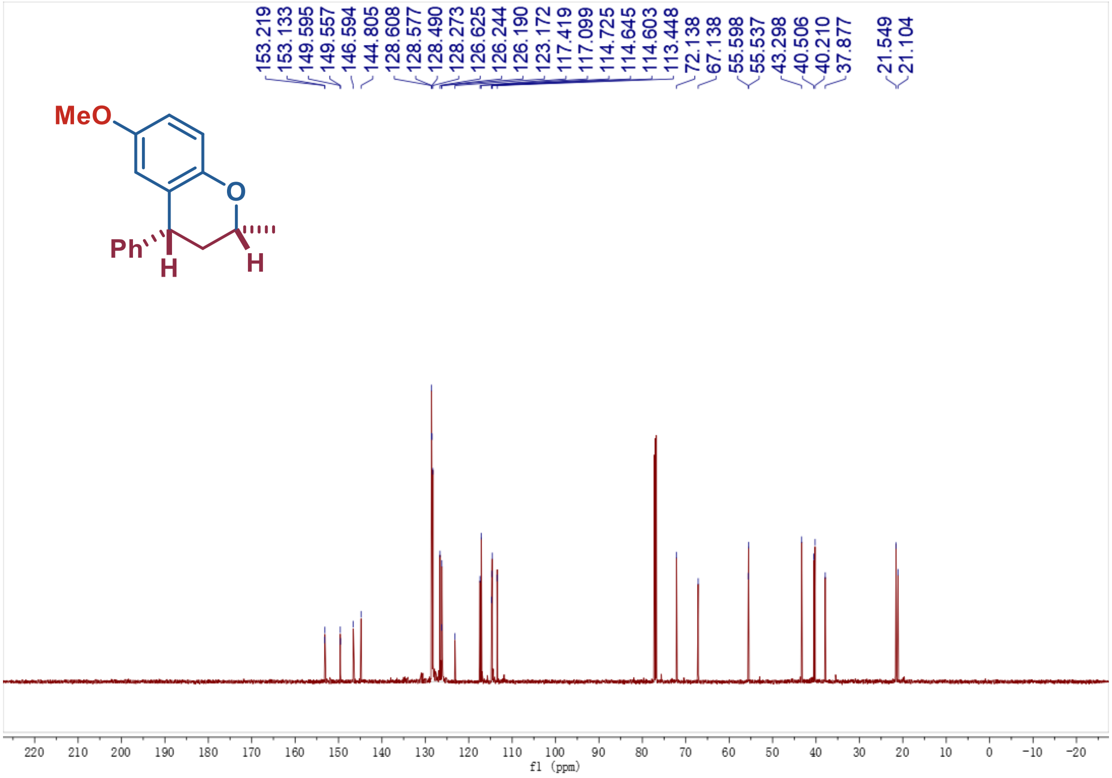

Fig. S115 ^{13}C NMR data of product 4i.

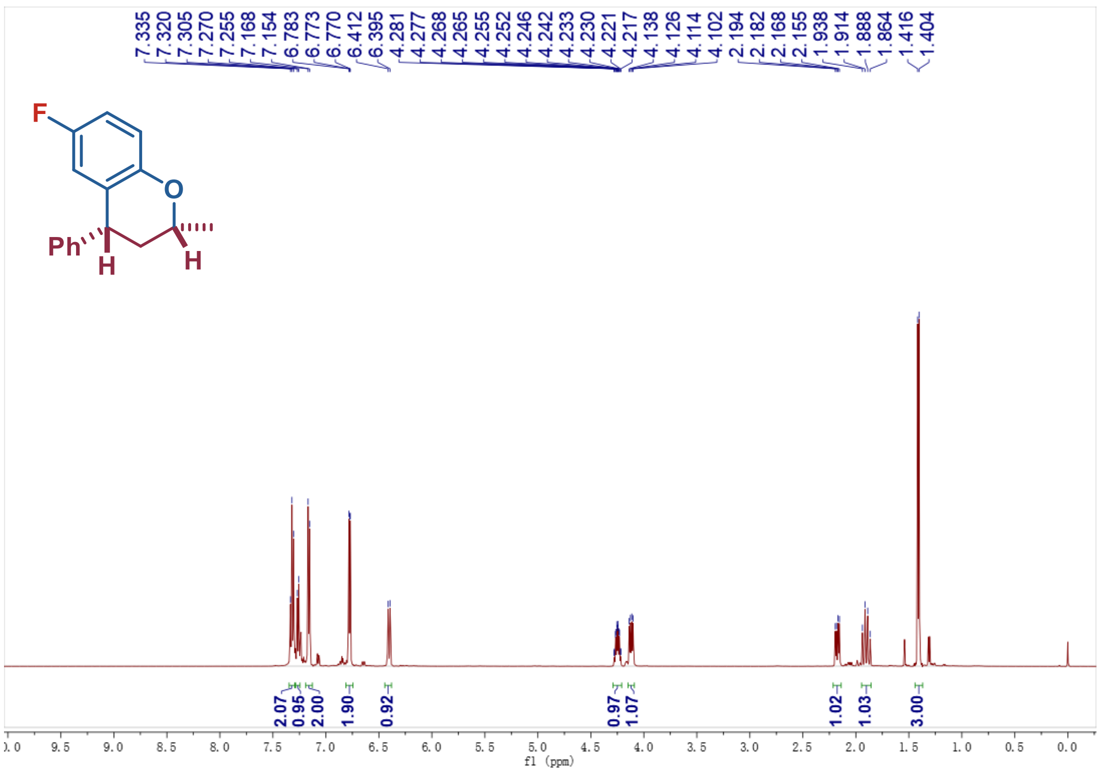

Fig. S116 ^{1}H NMR data of product 4j.

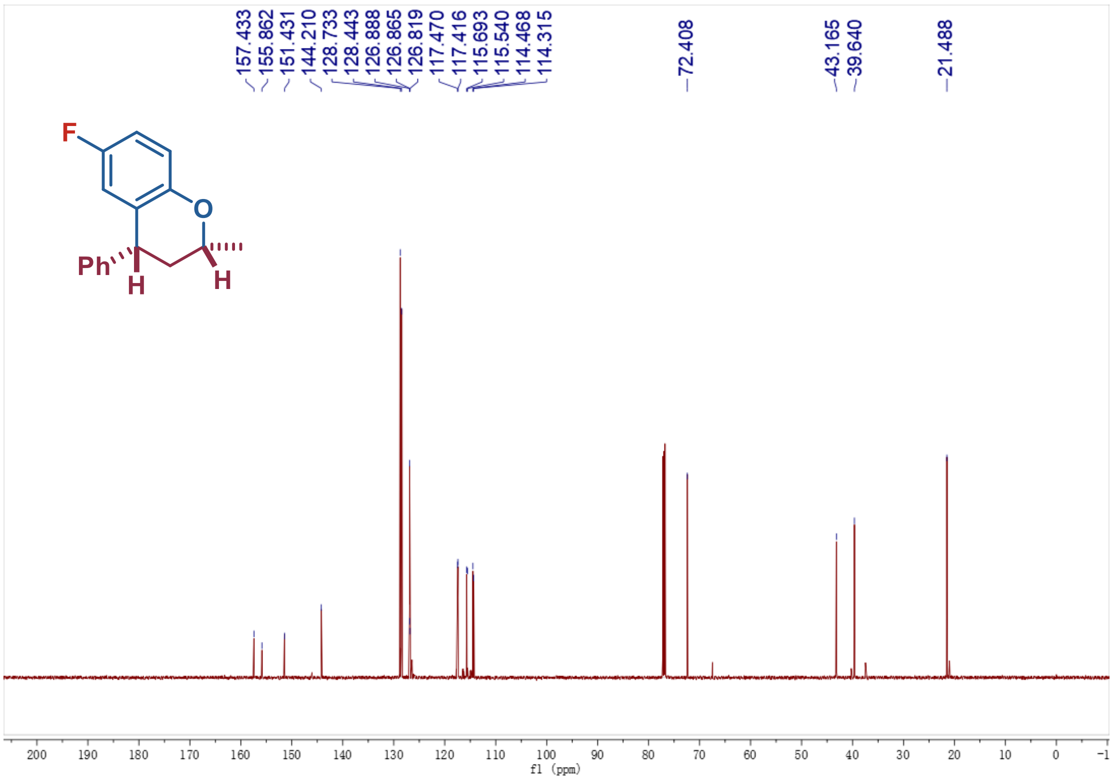

Fig. S117 ^{13}C NMR data of product 4j.

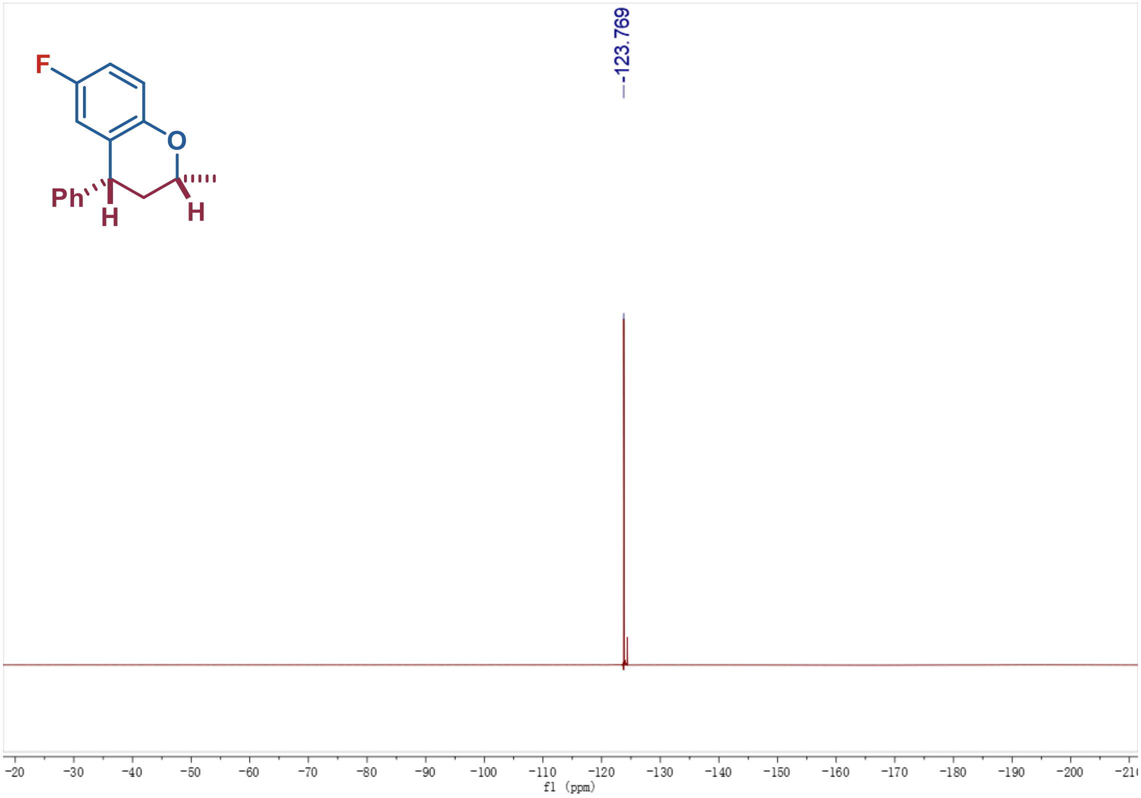

Fig. S118 ^{19}F NMR data of product 4j.

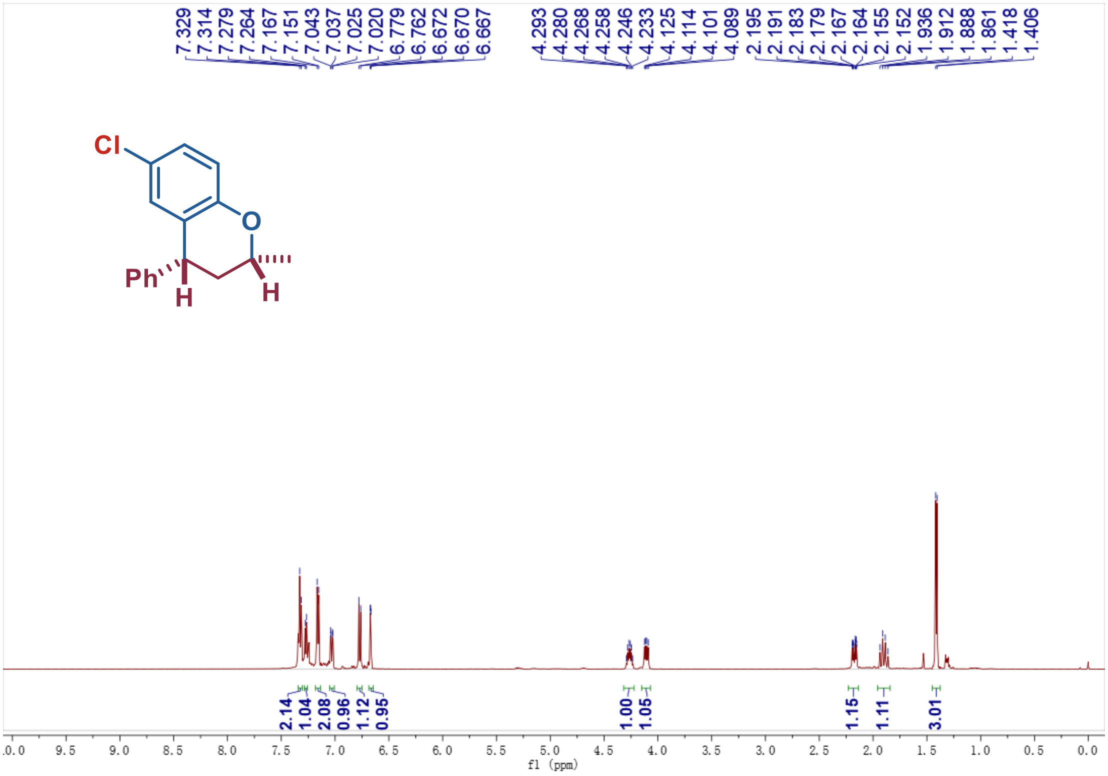

Fig. S119 ^{1}H NMR data of product 4k.

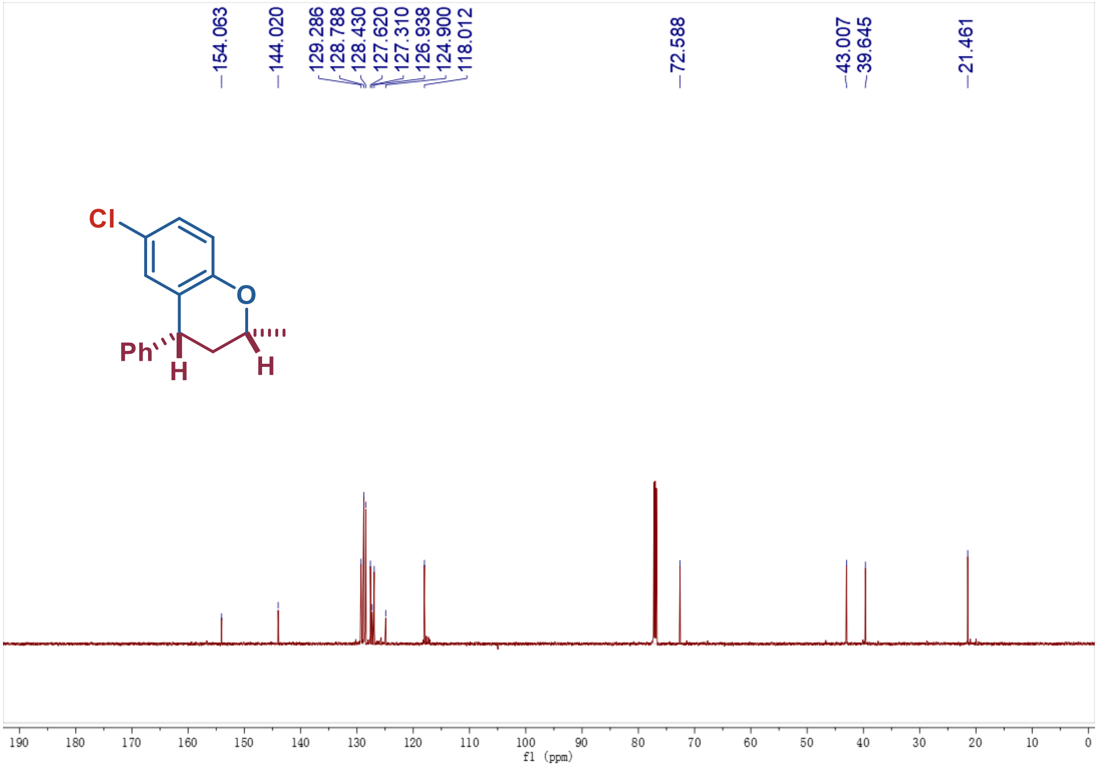

Fig. S120 ^{13}C NMR data of product 4k.

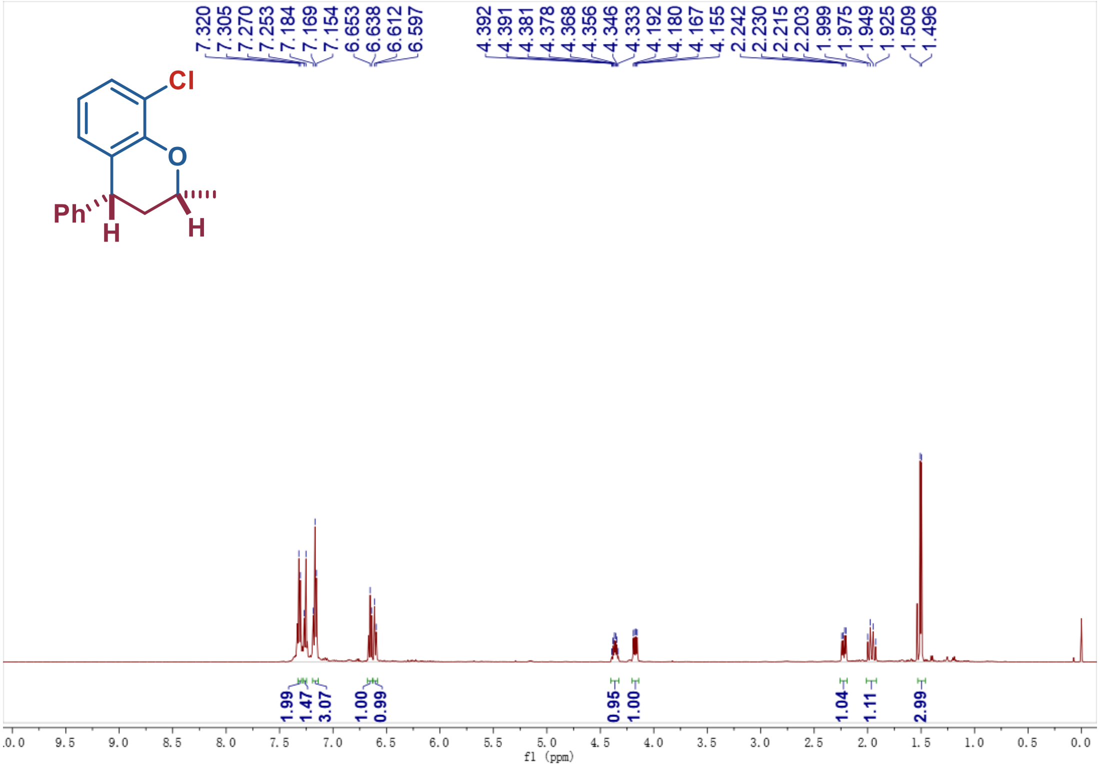

Fig. S121 ^{1}H NMR data of product 4l.

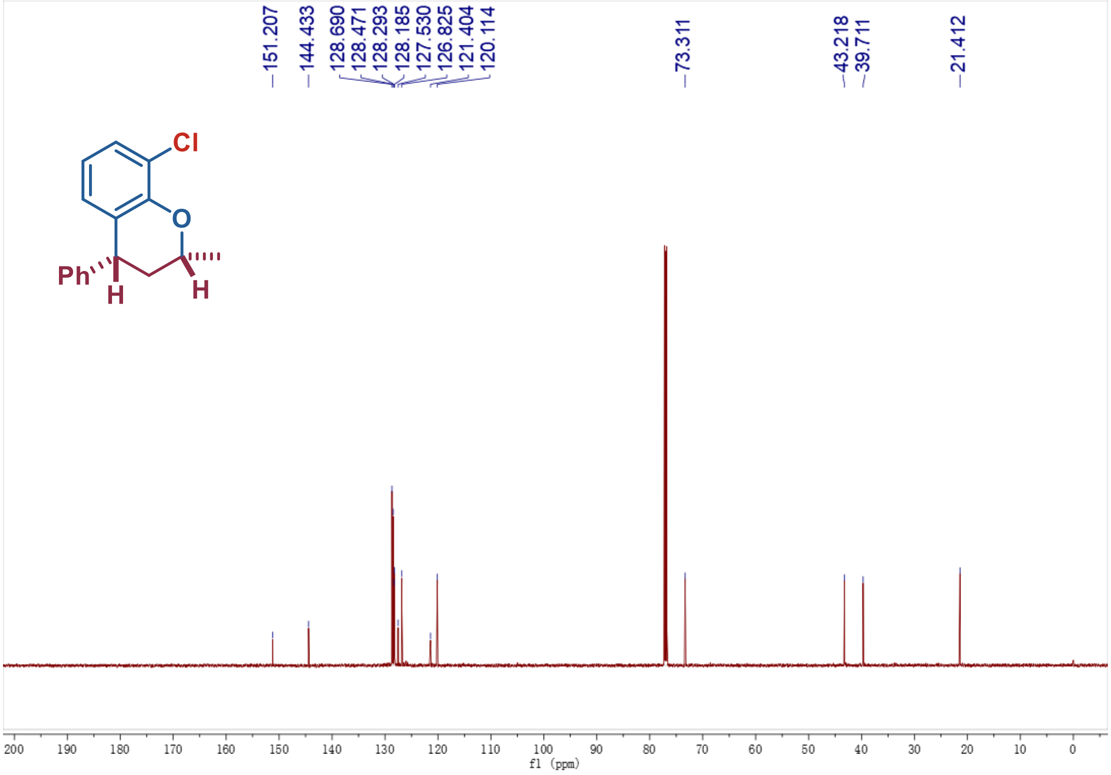

Fig. S122 ^{13}C NMR data of product 4l.

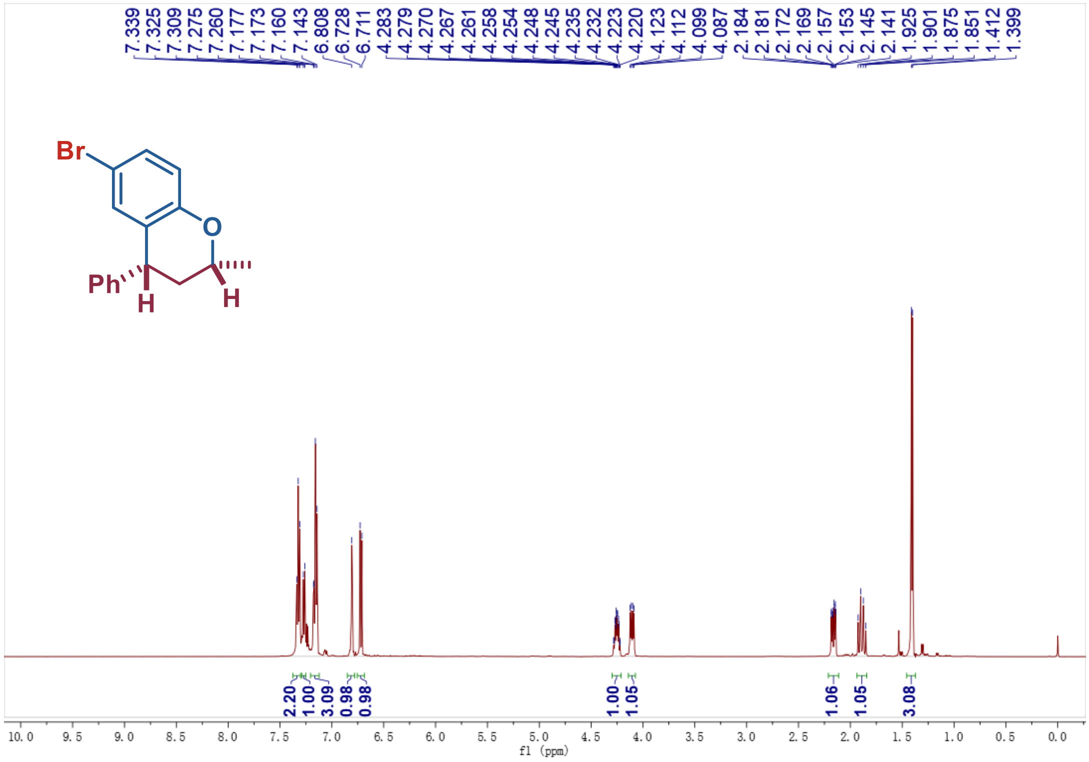

Fig. S123 1H NMR data of product 4m.

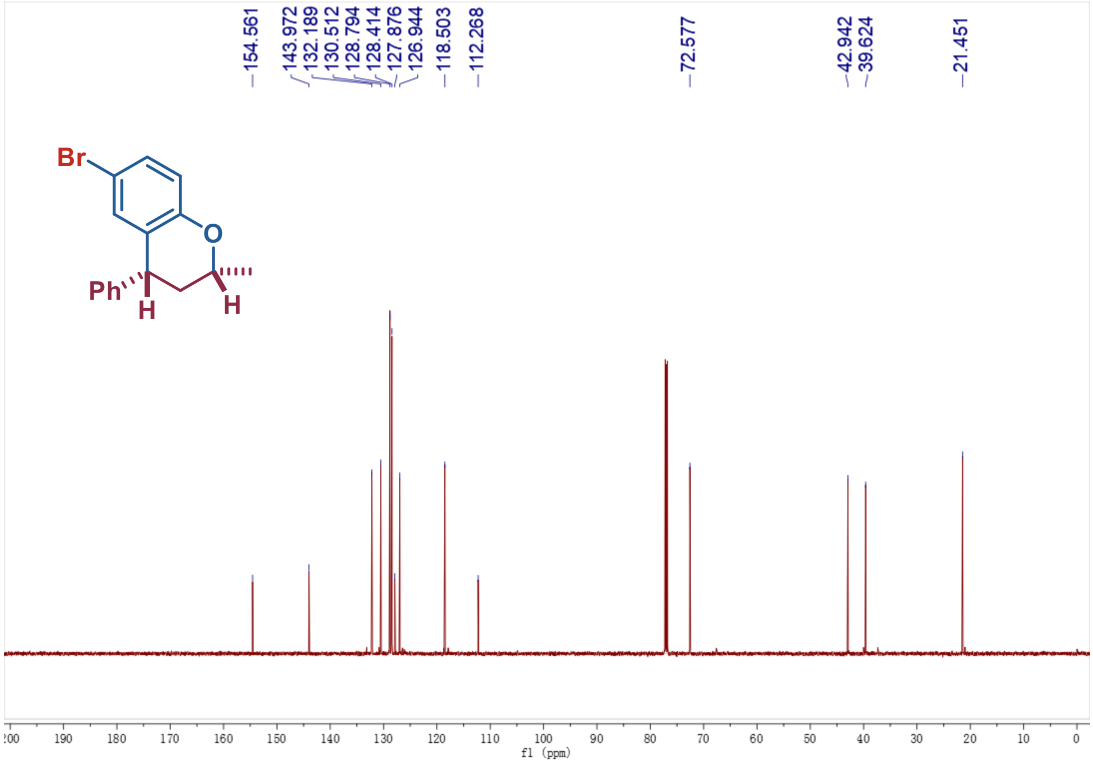

Fig. S124 13C NMR data of product 4m.

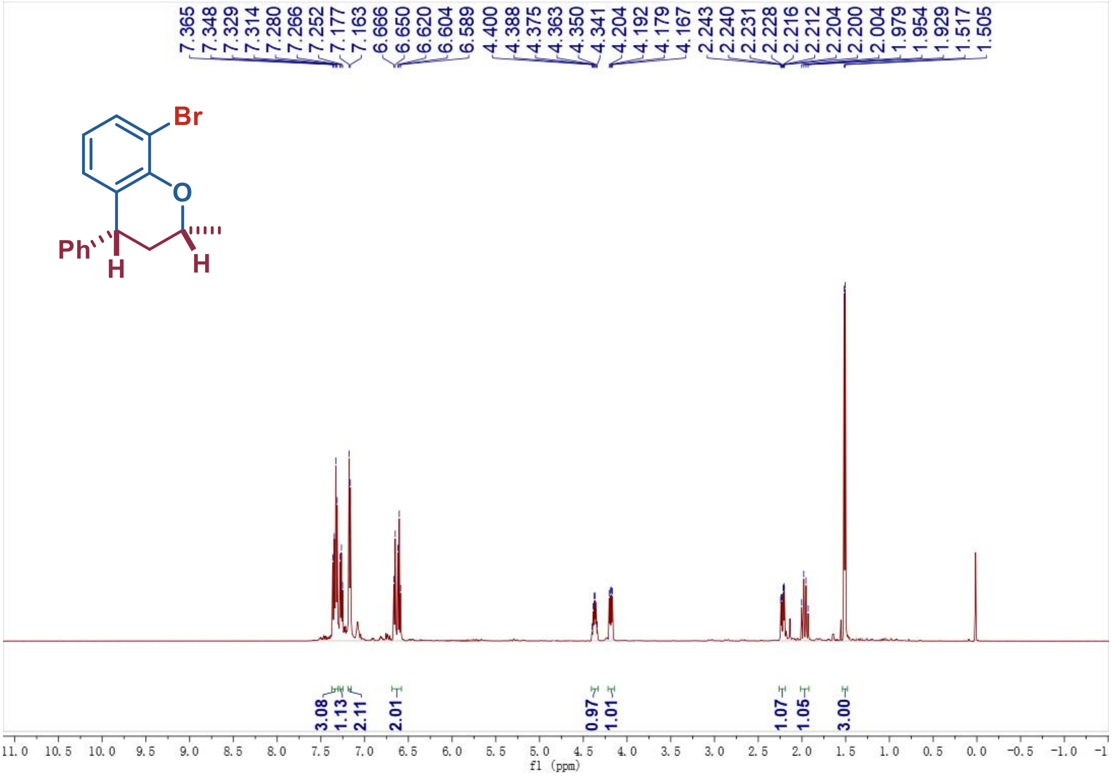

Fig. S125 ^{1}H NMR data of product 4n.

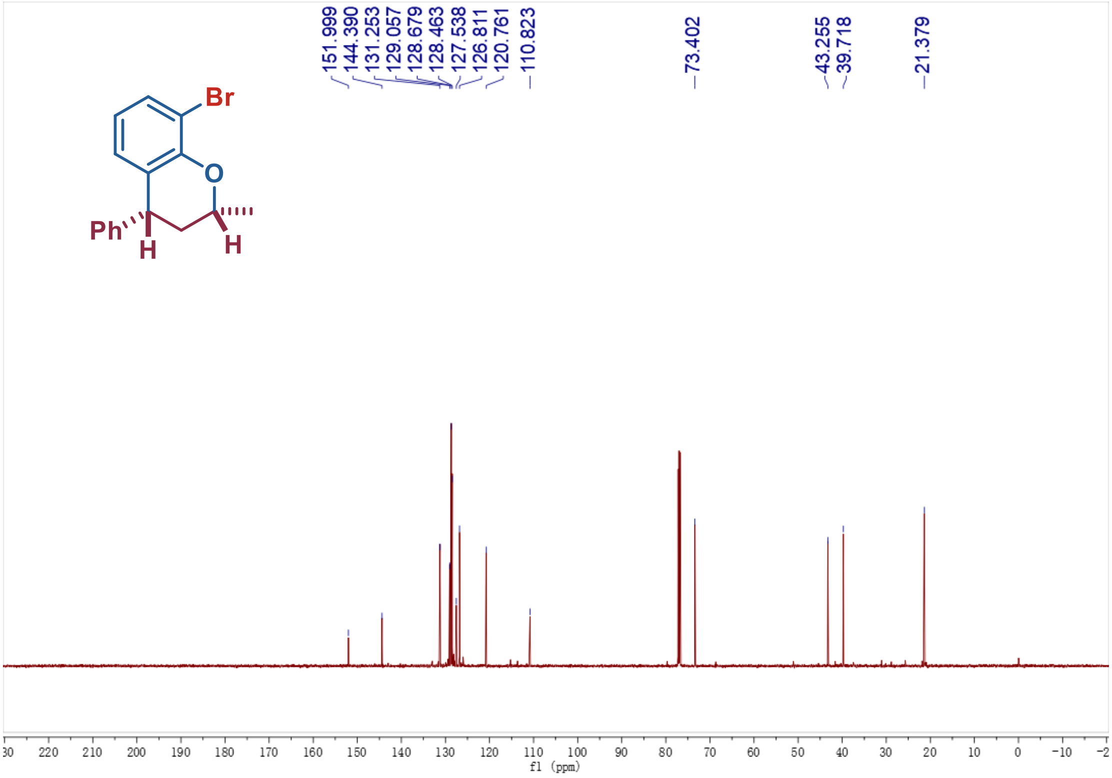

Fig. S126 ^{13}C NMR data of product 4n.

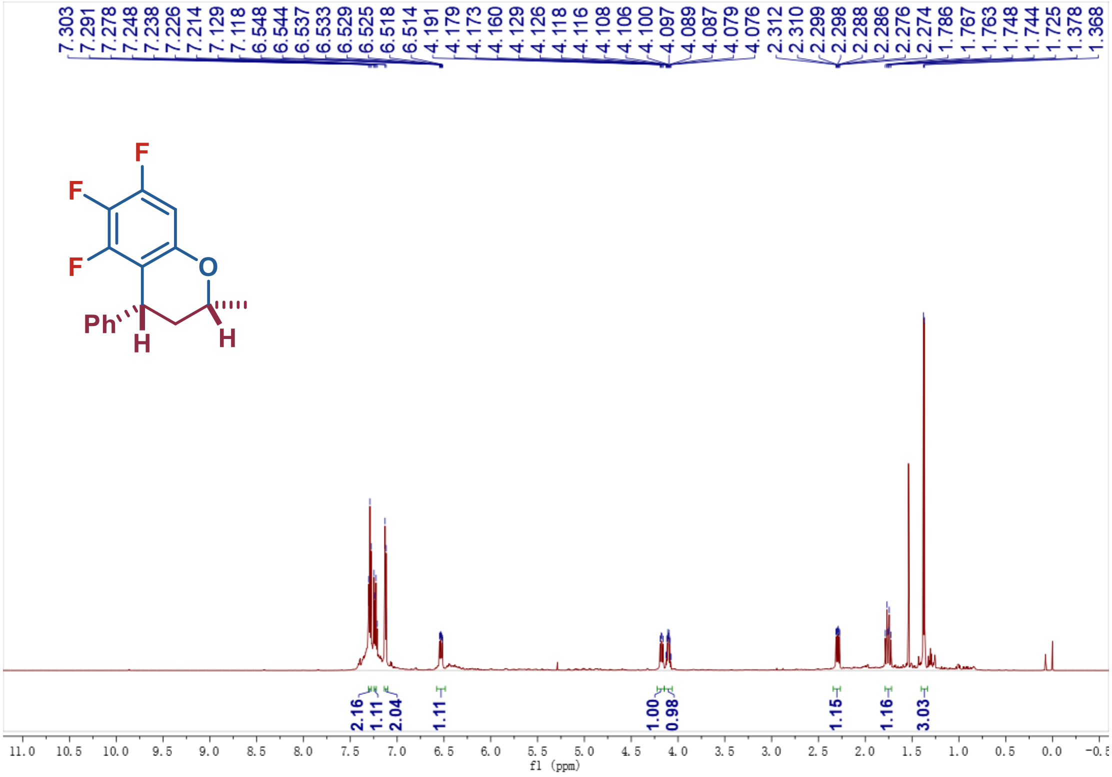

Fig. S127 ^{1}H NMR data of product 4o.

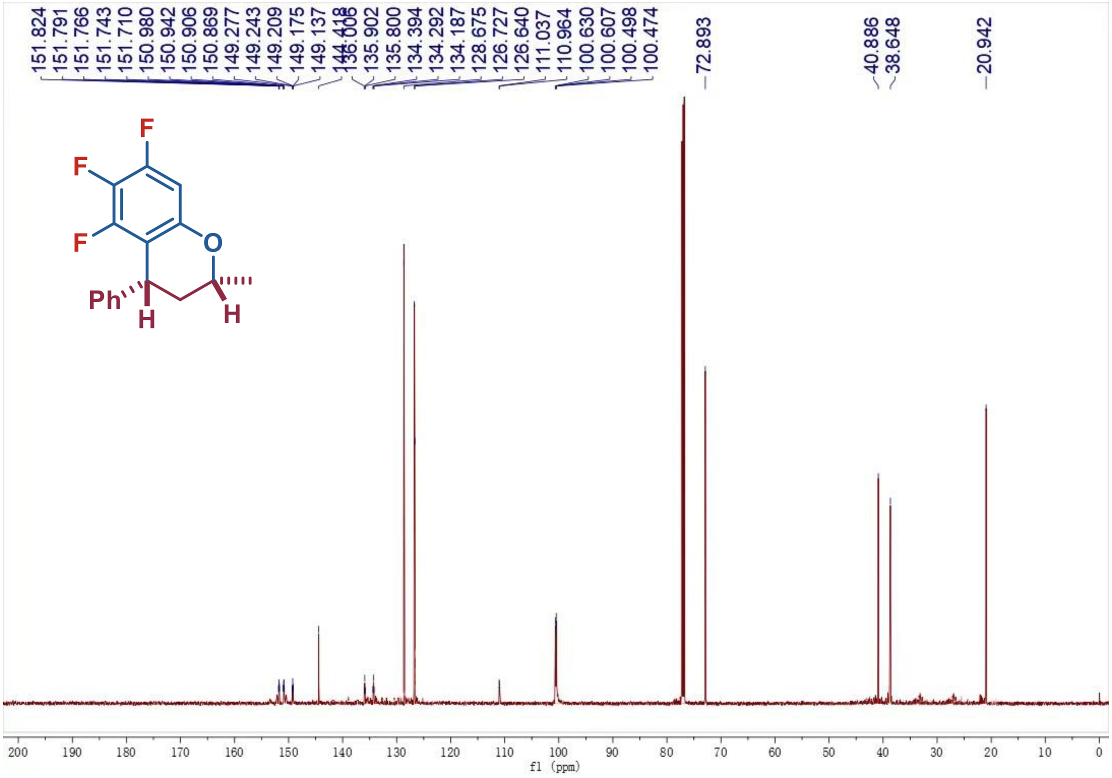

Fig. S128 ^{13}C NMR data of product 4o.

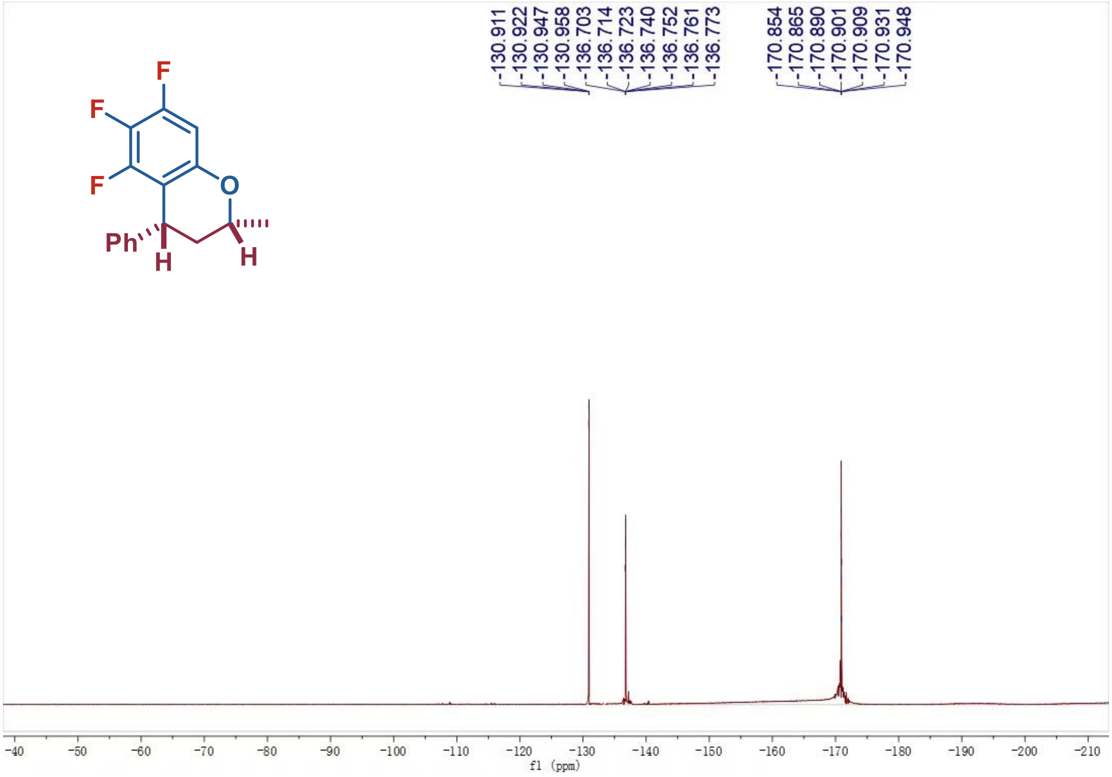

Fig. S129 ^{19}F NMR data of product 4o.

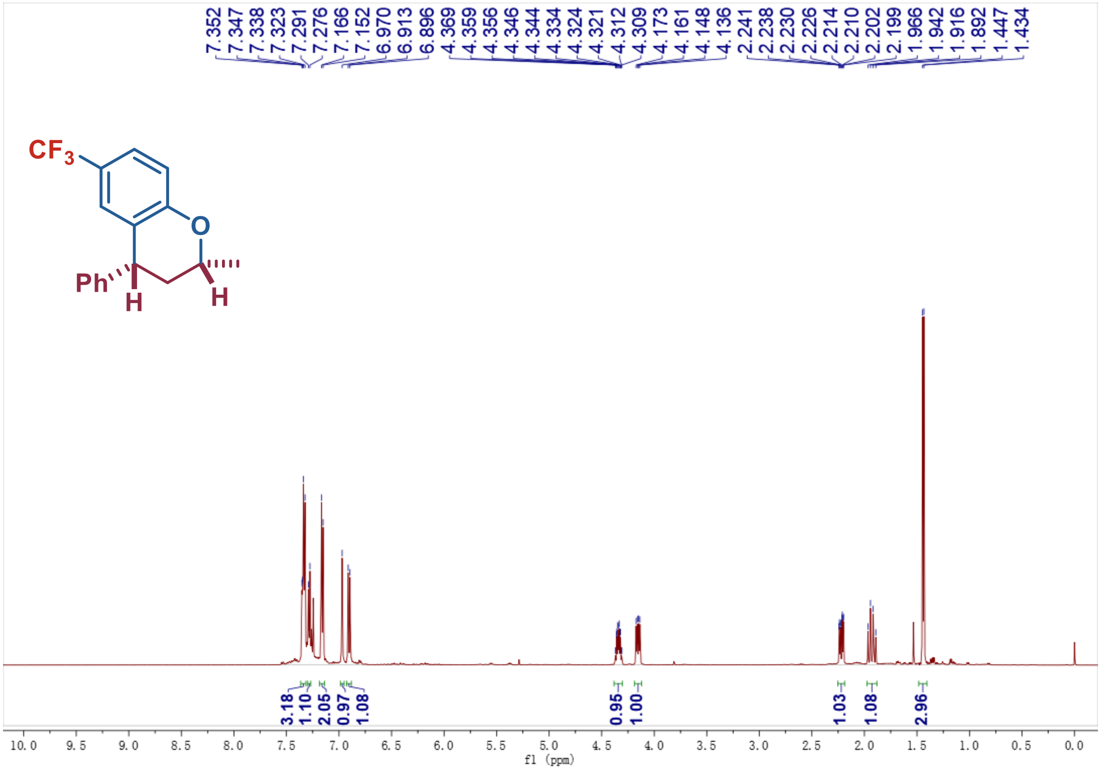

Fig. S130 ^{1}H NMR data of product 4p.

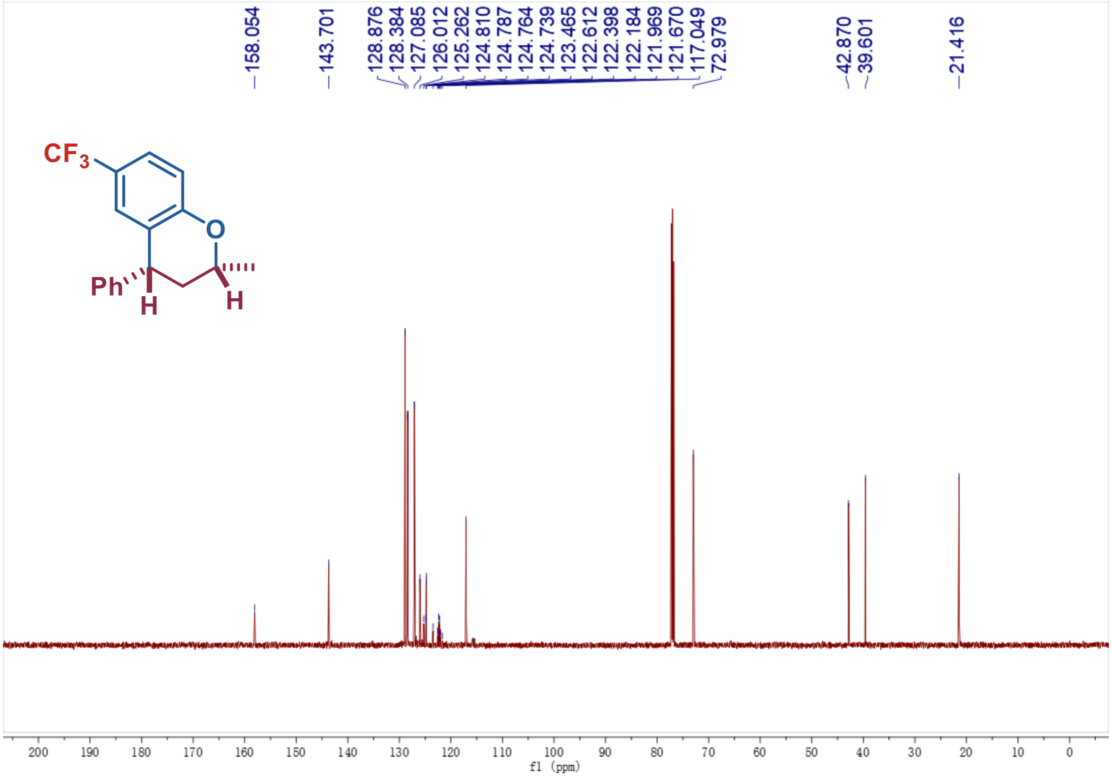

Fig. S131 ^{13}C NMR data of product 4p.

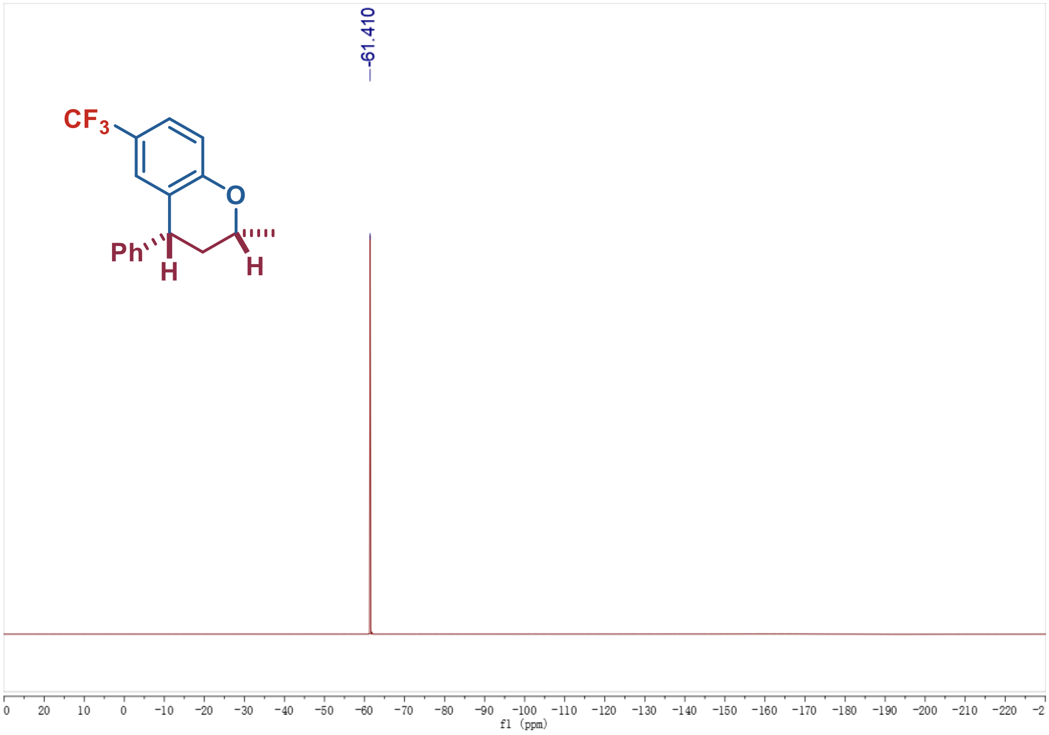

Fig. S132 ^{19}F NMR data of product 4p.

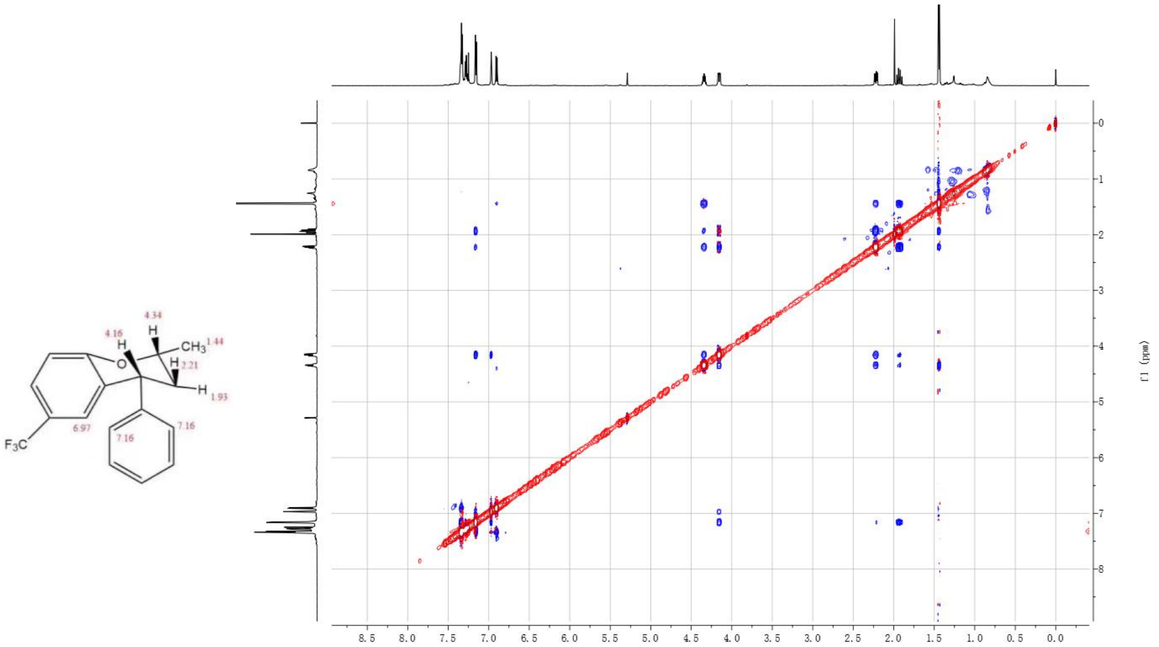

Fig. S133 Noesy data of product 4p.

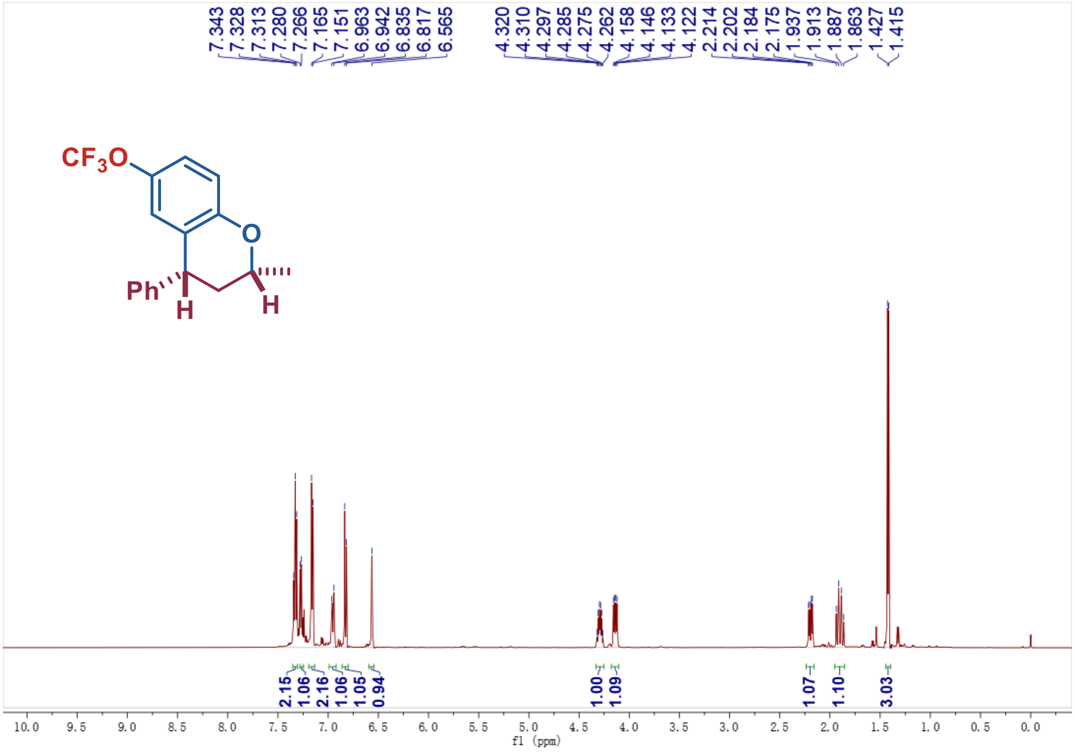

Fig. S134 ¹H NMR data of product 4q.

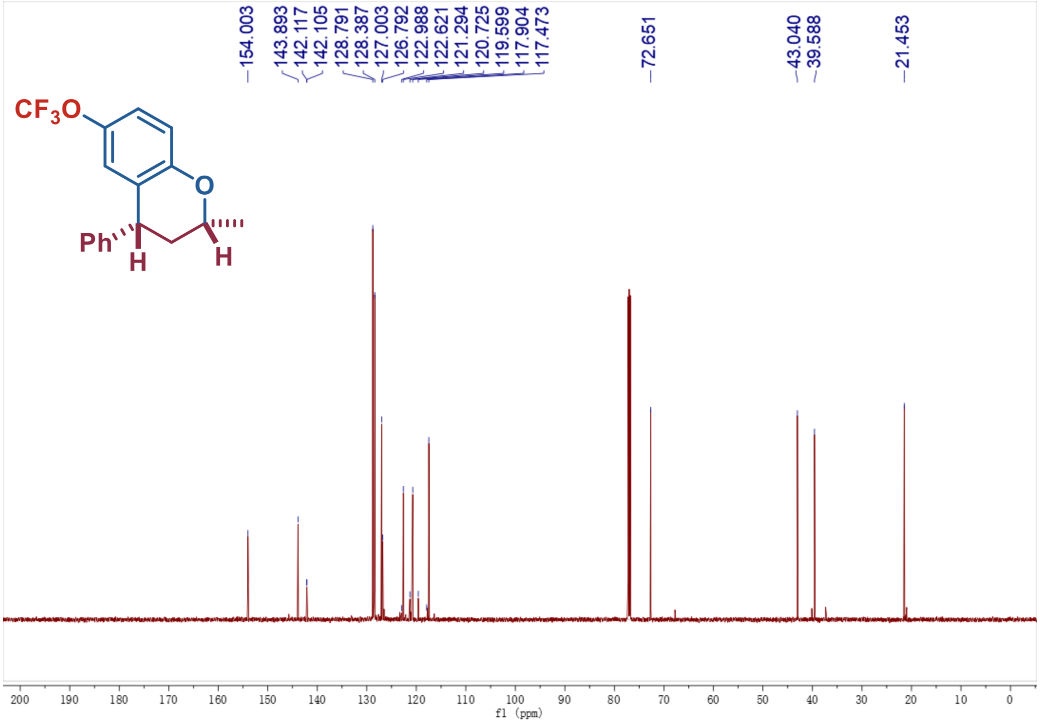

Fig. S135 ¹³C NMR data of product 4q.

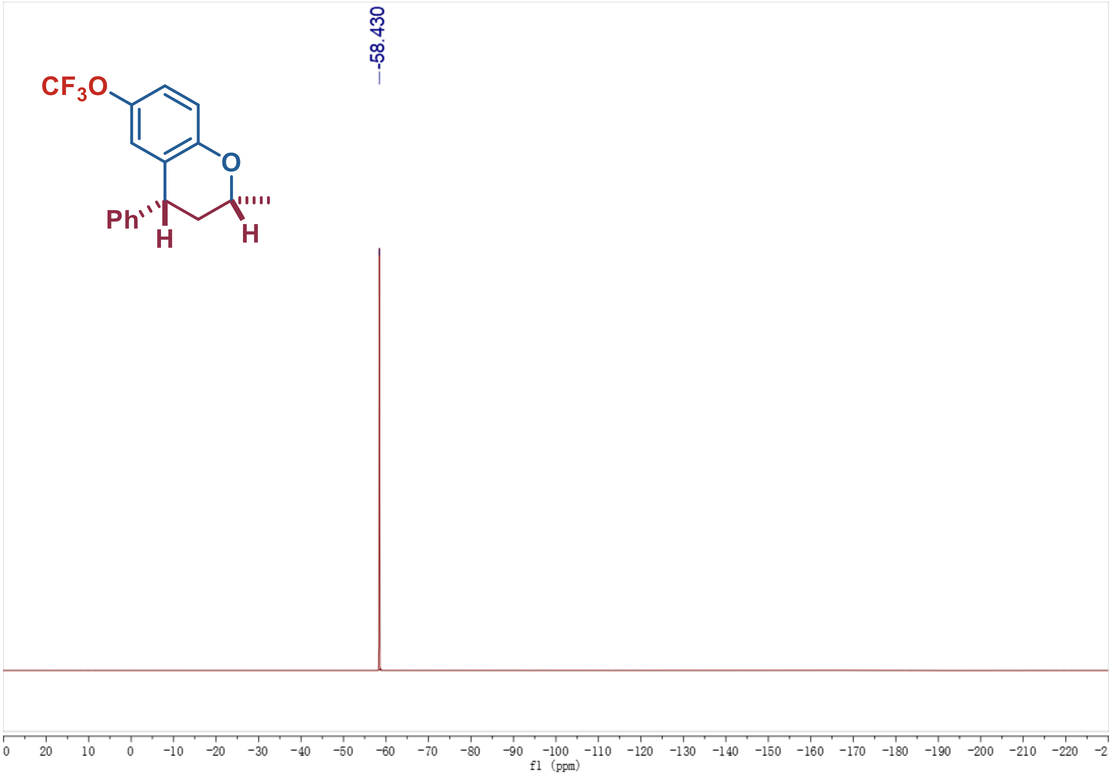

Fig. S136 ^{19}F NMR data of product 4q.

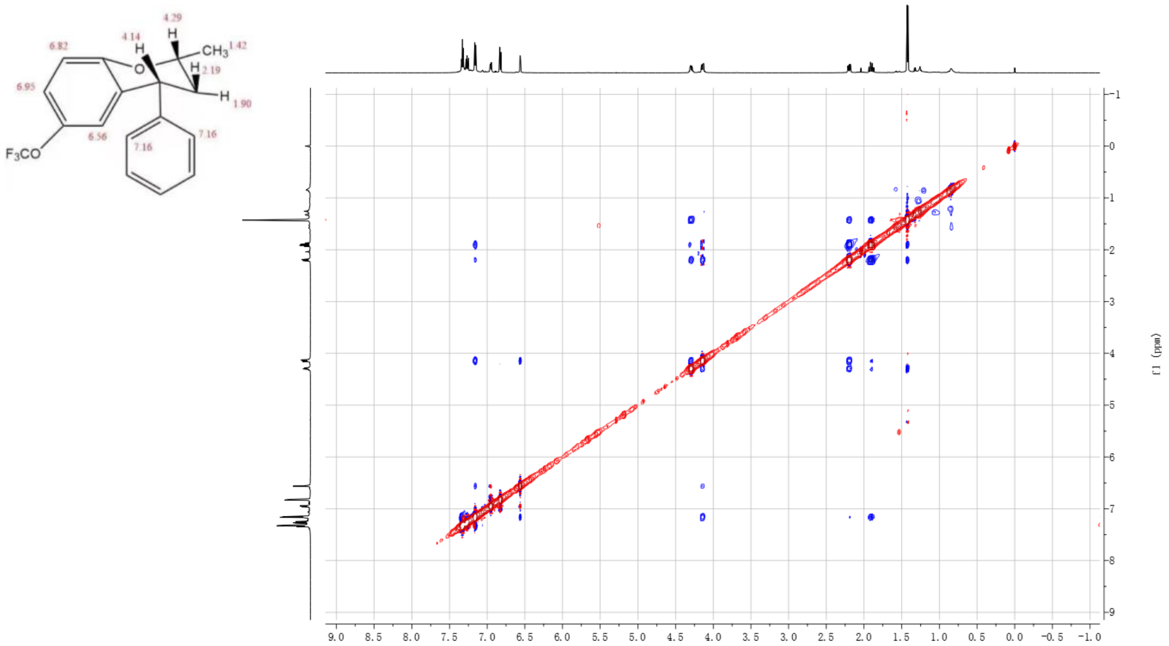

Fig. S137 Noesy data of product 4q.

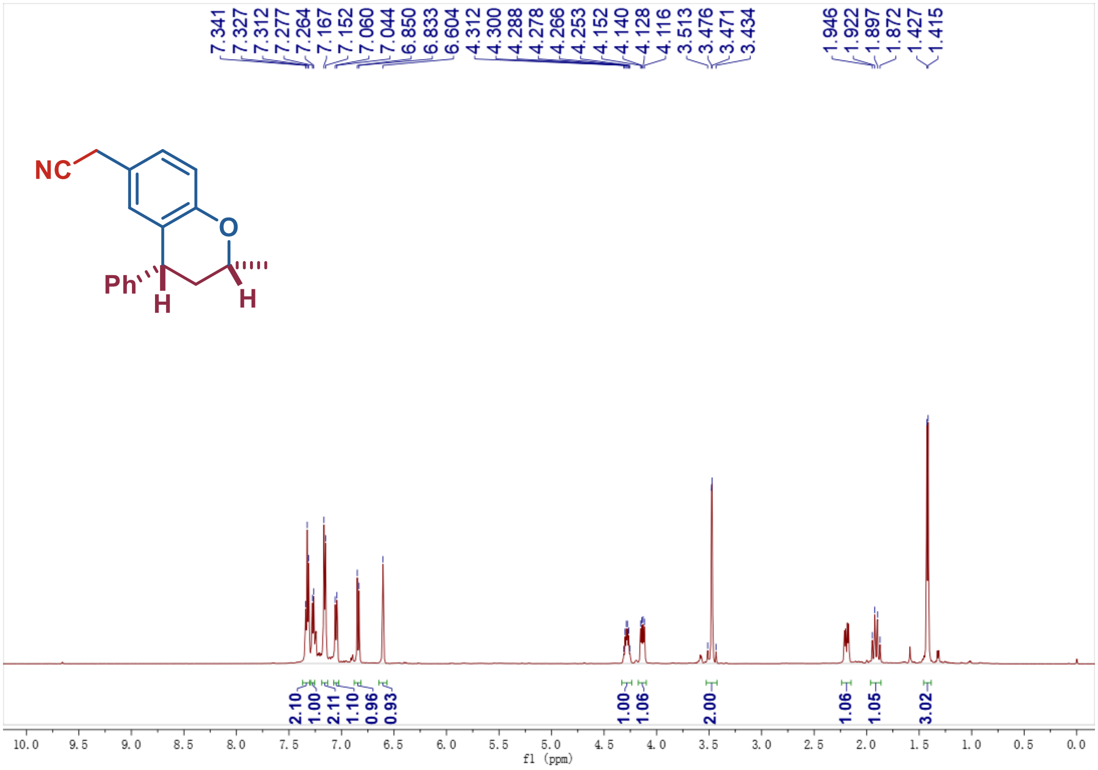

Fig. S138 ^{1}H NMR data of product 4r.

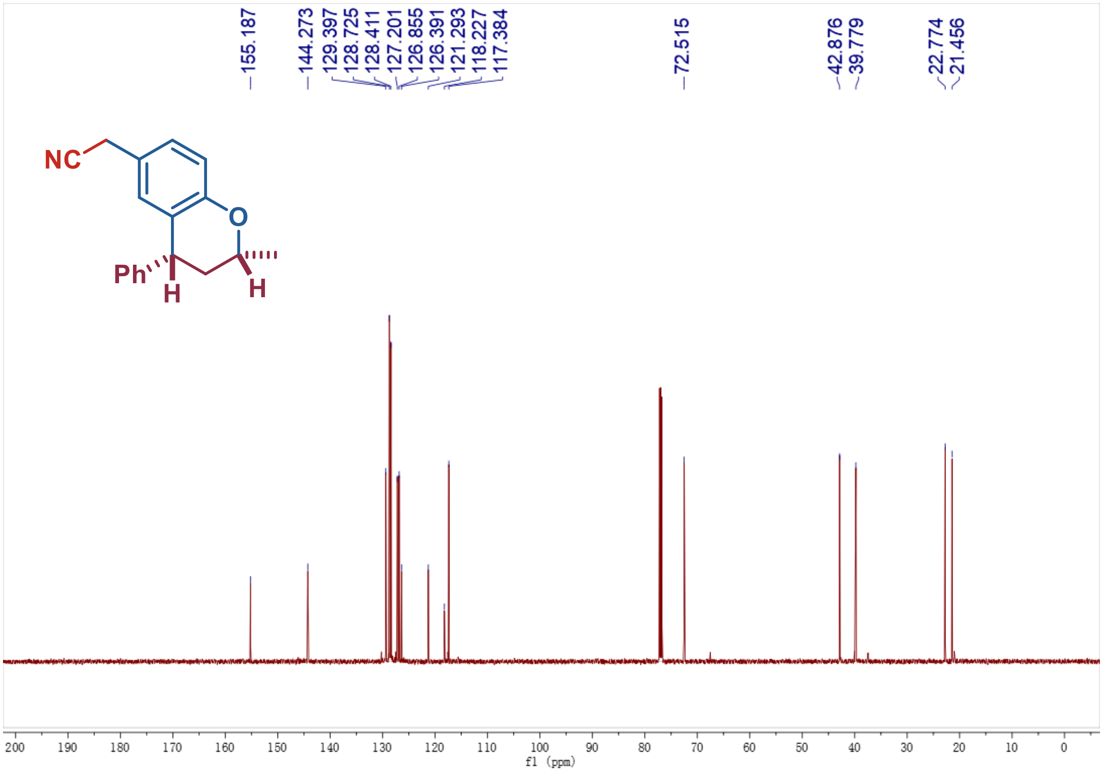

Fig. S139 ^{13}C NMR data of product 4r.

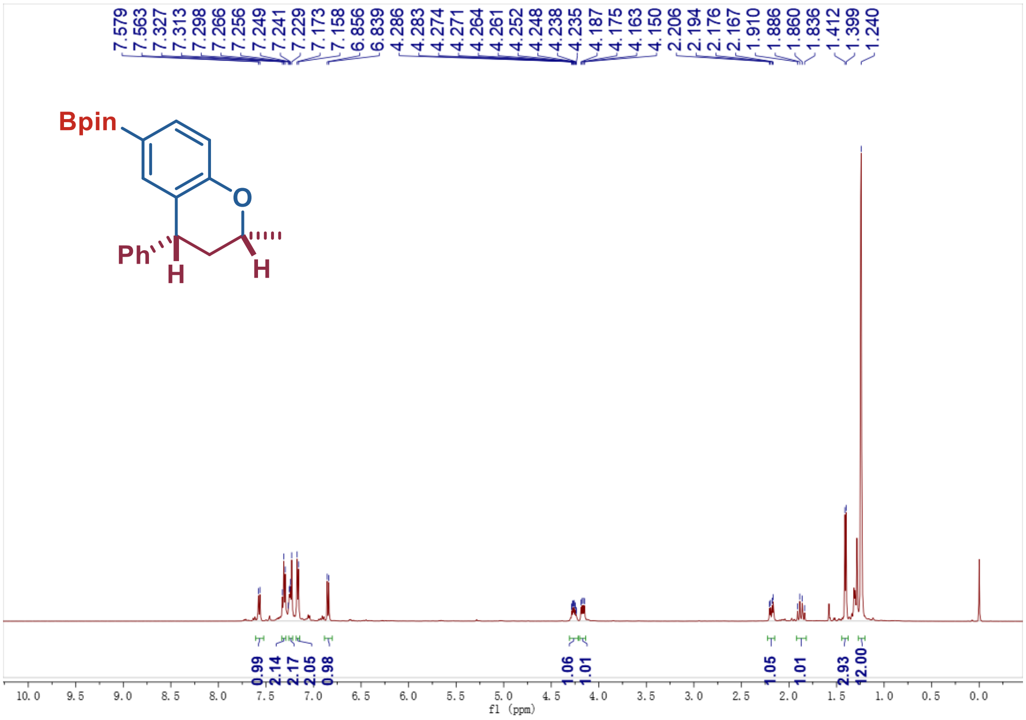

Fig. S140 ^{1}H NMR data of product 4s.

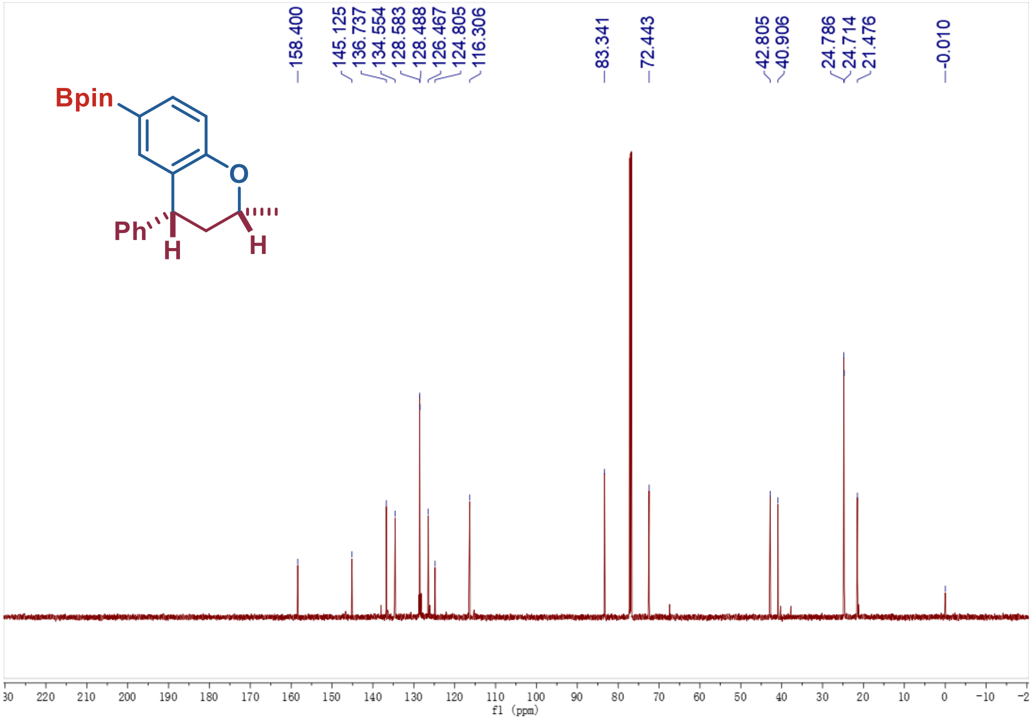

Fig. S141 ^{13}C NMR data of product 4s.

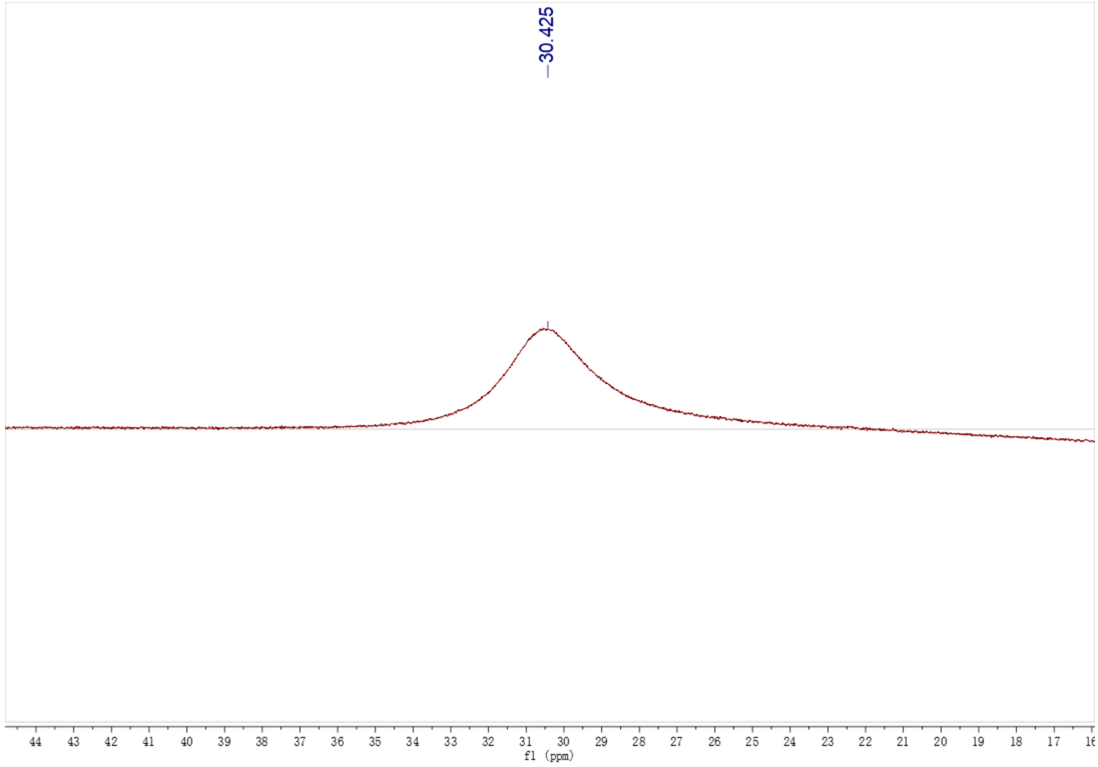

Fig. S142 ^{11}B NMR data of product 4s.

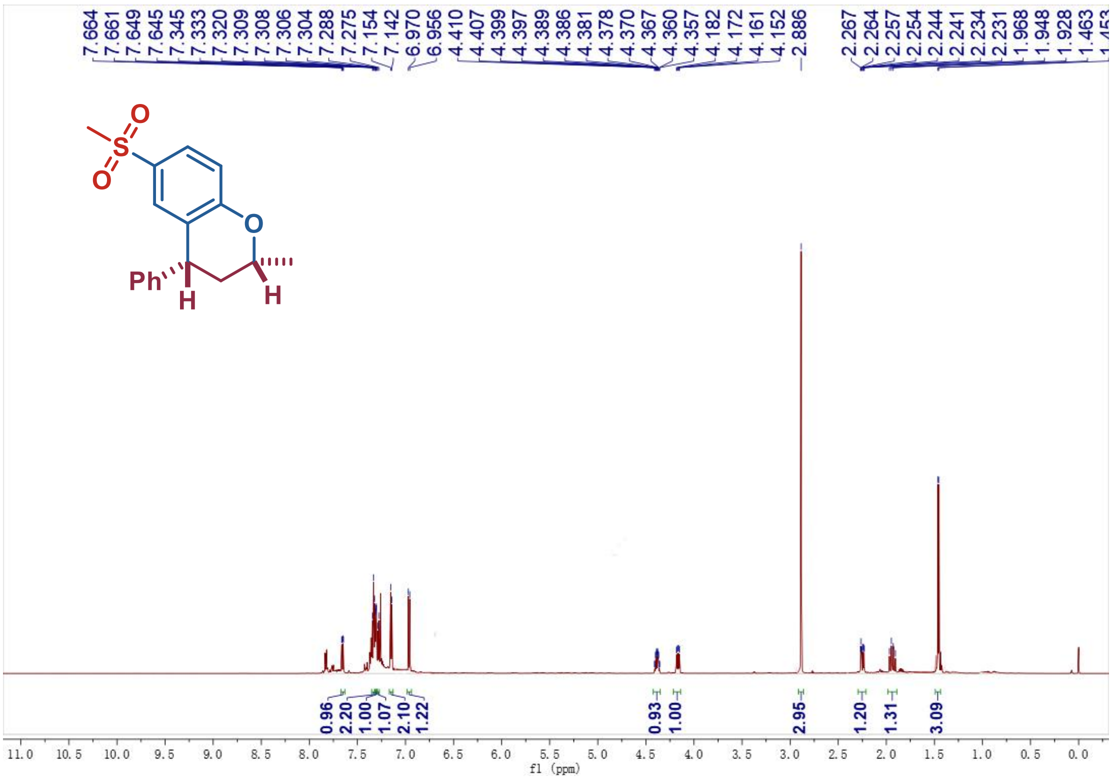

Fig. S143 ^{1}H NMR data of product 4t.

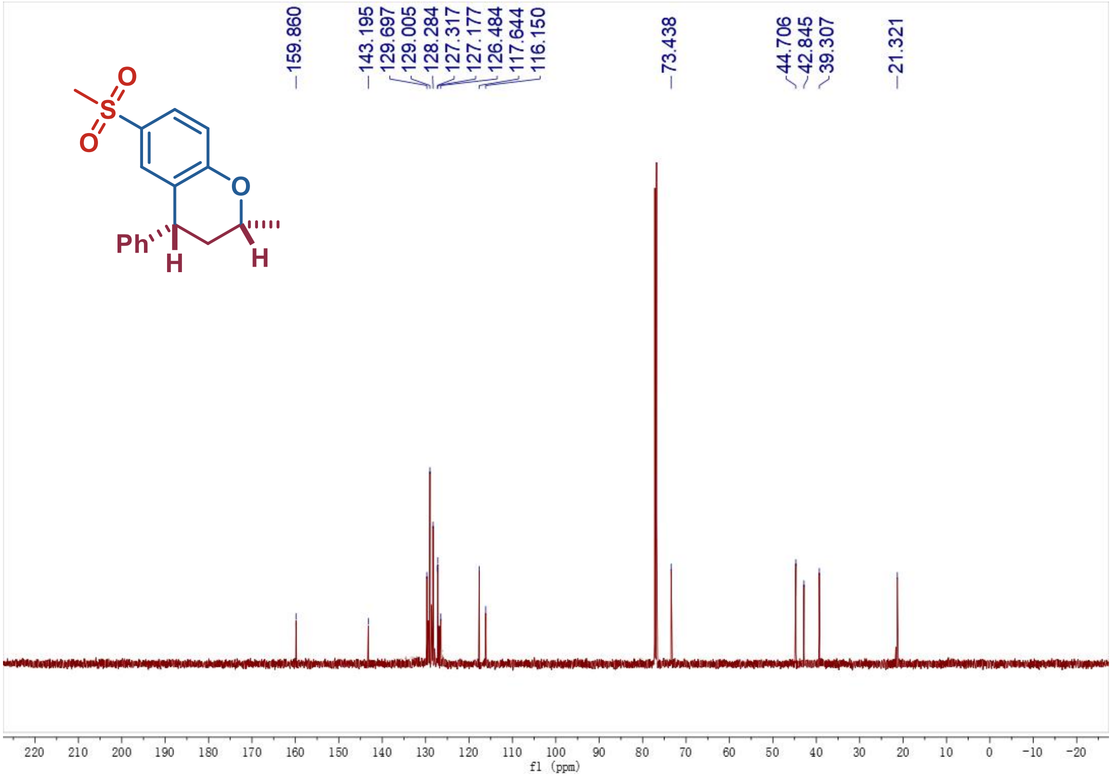

Fig. S144 ^{13}C NMR data of product 4t.

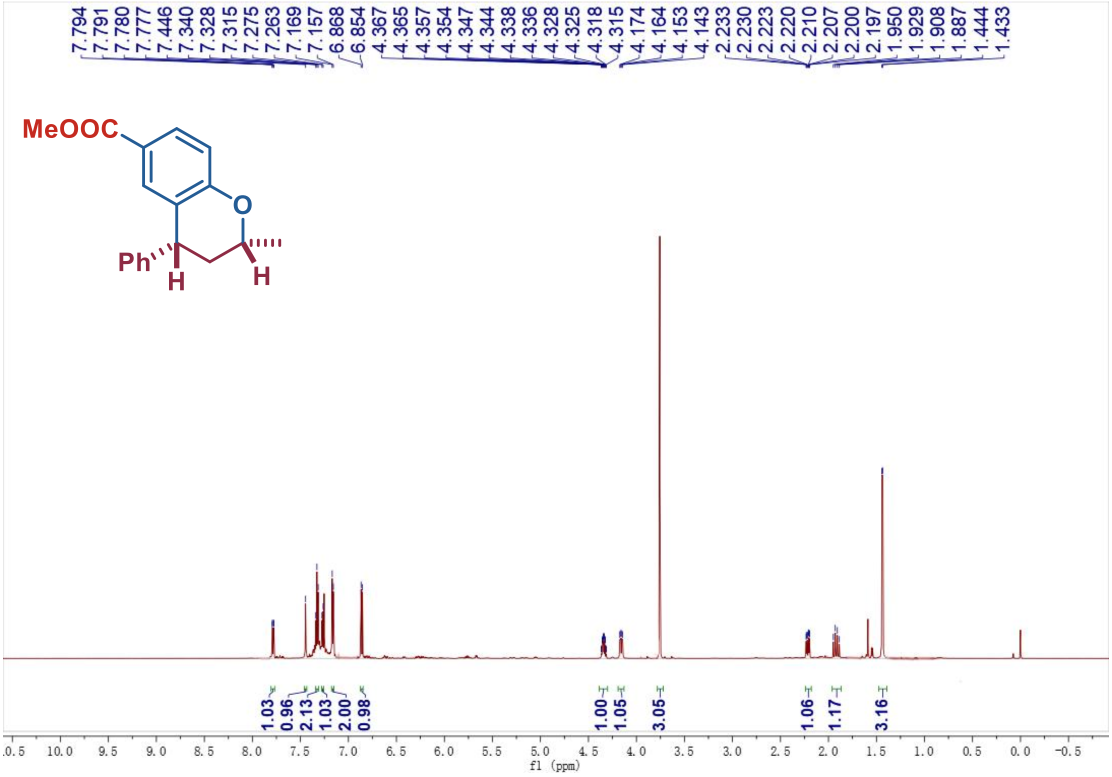

Fig. S145 ^{1}H NMR data of product 4u.

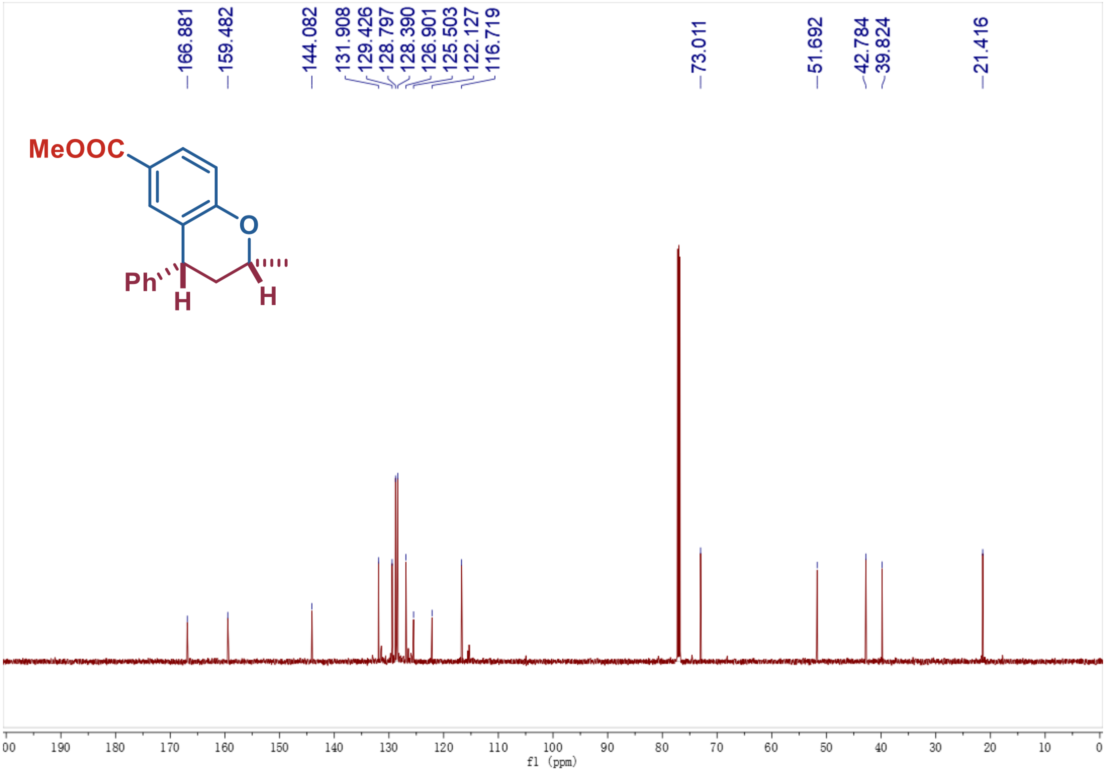

Fig. S146 ^{13}C NMR data of product 4u.

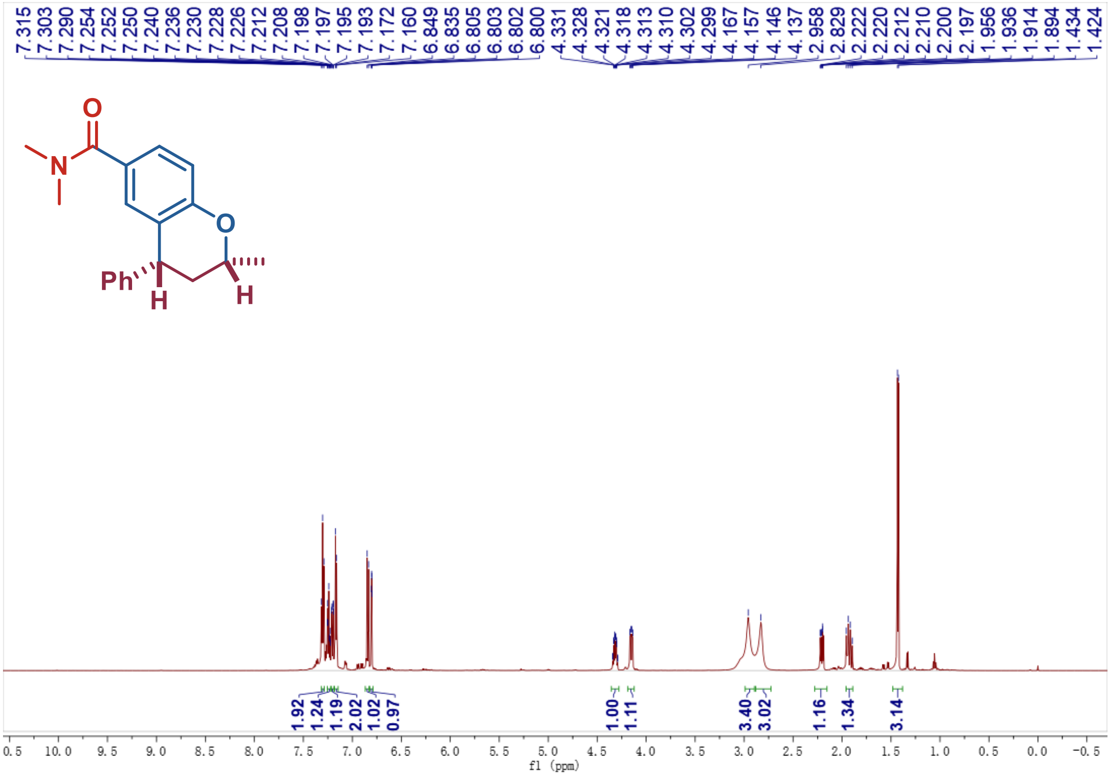

Fig. S147 ^{1}H NMR data of product 4v.

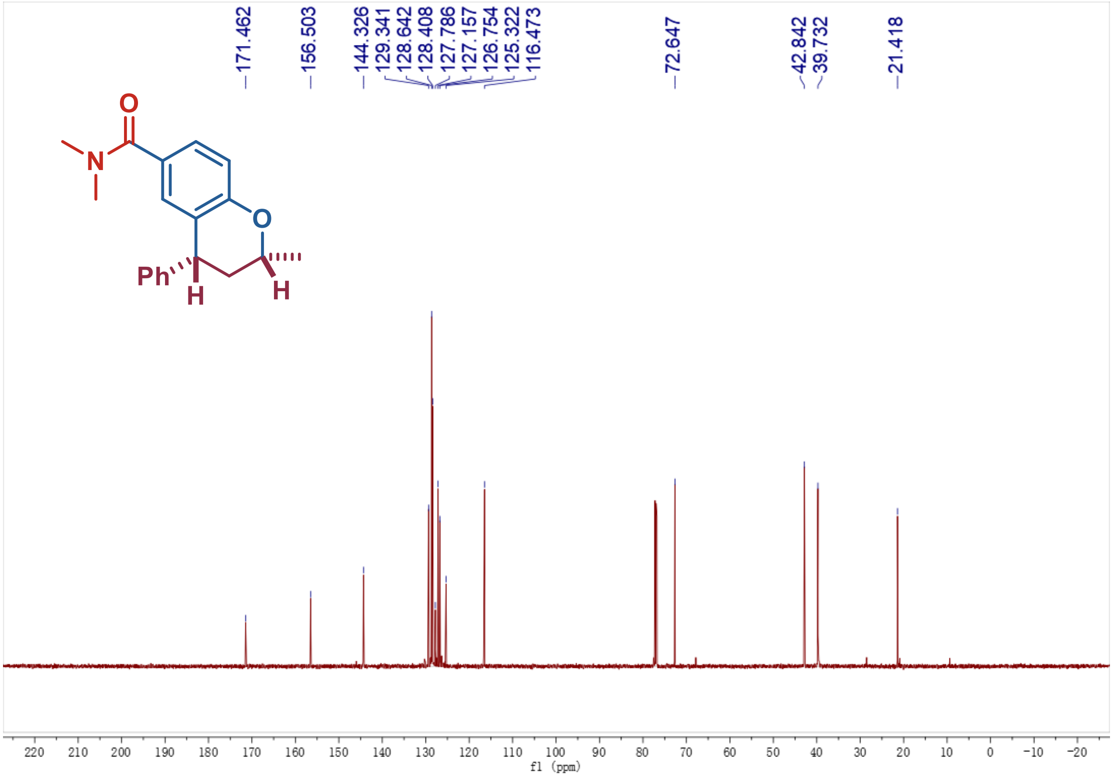

Fig. S148 ^{13}C NMR data of product 4v.

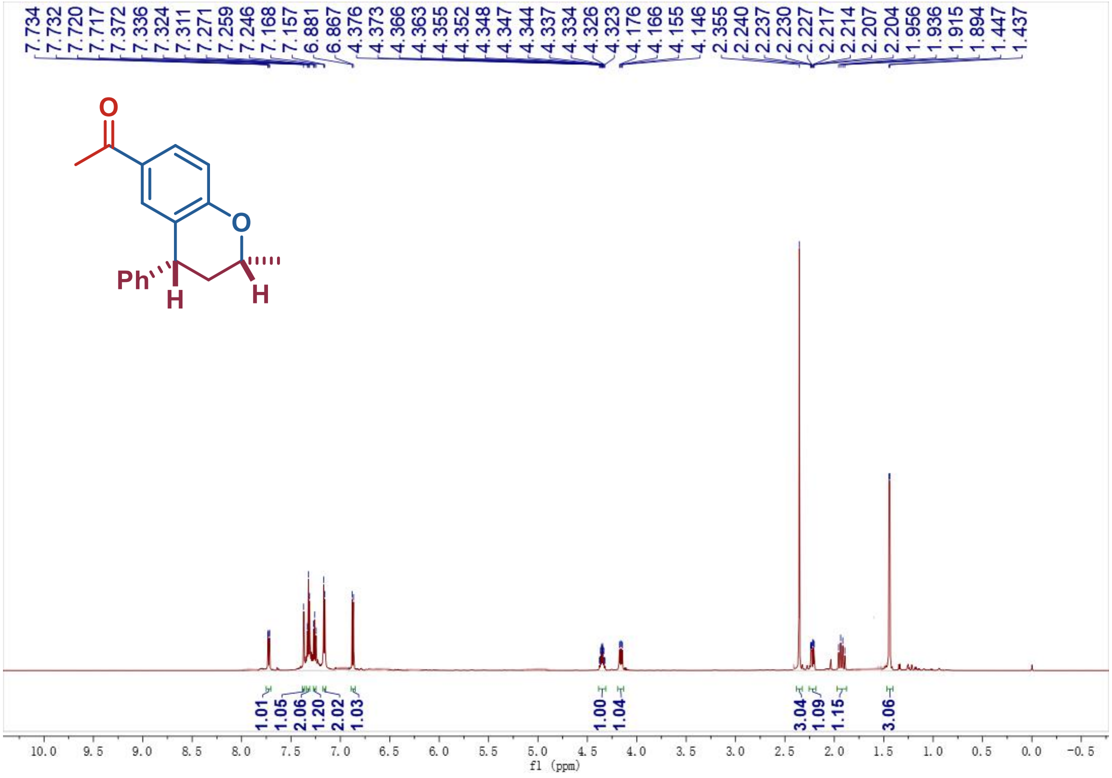

Fig. S149 ^{1}H NMR data of product 4w.

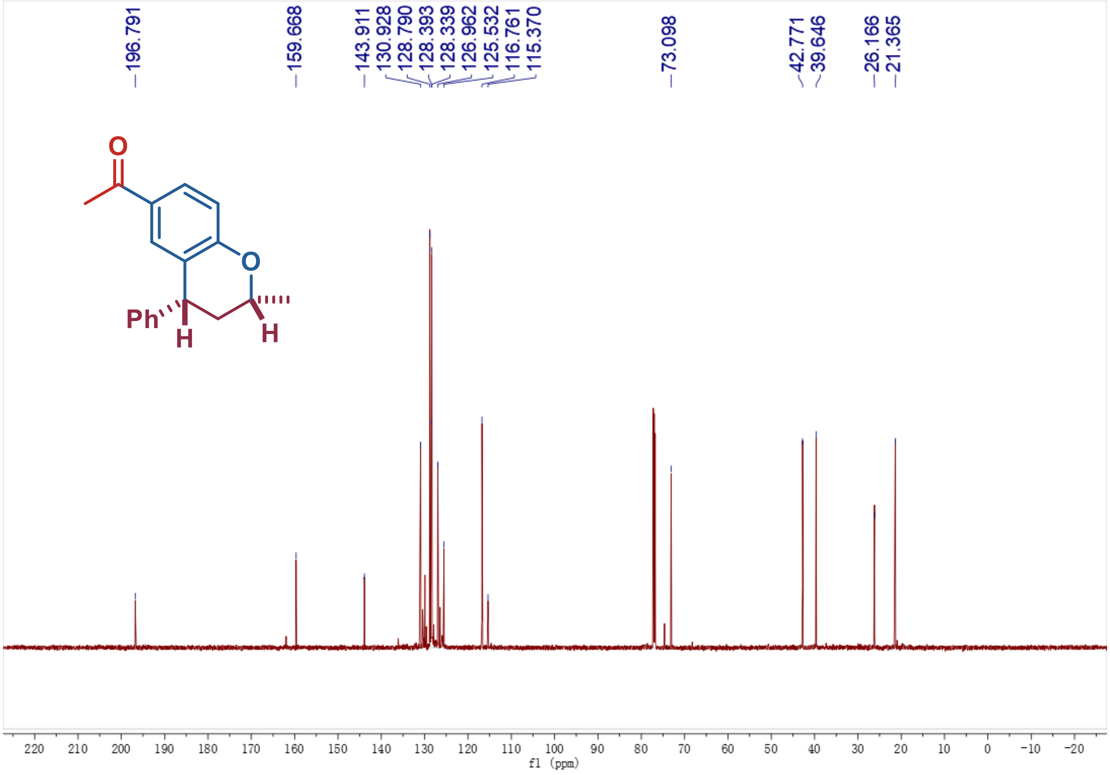

Fig. S150 ^{13}C NMR data of product 4w.

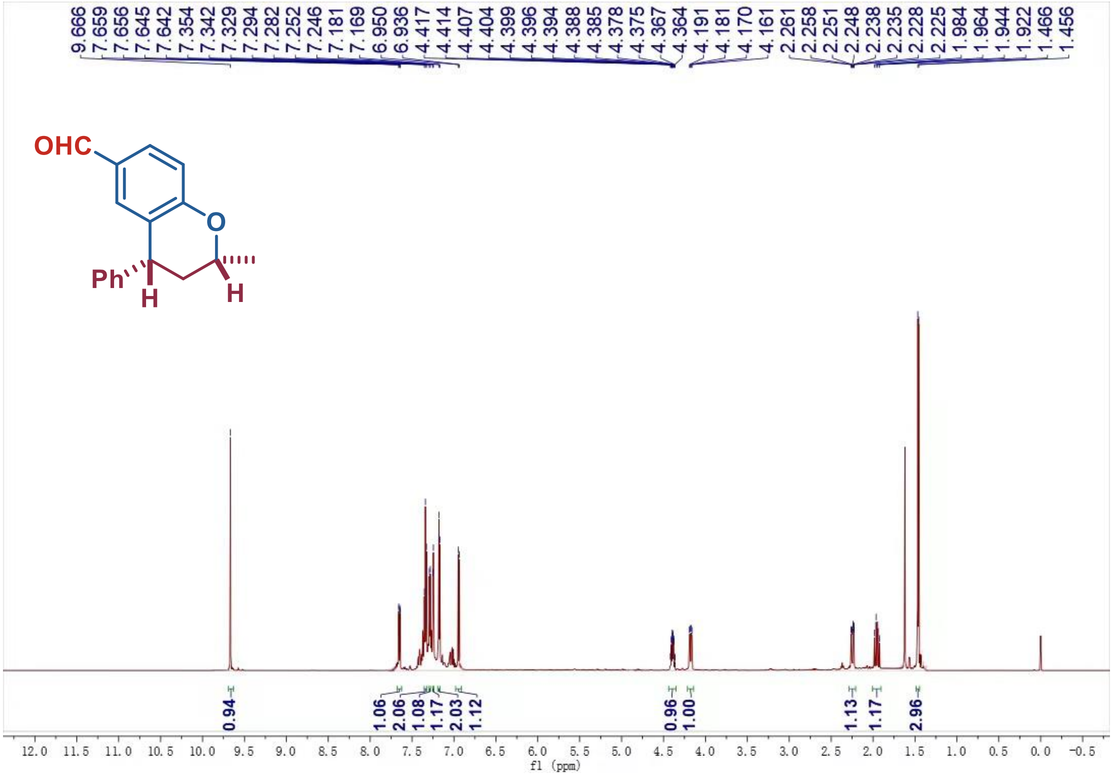

Fig. S151 ^{1}H NMR data of product 4x.

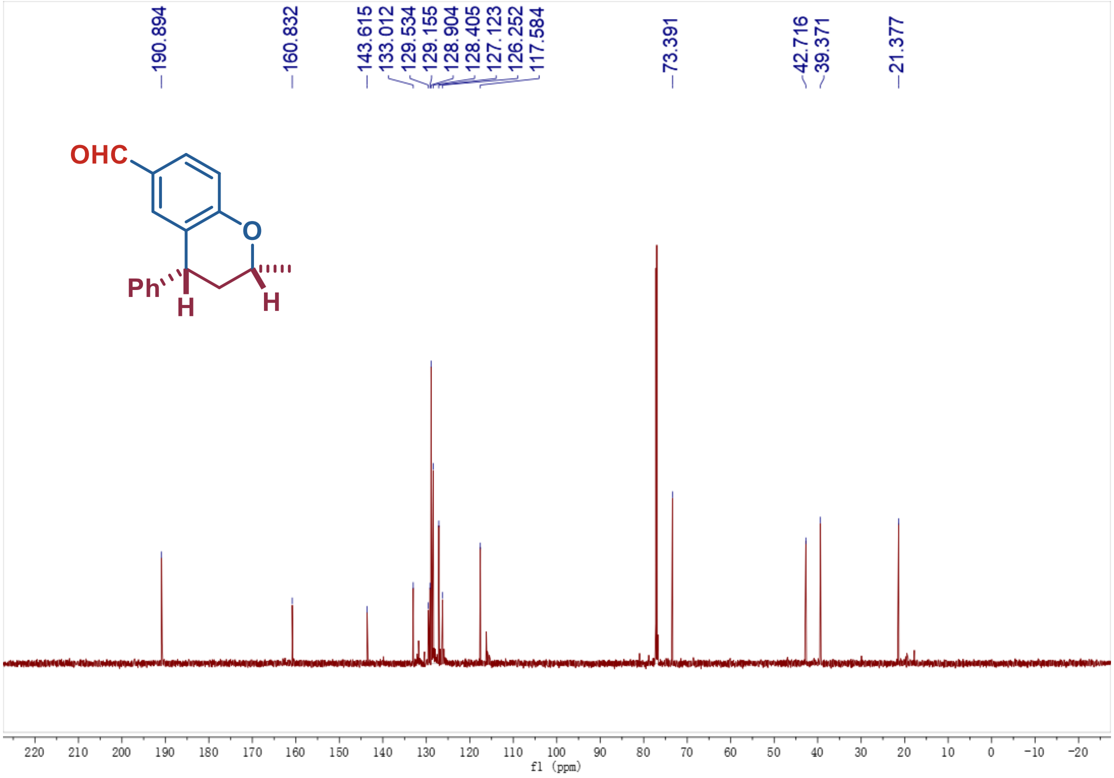

Fig. S152 ^{13}C NMR data of product 4x.

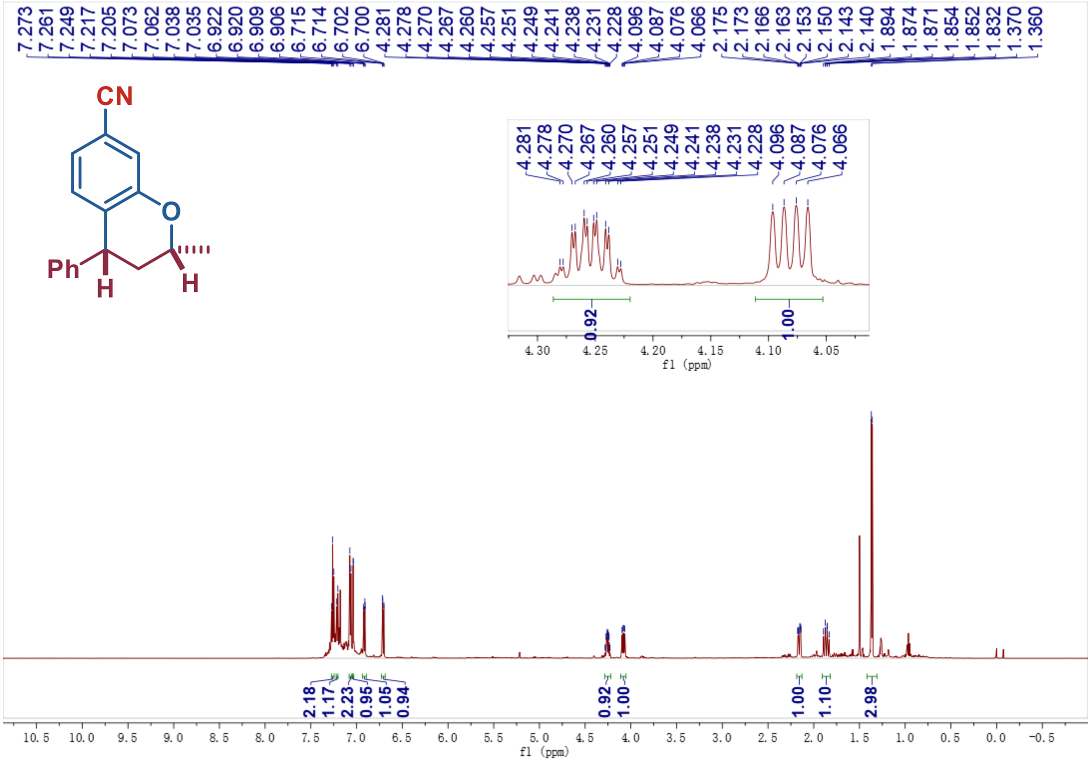

Fig. S153 ^{1}H NMR data of product *trans*-4y.

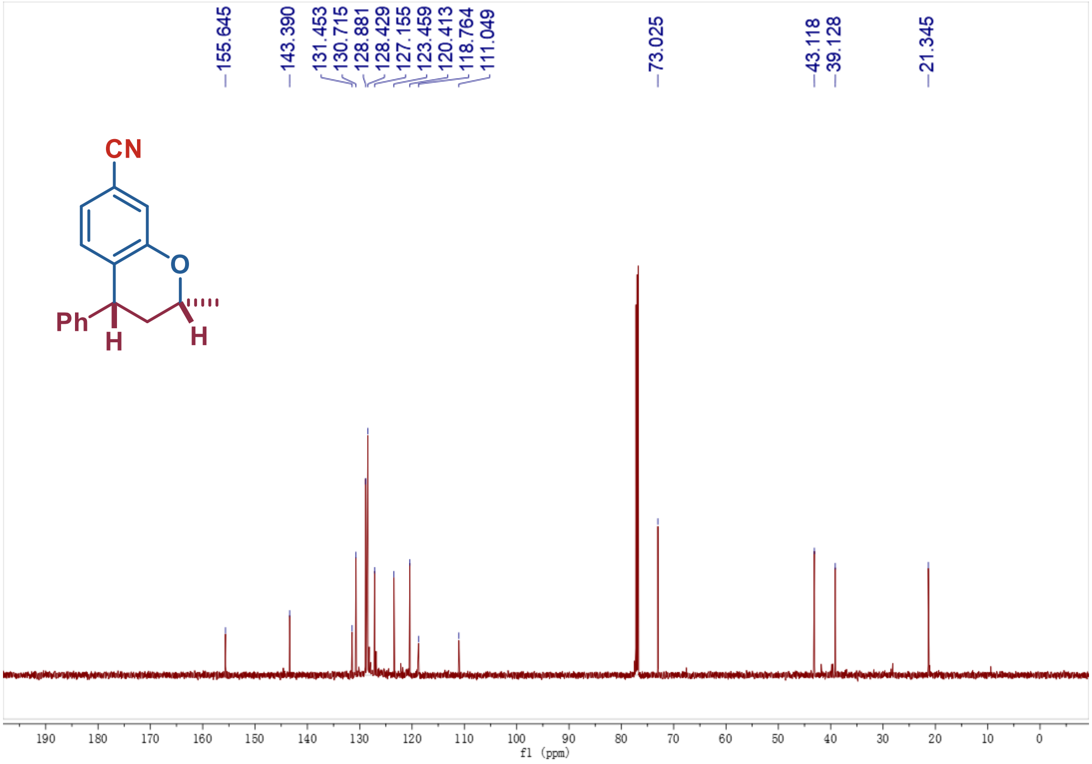

Fig. S154 ^{13}C NMR data of product *trans*-4y.

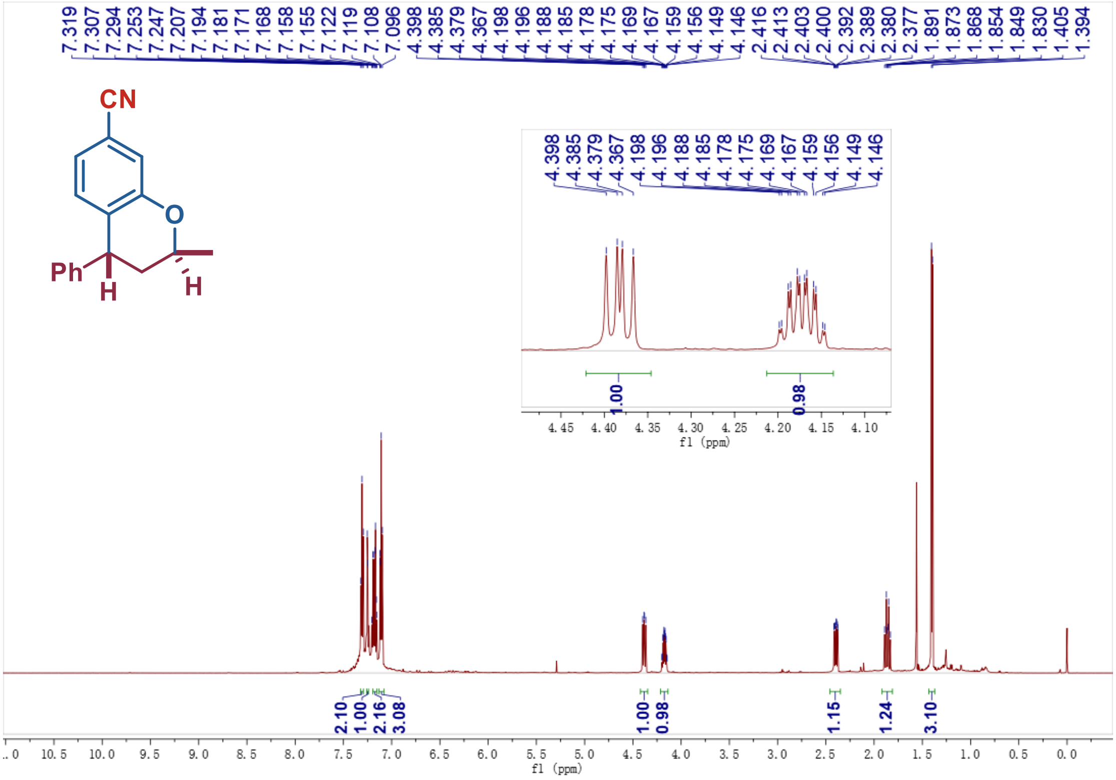

Fig. S155 ^{1}H NMR data of product *cis*-4y.

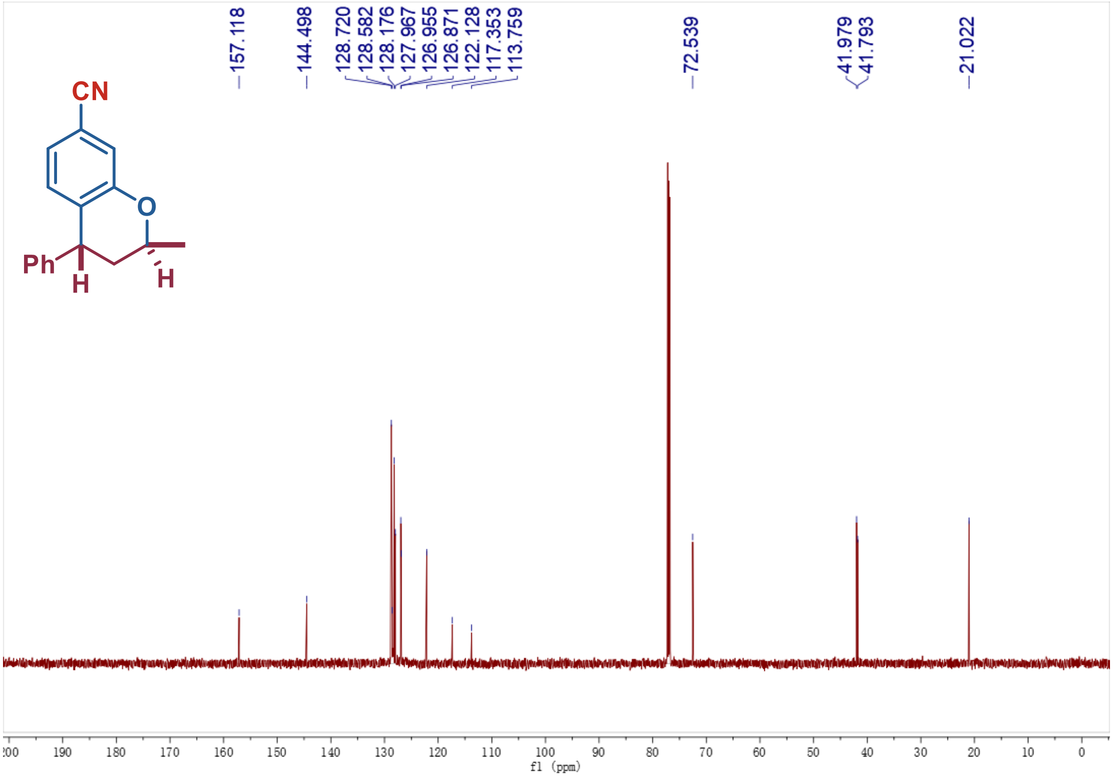

Fig. S156 ^{13}C NMR data of product *cis*-4y.

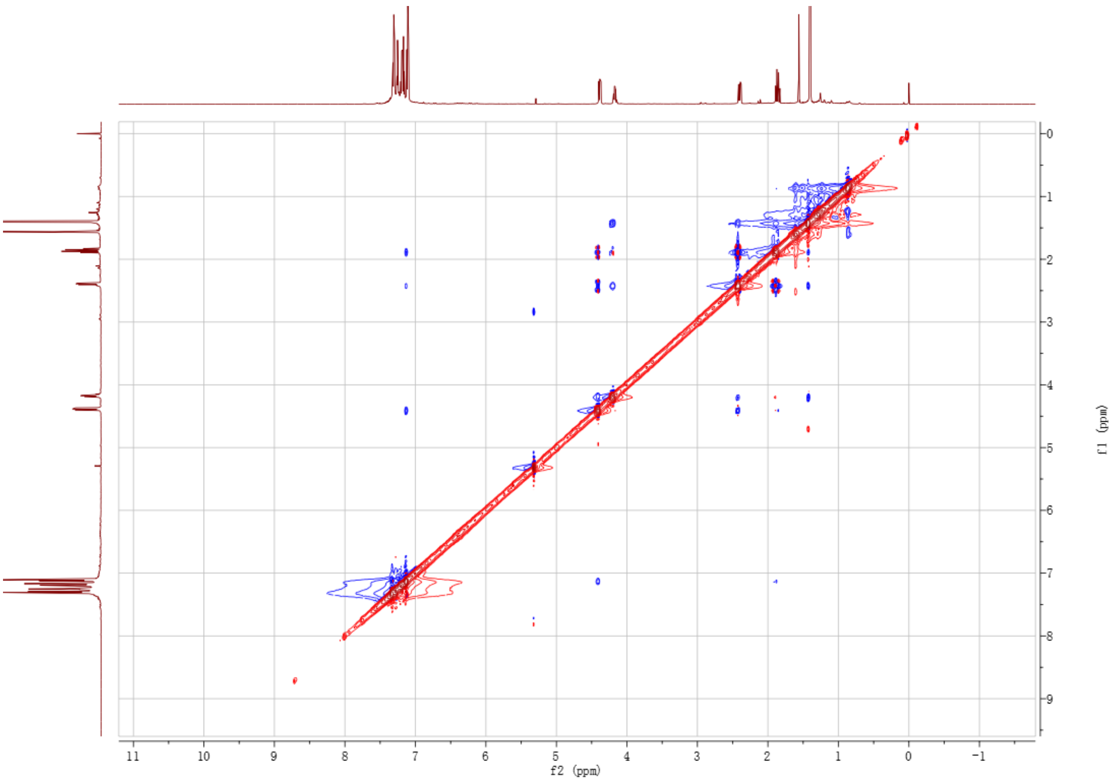

Fig. S157 Noesy data of product cis-4y.

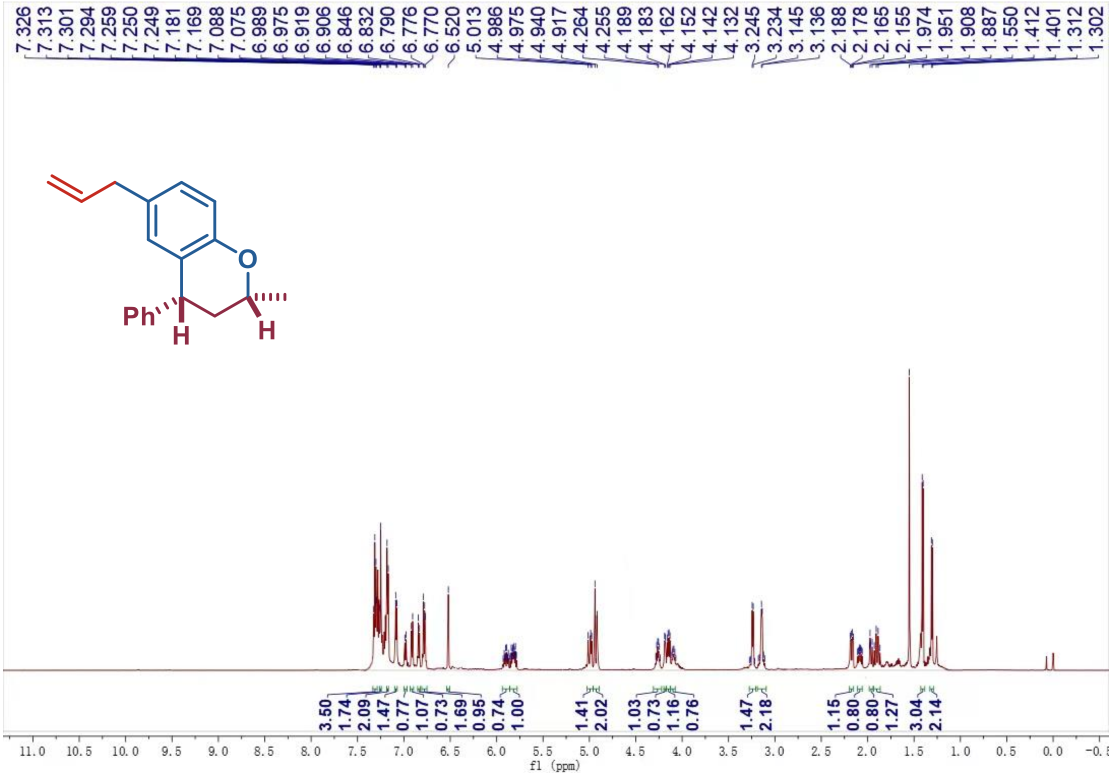

Fig. S158 ^{1}H NMR data of product 4z.

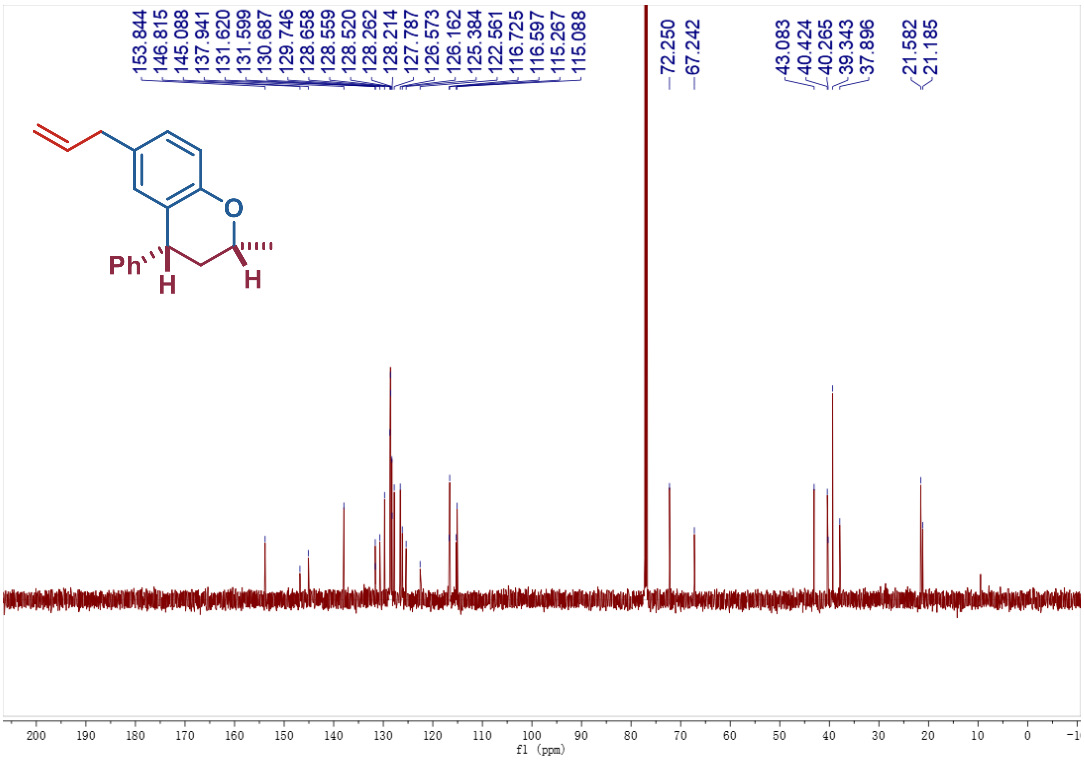

Fig. S159 ^{13}C NMR data of product 4z.

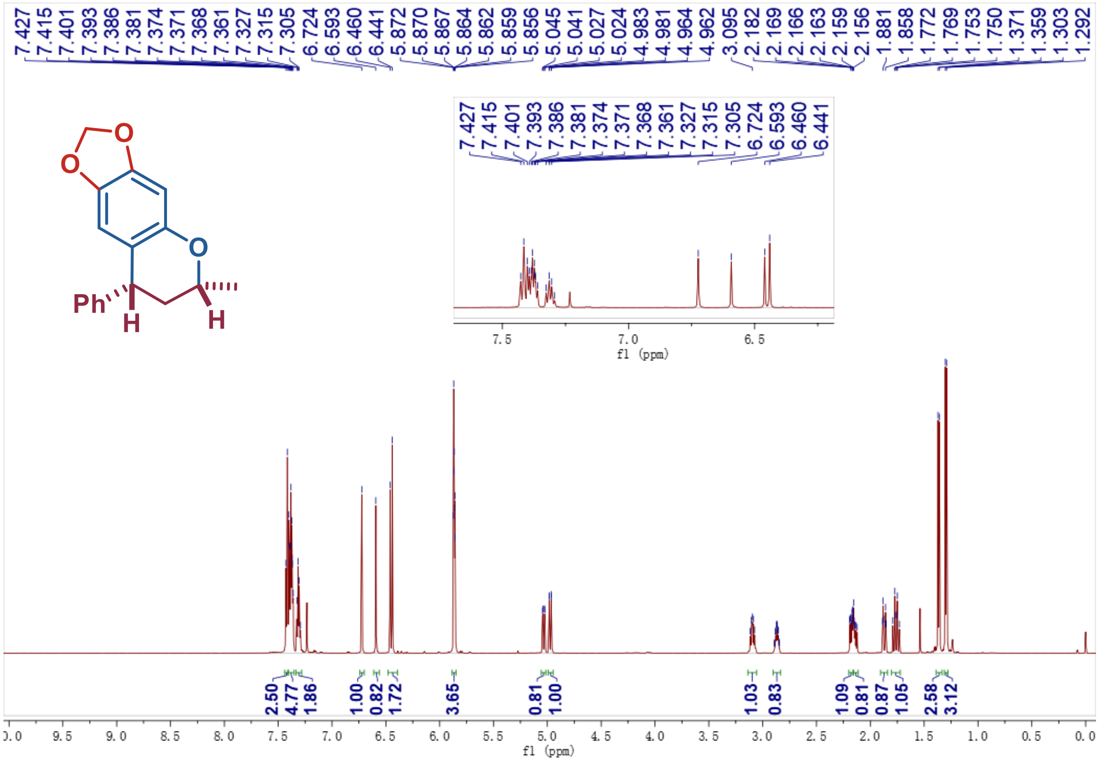

Fig. S160 ^{1}H NMR data of product 4aa.

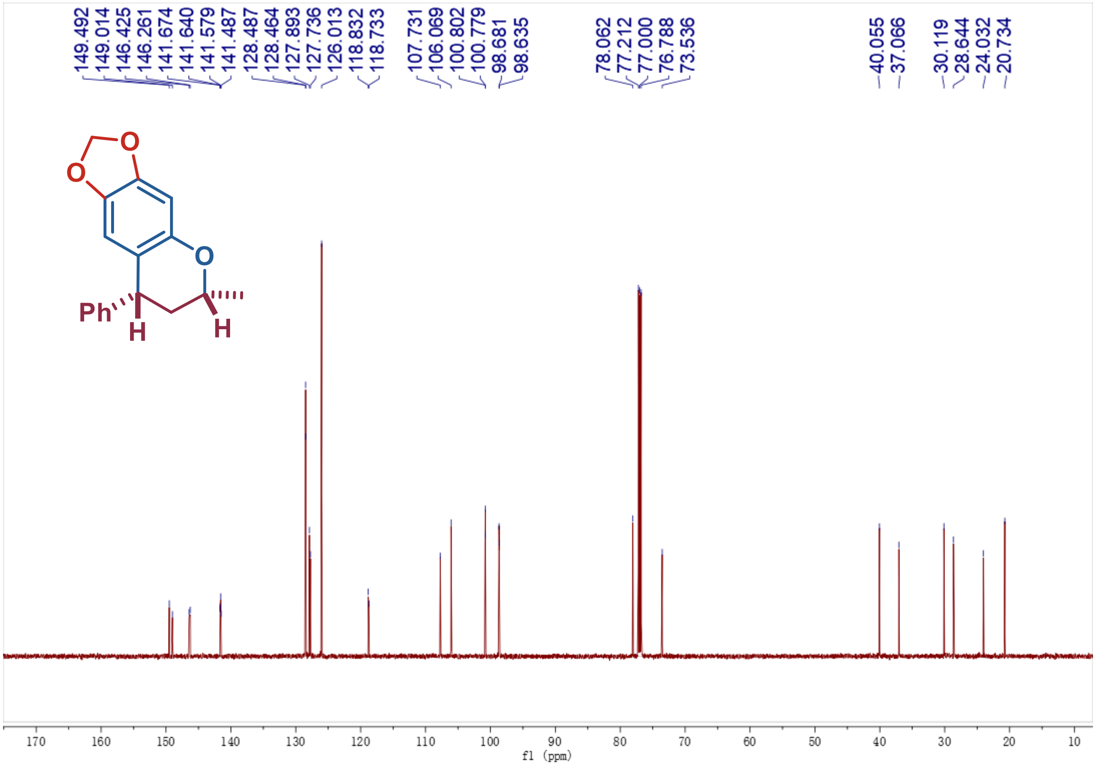

Fig. S161 ^{13}C NMR data of product 4aa.

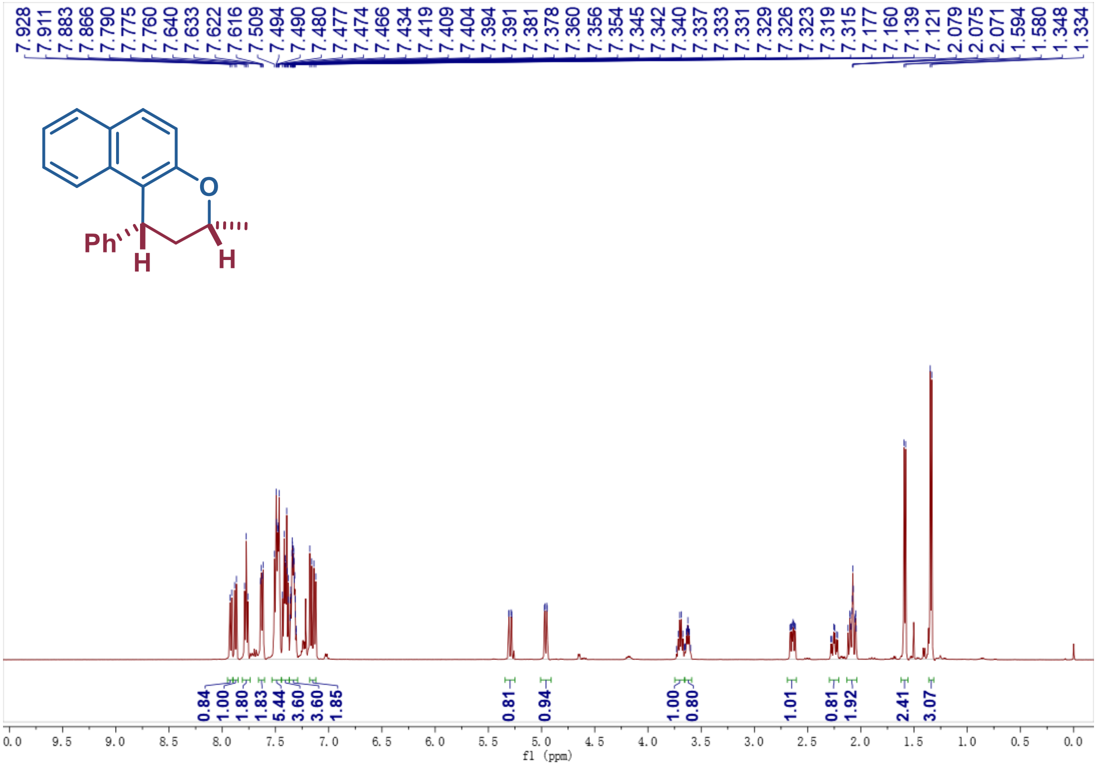

Fig. S162 ^{1}H NMR data of product 4ab.

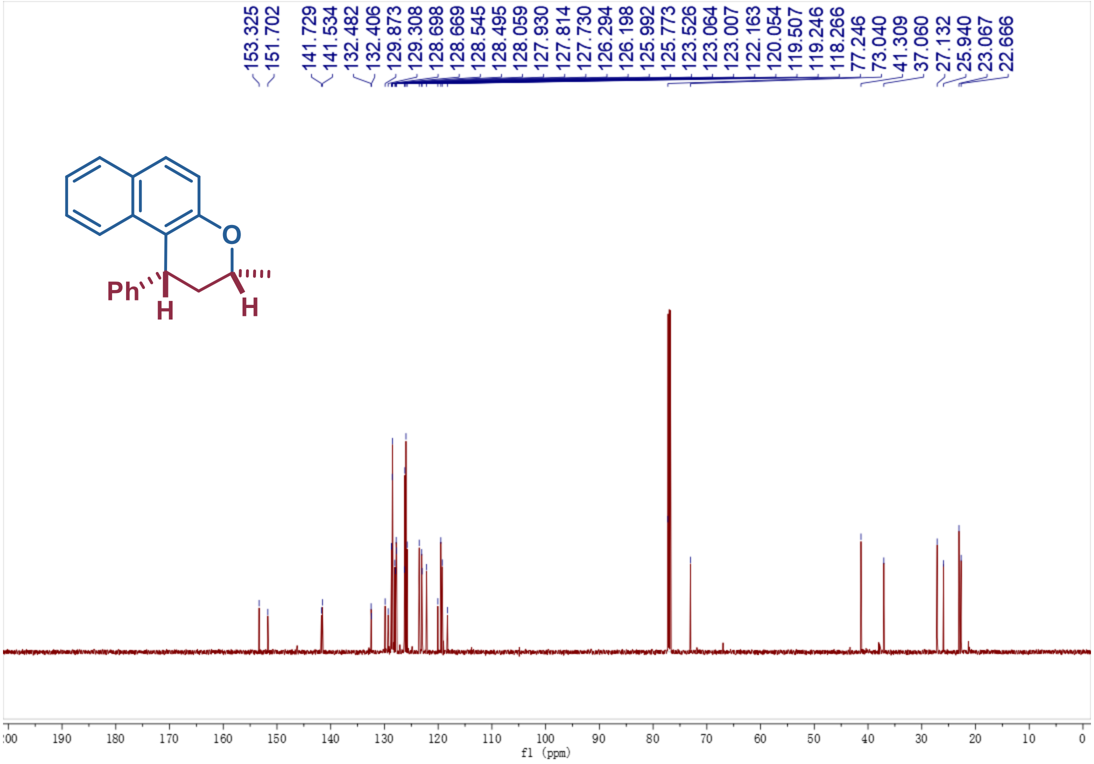

Fig. S163 ^{13}C NMR data of product 4ab.

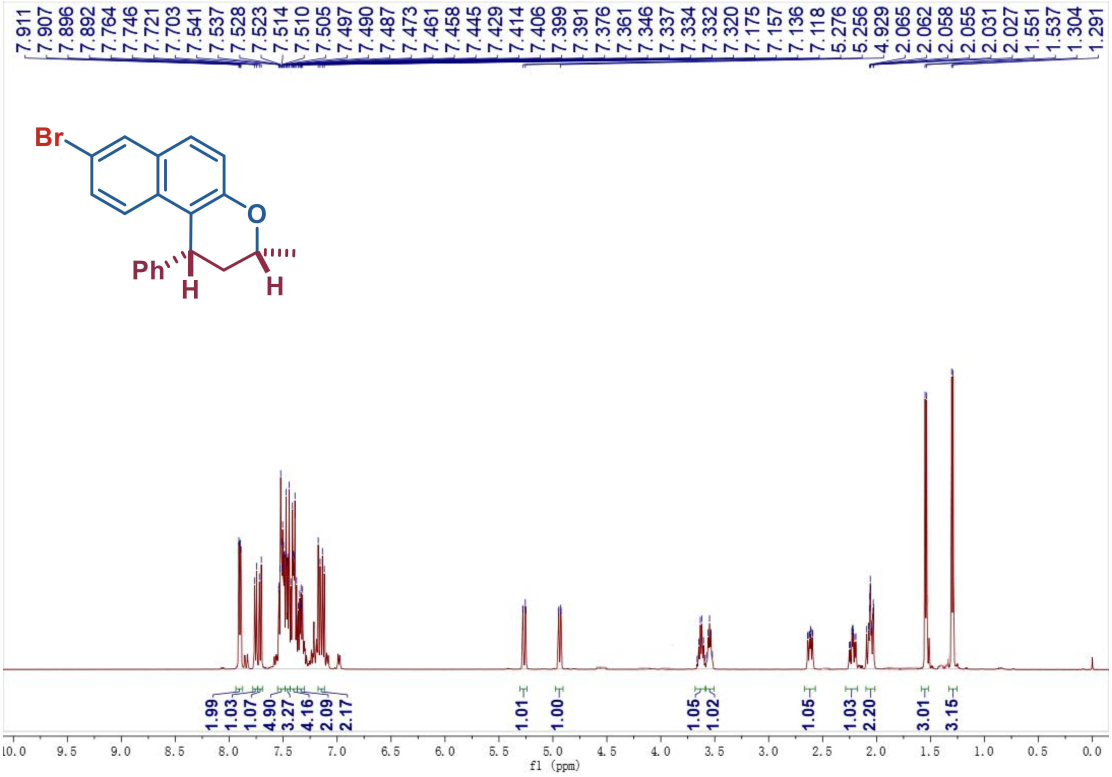

Fig. S164 ^{1}H NMR data of product 4ac.

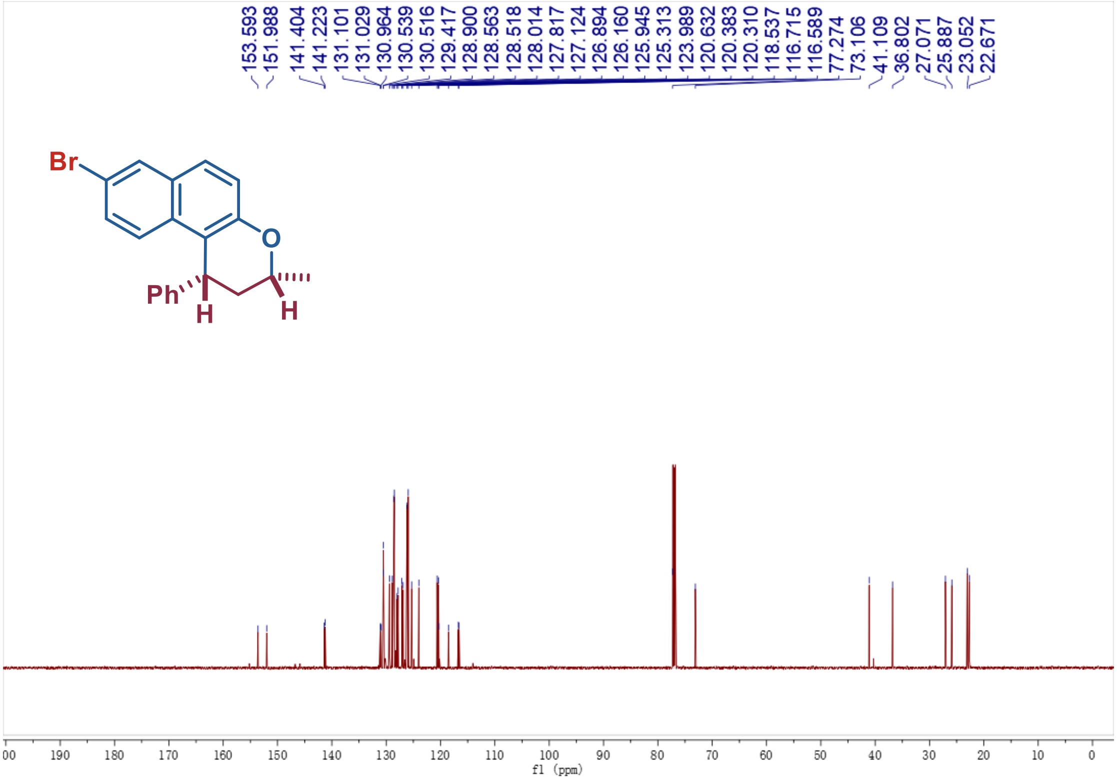

Fig. S165 ^{13}C NMR data of product 4ac.

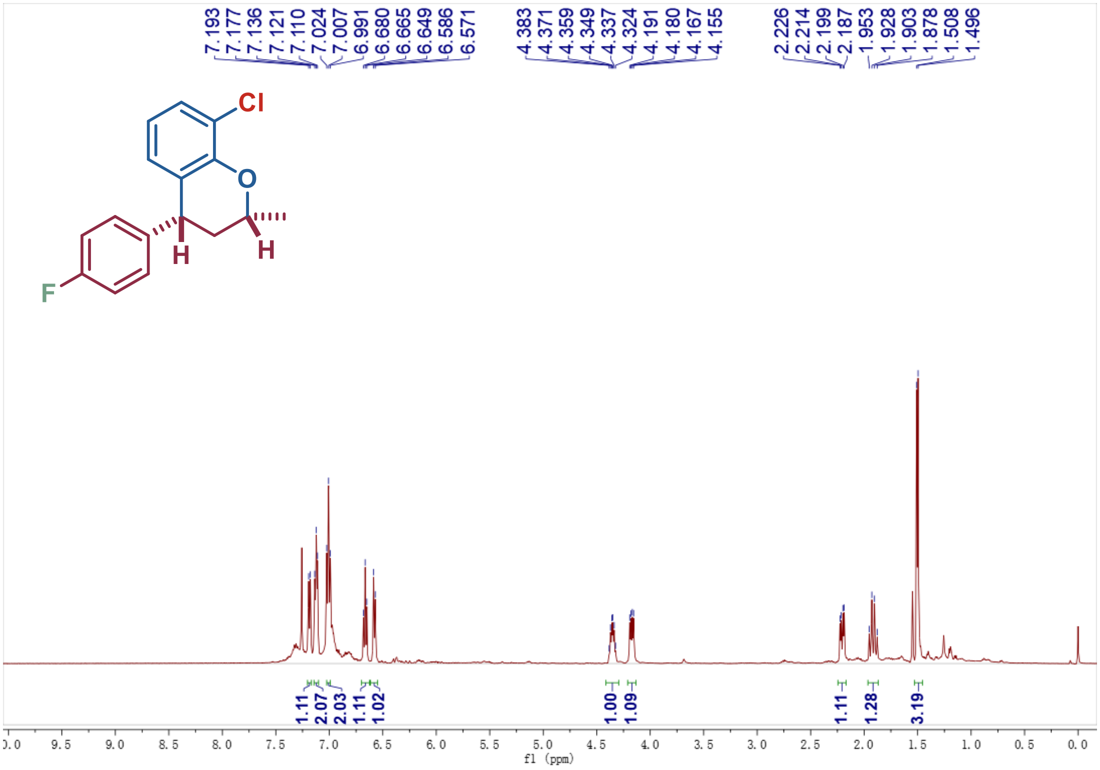

Fig. S166 ^{1}H NMR data of product 4ad.

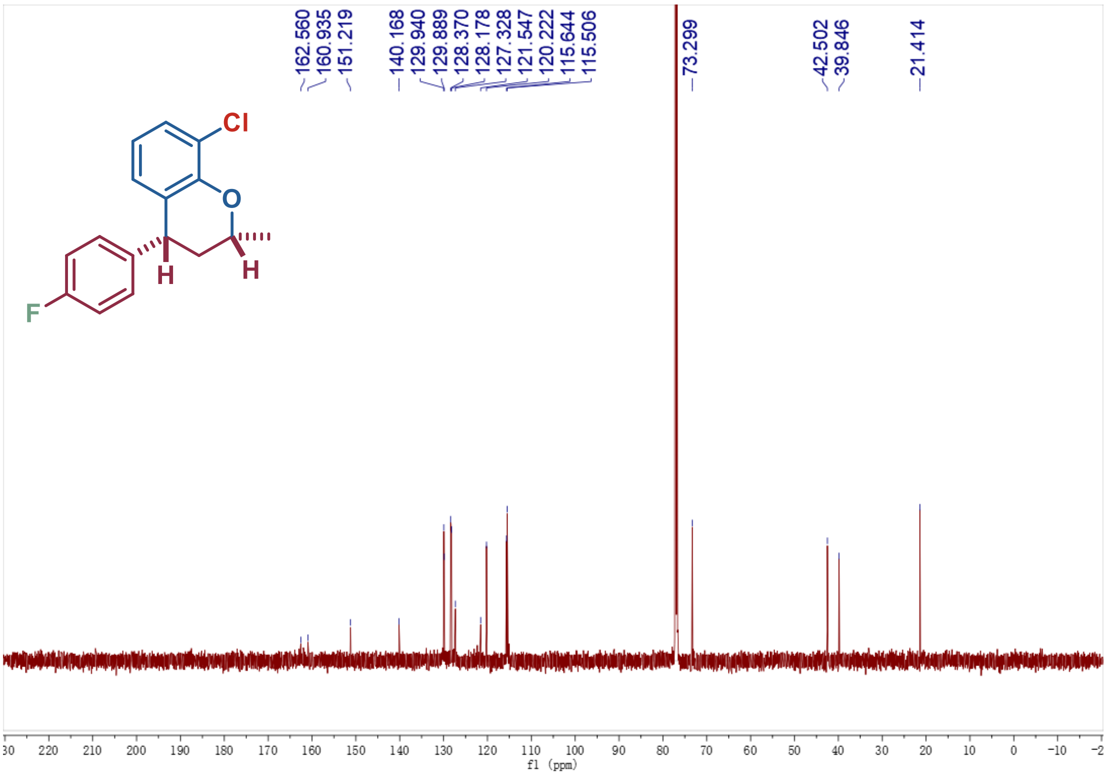

Fig. S167 ^{13}C NMR data of product 4ad.

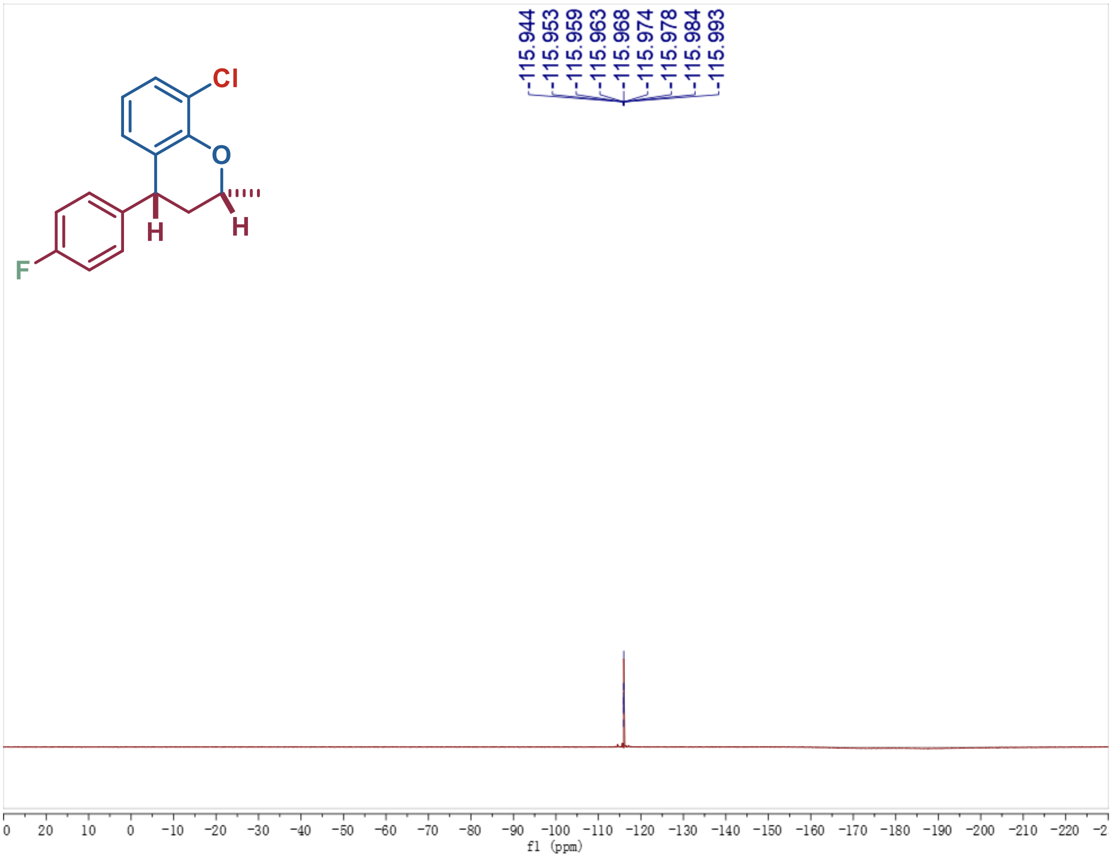

Fig. S168 ^{19}F NMR data of product 4ad.

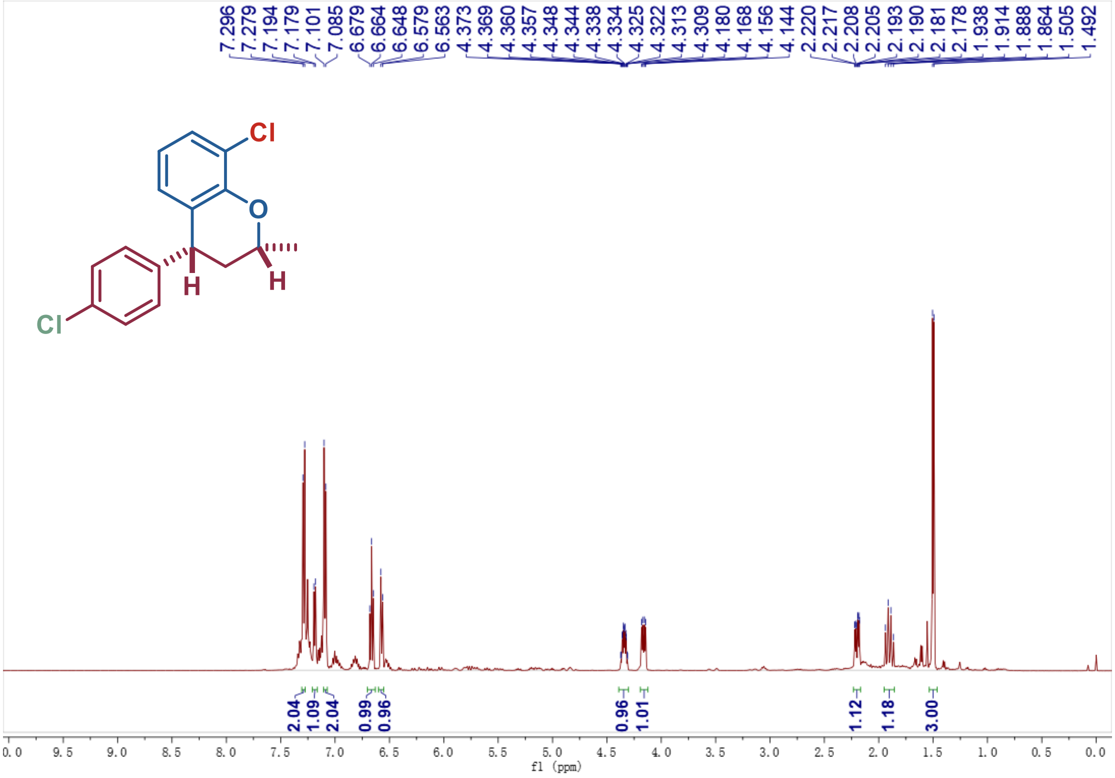

Fig. S169 ^{1}H NMR data of product 4ae.

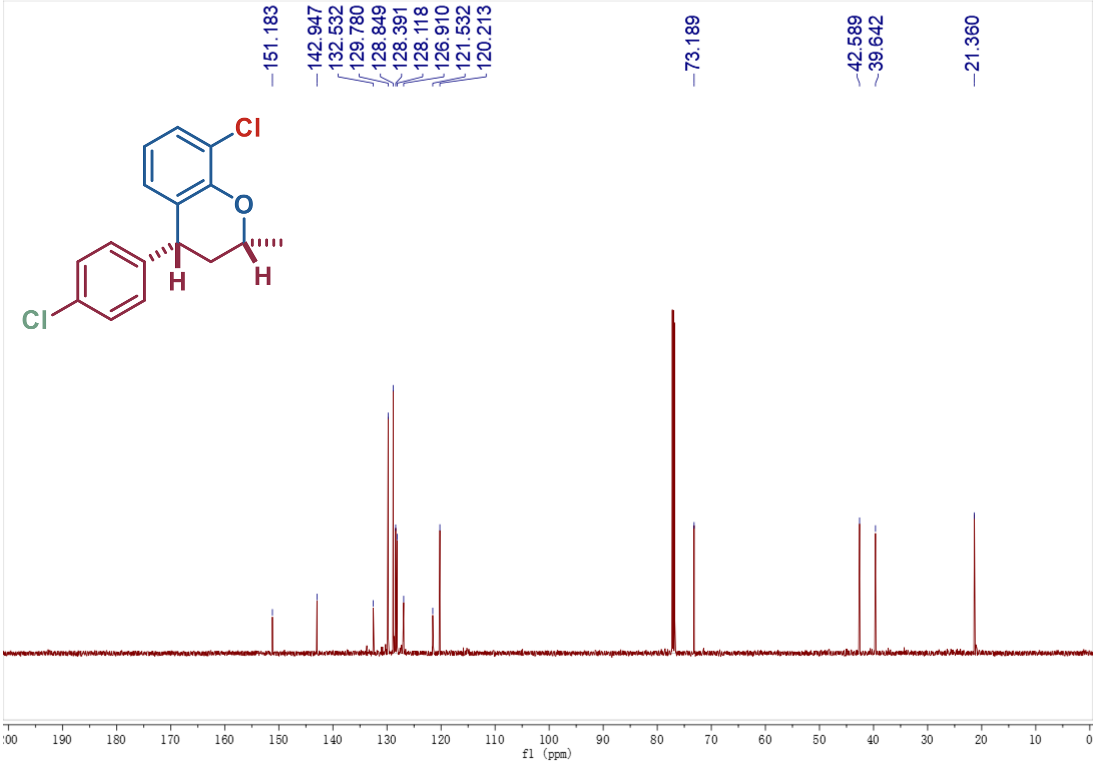

Fig. S170 ^{13}C NMR data of product 4ae.

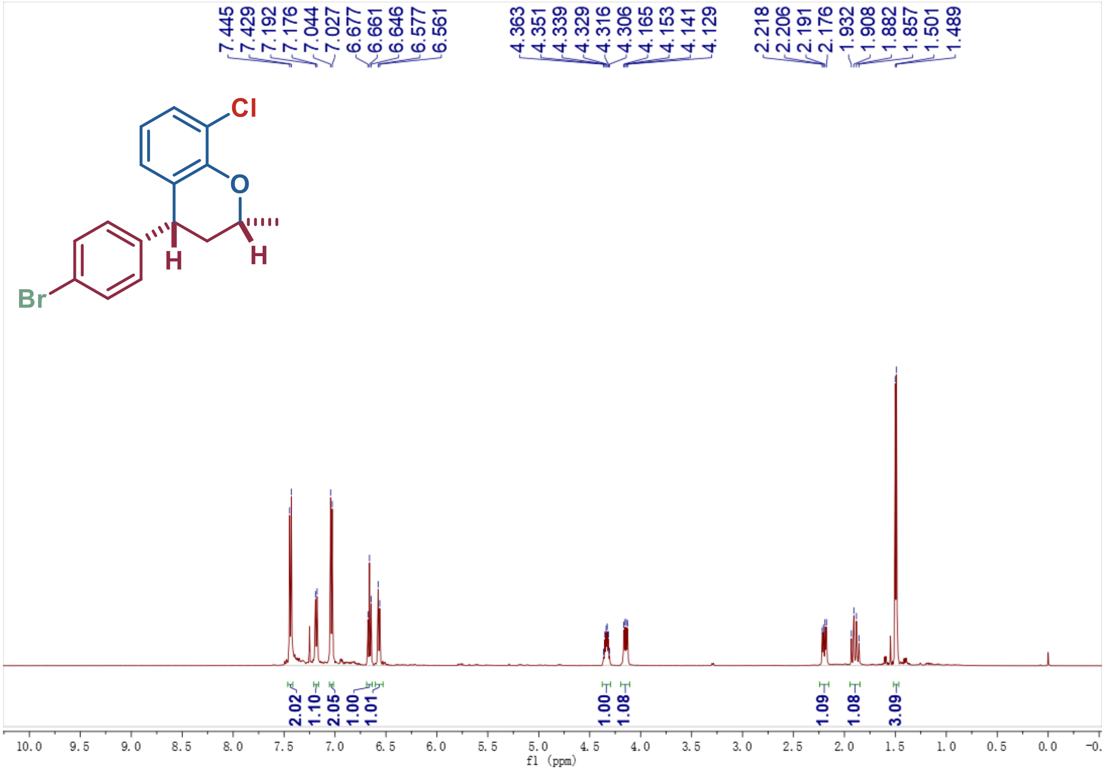

Fig. S171 ^{1}H NMR data of product 4af.

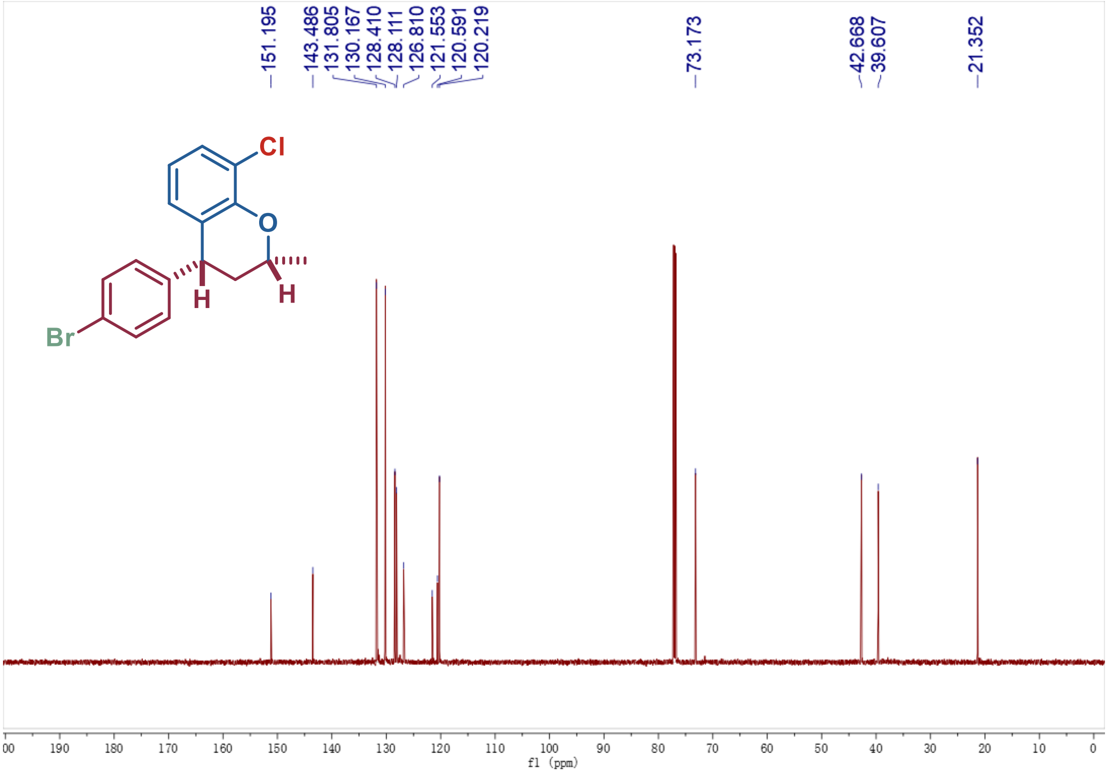

Fig. S172 ^{13}C NMR data of product 4af.

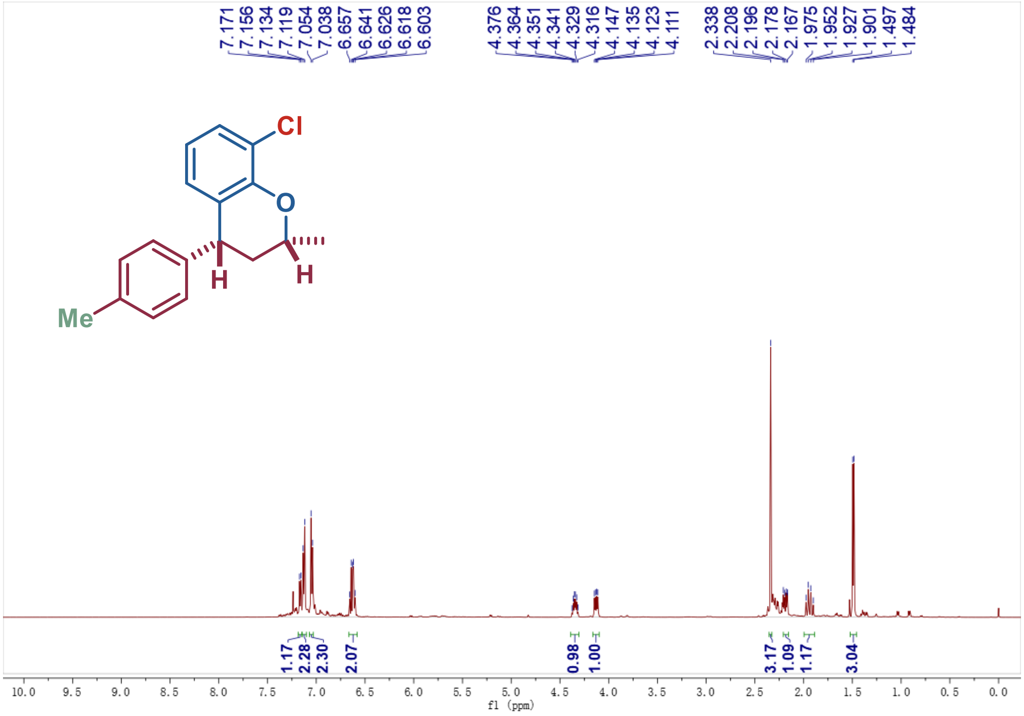

Fig. S173 ^{1}H NMR data of product 4ag.

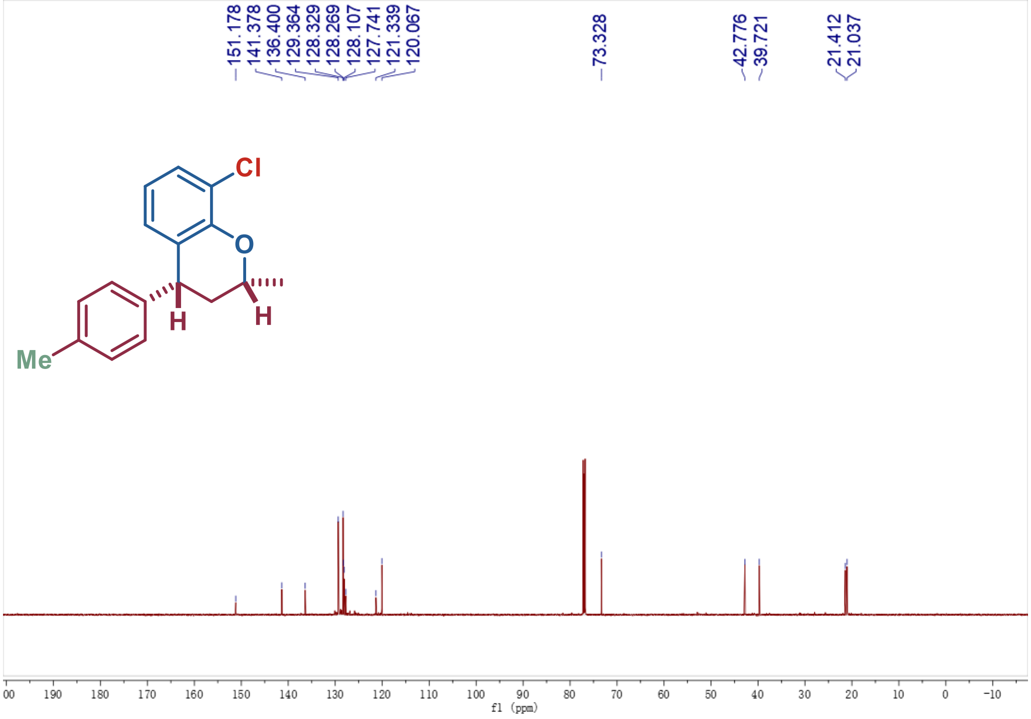

Fig. S174 ^{13}C NMR data of product 4ag.

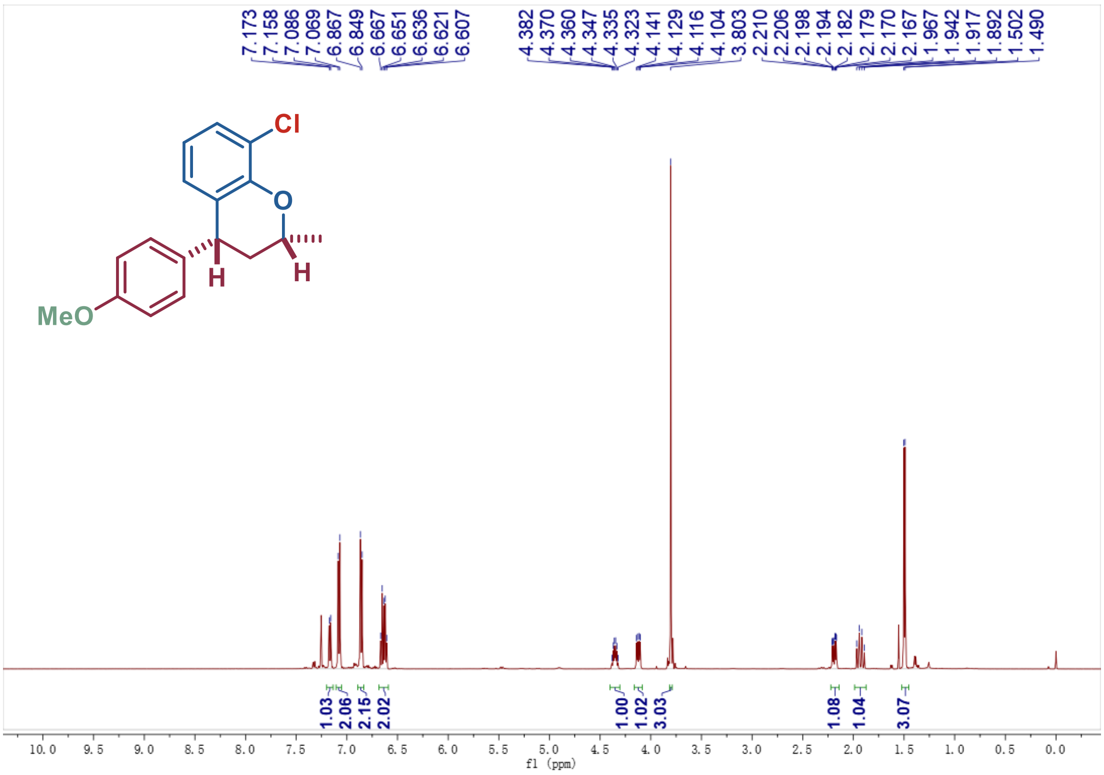

Fig. S175 ^{1}H NMR data of product 4ah.

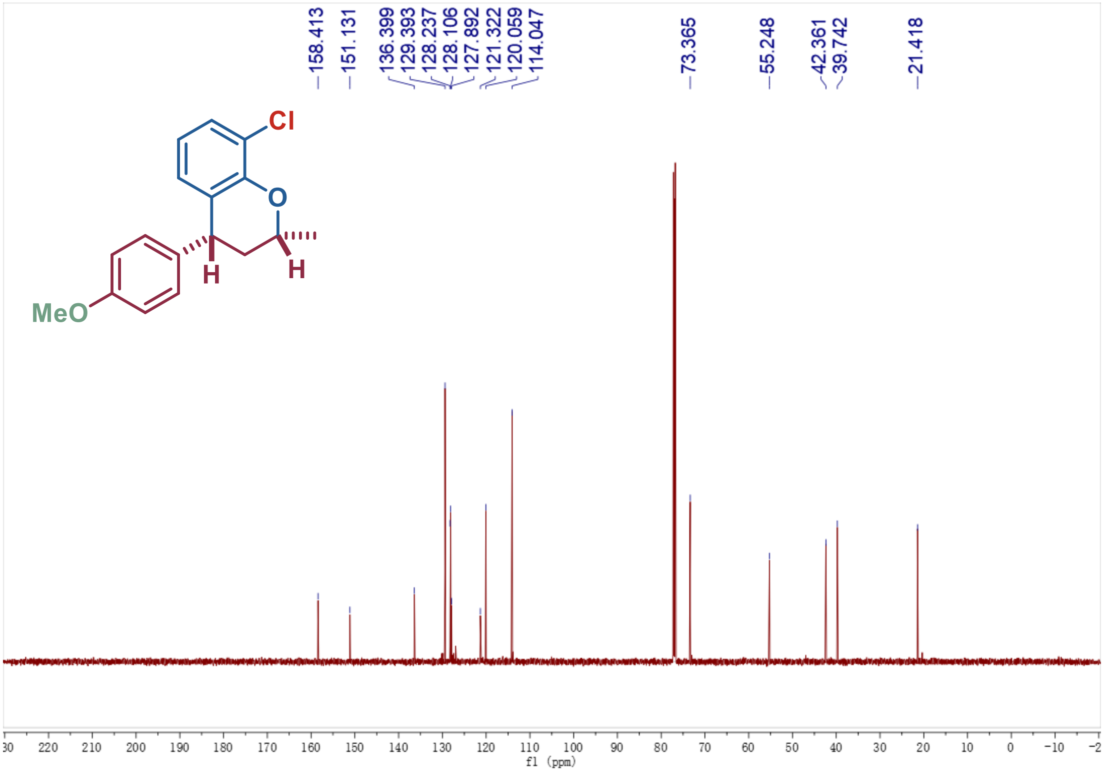

Fig. S176 ^{13}C NMR data of product 4ah.

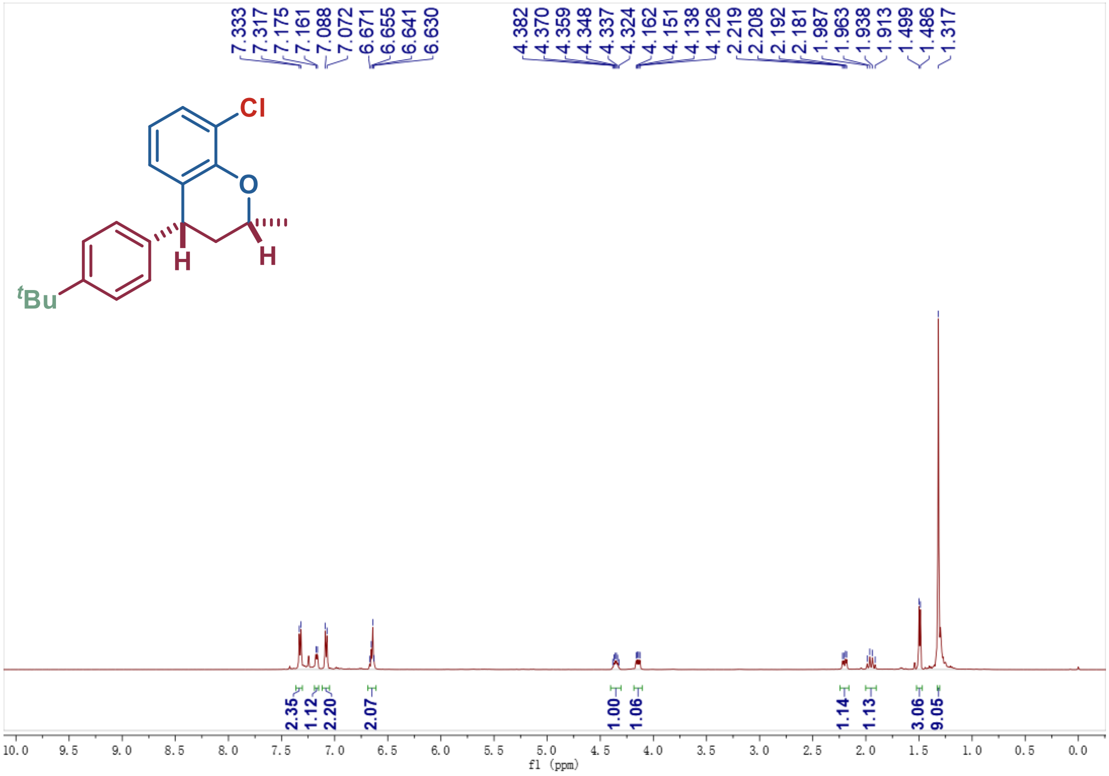

Fig. S177 ^{1}H NMR data of product 4ai.

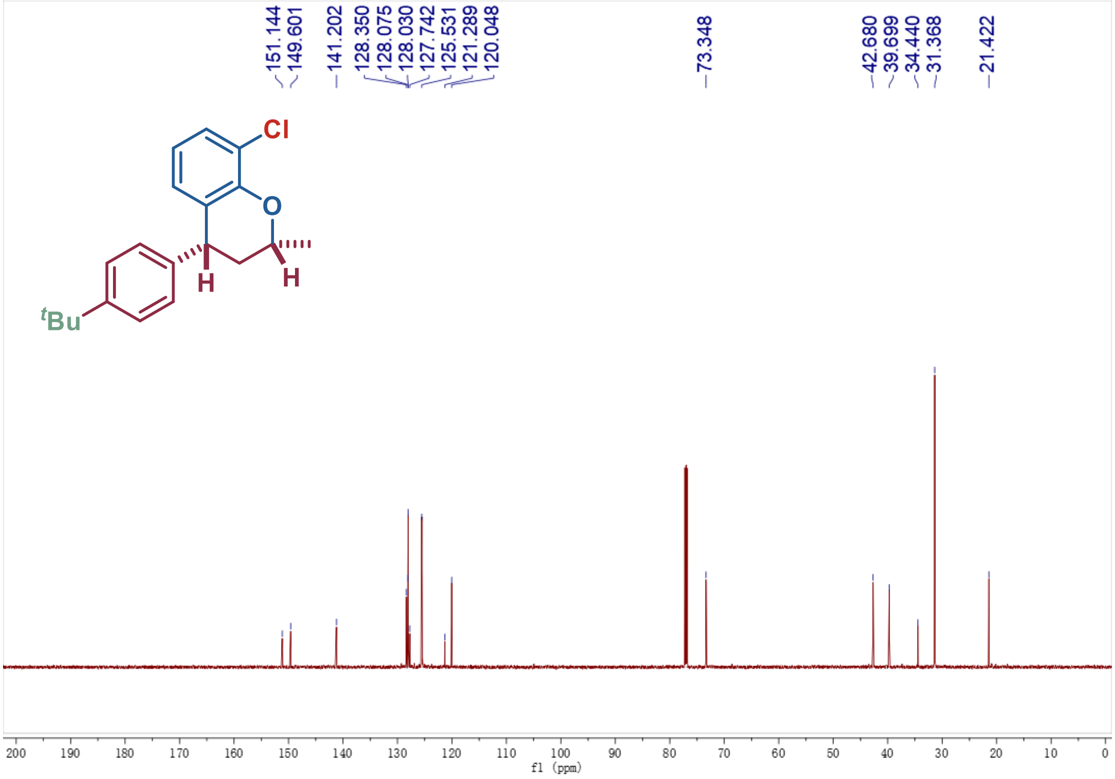

Fig. S178 ^{13}C NMR data of product 4ai.

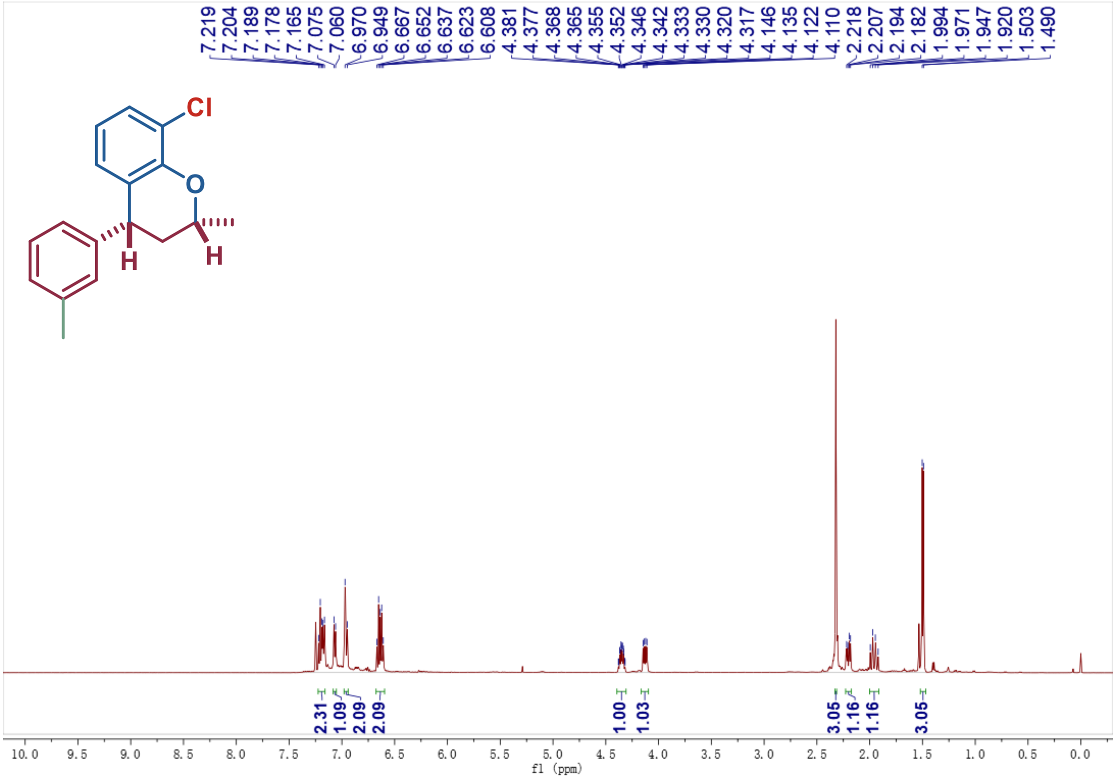

Fig. S179 ^{1}H NMR data of product 4aj.

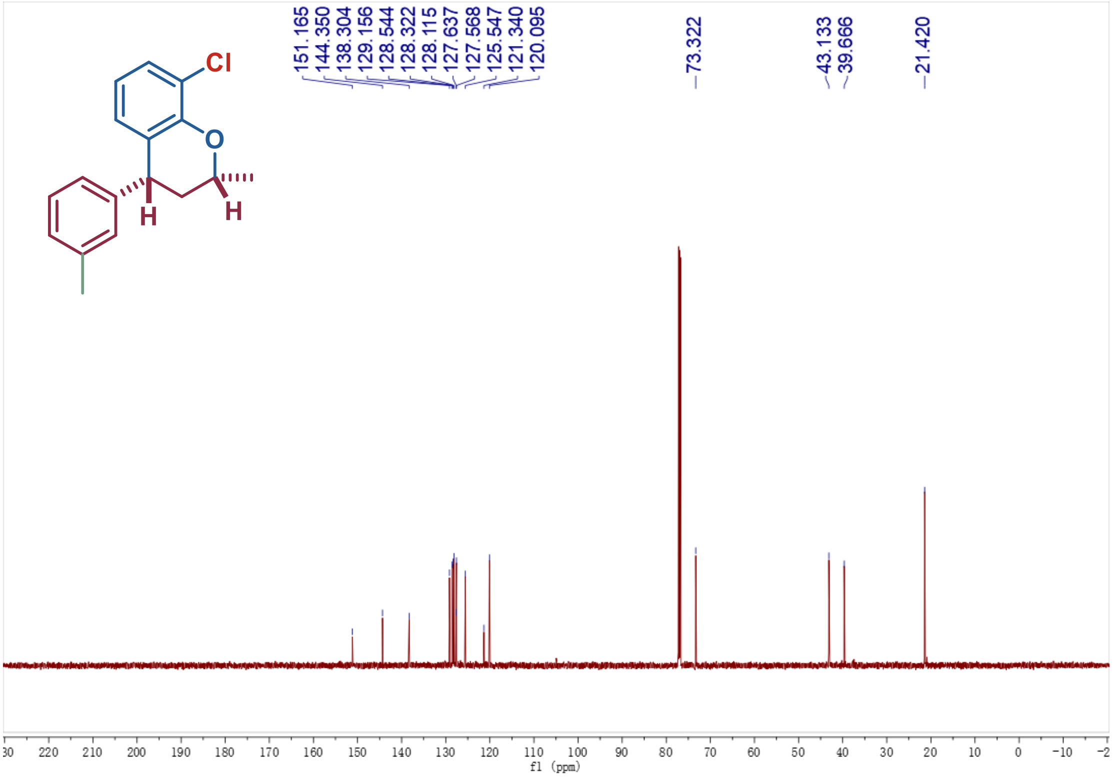

Fig. S180 ^{13}C NMR data of product 4aj.

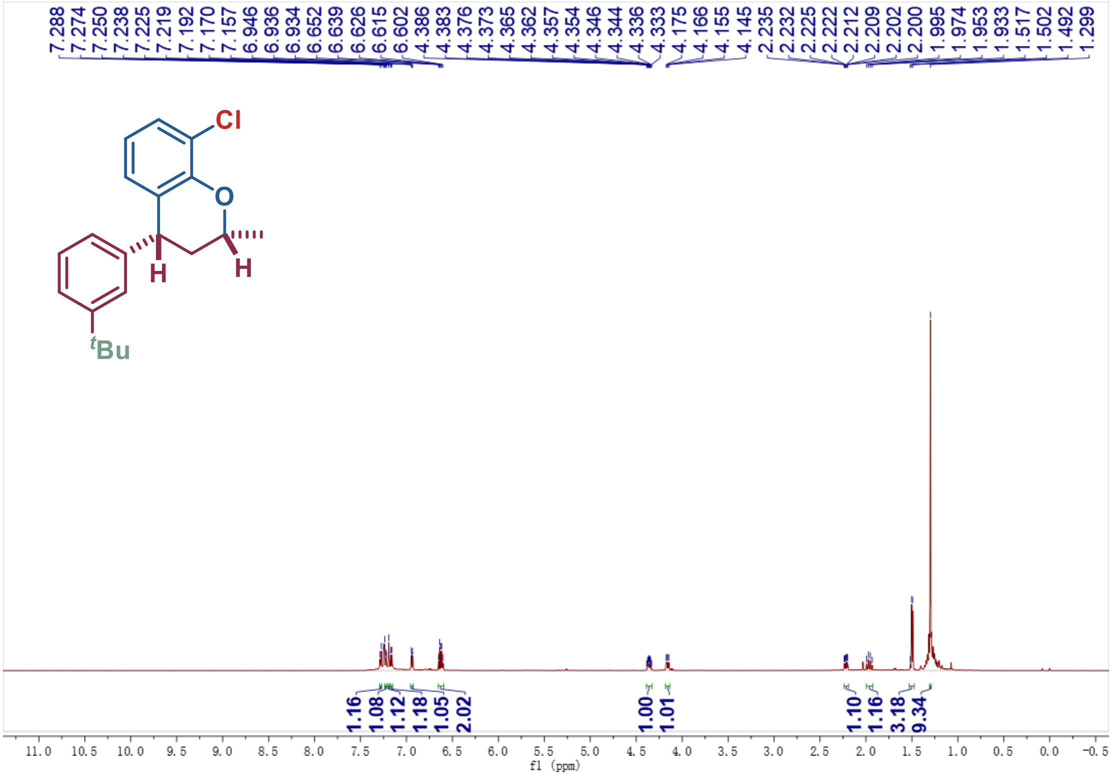

Fig. S181 ¹H NMR data of product 4ak.

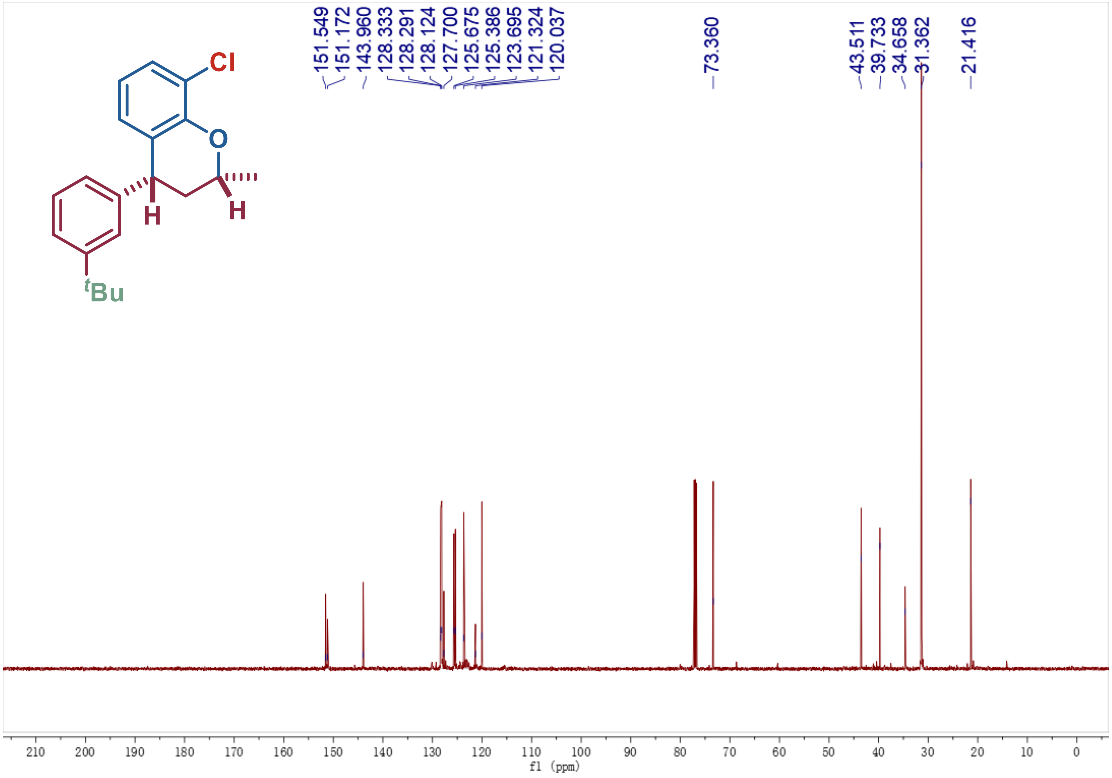

Fig. S182 ¹³C NMR data of product 4ak.

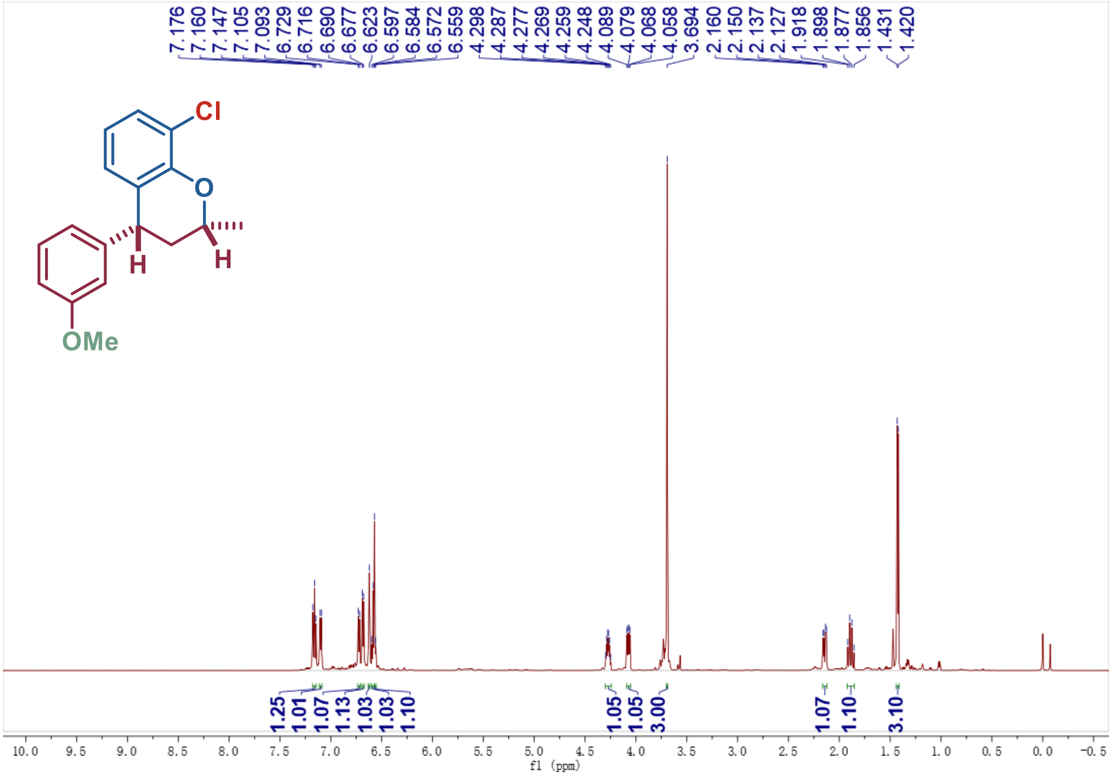

Fig. S183 ^{1}H NMR data of product 4al.

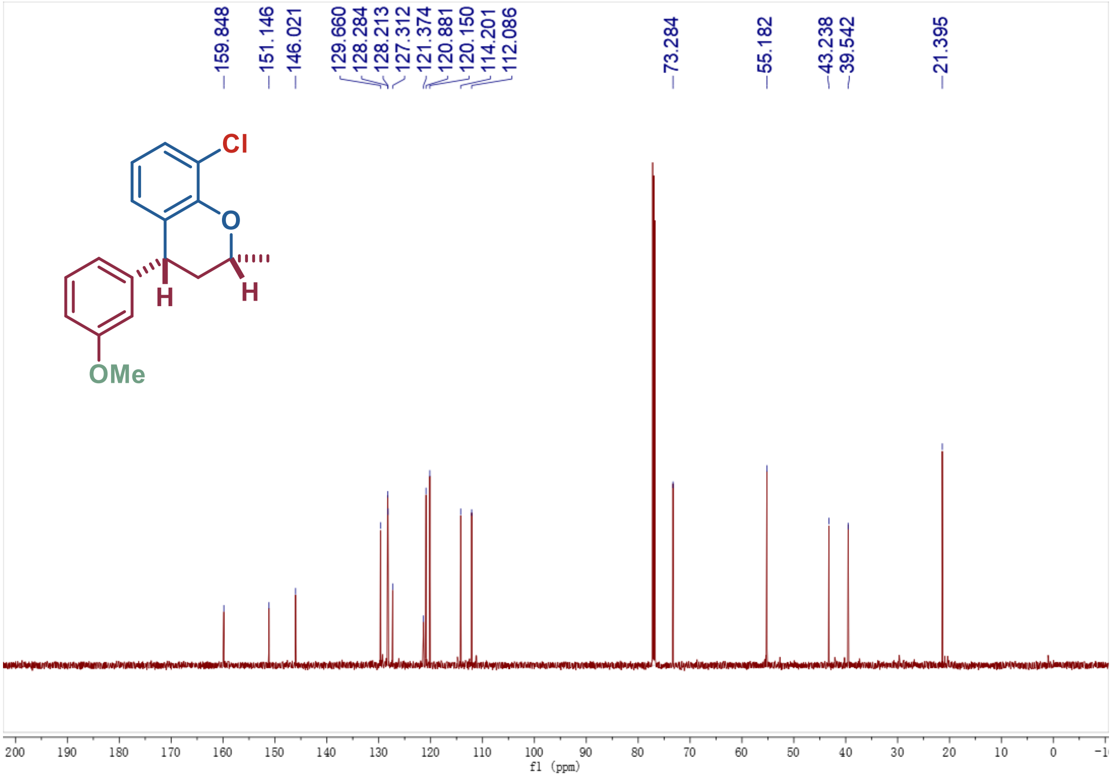

Fig. S184 ^{13}C NMR data of product 4al.

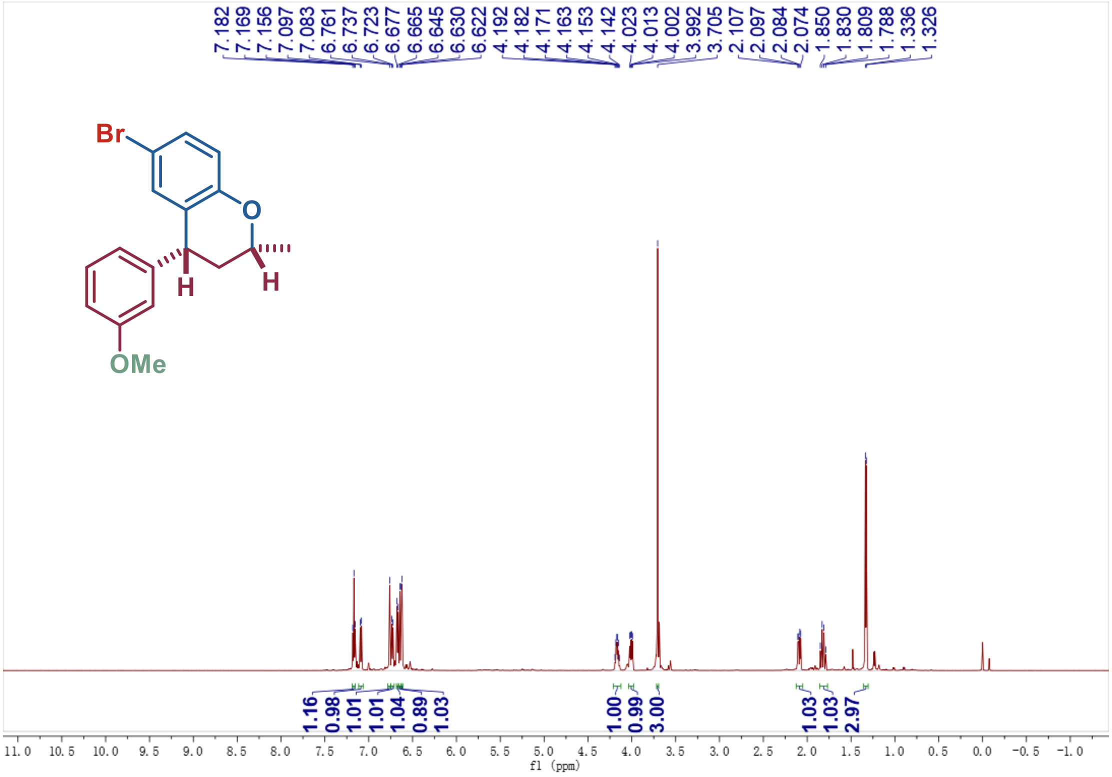

Fig. S185 ^{1}H NMR data of product 4am.

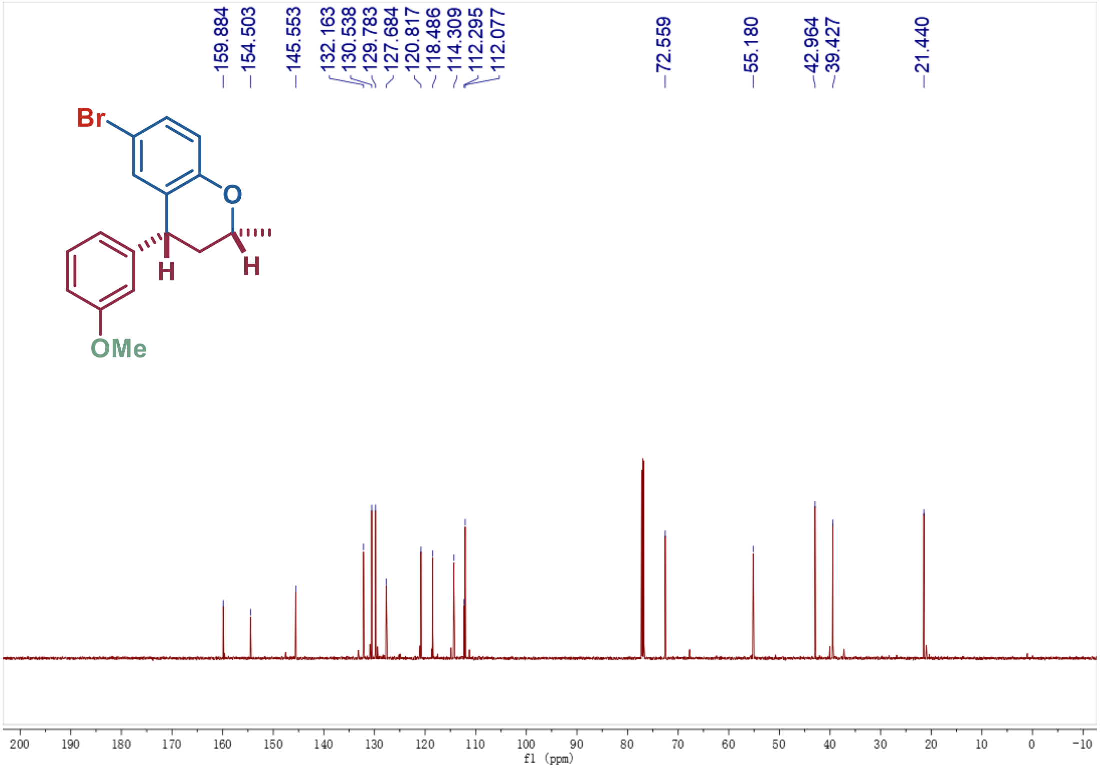

Fig. S186 ^{13}C NMR data of product 4am.

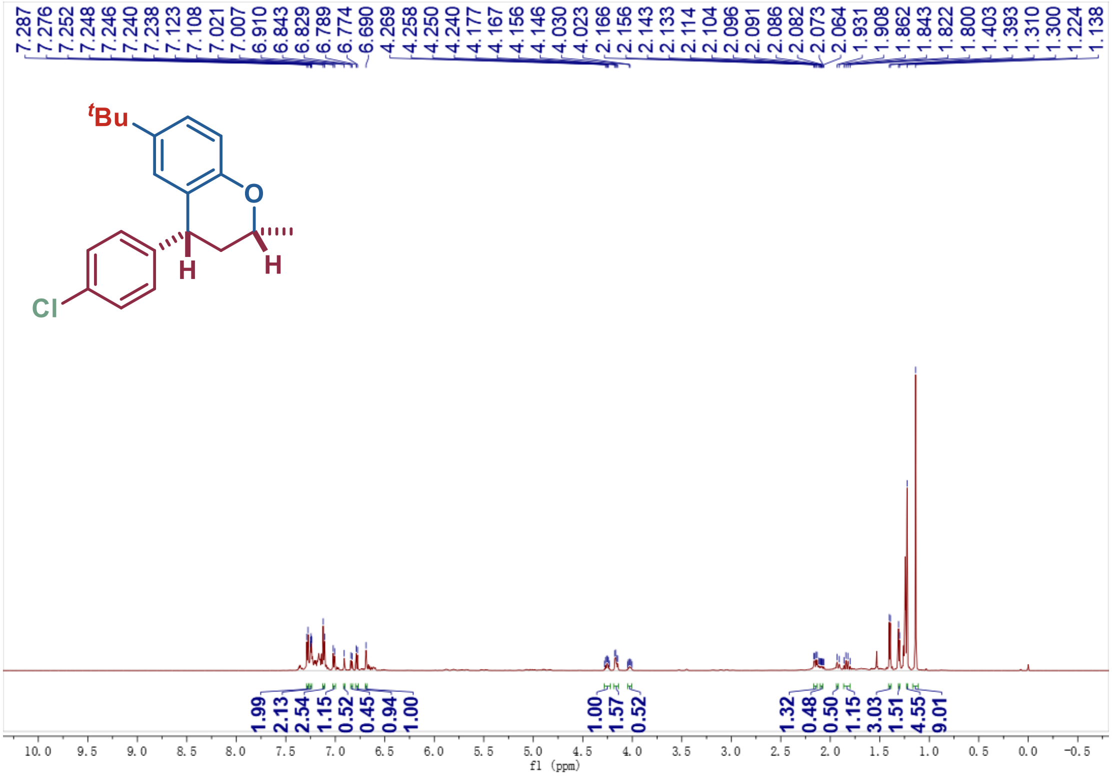

Fig. S187 ^{1}H NMR data of product 4an.

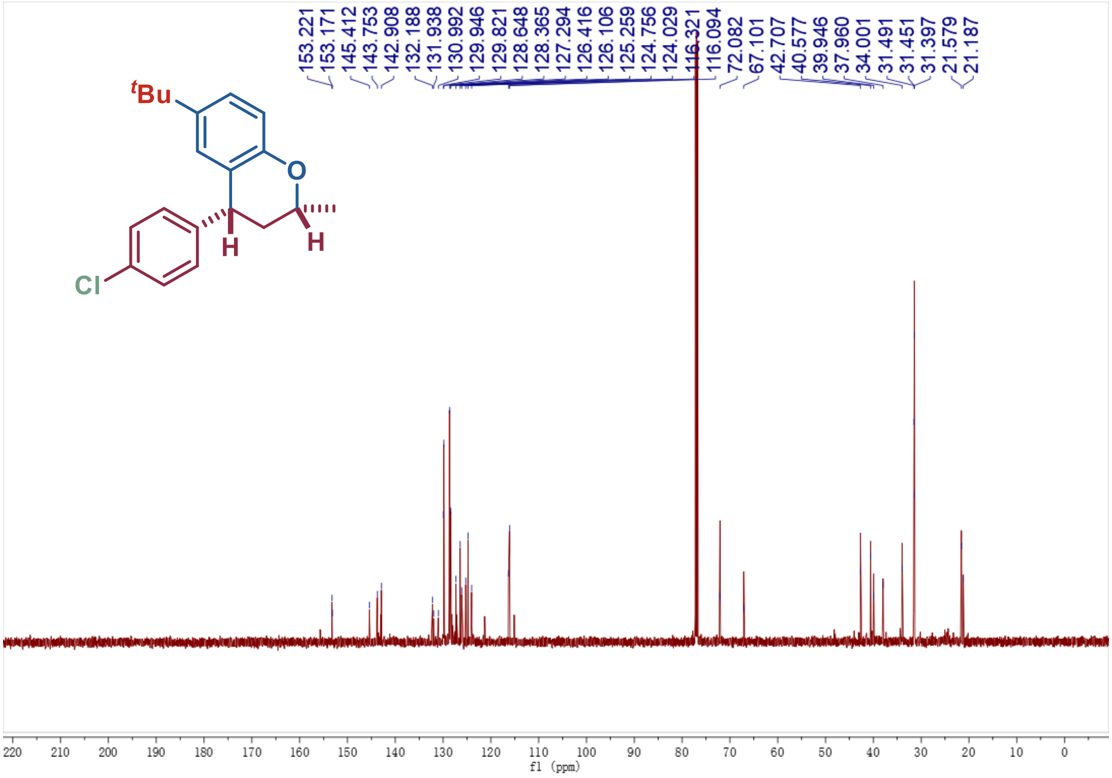

Fig. S188 ^{13}C NMR data of product 4an.

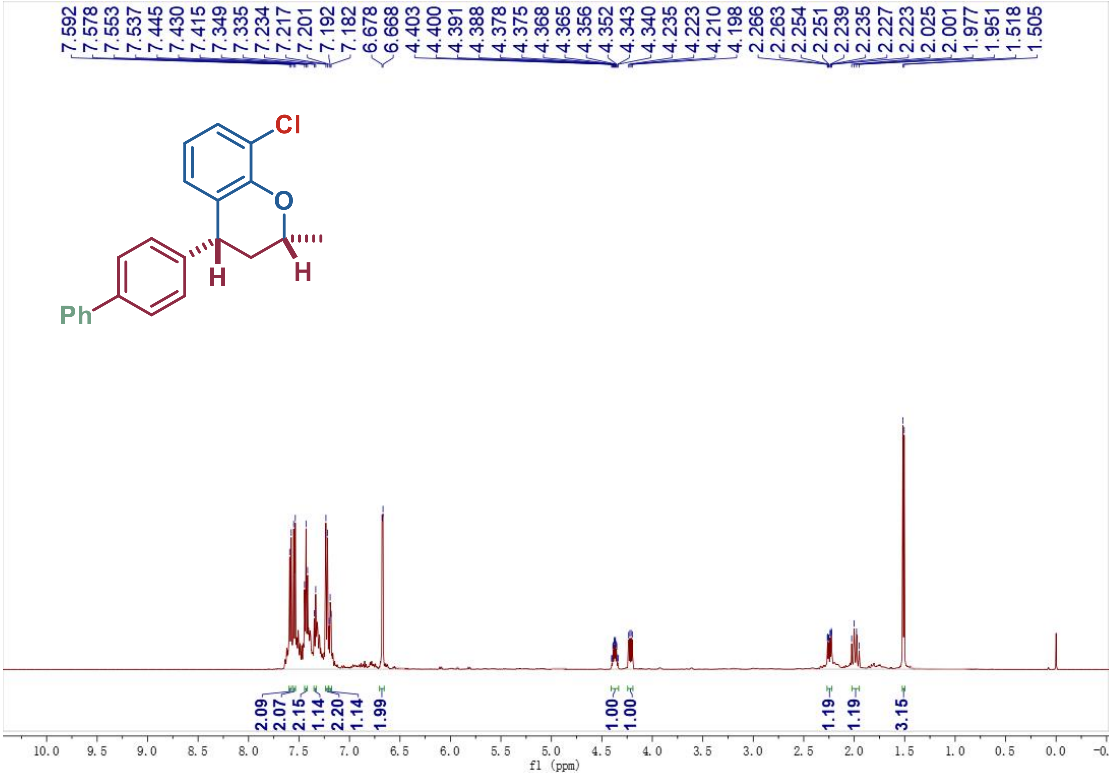

Fig. S189 ^{1}H NMR data of product 4ao.

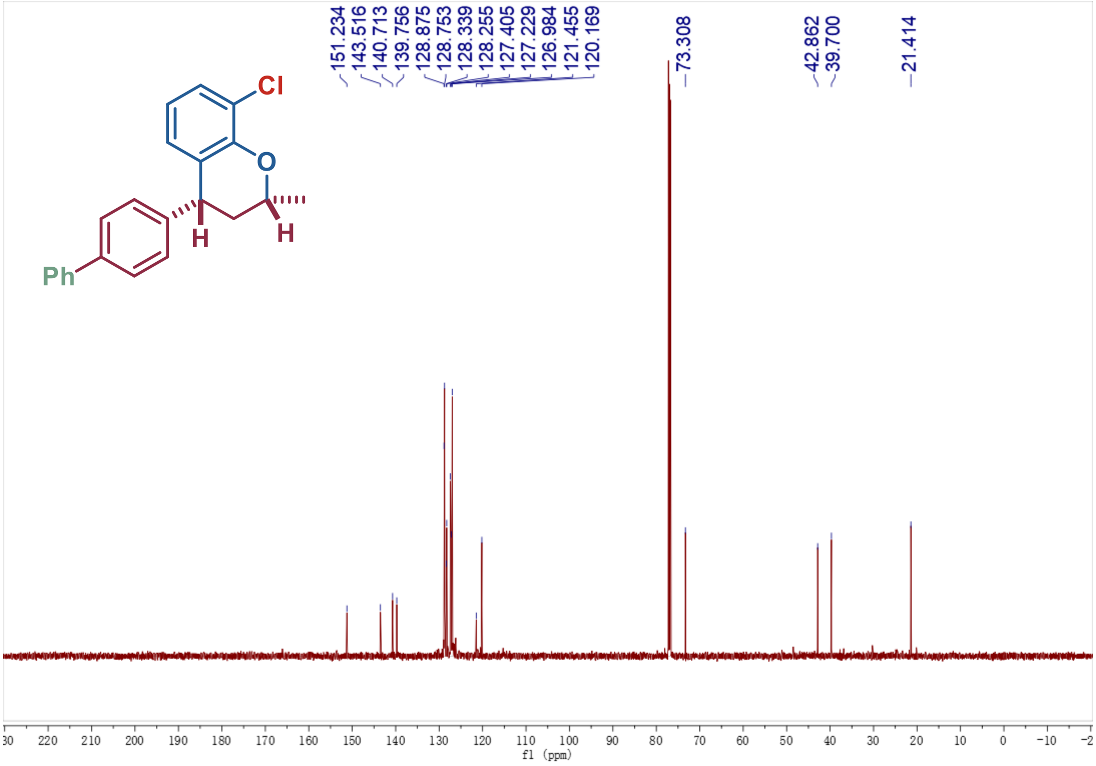

Fig. S190 ^{13}C NMR data of product 4ao.

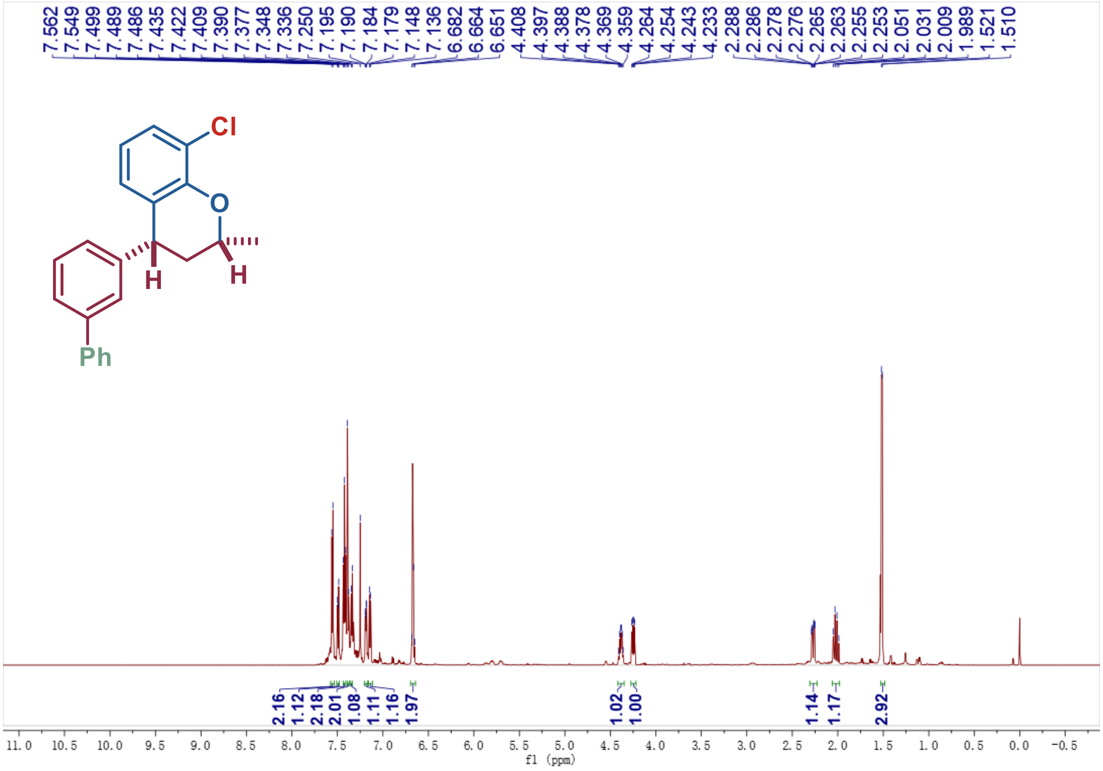

Fig. S191 ^{1}H NMR data of product 4ap.

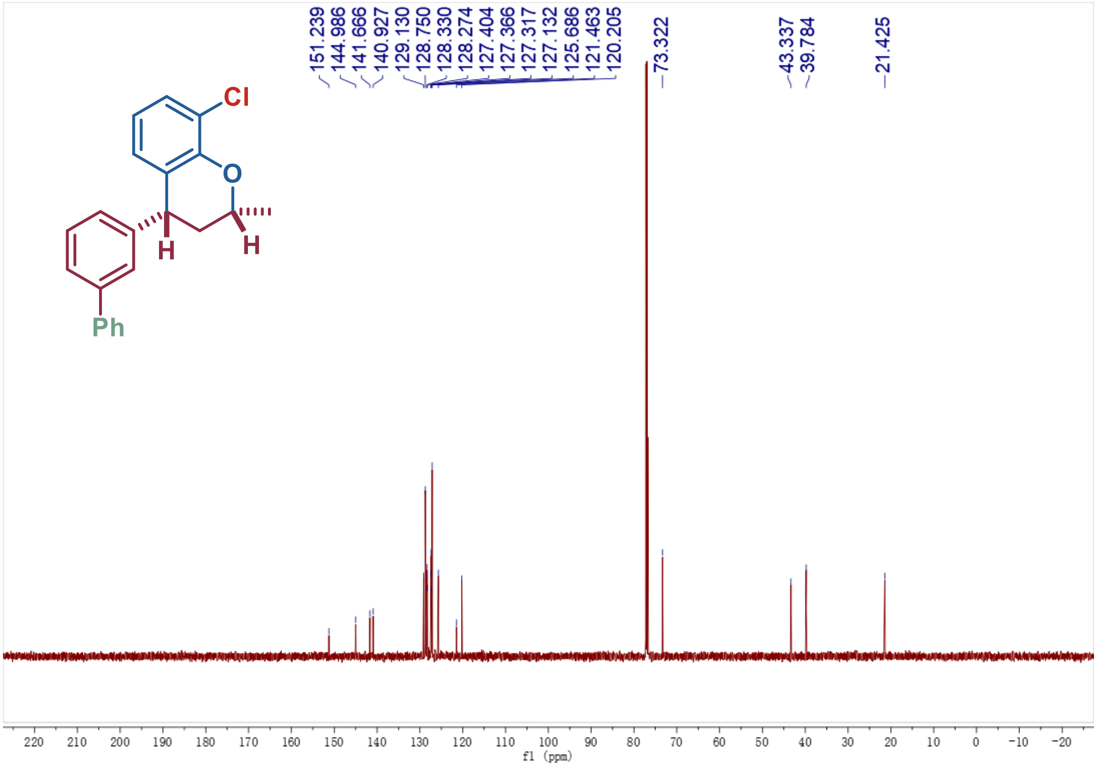

Fig. S192 ^{13}C NMR data of product 4ap.

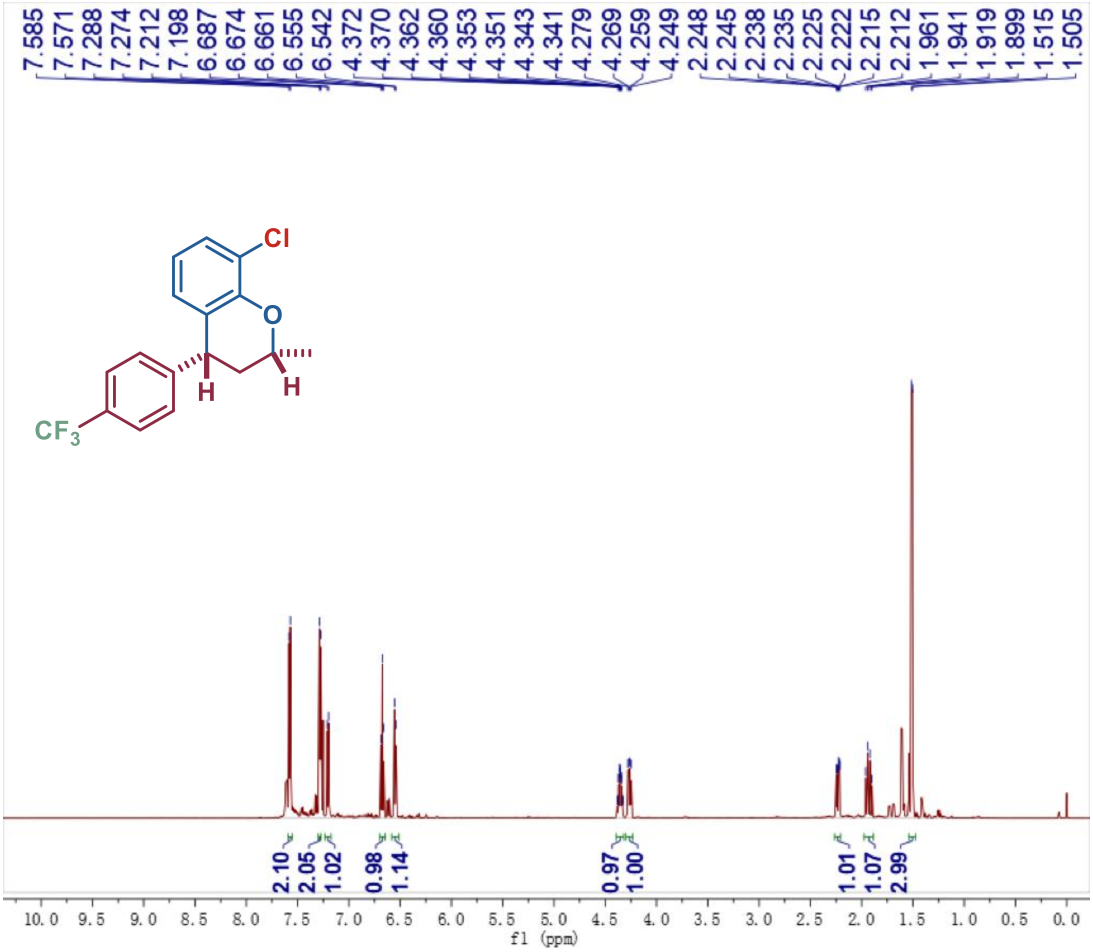

Fig. S193 ^{1}H NMR data of product 4aq.

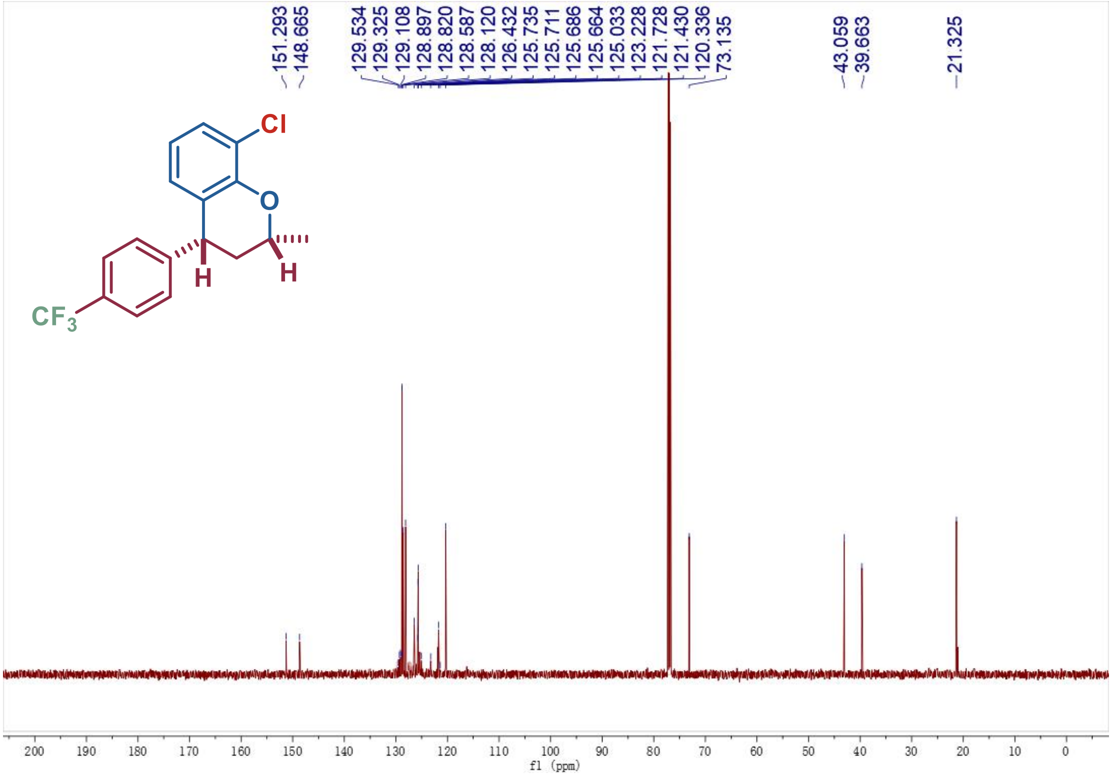

Fig. S194 ^{13}C NMR data of product 4aq.

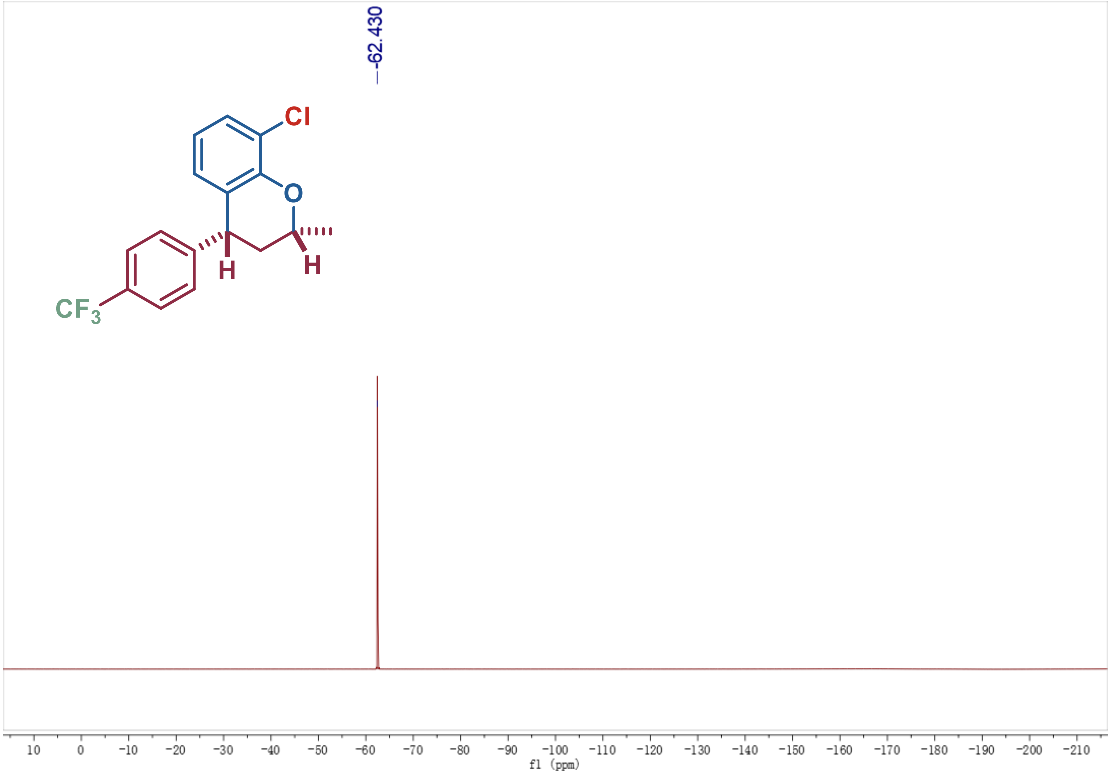

Fig. S195 ^{19}F NMR data of product 4aq.

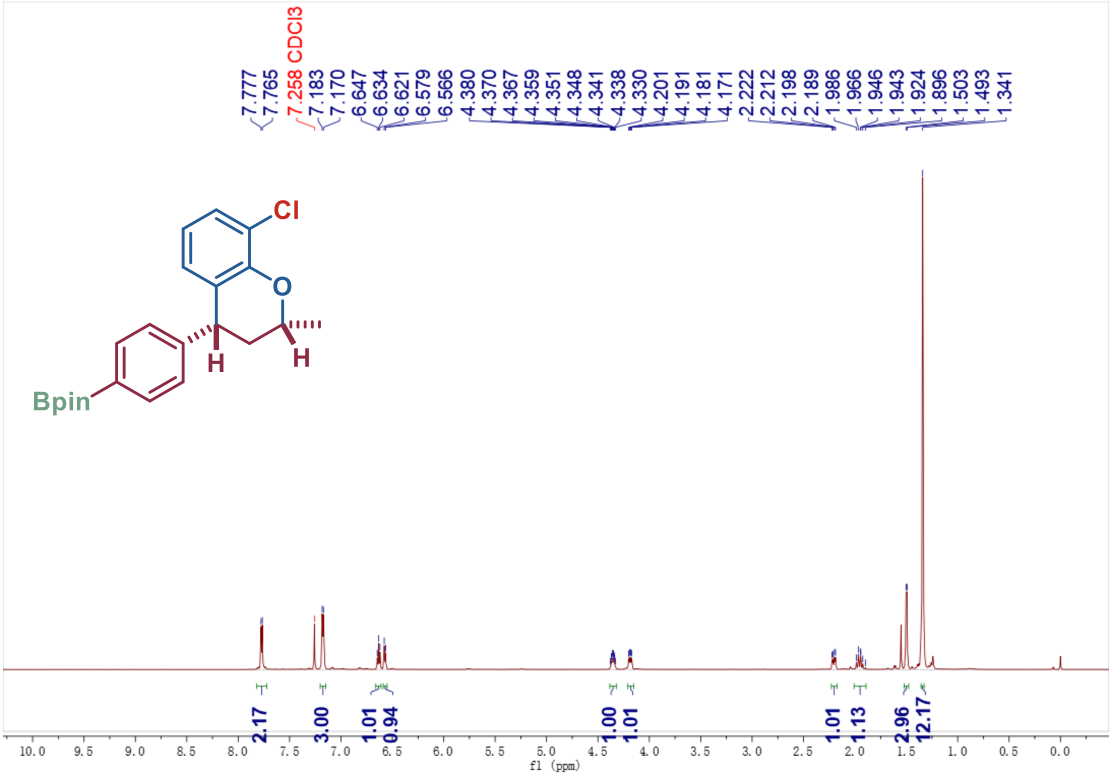

Fig. S196 ^{1}H NMR data of product 4ar.

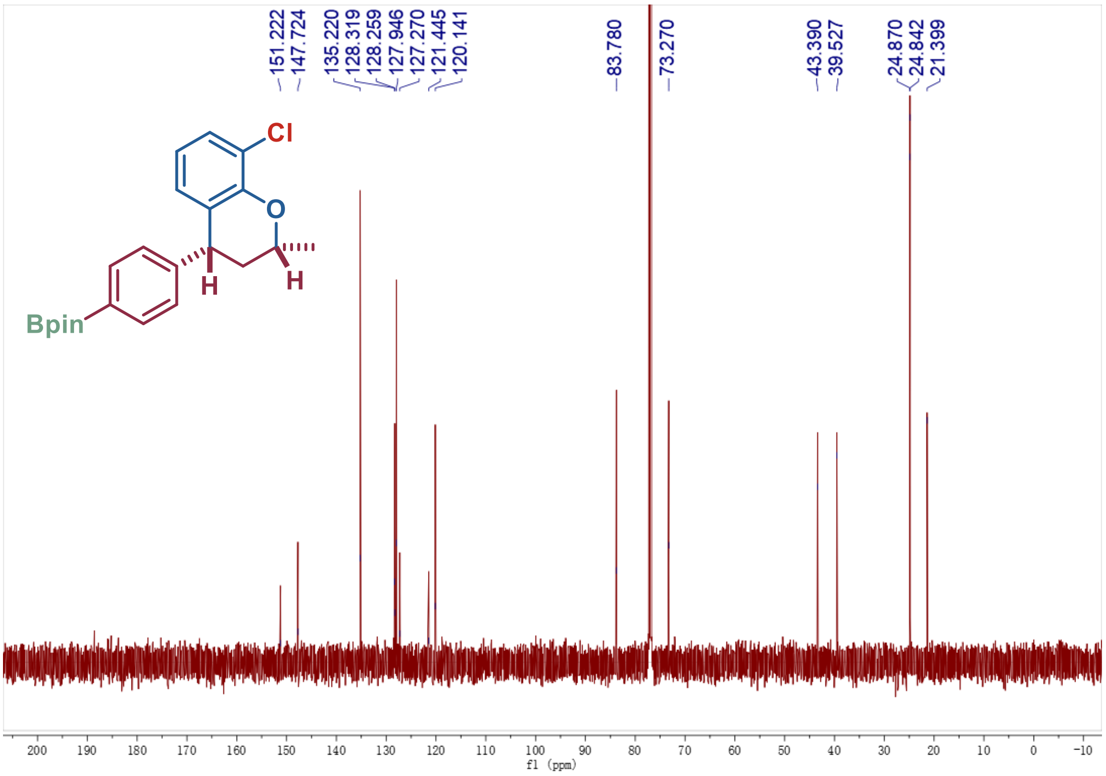

Fig. S197 ^{13}C NMR data of product 4ar.

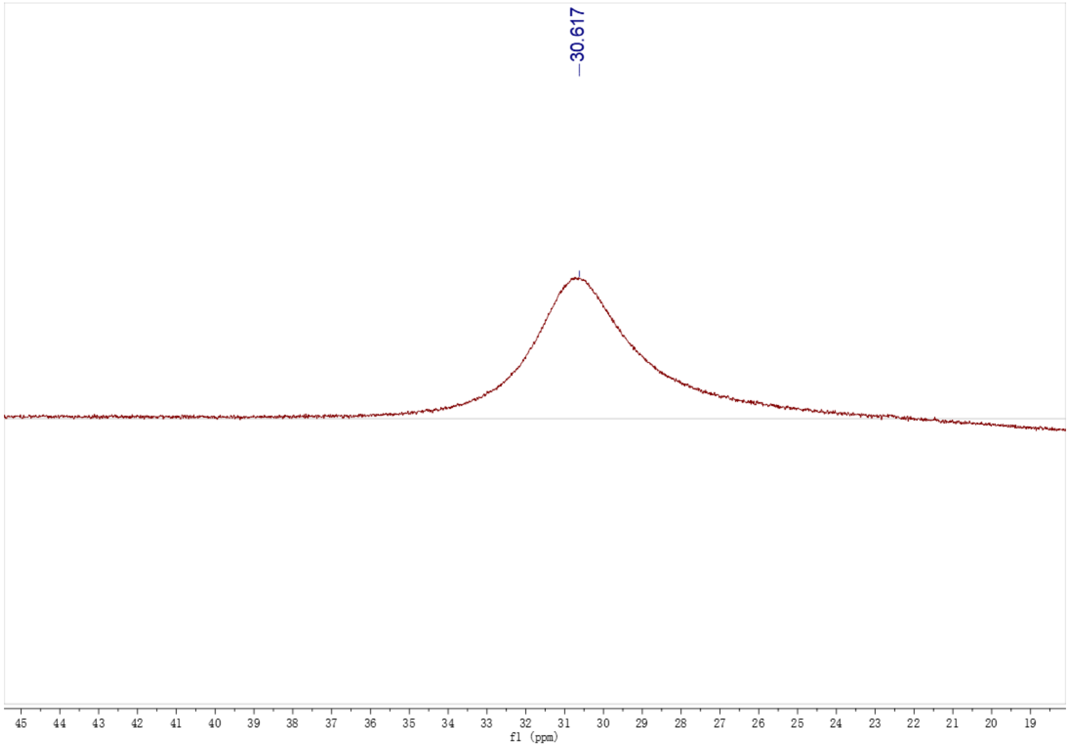

Fig. S198 ^{11}B NMR data of product 4ar.

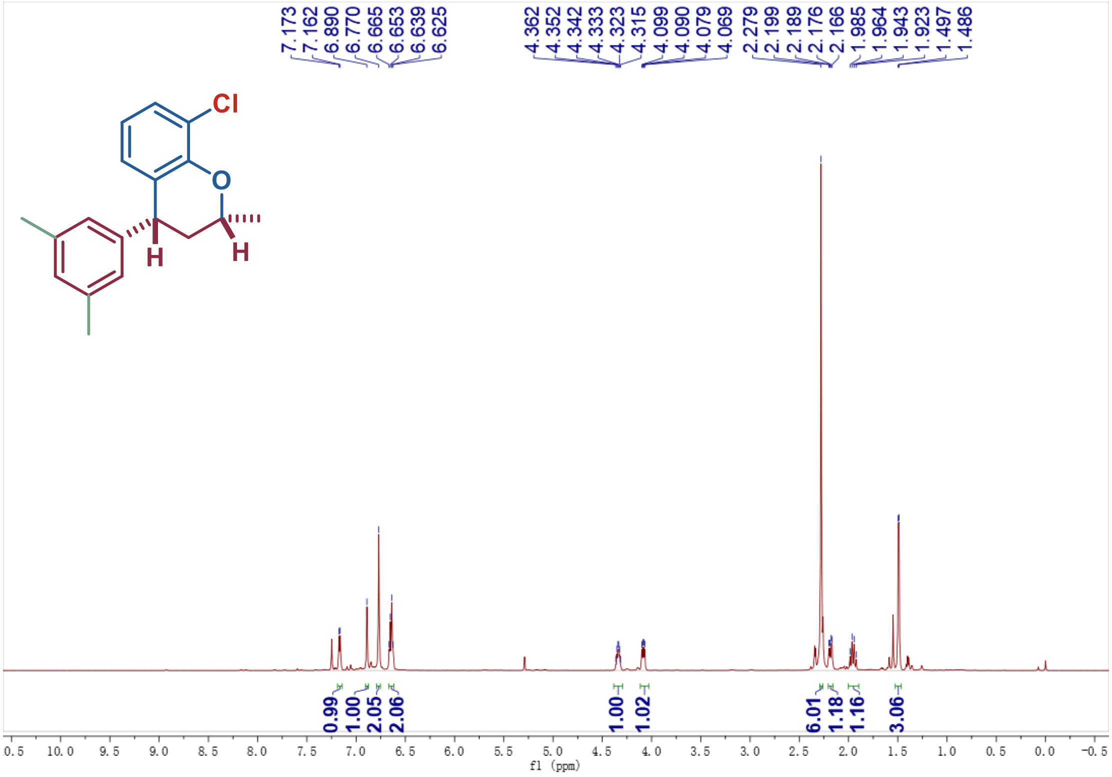

Fig. S199 ¹H NMR data of product 4as.

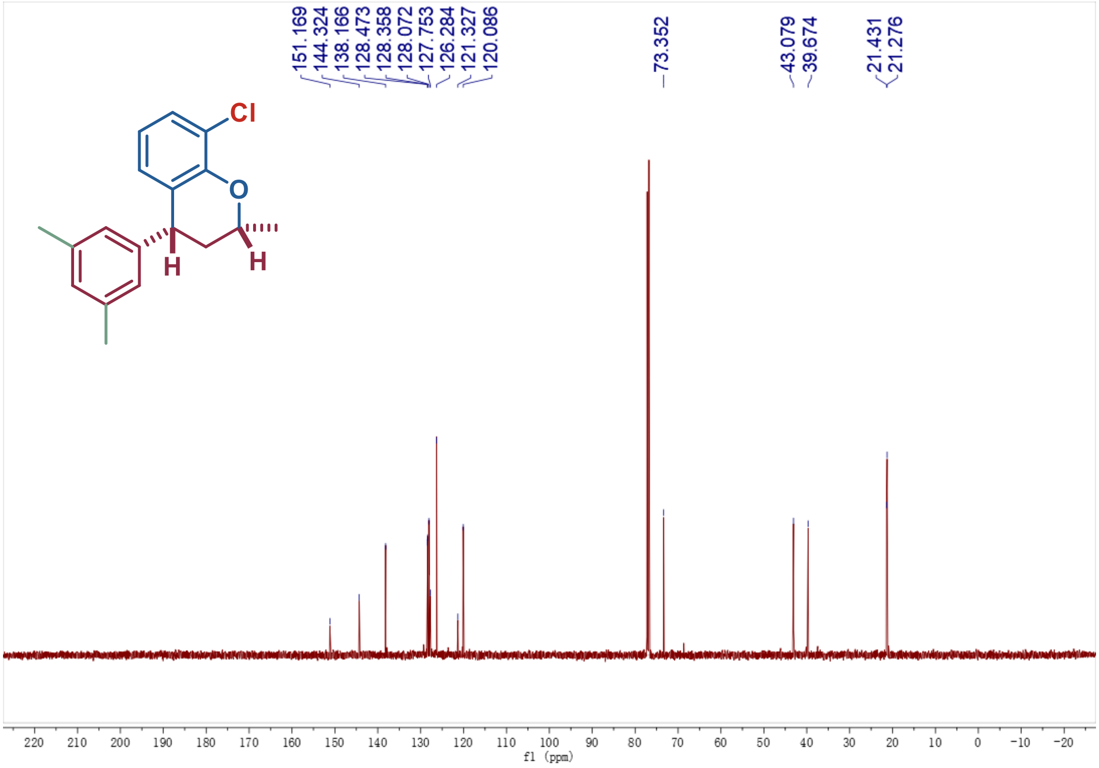

Fig. S200 ¹³C NMR data of product 4as.

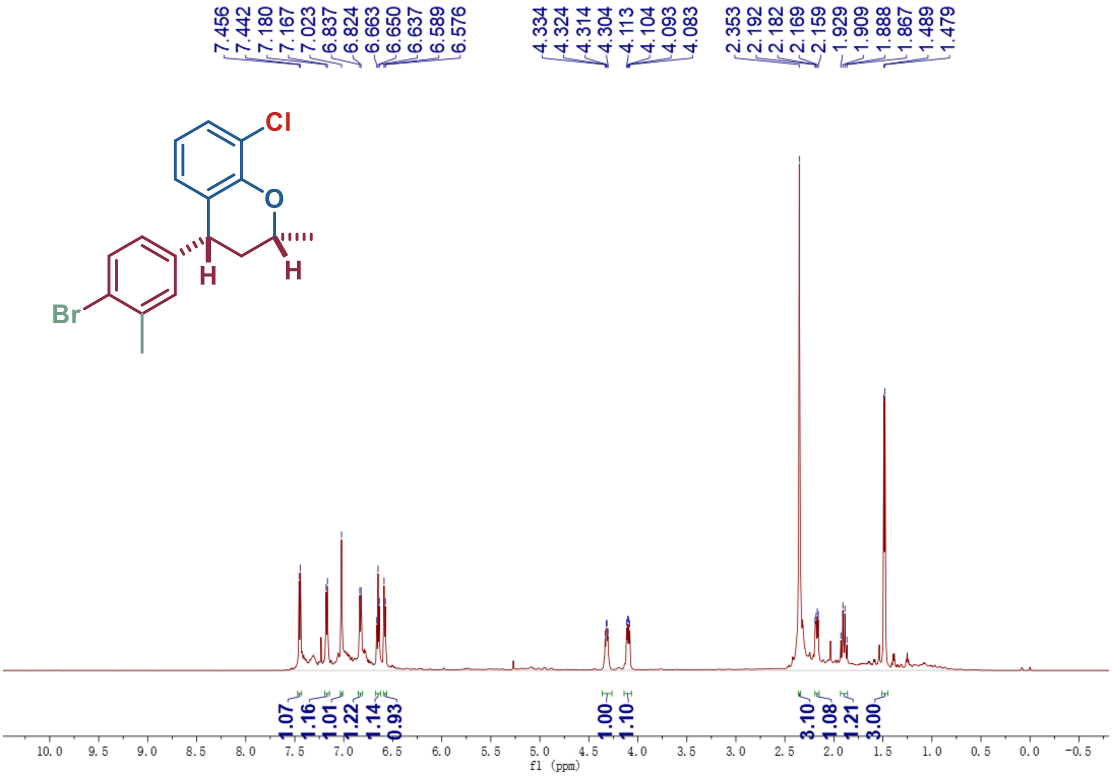

Fig. S201 ^{1}H NMR data of product 4at.

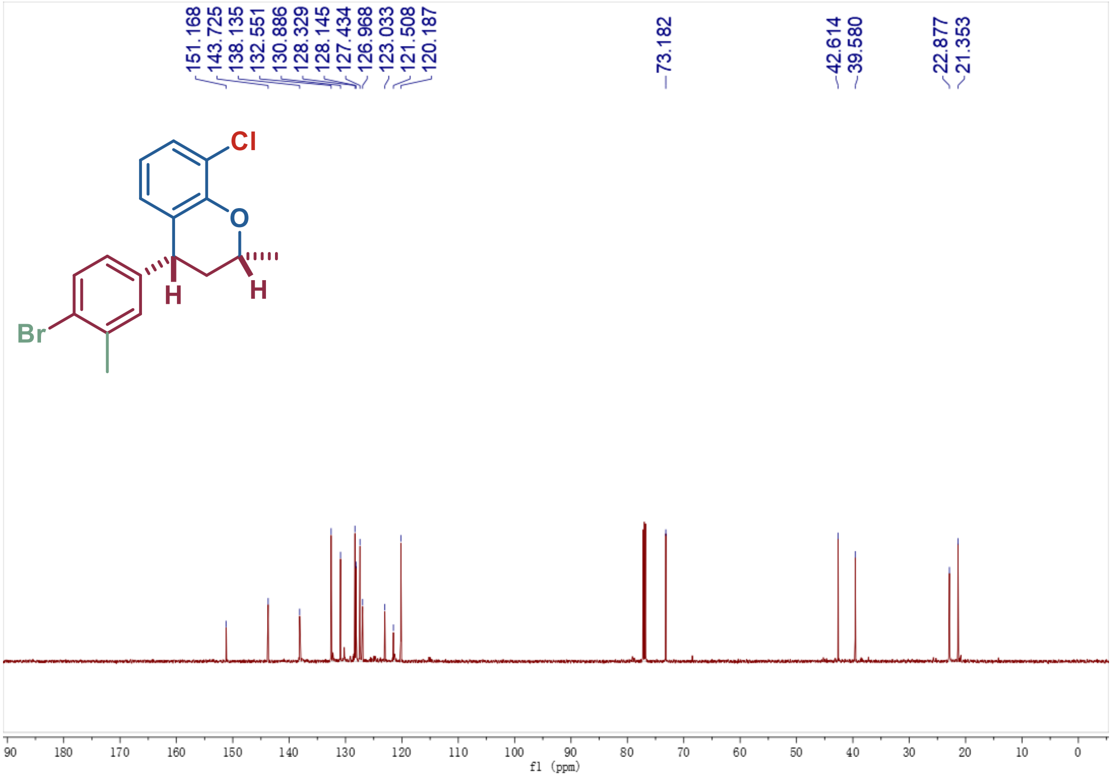

Fig. S202 ^{13}C NMR data of product 4at.

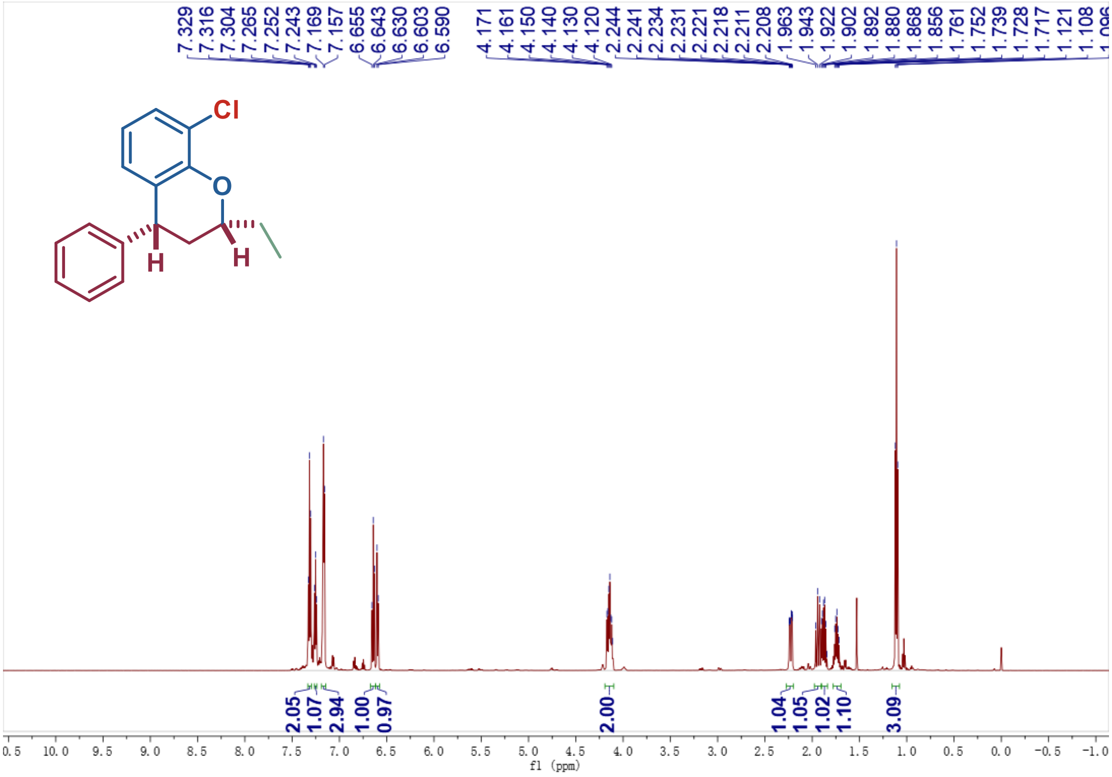

Fig. S203 ^{1}H NMR data of product 4au.

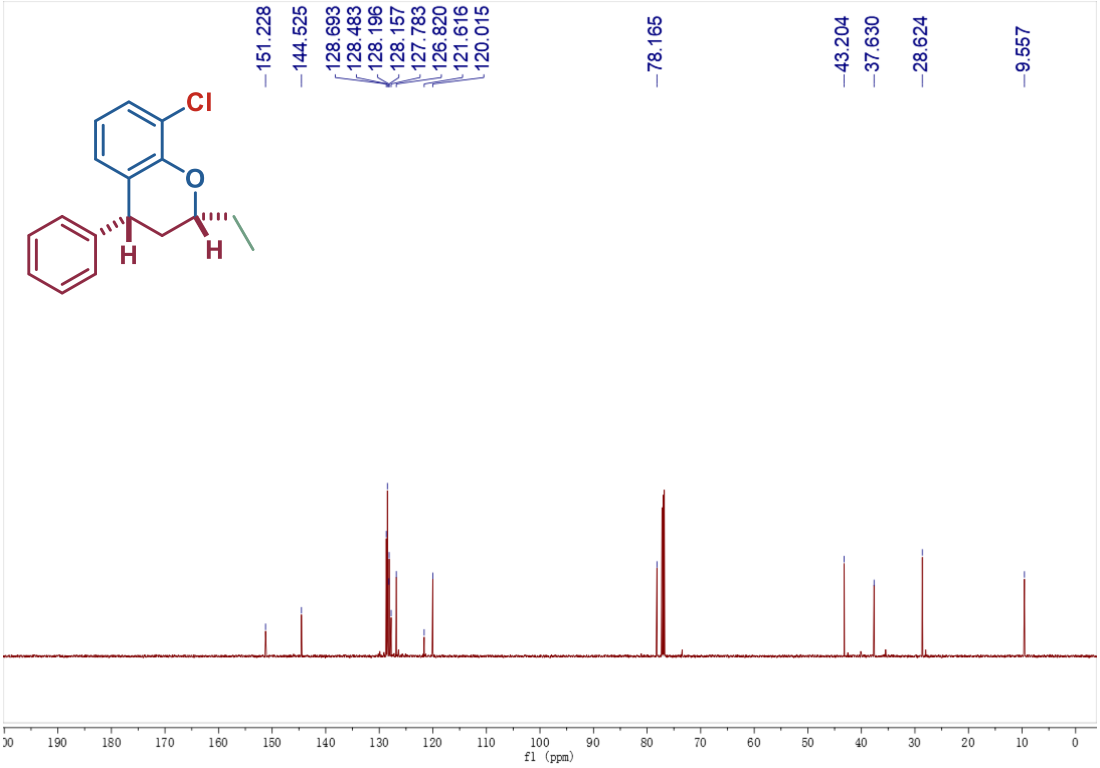

Fig. S204 ^{13}C NMR data of product 4au.

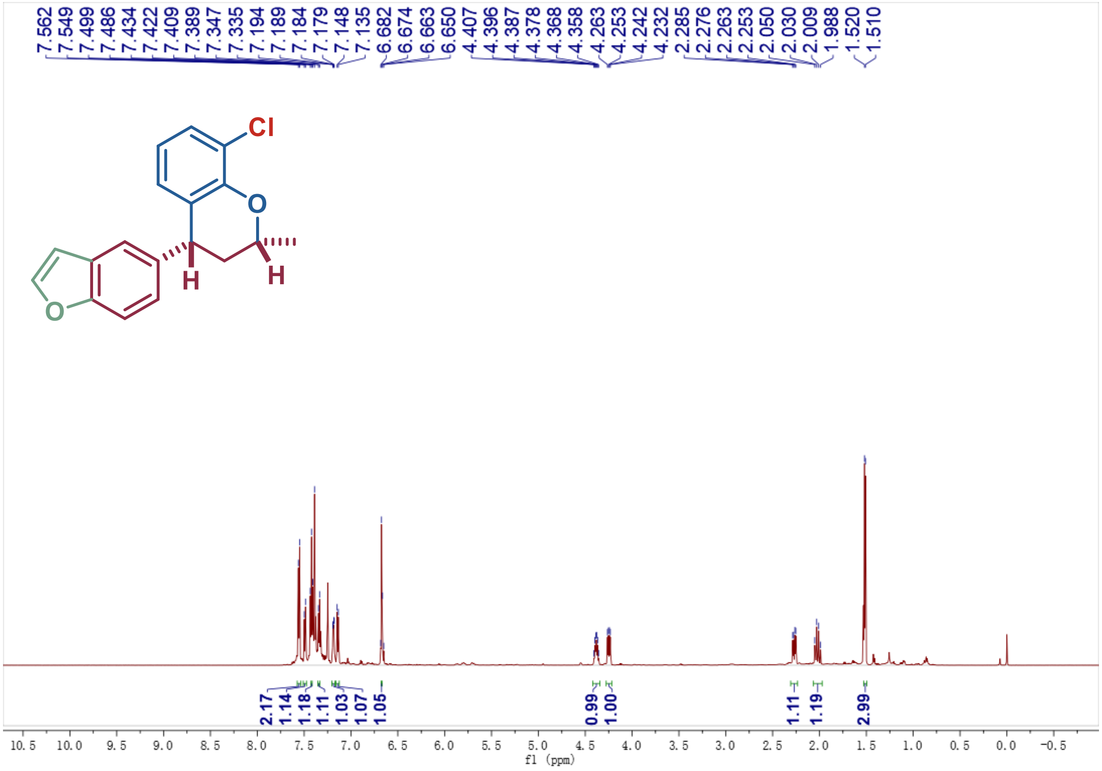

Fig. S205 ^{1}H NMR data of product 4av.

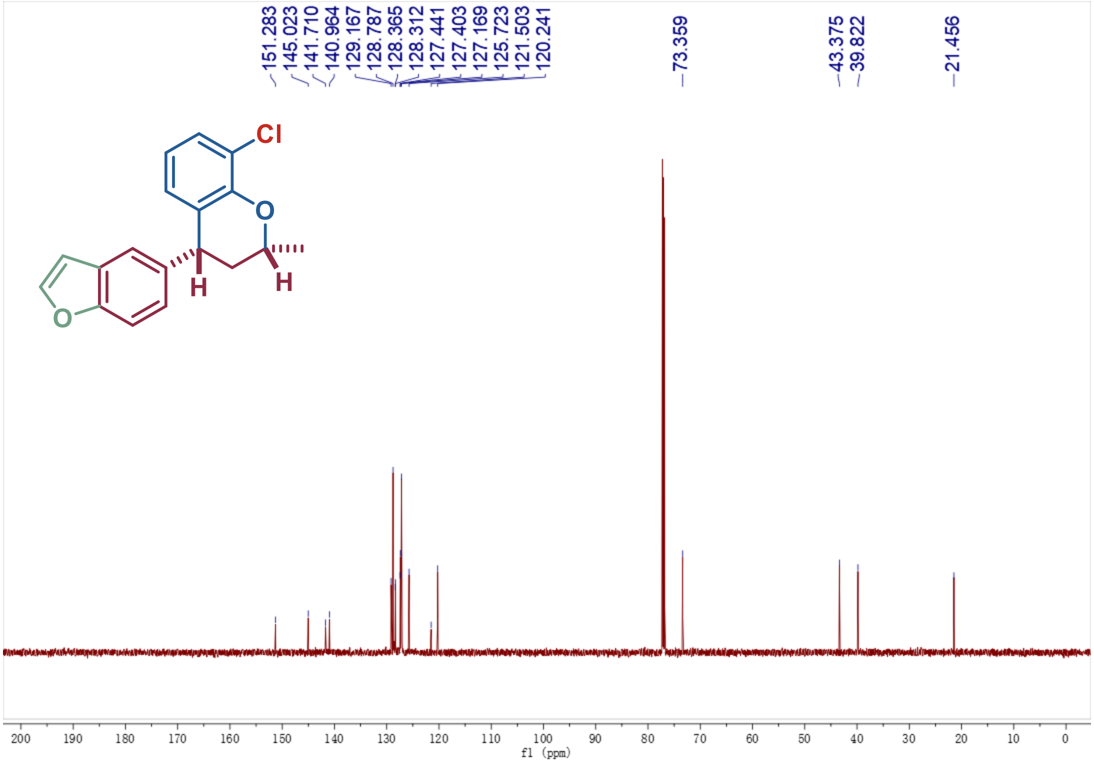

Fig. S206 ^{13}C NMR data of product 4av.

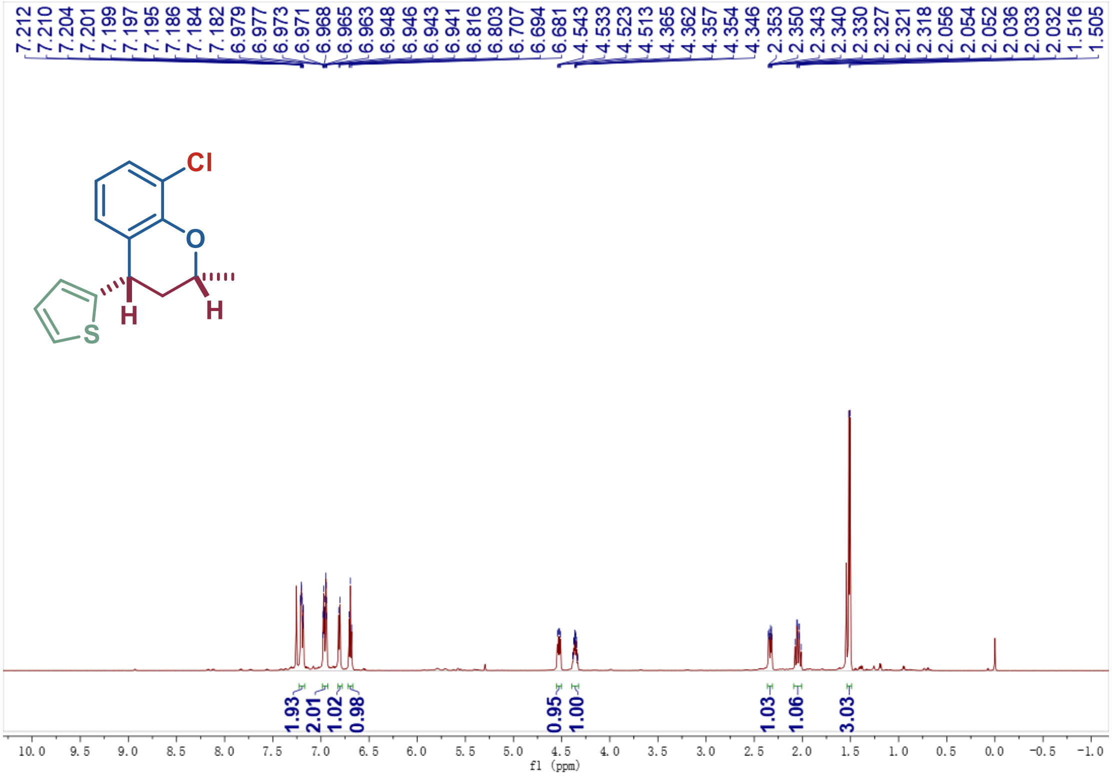

Fig. S207 ^{1}H NMR data of product 4aw.

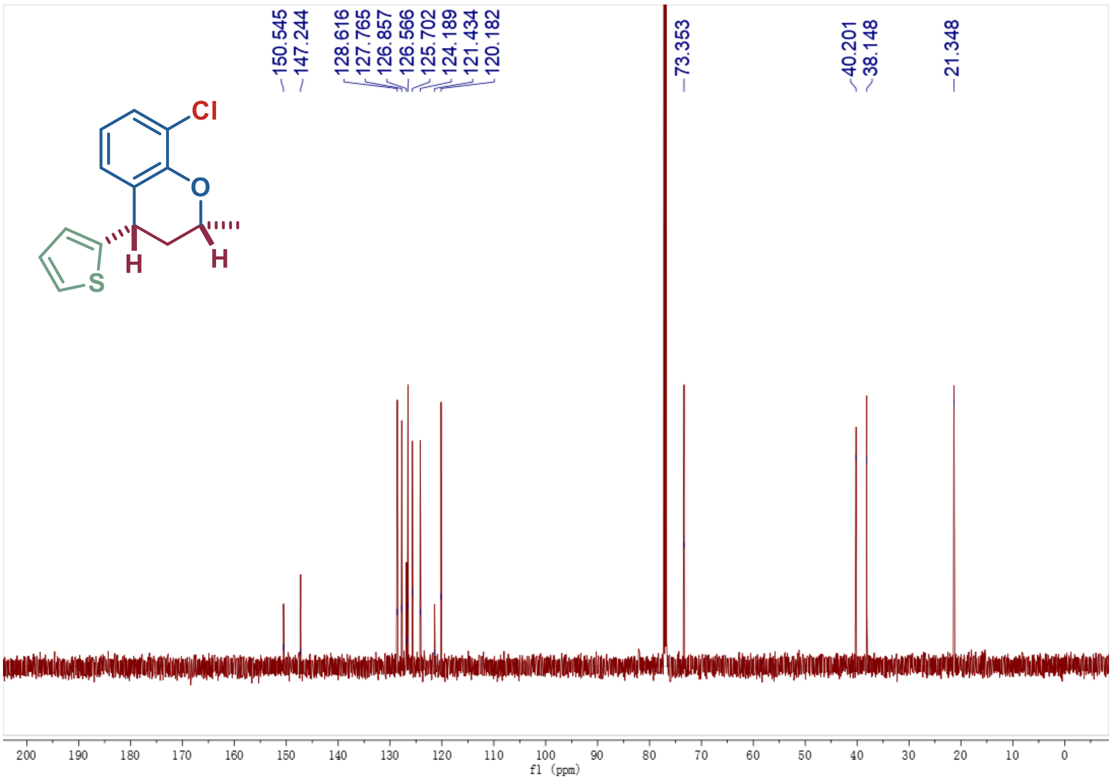

Fig. S208 ^{13}C NMR data of product 4aw.

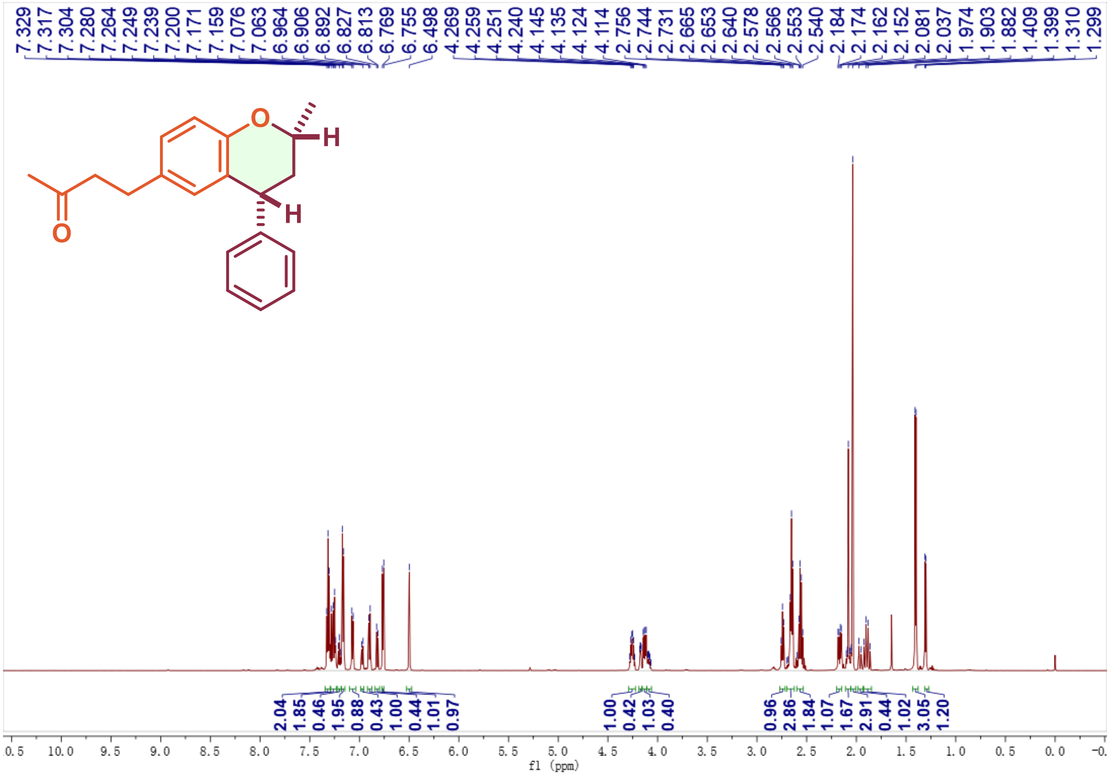

Fig. S209 ^{1}H NMR data of product 5a.

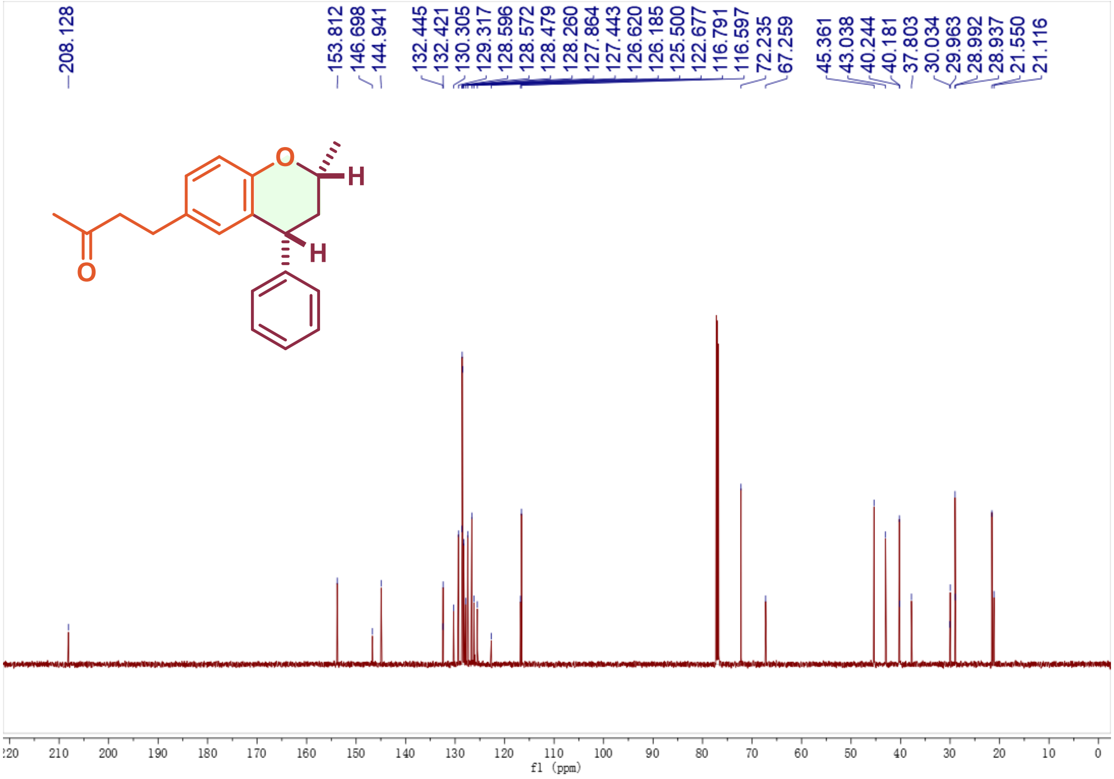

Fig. S210 ^{13}C NMR data of product 5a.

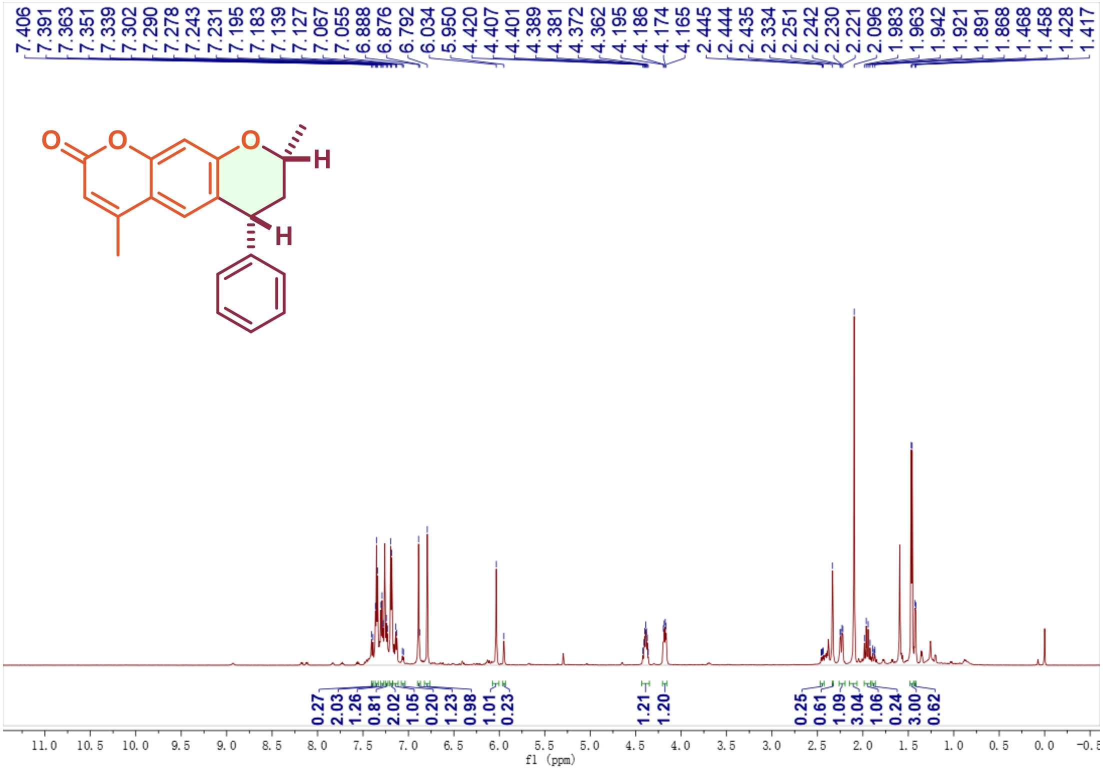

Fig. S211 ^{1}H NMR data of product 5b.

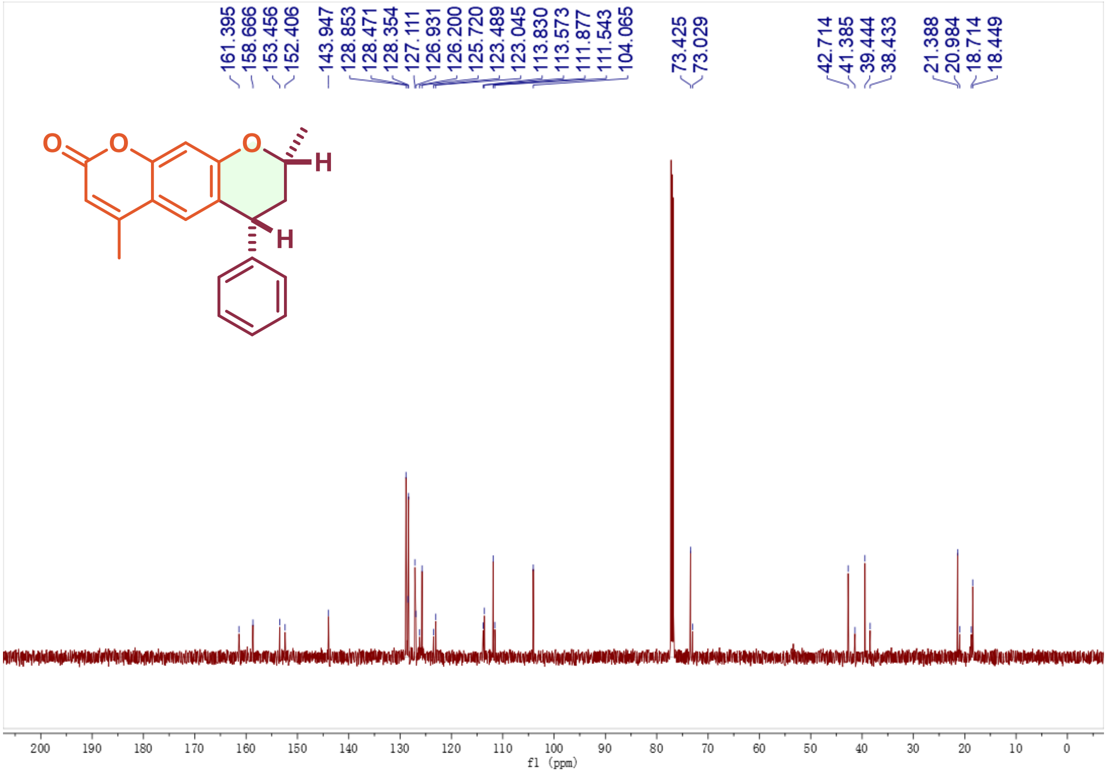

Fig. S212 ^{13}C NMR data of product 5b.

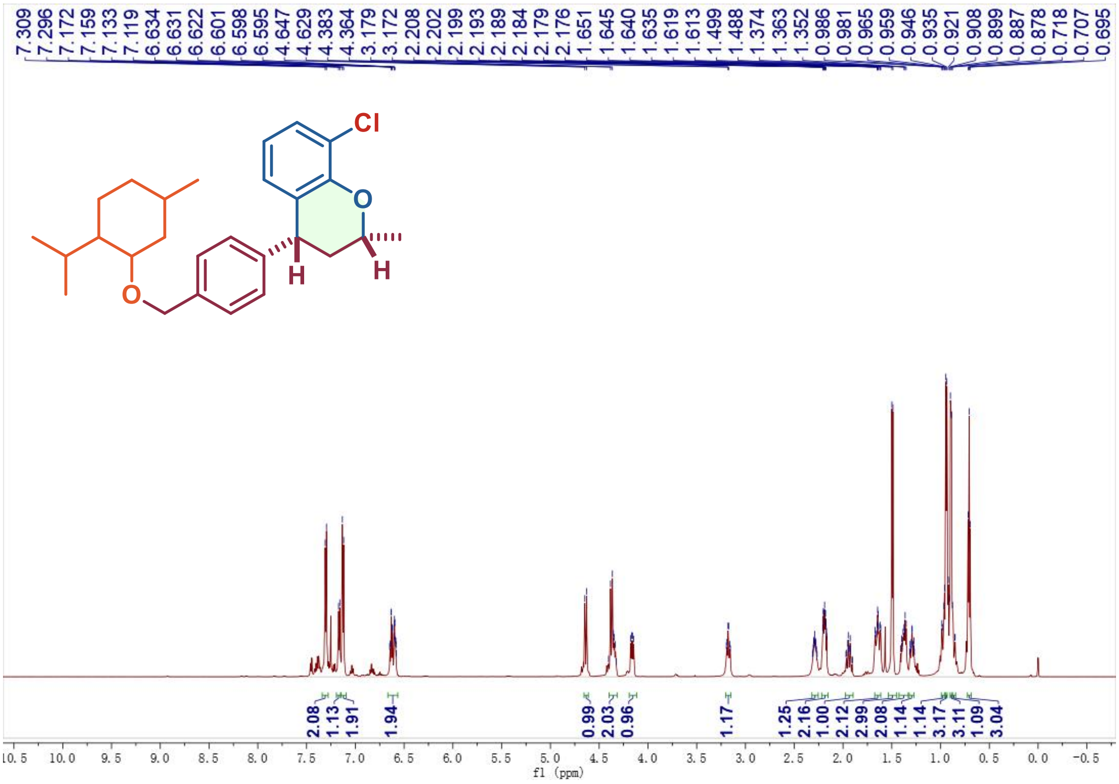

Fig. S213 ^{1}H NMR data of product 5c.

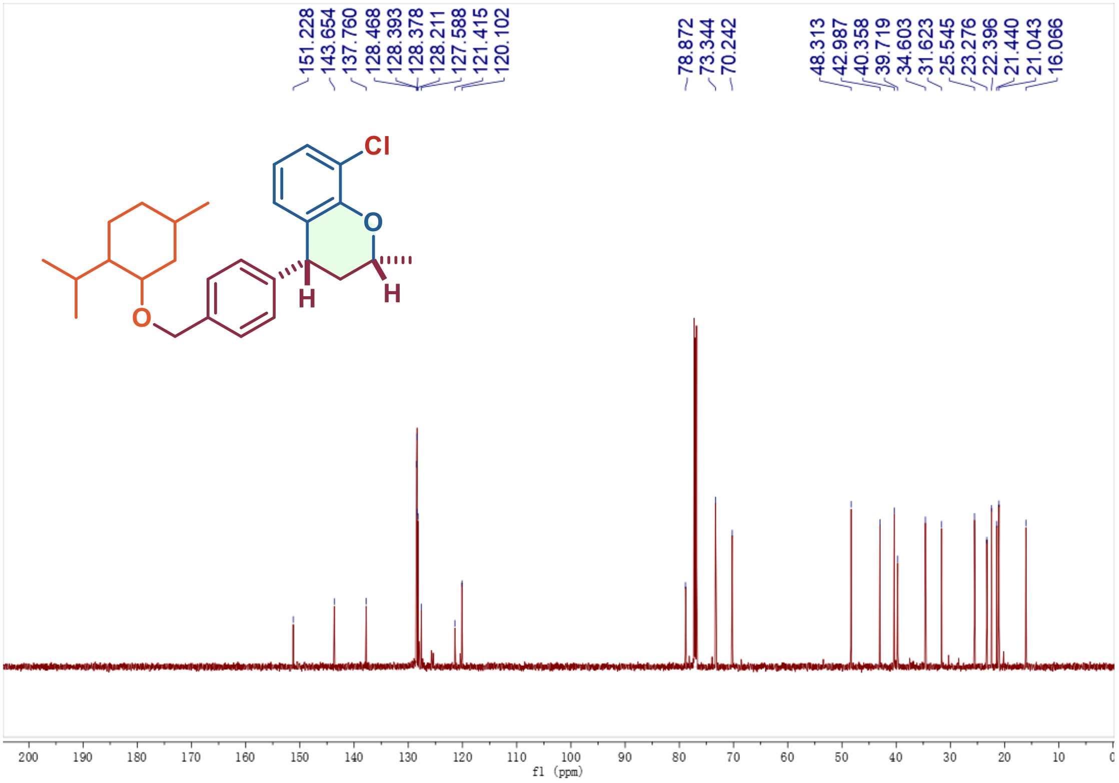

Fig. S214 ^{13}C NMR data of product 5c.

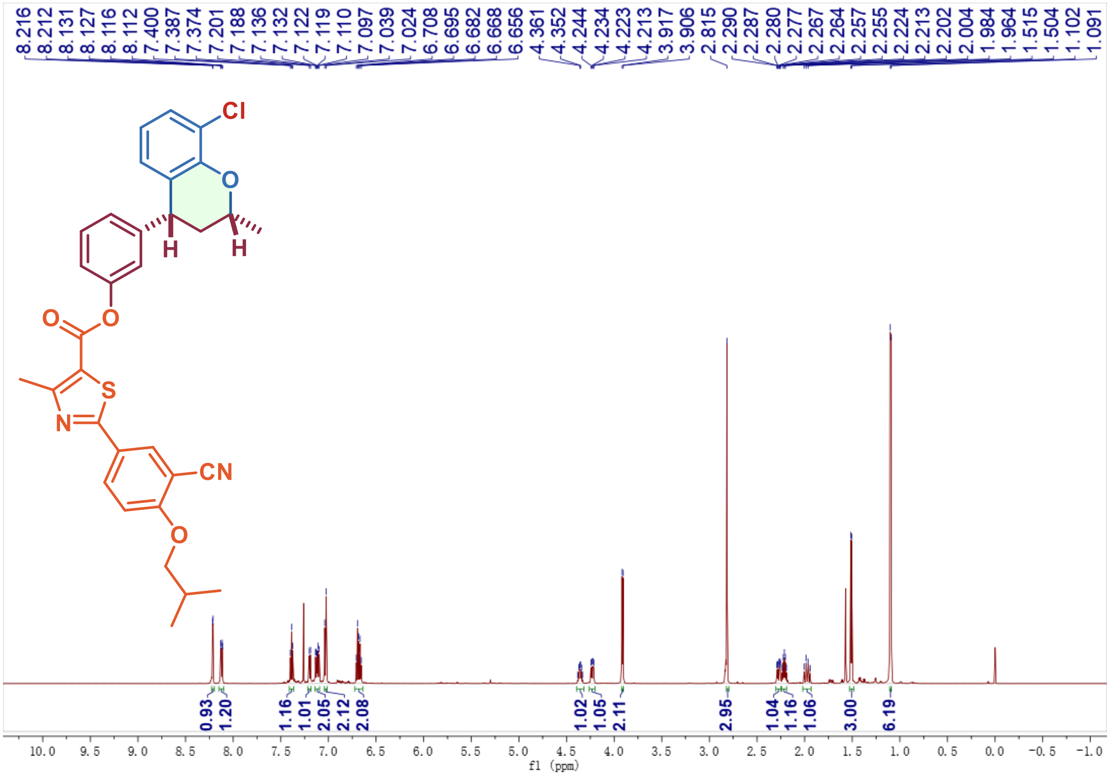

Fig. S215 ^{1}H NMR data of product 5d.

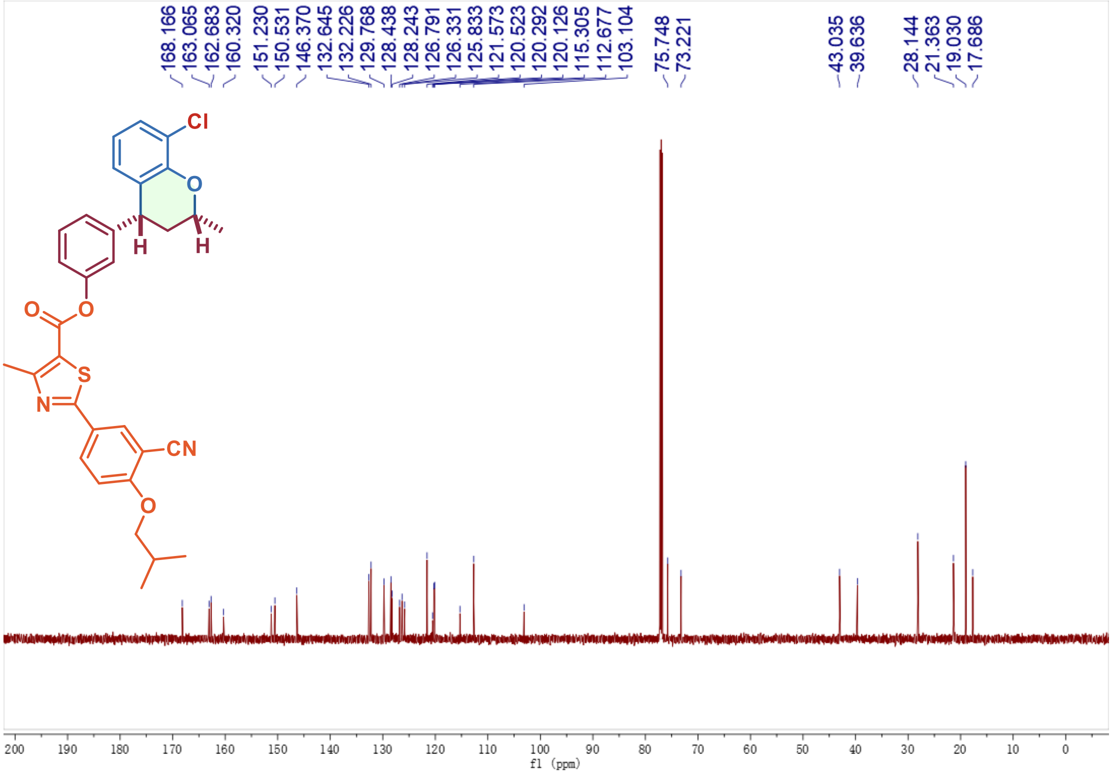

Fig. S216 ^{13}C NMR data of product 5d.

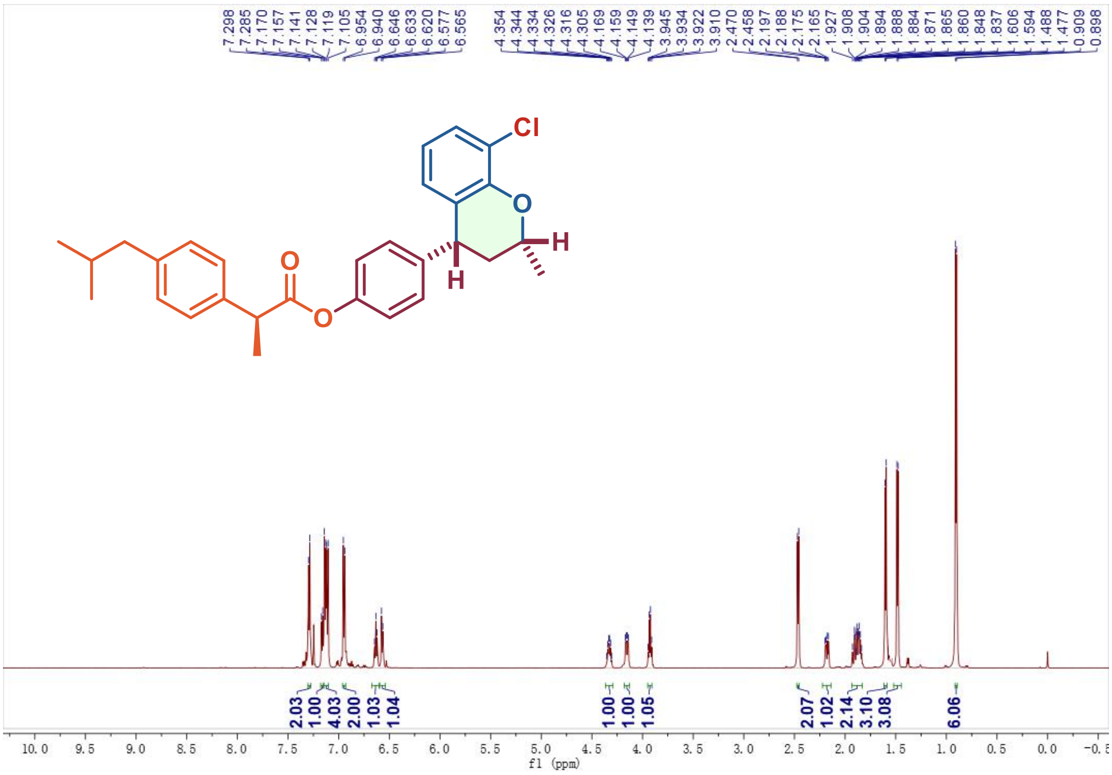

Fig. S217 ^{1}H NMR data of product 5e.

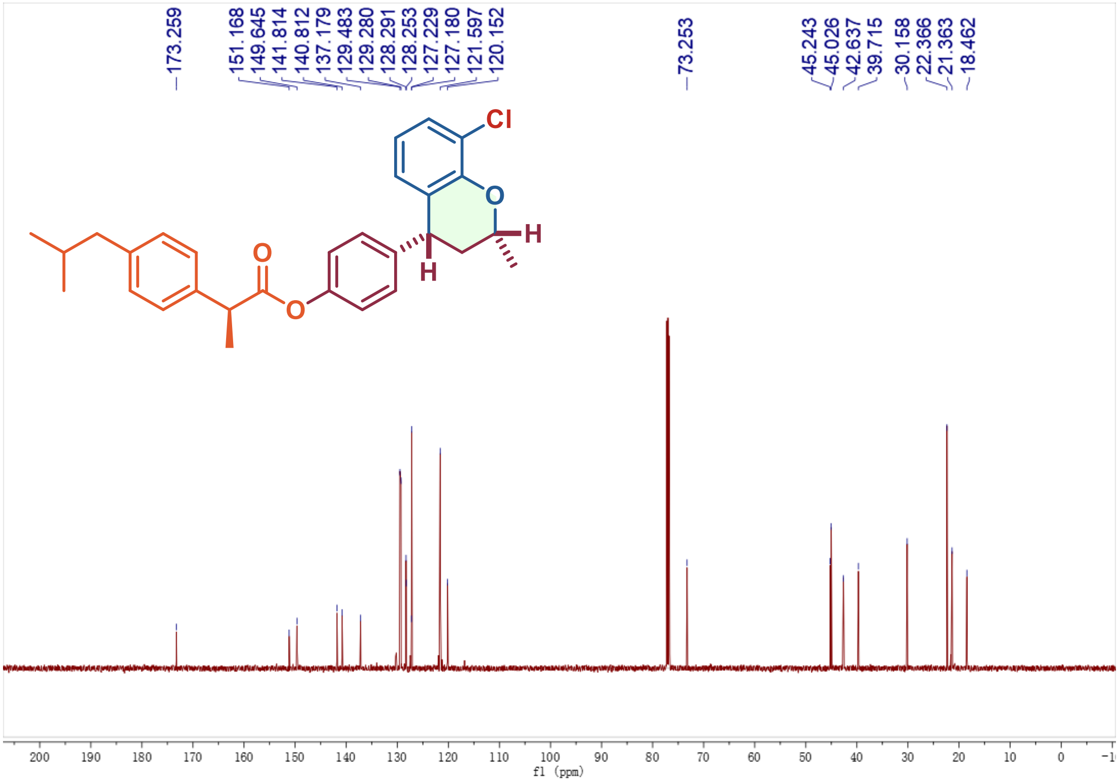

Fig. S218 ^{13}C NMR data of product 5e.

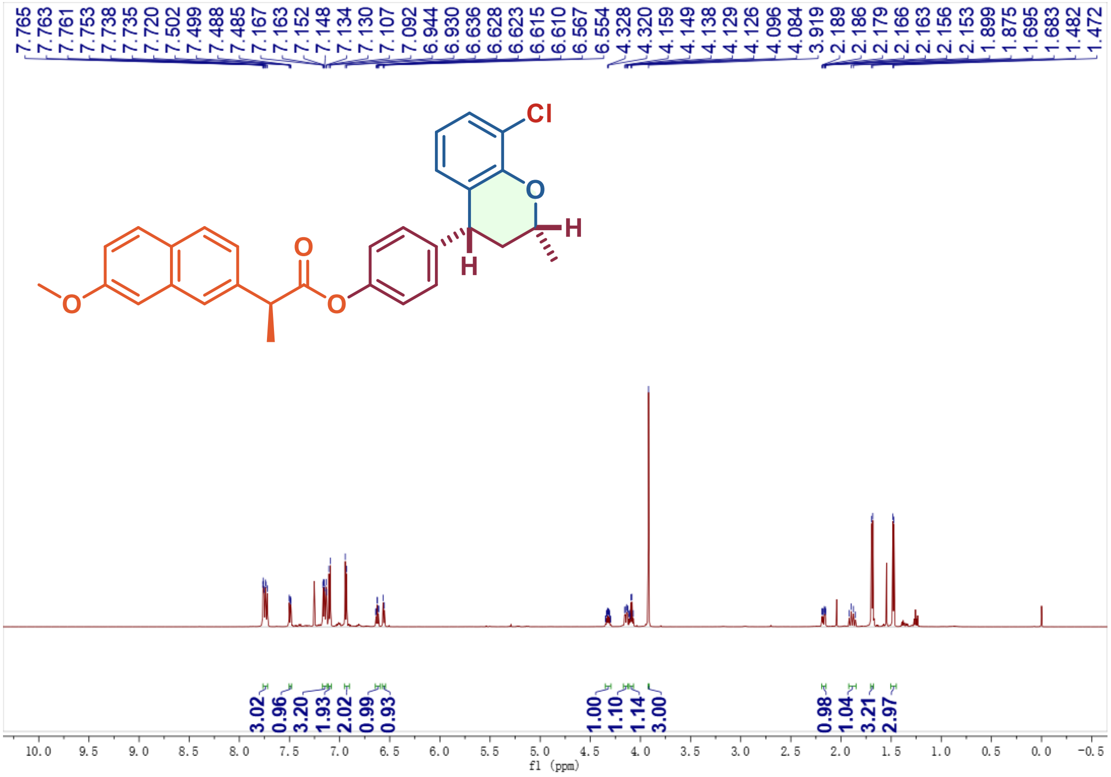

Fig. S219 ^{1}H NMR data of product 5f.

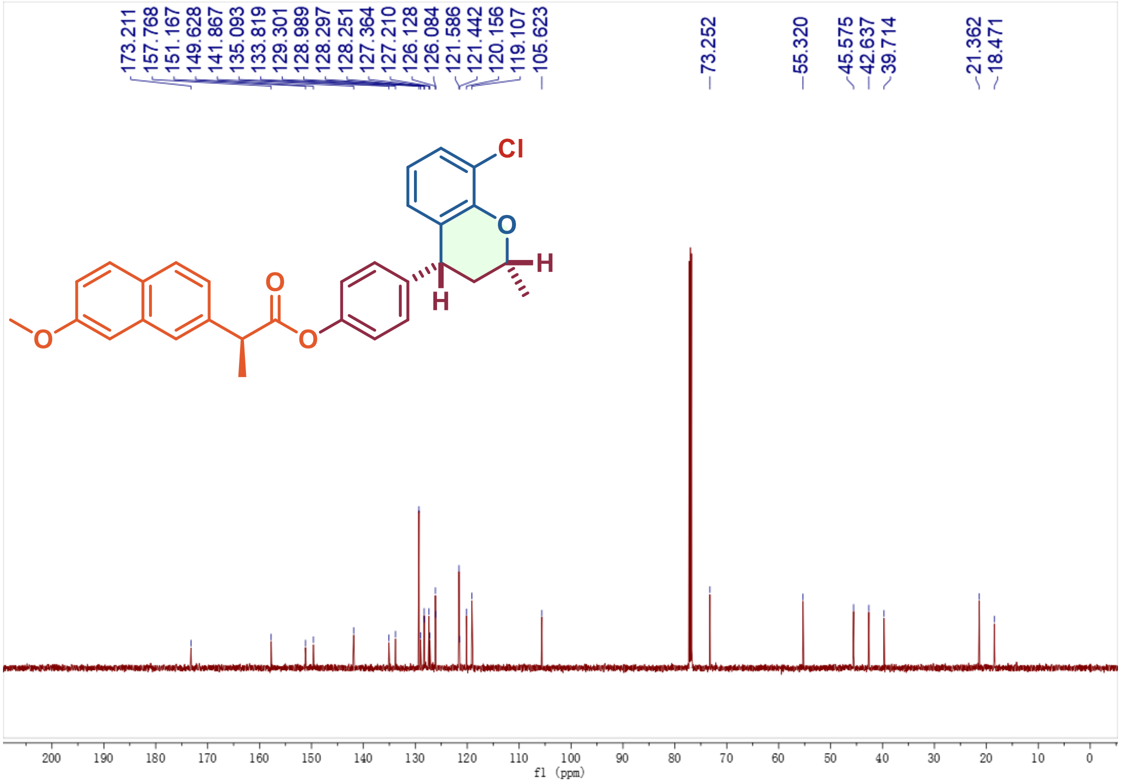

Fig. S220 ^{13}C NMR data of product 5f.
